# Protein posttranslational modifications in health and diseases: Functions, regulatory mechanisms, and therapeutic implications

**DOI:** 10.1002/mco2.261

**Published:** 2023-05-02

**Authors:** Qian Zhong, Xina Xiao, Yijie Qiu, Zhiqiang Xu, Chunyu Chen, Baochen Chong, Xinjun Zhao, Shan Hai, Shuangqing Li, Zhenmei An, Lunzhi Dai

**Affiliations:** ^1^ Department of Endocrinology and Metabolism General Practice Ward/International Medical Center Ward General Practice Medical Center and National Clinical Research Center for Geriatrics State Key Laboratory of Biotherapy West China Hospital, Sichuan University Chengdu China

**Keywords:** aging, cancers, metabolic diseases, neurodegenerative diseases, protein posttranslational modifications, targeted therapy

## Abstract

Protein posttranslational modifications (PTMs) refer to the breaking or generation of covalent bonds on the backbones or amino acid side chains of proteins and expand the diversity of proteins, which provides the basis for the emergence of organismal complexity. To date, more than 650 types of protein modifications, such as the most well‐known phosphorylation, ubiquitination, glycosylation, methylation, SUMOylation, short‐chain and long‐chain acylation modifications, redox modifications, and irreversible modifications, have been described, and the inventory is still increasing. By changing the protein conformation, localization, activity, stability, charges, and interactions with other biomolecules, PTMs ultimately alter the phenotypes and biological processes of cells. The homeostasis of protein modifications is important to human health. Abnormal PTMs may cause changes in protein properties and loss of protein functions, which are closely related to the occurrence and development of various diseases. In this review, we systematically introduce the characteristics, regulatory mechanisms, and functions of various PTMs in health and diseases. In addition, the therapeutic prospects in various diseases by targeting PTMs and associated regulatory enzymes are also summarized. This work will deepen the understanding of protein modifications in health and diseases and promote the discovery of diagnostic and prognostic markers and drug targets for diseases.

## Graphical abstract

The reversible and irreversible protein posttranslational modifications, such as acetylation, methylation, phosphorylation, ubiquitination, glycosylation, SUMOylation, and redox modifications, are essential regulators in organisms and cells. This work systematically summarizes the features, regulatory mechanisms, substrates, functions, and related treatments of protein modifications and will deepen the understanding of protein modifications in health and diseases.

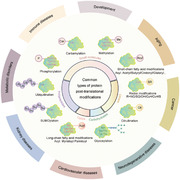

## 1 INTRODUCTION

Protein posttranslational modifications (PTMs) refer to the breaking or generation of covalent bonds on the backbones or amino acid side chains of proteins and are also called covalent modifications.^1^, ^2^, ^3^, ^4^ By covalently modifying proteins, cells, tissues, and biological individuals expand the chemical composition and information of twenty amino acids. PTMs escape from genetic confinement in nature.^5^ Rapid changes in gene sequences on evolutionary timescales are not suitable for organisms to develop and survive.^6^ PTMs can dynamically change the properties of amino acids according to the requirements on developmental 00and physiological timescales.^7^ Consequently, numerous PTMs lead to an explosion in the number of proteins with potential molecular states, which provides the basis for the emergence of organismal complexity.^8^ More than 650 types of protein modifications, such as the most well‐known phosphorylation, acetylation, methylation, ubiquitination, glycosylation, acylation, cysteine oxidation, SUMOylation, ADP‐ribosylation, neddylation, citrullination, and carbamylation, have been described to date (http://www.uniprot.org/docs/ptmlist.txt), and the inventory is still increasing.^9^, ^10^

**FIGURE 1:**
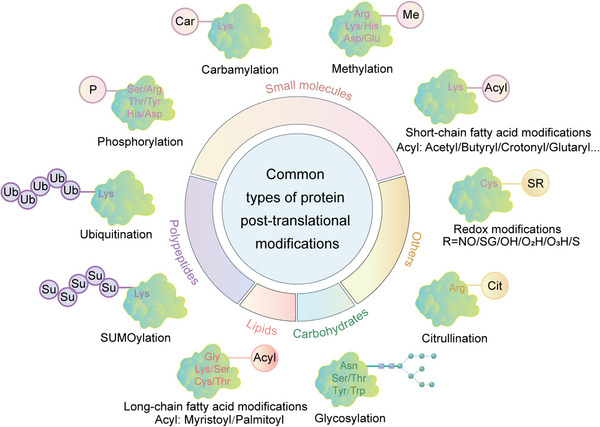
Common types of protein posttranslational modifications. Small molecules, lipids, carbohydrates, and polypeptides can be added to amino acid side chains to form modifications. In addition, changes in the chemical properties of amino acids are also common modifications, such as citrullination.

The PTM process is divided into the following classes (Figure 1). First, modifiers such as small chemicals and complex biomolecules are added to the amino acid side chains. Small chemicals such as phosphate, sugar, methyl group, and acetyl group are usually electrophilic. In contrast, the amino acid side chains that receive modifiers are usually rich in electrons and act as nucleophiles during the modification process, such as lysine and cysteine side chains.^11^ Second, there are changes in the chemical properties of amino acids, such as deamination, deamidation, citrullination, and oxidation.^12^ Notably, some types of redox modifications can also be recognized as the addition of small chemicals onto the side chain of cysteine such as S‐nitrosylation (SNO) and S‐glutathionylation. Third, the cleavage of protein backbone. This process can be conducted by enzyme catalysis or by protein autocatalysis. The cleavage process controls protein localization in or around the cell, protein activity, and protein turnover.^11^ Most PTMs are dynamically reversible, and the addition and removal of these PTMs are enzymatically regulated.^13^ These protein modifications occur faster than the synthesis of new proteins, which allows cells or organisms to respond rapidly to changes in the surrounding environment,^14^ making the PTM process essential for signal transduction and life processes.^15^ PTMs can occur at various stages of a protein's “life cycle.” New proteins can be modified immediately after synthesis to mediate their folding into the correct structures,^16^ while stable proteins are modified in response to stimuli to trigger or block downstream signaling pathways.^17^

By changing protein conformation, activity, charges and stability and interactions with DNA, RNA, and other proteins within and between cells, PTMs ultimately alter the phenotypes and biological functions of cells^18^ and participate in the regulation of numerous cellular processes and pathways, such as cell cycle,^19^ cell differentiation,^20^ transcriptional regulation,^21^ cell metabolism,^17^ immunity,^22^ signal transduction,^23^ and autophagy.^24^ For example, phosphorylation is involved in cell signal transduction and the cell cycle^25^; acetylation and methylation are associated with transcriptional regulation and cell metabolism^26^, ^27^; glycosylation plays an important role in protein folding and cell adhesion^28^; and ubiquitination regulates protein degradation and localization.^29^

Abnormal PTMs may cause changes in protein properties and loss of protein biological functions, directly participating in the occurrence and development of diseases.^30^ For example, Tau hyperphosphorylation usually leads to neurodegenerative diseases such as Alzheimer's disease (AD).^31^ Low palmitoylation of the mutant huntingtin (HTT) protein in the nervous system results in increased neurotoxicity and greater susceptibility to aggregate formation, which may induce Huntington's disease (HD).^32^ Protein acetylation is a critical regulator of insulin sensitivity and metabolism, global SIRT1 overexpression can improve insulin sensitivity, glucose tolerance, and hepatic steatosis.^33^ The disorder of glucose and lipid metabolism in type 2 diabetes mellitus (T2DM) may be related to the malfunction of key enzymes caused by malonylation.^34^ In cancers, many signaling pathways are in a state of continuous activation and are mainly conducted through a cascade of reversible phosphorylation of different proteins, such as the MAPK, JAK/STAT, and PI3K/AKT signaling pathways.^35^ Moreover, the continuous ubiquitination of tumor suppressors causes protein degradation and functional loss, also contributing to the development of various tumors.^36^ In addition to nonhistone modifications, the roles of histone modifications in health and diseases are also very important. Ataxin‐3 protein causes spinocerebellar ataxia by altering histone acetylation profiles and inducing transcriptional defects.^37^ Loss of H4K16ac and H4K20me3 are key hallmark of human cancer.^38^ In hematological malignancies, hypermethylation frequently occurs at H3K79^39^ and H3K4.^40^ Thus, deciphering PTMs is of great significance for the prevention, diagnosis, and treatment of diseases.^19^

In this review, we systematically examine the various PTMs, including phosphorylation, acetylation, acylation with short‐ or long‐chain fatty acids, methylation, ubiquitination, SUMOylation, glycosylation, citrullination, carbamylation, cysteine oxidation, and other modifications. We discuss their characteristics, regulatory mechanisms, and functions in both health and diseases, including development and aging, immune diseases, metabolic disorders, cancers, neurodegenerative diseases, and cardiovascular diseases (CVDs). Moreover, the therapeutic prospects in various diseases by targeting PTMs and associated regulatory enzymes are also summarized.

## 2 PHOSPHORYLATION

**FIGURE 2:**
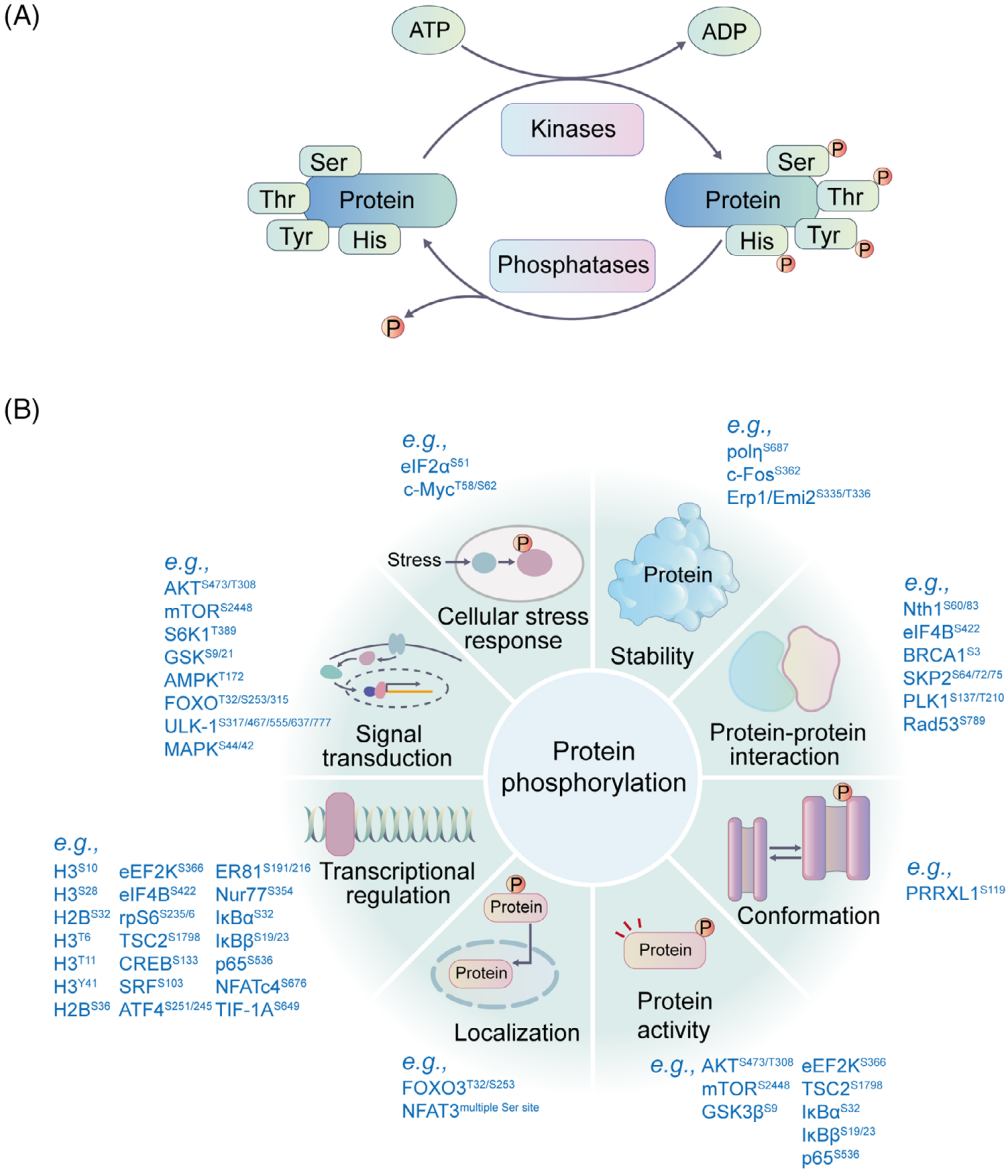
The phosphorylation process and the potential functions of phosphorylation. (A) The phosphorylation and dephosphorylation process. Phosphorylation is catalyzed by kinases, and dephosphorylation is mediated by phosphatases. Most phosphorylation events occur on serine, threonine, and tyrosine residues. (B) Representative functions of protein phosphorylation are shown. Protein phosphorylation extensively affects cellular signal transduction, protein stability, activity, localization, conformation, protein–protein interactions, gene transcription, and so on. Representative phosphosites with related functions are shown.

Protein phosphorylation, formed by adding a phosphate group from ATP to the side chains of amino acids by kinases, usually turns hydrophobic nonpolar proteins into hydrophilic polar proteins. Phosphorylation is a reversible PTM, and the reverse process of phosphorylation is called dephosphorylation catalyzed by phosphatases (Figure 2A).^41^ Phosphorylation modifications occur most commonly on serine, followed by threonine and tyrosine residues, accounting for 86.4, 11.8, and 1.8%, respectively.^42^ However, it is important to note that kinases can also act on the side chains of other amino acids, such as cysteine, lysine, histidine, arginine, aspartic acid, and glutamic acid, although with reduced frequency.^43^ Histidine and aspartate phosphorylation are much less stable than other modifications.^42^ The phosphosites can be recognized and bound by specific phosphorylation‐binding proteins.^44^ Therefore, the protein phosphorylation system consists of kinases, phosphatases, phosphorylation substrates, and phosphorylation‐binding proteins.^45^

**FIGURE 3:**
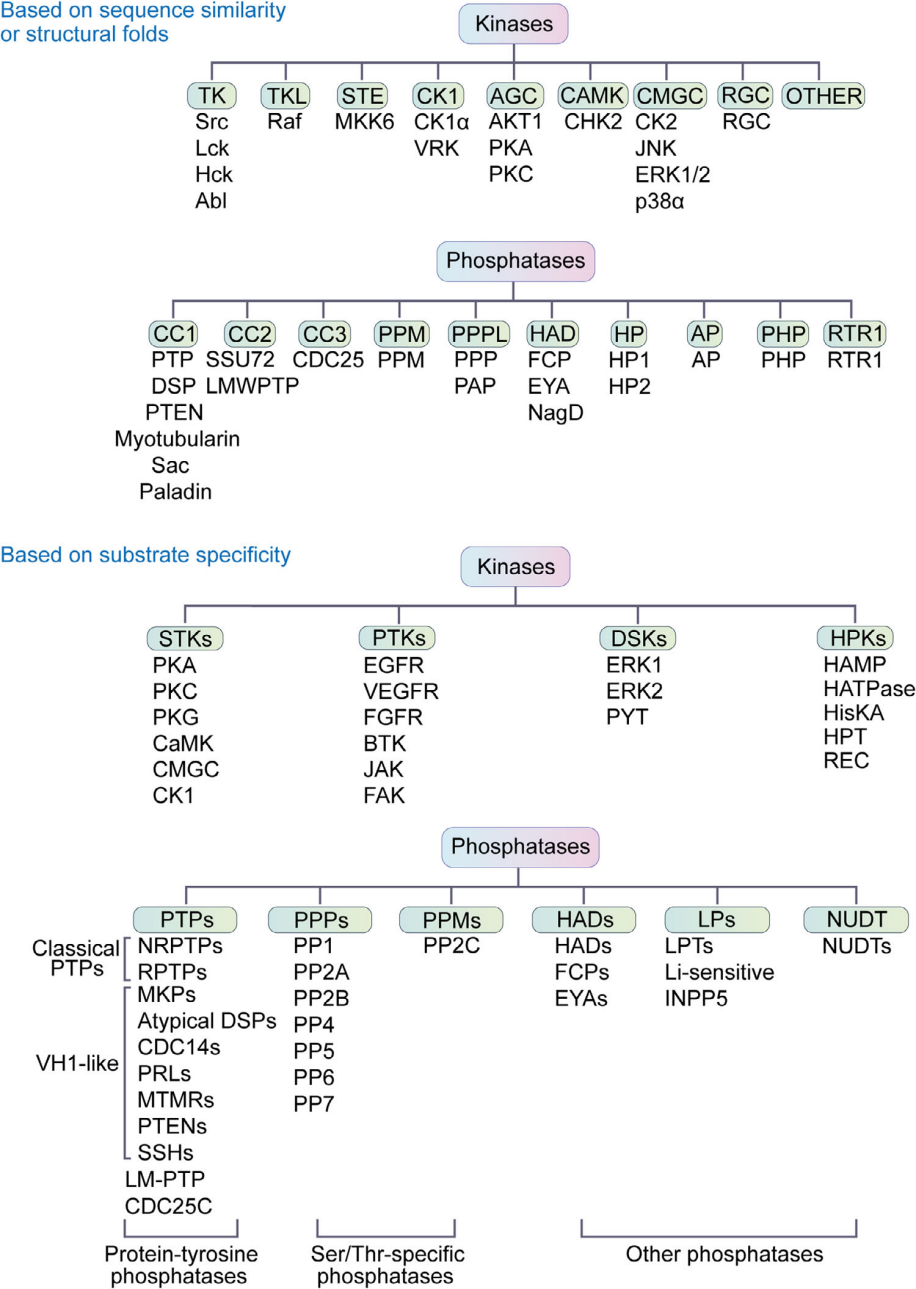
The kinases and phosphatases can be classified into different groups based on their phosphorylation substrates or based on their sequence similarity.

Protein kinases are widely distributed in cells throughout the nucleus, cytosol, mitochondria, and microsomes. To date, 518 protein kinases have been identified and verified.^42^ The 518 protein kinases are mainly divided into the following three categories according to the type of amino acids on which protein phosphorylation occurs, including serine/threonine protein kinases (STKs),^46^ protein tyrosine kinases (PTKs),^47^ dual‐specificity kinases (DSKs),^42^ and histidine protein kinases (HPKs) (Figure 3).^48^ The STKs are enzymes that phosphorylate serine or threonine and are activated by different events such as DNA damage and chemical signals. STKs include protein kinase A (PKA), protein kinase C (PKC), PKG, calcium/calmodulin‐regulated kinase (CaMK), CMGC, CK1, and so on.^49^ According to whether PTK is a cell membrane receptor, PTKs can be divided into nonreceptor type and membrane receptor type.^50^ Receptor‐type tyrosine kinases include EGFR, VEGFR, and FGFR. Abnormal activation of these kinases is related to angiogenesis, tumor invasion, and metastasis.^51^, ^52^ Nonreceptor tyrosine protein kinases mainly contain BTK, JAK, and FAK, which are related to cell proliferation and migration.^53^ DSKs can phosphorylate STKs and PTKs.^42^ HPKs are a large class of enzymes involved in signal transduction by auto‐phosphorylating conserved histidine residues.^48^ In addition, based on sequence similarity in the kinase domain, protein kinases can be divided into the following categories: tyrosine kinase (TK) family, tyrosine kinase‐like (TKL) family, sterile 20 serine/threonine (STE) kinase family, casein kinase 1 (CK1) family, protein A, G, and C (AGC) kinase family, CAMK family, CMGC family (including cyclin‐dependent kinases (CDKs), mitogen‐activated protein kinases (MAP kinases), glycogen synthase kinases (GSK) and CDK‐like kinases, receptor guanylate cyclase family (RGC), and others (Figure 3).^54^

In contrast, many phosphatases are thought to be passive housekeeping enzymes and seem less important than protein kinases.^55^ According to the pH required for their proper functions, phosphatases can be divided into alkaline phosphatases and acid phosphatases.^56^ Protein phosphatases can also be classified into three main families based on their substrate specificity, including the phosphoprotein phosphatase (PPP) family, metallo‐dependent protein phosphatase (PPM) family and protein‐tyrosine phosphatase (PTP) family^57^, with three additional families: HADs, LPs, and NUDT (Figure 3).^58^ The PPP and PPM families are serine/threonine‐specific phosphatases that appear to have evolved independently of each other.^41^ The phosphatases PP1, PP2A and PP2B and the newly discovered subfamilies PP4, PP5, PP6 and PP7 belong to the PPP family.^42^, ^59^ PP2C belongs to the magnesium ion‐dependent PPM family.^60^ Notably, most PTPs belong to the same class but can be assigned to different subfamilies based on their selectivity for tyrosine or tyrosine/serine/threonine phosphorylation substrates.^61^ The first type is the classical PTPs, which are specific to tyrosine phosphorylation. The second type is dual‐specificity phosphatases, which can dephosphorylate both serine and threonine residues in addition to tyrosine residues.^62^ Of all the phosphatases, at least 100 belong to those that dephosphorylate tyrosine residues, such as the tyrosine‐specific phosphatase subfamily, Cdc25 family, myotubularin‐related phosphatase and low molecular weight tyrosine phosphatase.^42^ PTP can also dephosphorylate aspartate‐based phosphatases such as FCP/SCP (small CTD phosphatase) and TAD (haloacid dehalogenase) family enzymes and nonprotein targets such as carbohydrates, mRNA, and phosphoinositides.^42^ According to the structural folds, protein phosphatases can be classified into 10 types, including CC1, CC2, CC3, PPM, PPPL, HAD, AP, HP, PHP, and RTR1 (Figure 3).^63^

Protein phosphorylation is one of the most abundant PTMs in humans and is involved in the regulation of numerous physiological processes, such as protein activity,^64^ protein stability, protein conformation, protein–protein interaction (PPI), growth signal response, cell cycle, cellular stress response, neuronal function, and immune response.^65^ It also plays important roles in cellular activities such as cell proliferation,^66^ transcriptional regulation,^67^ DNA repair,^68^, ^69^ subcellular localization,^70^ and tumor development (Figure 2B).^71^ Mutation and abnormal expression of kinases lead to abnormal activation or dysregulation of downstream signaling pathways^72^ and have been found to be the causes of many human diseases,^73^ such as immune diseases,^74^ hyperuricemia,^75^ neurodegenerative diseases,^76^ and cancers.^77^, ^78^

### 2.1 Phosphorylation in development

Protein phosphorylation is critical in growth and development.^79^ It is essential for the precise regulation of cell proliferation, cell cycle arrest, and differentiation into various cell types during embryonic development.^80^ During early embryonic development, the metabolism of mammalian totipotent stem cells is tightly regulated by the kinases HK and PFK1. In addition, phosphorylation regulates the process of embryonic development by mediating chromosome condensation and spindle assembly.^81^ EGF promotes AKT1 phosphorylation through PI3K, which further stimulates the proliferation of stem cells and precursor mesenchymal cells while blocking their differentiation.^82^ Phosphorylation of the RNA‐binding protein MSY2 during oocyte‐to‐embryo transition drives maternal mRNA degradation and converts a highly differentiated oocyte to totipotent blastomeres.^83^ PKD stimulates the phosphorylation of MAPK for spindle organization and cofilin for actin assembly and plays an important role in meiotic maturation of porcine oocytes.^84^

Growth inhibitory signaling is regulated by the Raf/MEK/ERK pathway, which plays an important role in early development and neuronal differentiation.^85^ The persistent activation of ERK1/2 is a common feature of growth inhibitory signaling in the Raf/MEK/ERK pathway.^86^ The target of rapamycin (TOR) is a kinase that regulates cell growth and metabolism by stimulating cell growth through anabolism and inhibiting catabolism.^87^ In mammalian cells, cyclin E plays a role in the G1 and S phases of cell cycle. Cyclin E1 and cyclin E2 affect cell growth and development by activating the cyclin‐dependent kinase CDK2 and then phosphorylating a series of proteins involved in cell cycle progression, male meiosis, and stem cell maintenance.^88^ Deficiency of both cyclin E1 and cyclin E2 in mice is embryonic lethal.^89^

### 2.2 Phosphorylation in aging

Aging is a process characterized by declines in both organism and organ functions, which can result in various diseases.^90^ This process is characterized by cell cycle arrest,^91^ abnormal accumulation of senescent cells in tissues,^92^ and altered neurotransmission and response ability to external stimuli.^93^ In quiescent cells, most protein phosphorylation does not change significantly with age.^94^ However, some protein phosphorylation significantly changes with age and has crucial physiological functions. For example, αB‐crystallin is a lenticular protein, and its phosphorylation can be boosted by aging, stress, and diseases.^95^ αB‐crystallin phosphorylation is also increased in aged muscle tissues and eye lenses.^96^, ^97^ Modulation of αB‐crystallin phosphorylation is a potential strategy to address aging‐related complications.^98^

p53 is an important tumor suppressor,^99^ and its ability to suppress tumors is related to the function of p53 in regulating the transcription of genes associated with cell cycle arrest and senescence.^100^ Phosphorylation of the p53 DNA‐binding domain can reduce its activity and prevent senescence.^98^ p53‐triggered senescence is also mediated by phosphorylation of other proteins, such as MDM2 at Ser183, which can activate p53‐mediated senescence and delay tumor progression.^100^

The brain is one of the most functionally affected organs during aging, and dysregulation of protein phosphorylation is common during brain aging.^101^ Protein phosphorylation signals in the brain are rich and diverse and mediated by kinases such as PKA, PKC, and CAM during aging.^102^, ^103^ The phosphorylation levels of B50/GAP‐43 protein, which plays a role in long‐term memory, are significantly reduced in the hippocampus of aging rats. An imbalance in protein phosphorylation, including Tau phosphorylation, acts as a key factor causing brain aging.^101^ Specifically, the accumulation of Tau phosphorylation at Ser396/404 in mitochondria is associated with cognitive dysfunction.^104^

Sarcopenia is characterized by the loss of skeletal muscle mass and strength with age.^105^, ^106^ The elderly population may experience basal hyperphosphorylation of mTORC1, which could potentially contribute to insulin resistance and the age‐related anabolic resistance of skeletal muscle protein metabolism in response to nutrition and exercise.^107^ The decreased phosphorylation of myosin regulatory light chain (RLC), a critical protein involved in the modulation of muscle contractility, at Ser14/15 with age is the cause of sarcopenia‐associated muscle dysfunction (Table 1).^108^

**TABLE 1:** Representative phosphorylation events in health and diseases.

Diseases and biological processes		Protein substrates	Effects
Aging		αB‐crystallin	αB‐crystallin phosphorylation increases in muscle tissues and eye lens with age.^96^, ^97^
		p53	Phosphorylation of p53 DNA‐binding domain reduces p53 activity and prevents senescence.^98^
			MDM2 phosphorylation at Ser183 activates p53‐mediated senescence and delays tumor progression.^100^
		B50/GAP‐43 protein	Phosphorylation B50/GAP‐43 is critical for long‐term memory and reduced in the hippocampus of aging rats.^193^
		Tau	Accumulation of Tau phosphorylation at Ser396/404 in mitochondria contributes to cognitive dysfunction during aging.^104^
		mTORC1	Basal mTORC1 hyperphosphorylation in the elderly may contribute to insulin resistance and the age‐related anabolic resistance of skeletal muscle protein metabolism to nutrition and exercise.^107^
		RLC	Decreased phosphorylation of RLC at Ser14/15 with age causes sarcopenia‐associated muscle dysfunction.^108^
Development		MeCP2	S421 phosphorylation controls the ability of MeCP2 to regulate dendritic patterning, spine morphogenesis, and the activity‐dependent induction.^194^
		AKT1	EGF promotes AKT1 phosphorylation, which further stimulates the proliferation of stem cells and precursor mesenchymal cells while blocks their differentiation.^195^
		MSY2	Phosphorylation of the MSY2 drives maternal mRNA degradation and converts a highly differentiated oocyte to totipotent blastomeres.^196^
Immune regulation	Infection	STAT1	Serine phosphorylation of STAT1 is required for the body's resistance to viral infection.^115^
		STAT6	STAT6 regulates the innate immunity by transducing signals from extracellular cytokines through phosphorylation by TBK1.^119^
		STAT2	STAT2 phosphorylation at S734 inhibits IFN‐α‐induced antiviral responses.^120^
		TRAF4	TRAF4 phosphorylation downregulates innate immune signaling.^197^
		STING	Cyclic dinucleotides trigger ULK1 (ATG1) phosphorylation of STING to prevent sustained innate immune signaling.^198^
		NF‐κB	NF‐κB activation regulated by phosphorylation controls the expression of a series of inflammatory cytokine genes and triggers the antiviral innate immune response.^199^
		MAVS	Phosphorylation of MAVS and STING by IKK/TBK1 induces type‐I IFNs and other antiviral molecules.^117^
		MITA	MITA phosphorylation by TBK1 during antiviral immunity activates IRF3.^200^
		YAP	Viruses activate the kinase IKKε to further phosphorylates YAP at Ser403 and trigger YAP degradation to antagonize innate antiviral immunity.^121^
	Tumor immunology	RAB7	RAB7 phosphorylation by TBK1/IKKε regulates innate immune signaling in triple‐negative breast cancer.^201^
		PDHE1α	Phosphorylated PDHE1α at S327 by ERK2 in cytoplasm can induce its transfer to mitochondria and improve NF‐κB signal in the cytoplasm, which increases resistance to cytotoxic lymphocytes and promotes tumor immune escape.^125^
		IκBα	In GBM, HK2 binds to IκBα and phosphorylates it at Thr291, which increases PD‐L1 expression and promotes tumor immune escape.^126^
		p73	CDK4/6 of tumor cells phosphorylate p53 family member p73 to prevent DR5 activation and promote antitumor immunity.^202^
		NEDD4	In urothelial carcinoma, activated FGFR3 phosphorylates NEDD4 and further regulates Lys48‐linked ubiquitination of PD‐L1 to activate CD8^+^ T cell infiltration and antitumor activity.^203^
		METTL3	TBK1 phosphorylates m6A methyltransferase METTL3 to enhance the interaction between METTL3 and the translation complex, which promotes antitumor immune response.^204^
Metabolic disorders	DM	GLP1	Phosphorylation at Arg91 may inhibit processing of glucagon precursor to GLP1 to affect the blood glucose levels.^132^
		PPARγ	Phosphorylation of PPARγ at S273 induces insulin resistance by upregulating Gdf3 expression and inhibiting BMP signaling pathway.^133^
		Afadin	Phosphorylation of Afadin at S1795 promotes insulin resistance in the early stages of diet‐induced obesity.^134^
	Obesity	PPARγ	Blocking PPARγ phosphorylation at Thr166 prevents obesity‐related metabolic dysfunction.^138^
Cancers	Multiple cancers	HK1	c‐Src phosphorylates HK1 at Tyr732 to promote the glycolysis rate of tumor cells and their proliferation, invasion, and metastasis abilities.^71^
	Multiple cancers	IκBα	Aerobic glycolysis promotes tumor immune escape through phosphorylation of IκBα at T291 mediated by HK2.^126^
	Breast cancer	HK2	Phosphorylation of HK2 at Thr473 by PIM2 enhances HK2 stability and activity and promotes glycolysis, tumor growth, and drug resistance to paclitaxel.^165^
	Glioma	PFKP	Phosphorylation of PFKP by AKT at Ser386 inhibits PFKP degradation and promotes aerobic glycolysis of glioma cells and tumor growth.^167^
	Melanoma	PFKFB2	RSK phosphorylates PFKFB2 to increase PFKFB2 activity and the glycolysis pathway, which accelerates the growth of BRAF‐mutated melanoma.^168^
	Multiple cancers	PKM2	Phosphorylation of PKM2 at Tyr105 mediates the transformation of tumor cell metabolic mode to aerobic glycolysis.^170^
	Multiple cancers	PDHA	Hyperphosphorylation of PDHA at Ser295 and Ser314 redirects tumor metabolism to TCA cycle. This protects spread cancer cells from metabolic and oxidative stress‐induced cell death and promotes tumor metastasis.^171^
	Gastric cancer	ULK1	DAPK3 directly phosphorylates Ser556 of ULK1 to increase ULK1 activity and promote the formation of ULK1 complex, leading to inhibition of the proliferation of gastric cancer cells.^173^
	GBM	ACSS2	Phosphorylation at Ser267 of ACSS2 by CDK5 inhibits the degradation of ACSS2 and promote the growth of GBM tumor cells.^174^
	Colon cancer	Drp	ERK phosphorylates Drp1 at Ser616 to activate it. Activated Drp1 facilitates the oxidation of fatty acids to promote the proliferation of colon cancer cells.^176^
	Breast cancer	RNF12	AKT promotes TGF‐β‐driven breast cancer metastasis by mediating RNF12 phosphorylation and enhancing RNF12 stability.^177^
	Bladder cancer	AKT	KNSTRN phosphorylates AKT at Thr308 and Ser473 to activate AKT and promotes bladder cancer metastasis.^178^
	Breast cancer	PKM2	Phosphorylation of PKM2 at Ser37 is a prominent feature of invasive breast cancer.^180^
	PC	PD‐L1	NEK2 phosphorylates PD‐L1 at Thr194/Thr210 to maintain its stability, leading to less effectiveness of PD‐L1‐targeted therapy in PC.^205^
Neurodegenerative diseases	PD	Parkin	Dyrk1A phosphorylates Parkin at Ser131 to inhibit its E3 Ub ligase activity, which may be involved in the pathogenesis of PD.^206^
		XBP1s	PINK1 can control XBP1s transcriptional activity by phosphorylating XBP1s at Ser61 and The48, which consequently enhances PINK1's own transcription.^186^
	AD	Tau	Transient Tau hyperphosphorylation has a protective effect on neurons. While persistent accumulation of phosphorylated Tau causes neurodegeneration.^189^ Hyperphosphorylated Tau depolymerizes normal microtubule‐associated proteins after forming neuronfibrillary tangle, disrupts cellular dynamic structures, blocks intracellular material exchange and cell signaling, inhibits Ub–proteasome activity.^190^, ^191^

Abbreviations: AD, Alzheimer's disease; DM, diabetes mellitus; GBM, glioblastoma; PC, pancreatic cancer; PD, Parkinson's disease; RLC, regulatory light chain.

### 2.3 Phosphorylation in immune regulation

Phosphorylation, a common PTM, plays a crucial role in regulating innate and acquired immunity, a process that is coregulated by kinases and phosphatases.^109^, ^110^ Phosphorylation and other PTMs work together to regulate the signaling networks of the immune system. For example, the MAP4K family of kinases play an important role in immune cell signaling, immune response, and inflammation; PKC is involved in regulating the important signaling pathways of innate and adaptive immunity, and plays an intermediary role in the signaling process of immune cells through immune synapses; PKA is involved in multiple processes that regulate immune activation and immune control, not only regulating lymphocyte activation, but also modulating antigen receptor‐induced signaling by altering protein interactions and altering enzyme activity of substrate proteins.^111^, ^112^, ^113^

Normally, phosphorylation and dephosphorylation maintain a dynamic balance in maintaining the immune homeostasis of organisms. On the one hand, protein phosphorylation is widely involved in immune regulation, for example, the receptors on immune cells trigger phosphorylation signals through the recruitment of TKs, resulting in the activation of immune cells.^114^ Shuai et al.^115^ showed that serine phosphorylation of STAT1, an important signal converter in IFN signaling, is required for the body's resistance to viral infection. On the other hand, dephosphorylation of proteins is also widely involved in immune responses and this process is mediated by phosphatases.^110^

Innate immunity is the first line of defense against pathogen invasion. Phosphorylation plays an important role in innate immunity. It has been shown that the transcription factor (TF) interferon regulatory factor 3 (IRF‐3) regulates gene expression in innate immune responses, and IRF‐3 activation is mediated by phosphorylation of kinase IKK/TBK1.^116^ The toll‐like receptors (TLRs) are principal sensors capable of sensing multiple microbial stimuli and inducing innate immune responses through a cascade of phosphorylation signals. TLR signaling reaches its peak during the activation of nuclear factor‐kappaB (NF‐κB), which is mediated by phosphorylation and controls the expression of a series of inflammatory cytokine genes and further triggers the innate immune response against viruses. During viral infection, the adaptor proteins MAVS and STING are phosphorylated by the kinase IKK/TBK1 in response to stimulation, inducing type I interferons (IFNs) and other antiviral molecules.^117^ Chen et al. found that ionizing radiation leads to phosphorylation of phosphoribosyl pyrophosphate synthetase 1/2 at T228, triggering innate immune response in the body.^118^

The antiviral immune response also requires phosphorylation to mediate. STAT6 is essential for antiviral innate immunity. After viral infection, STAT6 is aggregated in the endoplasmic reticulum and phosphorylated by TBK1, which then dimerizes into the nucleus and regulates the expression of antiviral immunity genes.^119^ And phosphorylation of STAT2 at S‐734 inhibits IFN‐α‐induced antiviral response.^120^ The virus also activates the kinase IKKε, which phosphorylates YAP at Ser403, triggering degradation of YAP in lysosomes and antagonizing innate antiviral immunity.^121^ In addition, kinase complex mTORC2, which is involved in phosphorylation of AKT and GSK3β kinase, can maintain reactive oxygen species balance in mitochondria and maintain the lifespan of virus‐specific memory CD4^+^ T cells in vivo, playing an important role in antiviral immunity.^122^

Phosphorylation plays a key role in signal transduction during tumor immunity, mediating immune escape in a variety of tumors. PD‐1 is crucial for inhibiting the activation of T cells in vitro and in vivo, and its immunosuppression process also requires phosphorylation mediated by the specific mechanism as follows: PD‐1 binds to its ligand PD‐L1, then aggregates with T cell receptors (TCRs) and binds briefly to phosphatase SHP2 to initiate dephosphorylation of TCR, resulting in inhibition of T cell activation.^123^ Inhibition of CDK4/6 in vivo has been shown to inhibit cyclin D‐CdK4‐mediated Spoz protein phosphorylation, thereby increasing PD‐L1 protein levels, and this can increase the number of tumor infiltrating lymphocytes and enhance tumor immunity.^124^ Yang et al. found that phosphorylated PDHE1α (pyruvate dehydrogenase complex E1 subunit α) at S327 by ERK2 in cytoplasm can induce its transfer to mitochondria and improve NF‐κB signal in the cytoplasm, which increases resistance to cytotoxic lymphocytes and promotes tumor immune escape.^125^ In human glioblastoma cells, a high glucose environment promotes mitochondrial separation of hexokinase 2 (HK2), which binds to the T291 site of IκBα and phosphorylates it, subsequently mediating upregulation of PD‐L1 and promoting tumor immune escape.^126^

### 2.4 Phosphorylation in metabolic disorders

Abnormal phosphorylation may lead to the blockage of cell signaling and in turn result in metabolic disorders in the human body.^42^ Diabetes mellitus is a metabolic syndrome characterized by long‐term hyperglycemia, 90% of which is T2DM. Insulin resistance is a fundamental mechanism leading to T2DM.^127^ Glucose homeostasis is maintained by insulin in insulin‐responsive tissues, while phosphorylation is a critical mechanism for regulating insulin secretion and insulin signaling processes.^33^, ^128^, ^129^ Over 1000 phosphorylation events are dysregulated in T2DM.^130^ The effect of phosphorylation on diabetes occurs mainly through the cascade of kinases and phosphatases that regulate insulin signaling.^129^ Several key molecules in the insulin pathway, such as IR, IRS1, IRS2, PDK, and mTORC1, are phosphorylated upon insulin stimulation.^131^ In mammals, GLP1 acts as an incretin to promote the release of insulin from pancreatic B cells. It is speculated that phosphorylation at Arg91 may inhibit processing of the glucagon precursor to GLP1 to affect blood glucose levels.^132^ In addition, phosphorylation of obesity‐associated PPARγ at S273 induces insulin resistance by upregulating Gdf3 expression and inhibiting the BMP signaling pathway1.^133^ Phosphorylation of Afadin at S1795 also promotes insulin resistance in the early stages of diet‐induced obesity.^134^

Obesity, a common metabolic disorder, results from the accumulation of adipose tissue caused by energy imbalances.^135^ Phosphorylation plays a role in the pathogenesis of obesity by regulating adipogenesis and metabolism. For example, S6K1 participates in many key metabolic pathways, including lipid synthesis in the body, by mediating the phosphorylation of H2BS36 in obese patients. S6K1 is a potential therapeutic target for obesity.^136^ Mammalian white adipose tissue (WAT) is critical for whole‐body homeostasis. Smyd2 is abundant in WAT and regulates STAT2 phosphorylation to regulate adipocyte differentiation.^137^ PPARγ is indispensable in the process of adipocyte differentiation, and the phosphorylation level of PPARγ at Thr166 is positively correlated with obesity status. Specifically, blocking PPARγ phosphorylation at Thr166 prevents obesity‐related metabolic dysfunction (Table 1).^138^

### 2.5 Phosphorylation in cancers

Abnormal kinase activity and expression are implicated in various types of cancers. In recent years, with the increasing development of mass spectrometry (MS) technology, the Clinical Proteomic Tumor Analysis Consortium and many other teams have conducted phosphoproteomics investigations in various cancers, such as lung cancer,^139^, ^140^, ^141^, ^142^ colorectal cancer (CRC),^143^, ^144^, ^145^, ^146^ liver cancer,^147^ breast cancer,^148^ prostate cancer,^149^ gastric cancer,^150^, ^151^ head and neck cancer,^152^ esophageal cancer,^153^ pancreatic cancer (PC),^154^, ^155^ kidney cancer,^156^, ^157^ melanoma,^158^ skin cancer,^159^ leukemia,^160^ pancreatic ductal adenocarcinoma (PDAC),^161^ pituitary neuroendocrine tumors,^162^ cholangiocarcinoma,^163^ and urothelial carcinoma of the bladder.^164^

Protein phosphorylation mediates metabolic reprogramming of tumors. Studies have found that c‐Src phosphorylates HK1 at Tyr732, which promotes the glycolysis rate of tumor cells and their proliferation, invasion, and metastasis abilities.^71^ Aerobic glycolysis can promote tumor immune escape through IκBα^T291^ phosphorylation mediated by HK2.^126^ Moreover, HK2 can be phosphorylated at Thr473 by the kinase PIM2, which increases its stability and enzymatic activity and promotes glycolysis and breast tumor growth, enhancing its drug resistance to paclitaxel.^165^ AKT2 may also be an upstream kinase leading to HK2 Thr473 phosphorylation in CRC.^166^ PFK also plays an important role in the regulation of tumor metabolism. The homologous isoform PFKP of PFK1 can be phosphorylated by AKT at Ser386, which inhibits the degradation of PFKP and promotes aerobic glycolysis in glioma cells and tumor growth.^167^ RSK directly phosphorylates PFKFB2 to increase PFKFB2 activity and glycolysis, which accelerates the growth of BRAF‐mutated melanoma.^168^ PFKFB3 in the cytoplasm is phosphorylated and activated by AMPK. Targeted inhibition of PFKFB3 improves the sensitivity of chemotherapy drugs such as cisplatin.^169^ Phosphorylation at Tyr105 of PKM2 is significantly increased in various tumors to mediate the transformation of the tumor cell metabolic mode to aerobic glycolysis.^170^ Hyperphosphorylation of Ser295 and Ser314 of PDHA redirects tumor metabolism to the tricarboxylic acid (TCA) cycle by increasing PDH activity. This protects cancer cells from metabolic and oxidative stress‐induced cell death and promotes tumor metastasis.^171^

Protein phosphorylation extensively regulates cancer cell proliferation, metastasis, and invasion.^172^ For example, DAPK3 directly phosphorylates Ser556 of ULK1, which increases the activity of ULK1 and promotes the formation of the ULK1 complex, leading to inhibition of the proliferation of gastric cancer cells. The downregulation of DAPK3 in gastric cancer patients is related to poor prognosis.^173^ Phosphorylation at Ser267 of ACSS2 by CDK5 kinase inhibits the degradation of ACSS2 and promotes the growth of GBM tumor cells.^174^ BZW1 enhances the phosphorylation of eIF2α to promote tumor progression. This process can be prevented by the PERK/eIF2α phosphorylation inhibitors GSK2606414 and ISRIB.^175^ The kinase ERK catalyzes Drp1 phosphorylation at Ser616 to activate Drp1. Activated Drp1 changes the metabolic pathway, facilitates the oxidation of fatty acids, and promotes the proliferation of colon cancer cells.^176^

Dysregulated phosphorylation can promote tumor metastasis. For example, AKT promotes TGF‐β‐driven breast cancer metastasis by mediating RNF12 phosphorylation and enhancing RNF12 stability.^177^ KNSTRN, a component of the mitotic spindle, phosphorylates AKT at Thr308 and Ser473 to activate AKT and promote bladder cancer metastasis.^178^ TKT, a key metabolic enzyme in the pentose phosphate pathway (PPP), interacts with GRP78 to promote glycolysis by increasing AKT phosphorylation, which promotes CRC metastasis.^179^ PKM2 phosphorylation at Ser37 is a prominent feature of invasive breast cancer. The use of the pyruvate kinase activator TEPP‐46 or the potent CDK inhibitor dinaciclib to bind to phosphorylation sites can reduce its nuclear localization and inhibit cancer cell migration and invasion (Table 1).^180^

### 2.6 Phosphorylation in neurodegenerative diseases

*Parkin* is a tumor suppressor gene, and its overexpression can inhibit the growth of tumor cells. *Parkin* mutations exist in a variety of malignant tumors, such as colon cancer,^181^ PC,^182^ and cervical cancer.^183^ However, *Parkin* is also a causative gene related to Parkinson's disease (PD). It has a neuroprotective effect, and mutations in *Parkin* lead to the loss of dopaminergic neurons in the substantia nigra.^184^ Parkin is almost inactive in vitro, and its activation is regulated by PINK1‐mediated phosphorylation.^185^ After phosphorylation, the protein conformation, solubility, and affinity with the substrate of Parkin are changed. Parkin amplifies the PINK1‐induced signaling pathway through positive feedback, which enhances mitophagy and selectively degrades defective mitochondria to maintain the stability of the intracellular environment. Abnormalities in this pathway may cause PD.^184^ In addition, PINK1 controls XBP1s transcriptional activity by phosphorylating XBP1s at Ser61 and Thr48, which consequently enhances PINK1 transcription, and triggers a promitophagic phenotype.^186^ Notably, functional deficiency of Parkin leads to ineffective ubiquitination and a large accumulation of cyclins. These cyclins are responsible for initiating the cell cycle in both neurons and mitotically active cells. However, due to the lack of mitogenic capacity in neurons, their inability to undergo cell division ultimately leads to apoptosis.^187^

Tau hyperphosphorylation has an intrinsic link with neurodevelopment and degeneration, and the phosphorylation level of Tau in the AD brain is three to four times higher than that of normal peers.^188^ Transient Tau hyperphosphorylation is protective on neurons. However, persistent accumulation of hyperphosphorylated Tau may cause neurodegeneration.^189^ Hyperphosphorylated Tau depolymerizes normal microtubule‐associated proteins after forming neuronfibrillary tangles, disrupts cellular dynamic structures, blocks intracellular material exchange and cell signaling, inhibits ubiquitin (Ub)–proteasome activity, and finally leads to neurodegenerative diseases.^190^, ^191^ In a cohort study of 593 elderly people with an average age of 64 years, it was found that compared with cognitively normal controls, the plasma concentrations of P‐tau217 and P‐tau181 are increased in clinical AD patients, suggesting that P‐tau217 and P‐tau181 may be useful biomarkers for AD diagnosis (Table 1).^192^

### 2.7 Phosphorylation‐associated targeted therapies

Compared with traditional cytotoxic anticancer drugs, targeted anticancer drugs have the advantages of high efficiency, low toxicity, and strong specificity.^207^ Given the important roles of protein kinases in tumor growth and metastasis, if the kinase signaling pathway is effectively blocked, the malignant progression of tumors may be prevented.^208^ To date, the United States Food and Drug Administration (US FDA) has approved 68 small molecule kinase inhibitors.^209^ These kinase inhibitors can be roughly divided into four classes according to the way they bind to protein kinases. Type I kinase inhibitors are by far the most US FDA‐approved drugs, such as bosutinib, dasatinib, and crizotinib.^210^ Dasatinib acts on multiple targets, such as BCR‐Abl and the SRC kinase family, and is mainly used for the treatment of leukemia.^211^ Crizotinib has been confirmed in tumor patients with abnormal ALK, ROS kinase, and HGFR/c‐MET activities.^212^ Type II kinase inhibitors, including the BCR‐Abl inhibitors imatinib and nilotinib, are mainly used for the treatment of chronic myeloid leukemia (CML).^213^, ^214^ Another representative drug, sorafenib,^215^ is a typical multitarget drug targeting TKs such as VEGFR2 and PDGFR‐β, as well as the serine/threonine kinase Raf‐1,^216^ and can be used for the treatment of hepatocellular carcinoma (HCC) and renal cell carcinoma (RCC).^217^, ^218^ Allosteric kinase inhibitors are another type of kinase inhibitor.^215^ Trametinib and cobimetinib are allosteric kinase inhibitors targeting MEK1/2, both of which can be used for the treatment of non‐small cell lung cancer (NSCLC).^219^ Allosteric kinase inhibitors do not bind to the ATP binding site, so they act together with ATP‐competitive inhibitors, which makes allosteric inhibitors useful for overcoming the low selectivity, off‐target effects and resistance of small molecule inhibitors.^220^ The fourth type of kinase inhibitors are covalent inhibitors, such as afatinib, neratinib, ibrutinib and acalabrutinib.^215^, ^221^ Afatinib acts on EGFR and is mainly used to NSCLC.^222^ Neratinib inhibits HER2 and is used for the treatment of HER2‐positive breast cancer.^223^ The BTK inhibitors ibrutinib and acalabrutinib are mainly used for the treatment of chronic lymphocytic leukemia (CLL) and mantle cell lymphoma (MCL).^224^, ^225^ Notably, acalabrutinib significantly prolonged the progression‐free survival of patients with CLL (Table 2).^221^

**TABLE 2:** Representative approved kinase inhibitors and their clinical uses.

Classifications	Targets	Drugs	Clinical uses
TKIs	ALK	Alectinib, brigatinib, ceritinib, crizotinib, lorlatinib	ALK‐positive NSCLC^238^
	BCR‐Abl	Bosutinib, dasatinib, nilotinib, ponatinib	CML^239^
		Imatinib	Ph^+^ CML/ALL, GIST, aggressive systemic mastocytosis, chronic eosinophilic leukemias, dermatofibrosarcoma protuberans, hypereosinophilic syndrome, myelodysplastic, and myeloproliferative disease^207^, ^209^, ^210^
	BTK	Acalabrutinib	MCL, CLL, SLL^240^, ^241^
		Ibrutinib	CLL, MCL, marginal zone lymphomas, graft‐versus‐host disease^209^
	c‐MET	Cabozantinib	Metastatic medullary thyroid cancer^242^
		Crizotinib	Metastatic ALK‐, c‐MET‐, or ROS‐1‐positive NSCLC^243^
	c‐KIT	Axitinib	RCC^244^
		Cabozantinib	Metastatic medullary thyroid cancer^245^
		Erlotinib	NSCLC^246^
		Nilotinib	Ph^+^ CML^247^
		Pazopanib	Advanced RCC, advanced soft tissue sarcoma^248^, ^249^
	CSF1R	Pexidartinib	Tenosynovial giant cell tumors^250^
	EGFR	Erlotinib	NSCLC, PC^251^, ^252^
		Afatinib, dacomitinib, gefitinib, osimertinib	NSCLC^253^
		Lapatinib, neratinib	HER2‐positive breast cancer^254^
	FGFR	Erdafitinib	Urothelial bladder cancers^255^
		Nintedanib	IPF^256^
	FLT3	Gelteritinib	AML^257^
		Midostaurin	AML, mastocytosis, mast cell leukemias^258^, ^259^
	JAKs	Fedratinib, ruxolitinib	Myelofibrosis^260^
		Baricitinib, tofacitinib	RA^261^
	PDGFR	Axitinib	RCC^244^
		Erlotinib	NSCLC^207^
		Nilotinib	Ph^+^ CML^210^
		Pazopanib	Advanced RCC, advanced soft tissue sarcoma^210^, ^262^
		Sorafenib	RCC, HCC^210^, ^263^, ^264^
		Sunitinib	RCC, GIST^265^, ^266^
	RET	Alectinib	NSCLC^267^
		Cabozantinib	Medullary thyroid cancers, RCC, HCC^268^
		Lenvatinib	Differentiated thyroid cancers^269^
	ROS1	Crizotinib, entrectinib	ROS1‐postive NSCLC^270^
	SRC	Bosutinib	Ph^+^ CML^271^
		Dasatinib	Ph^+^ CML/ALL^210^
	Syk	Fostamatinib, R406	Chronic immune thrombocytopenia^272^
	TRKA/B/C	Entrectinib, larotrectinib	Solid tumors with NTRK fusion proteins^273^, ^274^
	Tyk	Ruxolitinib	Myelofibrosis, polycythemia vera^209^
	VEGFR	Axitinib	RCC^275^
		Cabozantinib	Medullary thyroid cancers, RCC, HCC^276^, ^277^, ^278^
		Lenvatinib	Differentiated thyroid cancer^279^
		Pazopanib	RCC, soft tissue sarcomas^248^, ^280^
		Regorafenib	CRC^281^
		Sorafenib	RCC, HCC, differentiated thyroid cancer^282^, ^283^, ^284^
		Sunitinib	GIST, RCC, pancreatic neuroendocrine tumors^285^, ^286^, ^287^
		Vandetanib	Medullary thyroid cancers^288^
STK inhibitors	BRAF	Dabrafenib	*BRAF^V600E/K^* melanomas, *BRAF^V600E^* NSCLC, *BRAF^V600E^* anaplastic thyroid cancers^289^, ^290^, ^291^
		Encorafenib, vemurafenib	*BRAF^V600E/K^* melanoma^292^
	CDKs	Abemaciclib, palbociclib, ribociclib	Breast cancer^293^
	FKBP12/mTOR	Everolimus	HER2‐negative breast cancers, pancreatic neuroendocrine tumors, RCC, angiomyolipomas, subependymal giant cell astrocytomas^294^, ^295^, ^296^, ^297^, ^298^
		Sirolimus	Kidney transplants, lymphangioleiomyomatosis (LAM)^299^, ^300^
		Temsirolimus	RCC^301^
	ROCK1/2	Netarsudil	Glaucoma^302^
Double specific protein kinase inhibitors	MEK1/2	Binimetinib, cobimetinib	*BRAF^V600E/K^* melanoma^303^
		Trametinib	*BRAF^V600E/K^* melanomas/*BRAF^V600E^* NSCLC^304^

Abbreviations: ALL, acute lymphocytic leukemia; AML, acute myeloid leukemia; CLL, chronic lymphocytic leukemia; CML, chronic myeloid leukemia; CRC, colorectal cancer; GIST, gastrointestinal stromal tumors; HCC, hepatocellular carcinoma; IPF, idiopathic pulmonary fibrosis; MCL, mantle cell lymphoma; NSCLC, non‐small cell lung cancer; PC, pancreatic cancer; RA, rheumatoid arthritis; RCC, renal cell carcinoma; SLL, small lymphocytic lymphoma.

Tyrosine kinase inhibitors (TKIs) are currently the most widely studied. The TK EGFR is mutated or overexpressed in a variety of tumors. Abnormal expression of EGFR is closely related to the occurrence of cancer. Thus, the development of drugs targeting EGFR is a research hotspot.^226^ The current small‐molecule inhibitors designed to target EGFR have been developed into the fourth generation.^227^ The first three generations of inhibitors are widely used in the clinic and have gradually become the first choice for NSCLC treatment, mainly by inhibiting the phosphorylation of the intracellular TK domain.^228^ First‐generation EGFR‐TKIs, including gefitinib and erlotinib, are reversible inhibitors.^229^, ^230^ Second‐generation EGFR‐TKIs, including dacomitinib and afatinib, are irreversible.^230^ Third‐generation EGFR‐TKIs mainly target T790M mutant EGFR and are irreversible as well. The representative drug is osimertinib.^231^ Although TKIs, represented by third‐generation EGFR‐TKIs, have achieved remarkable success in the field of cancer treatment, clinical results show that there are still inevitable toxic side effects in the gastrointestinal tract, skin and other organs.^232^ In addition, TKIs have also been used to treat T1DM and T2DM.^233^ For example, c‐Abl^234^ and VEGFR2^235^ inhibitors have been shown to enhance β cell survival and insulin secretion, while PDGFR^236^ and EGFR^237^ inhibitors have been demonstrated to improve insulin sensitivity.

## 3 ACETYLATION

**FIGURE 4:**
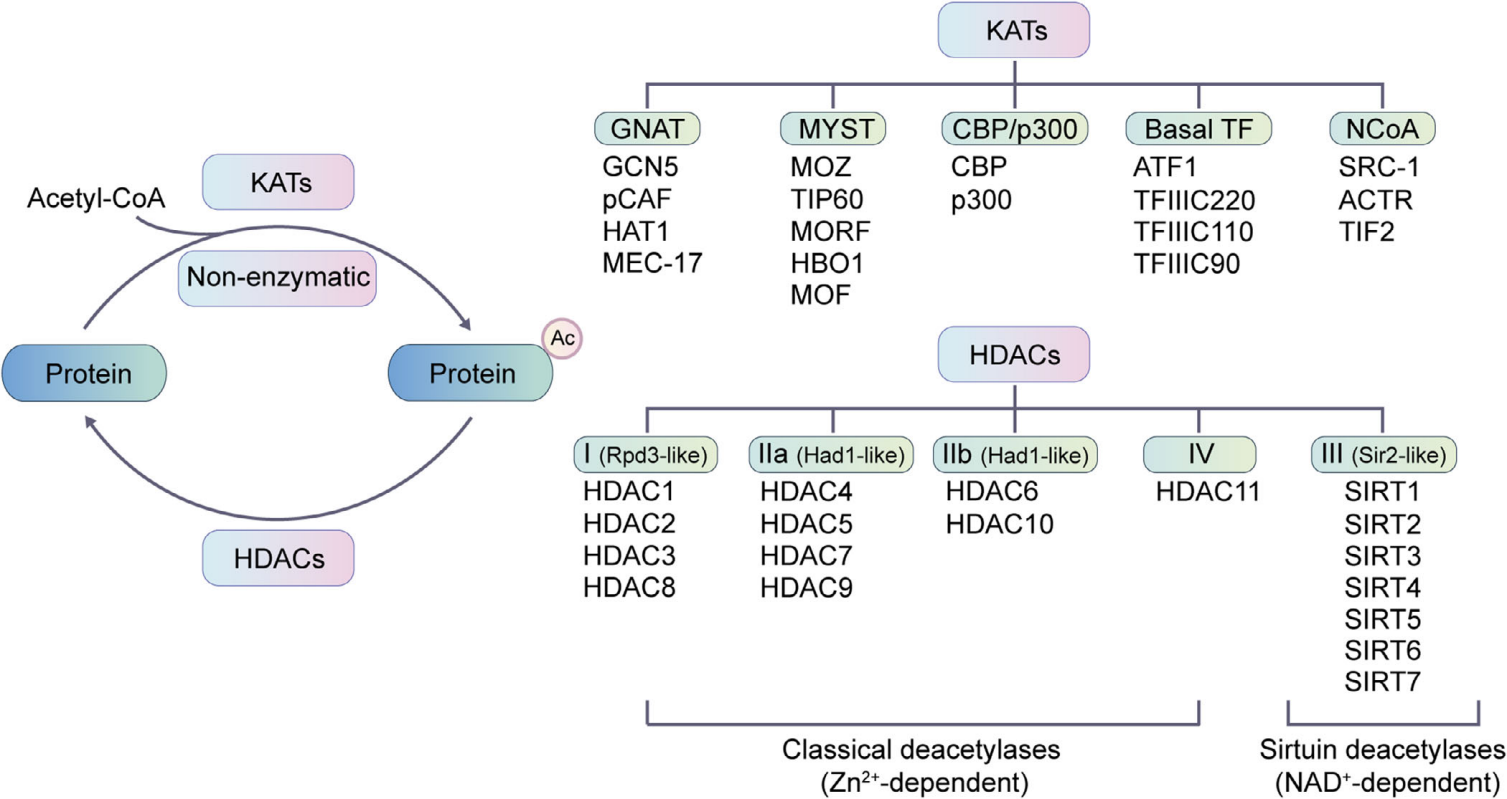
Representative scheme of reversible acetylation regulated by HATs and HDACs is shown. The classification of well‐known HATs and HDACs are organized. KATs are classified into three major families: GCN5, p300 and MYST. The remaining KATs belong to basal TF family and NCoA family. HDACs are divided into two categories: the classical Zn^2+^‐dependent HDACs and NAD^+^‐dependent sirtuin deacetylases. HDACs can be further grouped into class I, class Iia, class Iib, class III, and class IV.

Acetylation is a process in which acetyl group donors, such as acetyl‐CoA and acetyl phosphate, covalently bind to the protein N‐terminus and lysine side chains in an enzymatic or nonenzymatic manner, forming N‐terminal (Nα) and internal (Nε) acetylation (Figure 4).^305^, ^306^ The regulation of Nα‐acetylation remains unclear,^307^ while the process of Nε‐acetylation is dynamic and reversible.^308^ Nε‐acetylation changes with the physiological state of cells and the external environment. It serves as a regulatory switch for protein conformation and activity changes. However, when Nε‐acetylation becomes abnormal, it can lead to the development of diseases.^306^

The homeostasis of lysine acetylation is regulated by lysine acetyltransferase (HATs/KATs) and lysine deacetylase (HDACs/KDACs).^309^ Acetylase KATs are mainly divided into three families (Figure 4), including the GNAT superfamily (GCN5, pCAF, HAT1, and MEC‐17), the MYST family (MOZ, TIP60, MORF, HBO1, and MOF), and the CBP/p300 family. Apart from the above three main categories, there are also two other KAT families, including the basal TF family and the nuclear receptor coactivator (NCoA) family.^310^ The deacetylases are divided into two large families (Figure 4). The classical large family includes 11 members, HDAC1–11, which are similar in secondary structure to the yeast Hda1/Rpd3 protein, and all rely on Zn^2+^ to promote deacetylation. The deacetylases in the second major family are all NAD^+^‐dependent yeast Sir2 homologous proteins, including seven members SIRT1–7.^311^

**FIGURE 5:**
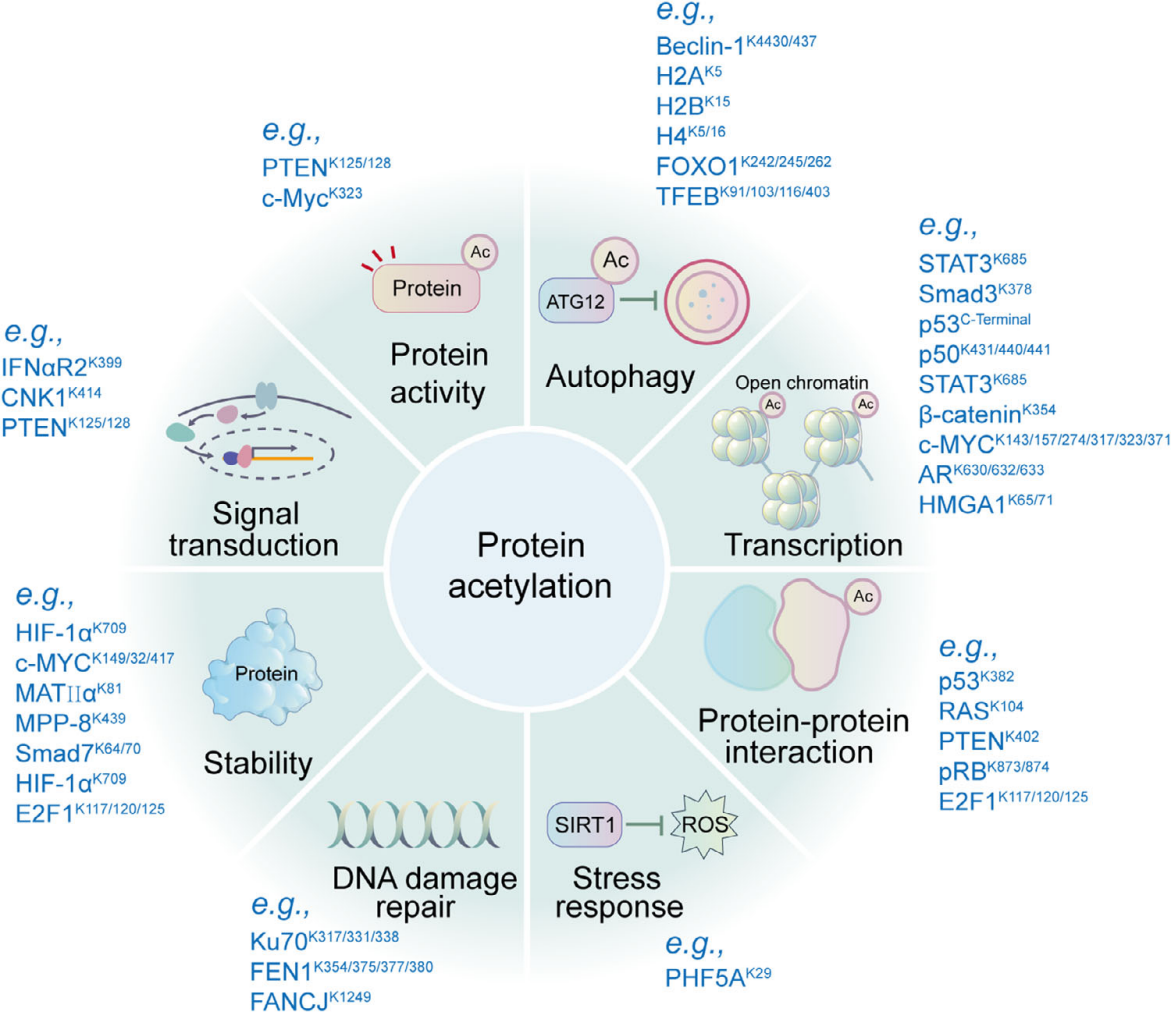
Representative functions of histone and nonhistone acetylation are shown. Protein acetylation is mainly involved in the regulation of gene transcription, metabolism, DNA damage repair, signal transduction, stress response, signal transduction, protein stability, protein activity, protein–protein interaction, and autophagy.

Acetylation is a widespread PTM involved in gene transcription, metabolism, DNA damage repair, signal transduction, PPIs, stress response, proteolysis, autophagy, and many other biological processes (Figure 5).^20^, ^312^, ^313^ In particular, histone acetylation is closely related to transcriptional activity, and hyperacetylated histones are specifically aggregated in active chromatin.^314^ Mechanically, negatively charged acetyl groups covalently added to specific lysine residues in histones can diminish the electrostatic affinity between histone proteins and DNA, thus disrupting the interaction of these histones with DNA and leading to chromatin relaxation that enables the activation of gene transcription.^314^ For example, SIRT2 catalyzes H4K16 deacetylation to maintain a condensed heterochromatin state and shut down gene transcription, whereas the histone acetyltransferase Sas2 counteracts this effect.^315^ In contrast, acetylation of H4K16 (rather than H4K5, H4K8, and H4K12) contributes to the folding of nucleosome arrays, which is essential for transcriptional regulation in vivo,^316^ suggesting that the position of acetylation in histone protein is much more important than the number of acetylation modifications. p300/CBP is a coactivator of various TFs, such as p53,^317^ HIF‐1α,^318^ and c‐Myc,^319^ which can remodel chromatin and transcription processes through the activity of acetyltransferase. In addition, acetylation also acts on almost all metabolic enzymes. By changing the PPI, localization, stability, and activity of metabolic enzymes, acetylation is extensively involved in metabolism regulation.^320^ In mouse hepatocyte mitochondria, more than 20% of mitochondrial proteins have been acetylated, including many growth factors and metabolic enzymes. In human liver tissue, 1300 lysine acetylation sites from 1047 proteins have been identified. Interestingly, almost all intermediate metabolic enzymes are acetylated.^321^ Acetylation is involved in cellular antioxidant processes. SOD2, IDH2, and G6PD are all regulated by acetylation. Deacetylation of SOD2^322^, ^323^ and IDH2^324^, ^325^ by SIRT3 and deacetylation of G6PD^326^ by SIRT2 can increase the catalytic capacity of SOD2, IDH2, and G6PD, as well as the level of NADPH, consequently reducing cellular oxidative damage.

The regulation of protein stability by acetylation is usually achieved by competing with ubiquitination. For example, FASN is a key enzyme of nascent adipogenesis, and HDAC3 can reduce the interaction of FASN with the E3 Ub ligase Trim21 by deacetylating FASN.^327^ K163, K174, K180, and other lysine sites of Tau can be acetylated by p300, which inhibits normal ubiquitination‐dependent protein degradation and microtubule assembly and promotes abnormal aggregation of Tau protein, leading to neurodegenerative diseases such as AD (Figure 5).^328^

Autophagy can be regulated by acetylation. Acetylation of histones and TFs regulates autophagy‐related gene expression and their activities. The rapid and precise regulation of autophagy contributes to the maintenance of cellular homeostasis.^329^ p300 appears to acetylate many ATG proteins that regulate autophagy at multiple steps. p300 depletion or p300 inhibitors induce autophagy, while p300 overexpression inhibits autophagy.^330^ The acetylation of the mTORC1 component raptor is increased through a p300‐dependent pathway, which activates mTORC1 and inhibits autophagy (Figure 5).^331^

Acetylation and deacetylation coordinated by acetyltransferase and deacetylase are in a dynamic balance to maintain normal physiological and biochemical processes of cells. However, once this balance is broken, it will lead to disordered regulation of gene expression and the occurrence of diseases.^332^ During normal aging, gene expression controlled by multiple epigenetic factors, including histone acetylation, is weakened. Interestingly, histone deacetylation can prolong lifespan by promoting autophagy and inhibiting oxidative stress and necrosis.^333^ However, many age‐related diseases are often characterized by lower levels of histone acetylation.^334^

The occurrence of various diseases, such as metabolic diseases, tumors, CVDs, neurodegenerative diseases, and immune diseases, is related to the imbalance of protein acetylation and deacetylation.^335^, ^336^ Studies have identified a large number of acetylated proteins in the cytoplasm and mitochondria, most of which are related to metabolism. Lysine acetylation affects the functions of metabolic enzymes by regulating their activity and stability.^321^ Loss of their acetylation regulation may lead to metabolic disorders and the accumulation or insufficient synthesis of some metabolic intermediates, resulting in metabolic‐related diseases.

### 3.1 Acetylation in development

Previous studies have shown that HDACs regulate histone acetylation to affect the proliferation, differentiation, apoptosis, migration, and synapse regeneration of nerve cells.^337^ *HDAC1* and *HDAC2* are essential for cortical lamination and play a crucial role in maintaining the progenitor pool during cortical development. Deletion of both *HDAC1* and *HDAC2* results in a deficiency in neocortical development.^338^ HDACs also play roles in the development and differentiation of various immune cells.^312^ Inactivating *HDAC3* during the double‐negative stages of thymocyte development will cause significant damage at the CD8 immature single‐positive (ISP) stage and the CD4/CD8 double‐positive stage, resulting in the production of few mature CD4(+) or CD8(+) single‐positive cells.^339^ Deletion of *HDAC3* in early B‐progenitor cells caused a defect in VDJ recombination and failure in B cell development.^340^ In addition, HDAC3 can also indirectly regulate the development and function of these immune cells through stromal cells or target cells interacting with immune cells. For example, HDAC3 is an important component of the Notch signaling pathway that regulates the development of medullary thymic epithelial cells (mTECs).^341^ Loss of *HDAC3* expression can lead to developmental arrest of mTECs with impaired T‐cell negative selection. However, whether the regulation of HDAC3 on the development of neurons and immune cells depends on its deacetylation function remains to be further studied.

### 3.2 Acetylation in aging

As a key metabolite, acetyl‐CoA is an important donor of acetylation modifications.^342^, ^343^ Previous studies have shown that fasting and caloric restriction (CR) reduce glucose‐derived metabolic flux and cytoplasmic acetyl‐CoA levels through ATP‐citrate lyase,^344^ which decreases p300 activity to stimulate long‐lived autophagy. However, increased nuclear acetyl‐CoA can promote lifespan by increasing the levels of histone acetylation.^343^, ^345^, ^346^ Sirtuins are epigenetic enzymes that are key regulators of aging and CR.^347^ In yeast, CR prolongs lifespan by increasing the activity of Sir2.^347^, ^348^ In mammals, the role of SIRT1–7 in extending lifespan is also largely based on their deacetylase functions.^349^

Nicotinamide mononucleotide (NMN) supplementation not only inhibits the aging‐associated increase in protein acetylation but also modulates fatty acid β‐oxidation, TCA cycle, and valine degradation. Aged livers show increased acetylation compared with young livers, but NMN supplementation decreases acetylation. These results reveal the potential of NMN in combating aging and aging‐related functional declines.^350^ Inflammatory aging of the brain is a hallmark of age‐related neurodegenerative diseases. Integrated analysis of H3K27ac and gene expression data in human and mouse brains shows that genes upregulated and downregulated with aging are correlated with different H3K27ac modification patterns.^351^ By using aging mouse models under inflammatory conditions, it has been found that the pattern recognition receptor NLRP3 is acetylated in macrophages and deacetylated by NAD^+^‐dependent sirtuins. Dysregulation of the NLRP3 inflammasome acetylation switch may be the cause of aging‐associated chronic inflammation.^352^

During the aging process of mesenchymal stem cells, histone acetylation on the promoters and enhancers of osteogenic genes, as well as the chromatin accessibility, decreases, which leads to the downregulation of osteogenic gene expression and a decrease in osteogenesis.^353^ Comparing the changes in sirtuins in experimental animals of three different age groups, young, middle‐aged and old, it has been found that the expression of sirtuin family proteins in skeletal muscle increases during the aging process, but acetylation is not effectively reduced, which is associated with a severe reduction in NAD^+^ content.^354^

In addition, the acetyltransferase KAT7 can promote H3K14ac‐related gene expression and induce cell senescence. Inactivation of KAT7 reduces H3K14ac and represses the transcription of p15INK4b, which attenuates the senescence of human peritoneal mesothelial cells.^355^ DNA damage can activate ATM and inhibit LARP7‐regulated SIRT1 activity, leading to increased p53 and p65 acetylation and transcriptional activity to promote cellular senescence. Activation of this pathway exacerbates aging and atherosclerosis in ApoE‐knockout mice, while inactivation of this pathway can reverse these phenotypes (Table 3).^356^

**TABLE 3:** Representative acetylation substrates and their functions in health and diseases.

Diseases and the biological processes		Substrates	Effects
Aging		NLRP3	SIRT2 and NLRP3 deacetylation prevent and can be targeted to reverse, aging‐associated inflammation, and insulin resistance.^352^
		H3K14	KAT7 promotes H3K14ac‐related gene expression and induces cell senescence.^355^
		p53, p65	DNA damage can increase p53 and p65 acetylation and transcriptional activity to promote cellular senescence.^356^
Metabolic disorders	Diabetes	IRS1	HDAC2 reduces IRS1 acetylation in hepatocytes, to reduce pancreatic insulin formation and secretion.^367^
	Obesity	H3K27	p300/CBP‐mediated H3K27ac in the PPARγ complex promotes adipogenesis.^385^
CVDs	Cardiometabolic diseases	CypD	Decrease of SIRT3 in failing hearts from patients with obesity and metabolic syndrome leads to CypD hyperacetylation, mitochondrial permeability transition pore opening, and cardiac dysfunction.^431^
		p53	Activation of SIRT1 protects against advanced glycation end products (AGEs)‐induced apoptosis in endothelial cells in diabetes through decreasing p53 acetylation.^432^
		MPC2	Increased MPC2 acetylation at K19/26 impairs mitochondrial pyruvate transport activity and metabolic inflexibility in Akita diabetic hearts.^433^
	Myocardial infarction	p53	p53 acetylation at K118 increases infarct size, and its inhibition promotes NOS3‐mediated cell survival and cardioprotection.^434^
		Prdx1	Tubastatin A (TubA) selectively inhibits HDAC6 and promotes Prdx1 acetylation at K197, which offers cardioprotection against injury in rats and H/R‐induced cell death in H9c2 cells.^435^
	Cardiac hypertrophy, remodeling and heart failure	NF‐κB	HDAC inhibitor TSA attenuates transverse aortic constriction (TAC)‐induced hypertrophy by regulating histone acetylation on promoters of NF‐κB target genes.^436^
		MHC	HDAC3 aggravates cardiac hypertrophy by deacetylating cardiac myosin heavy chain (MHC) isoforms.^437^
		H3	Prenatal exposure of PM2.5 leads to lower birth weight and cardiac hypertrophy in adulthood by increasing CBP/p300 and H3K9ac.^438^
	Hypertension	H3	In spontaneously hypertensive rats, HDAC inhibition suppresses cardiac hypertrophy and fibrosis through increasing H3 acetylation on promoters of mineralocorticoid receptor (MR) target genes.^439^
		SOD2	SIRT3 depletion causes hyperacetylation of mitochondrial SOD2 and overproduction of oxidative stress, which results in endothelial dysfunction, vascular inflammation, and hypertension in mice.^440^
	Cardiac arrhythmias	Connexin 43	Chronic tachypacing leads to abnormal ventricular activation and increases acetylation of connexin 43 in canines.^441^
Cancers	Cancer cell proliferation	H4K16	HDAC3 promotes histone H4K16ac, which acts on PI3K and enhances the interaction between LC3 and ATG4 to trigger autophagy that affects cancer cell proliferation.^420^
	GBM	PAK1	Phosphorylation of ATG5 at Thr101 in GBM is positively regulated by PAK1 acetylation, which promotes tumor growth.^423^
	PC	LDHA	K5 acetylation of LDH‐A is reduced in human PC, and K5 acetylation of LDH‐A inhibits LDH‐A activity.^425^
	HCC	H3K27	HDAC‐mediated suppression of FBP1 is correlated with decreased H3K27ac in the FBP1 enhancer. Treatment of HCC cells with HDAC inhibitors restores FBP1 expression and inhibits HCC cell growth.^426^
	Breast cancer	MORC2	MORC2 acetylation is associated with elevated NAT10 expression in breast cancer. Acetylated MORC2 binds to phosphorylation at H3^T11^ and contributes to DNA damage‐induced G2 checkpoint activation.^442^
	Pulmonary cancer	H3K27	H3K27 acetylation activated‐COL6A1 promotes osteosarcoma lung metastasis by repressing STAT1 and activating pulmonary cancer‐associated fibroblasts.^443^
	Prostate cancer	TPD52	Acetylation‐dependent regulation of TPD52 modulates CMA oncogenic function in prostate cancer.^444^
	PDAC	BCAT2	BCAT2 is acetylated at K44. K44R mutant promotes BCAA catabolism, cell proliferation, and pancreatic tumor growth.^430^
Neurodegenerative diseases	AD	H2B	The p300/CBP activator CSP‐TTK21 can rescue Aβ‐impaired synaptic plasticity induced by various pathways, presumably through reversing Aβ‐induced dysregulation of H2B acetylation and gene expression.^405^
	Axon dysfunction	Miro1	Deacetylation of Miro1 by HDAC6 blocks mitochondrial transport and mediates axon growth inhibition.^445^
	PD	SOD2, ATP synthase β	PGC‐1α/ERRα‐Sirt3 pathway protects against DAergic neuronal death by directly deacetylating SOD2 (K130) and ATP synthase β (K485) in PD.^416^

Abbreviations: AD, Alzheimer's disease; CVDs, cardiovascular diseases; GBM, glioblastoma; PC, pancreatic cancer; HCC, hepatocellular carcinoma; PDAC, pancreatic ductal adenocarcinoma; PD, Parkinson's disease.

### 3.3 Acetylation in metabolic disorders

Diabetes and obesity are related to mutations in the acetylation sites of metabolic enzymes.^357^, ^358^ Persistent hyperglycemia in diabetes can increase acetyl‐CoA and protein acetylation levels, which may impair protein functions.^359^ In diabetic rat models, organs with high protein acetylation are susceptible to diabetic complications.^360^ The acetylation level of NF‐κB in the hearts of diabetic rats is elevated, and the expression of Nrf2‐related genes and mitochondrial activity are impaired. Consequently, this results in the persistence of inflammation, impairs the functions of the heart to resist oxidative stress, and increases the risk of cardiovascular complications in diabetes.^361^ HDACs and sirtuins play key roles in diabetes by affecting insulin signaling and secretion.^362^, ^363^ The GLUT4 gene promoter is composed of an MEF2‐binding domain and domain I. Transcriptional activity is highest when MEF2 is bound to the MEF2‐binding domain and the GLUT4 enhancer GEF is bound to domain I.^364^ HDACs downregulate the transcription of MEF2‐related genes.^365^ HDAC2 reduces acetylation by binding to IRS1 in hepatocytes, thereby reducing insulin receptor‐mediated IRS1 tyrosine phosphorylation^366^ and downregulating pancreatic insulin formation and secretion.^367^ Notably, SIRT1 can enhance glucose‐induced pancreatic insulin secretion.^368^ HDACs and p300/CBP mediate STAT3 acetylation to regulate gluconeogenesis.^369^

The p300/CBP family and SIRT3/SIRT6 are involved in the process of obesity,^370^, ^371^, ^372^ and the histone acetylation level is positively correlated with adipogenic differentiation.^373^, ^374^, ^375^ p300/CBP in the PPARγ complex is the main enzyme that activates gene transcription, which can increase the expression of CEBPα and PPARγ and promote adipogenesis.^376^, ^377^, ^378^ This process has been associated with p300/CBP‐mediated H3K27ac.^379^, ^380^ p300/CBP double knockout mice develop severe lipodystrophy with hepatic steatosis, hyperglycemia, and hyperlipidemia.^381^ In a cardiac‐specific SIRT6 knockout mouse model fed a high‐fat diet (HFD), loss of SIRT6 function exacerbates cardiac injury, including left ventricular hypertrophy and lipid accumulation.^382^ Enzymes of the HDAC family are also involved in the regulation of obesity. For example, by regulating fat metabolism, HDAC3 can promote fat absorption and diet‐induced obesity.^372^ HDACs also inhibit adipogenesis by downregulating histone acetylation.^383^, ^384^ HDAC1 but not HDAC2 can inhibit adipogenesis by reducing CEBPα and PPARγ expression (Table 3).^385^

### 3.4 Acetylation in CVDs

The modulation of HDAC functions can improve CVDs such as cardiac hypertrophy, heart failure, arrhythmia, myocardial infarction, hypertension, atherosclerosis, and fibrosis.^386^, ^387^ Although both class I and class II HDACs have conserved HDAC domains, they have completely different functions in CVDs. Class I HDACs have procardiac hypertrophic effects, whereas class II HDACs are expressed in a relatively tissue‐specific manner and have anticardiac hypertrophic effects.^388^ HDAC7 is localized in the cardiac cytoplasm, and its overexpression induces the expression of cardiac hypertrophy and heart failure‐related genes such as *Nppa* and *Nppb*.^389^

Vascular endothelial dysfunction is the main cause of CVDs, and one of the characteristics of endothelial dysfunction is insufficient synthesis of nitric oxide (NO). The main enzyme responsible for the synthesis of NO in endothelial cells is endothelial nitric oxide synthase (eNOS). The interaction between SIRT1 and eNOS can activate eNOS by reducing its acetylation level, which promotes NO production and vasodilation.^390^ CKIP‐1 regulates physiological cardiac hypertrophy by inhibiting HDAC4 phosphorylation.^391^ In an AngII‐induced mouse model of pathological cardiac hypertrophy, the pan‐HDAC inhibitor (HDACI) emodin ameliorates hypertrophy by inhibiting the activity of class I, IIa, and IIb HDACs.^392^ HDACs are also involved in myocardial fibrosis. Overexpression of class I HDACs significantly enhances the proliferation of cardiac fibroblasts and the expression of proteins associated with fibrosis. Silencing of HDAC3 upregulates miR‐18a and reduces ADRB3 expression, thereby inhibiting cardiomyocyte fibrosis and hypertrophy.^393^ The HDAC8 inhibitor PCI34051 mediates the p38 MAPK pathway to alleviate isoproterenol‐induced cardiac hypertrophy and fibrosis^394^ and attenuates myocardial fibrosis induced by transverse aortic constriction in mice through downregulation of Ace1.^395^ The sirtuin family also plays key roles in preventing cardiomyocyte fibrosis, regulating cardiomyocyte apoptosis, improving cellular energy metabolism remodeling and inflammation, and maintaining cardiac homeostasis.^396^ Stimulation of SIRT3 reduces ROS and protein kinase levels and prevents cardiac hypertrophy, which may be a mechanism to inhibit cardiac remodeling.^397^ Last, p300 is a potential therapeutic target for heart failure. Mice with p300 knockout exhibit remarkable cardiac defects and embryonic lethality (Table 3).^398^, ^399^

### 3.5 acetylation in neurodegenerative diseases

The imbalance between acetylation and deacetylation processes is related to neurodegenerative diseases such as AD and HD.^400^, ^401^ Abnormal histone acetylation in AD affects the expression of memory‐related genes and dysregulates several signaling pathways, including cell differentiation, apoptosis, inflammation, and neuronal and vascular remodeling.^402^, ^403^ In a transgenic AD fly model, loss of Tip60 activity significantly increases the transcriptional expression of amyloid precursor protein (APP), leading to neuronal apoptosis, while overexpression of Tip60 HAT activity can potentially serve as a neuroprotective agent.^404^ p300/CBP is widely expressed in the nervous system. It has been proposed that inhibiting the activity of CBP/p300 acetyltransferase may affect the death of brain neurons and the long‐term memory of animals.^405^ Among various HDACs, HDAC2 modulates chromatin plasticity to regulate the expression of learning and memory‐related genes, and its dysregulation leads to the dysfunction of cholinergic nbM neurons, neurofibrillary tangle (NFT) pathology, and cognitive decline in AD.^406^ HDAC3 controls gene expression during the development and maintenance of neural stem cells,^407^ while HDAC4 may also play a role in the area of learning and memory. Selective deletion of HDAC4 in the brain leads to impaired long‐term synaptic plasticity.^408^ HDAC6 plays a leading role in neuronal health or dysfunction. Selective inhibition of HDAC6 can promote growth cone function, synaptic plasticity, transport, and autophagosomal degradation, which can help protect neurons.^409^ Notably, HDAC6 is significantly elevated in the brains of AD patients. Sirtuins restore protein microenvironmental homeostasis mainly by reducing toxic protein aggregates. They also improve neural plasticity by increasing gene transcription activity, which can reduce oxidative stress, enhance mitochondrial function, and improve learning and memory abilities.^410^ SIRT3 expression is significantly increased in the temporal cortex in AD patients.^411^ High SIRT3 expression can promote antioxidant effects in mutant HTT cells, enhance mitochondrial function, and exert neuroprotective effects in HD.^412^ In PD mice, a neuroprotective effect of SIRT3 has also been found.^413^ SIRT3 may play a protective role in neurons by scavenging free radicals in mitochondria.^414^ Decreased SIRT3 function increases mitochondrial oxidative stress and cell death in substantia nigra dopaminergic neurons in PD models.^415^ The expression of SIRT3 is significantly reduced in MPTP‐induced PD cell models, and overexpression of SIRT3 inhibits cell apoptosis. PGC‐1α can promote the transcription of SIRT3 and inhibit the loss of dopaminergic neurons (Table 3).^416^

### 3.6 Acetylation in cancers

Abnormal acetylation exists in various cancers.^310^ Most histones are in a hypoacetylated state in tumor cells, and mutations in the acetyltransferases CBP and p300 are often found in tumors. An imbalance in acetylation leads to dysregulated gene expression related to cancer cell proliferation, differentiation, migration, invasion, and apoptosis.^417^, ^418^ For example, H4K16ac alters the chromatin state and promotes gene transcription to regulate tumorigenesis and development.^419^ miR24‐2 inhibits histone deacetylase HDAC3 through miR675 to promote histone H4K16ac, which acts on PI3K and enhances the interaction between LC3 and ATG4, consequently triggering autophagy that affects cancer cell proliferation.^420^ The acetylation of the cytoskeleton is related to tumorigenesis. The acetylation of α‐tubulin, a component of the cytoskeleton, is an important indicator of microtubule stability. Tubulin is the target of many anticancer drugs.^421^ Tumors are resistant to apoptosis. PDCD5, a protein related to apoptosis, can bind to Tip60 and increase p53 acetylation at K120, which affects the expression of apoptosis‐related genes such as *Bax*.^422^ The acetylation of the hypoxia‐induced autophagy regulator PAK1 regulates the phosphorylation of ATG5 at Thr101 in GBM and is important for hypoxia‐induced autophagy and tumor growth.^423^

Increasing evidence shows that carcinogenesis is affected by metabolism in the body. Most metabolic proteins are substrates of lysine acetylation,^421^ such as ATM, ABL1, CDK9, BTK, and CDK1. PKM2 is the last rate‐limiting enzyme in the glycolytic pathway responsible for the conversion of phosphoenolpyruvate to pyruvate. In a high glucose environment, PCAF acetylates PKM2 at K305, which reduces its binding to the substrate PEP, inhibits its enzymatic activity and promotes its chaperone‐mediated autophagy and lysosome‐dependent degradation.^424^ The acetylation of LDHA at K5 inhibits its enzymatic activity and is recognized and mediated by the heat shock protein HSC70, which downregulates the level of LDHA. The acetylation level of LDHA at K5 in early PC tissues is significantly lower than that in adjacent tissues, suggesting that acetylation of LDHA at K5 may be related to the occurrence of PC.^425^ FBP1 is the rate‐limiting enzyme in gluconeogenesis and is lost in many types of cancer. Reduced FBP1 is associated with poor prognosis in HCC. HDAC‐mediated repression of FBP1 expression is associated with a reduction in H3K27ac in the FBP1 enhancer.^426^ PDC is located within the mitochondria and is responsible for the irreversible conversion of pyruvate to acetyl‐CoA. Phosphorylation of PDP1 at Tyr381 triggers SIRT3 to detach from the PDC center but recruits the acetyltransferase ACAT1 to the PDC center to acetylate PDP1 at K202 and PDHA at K321. This reconstructs the structure of the PDC center and inhibits PDC activity, further promoting tumor cell proliferation and growth.^427^ 6PGD is an important enzyme in the PPP. Acetylation of 6PGD at K294 promotes the formation of highly active 6PGD dimers, thereby further activating the 6PGD and PPP pathways to produce more ribulose‐5‐phosphate and NADPH for nucleic acid synthesis and resisting oxidative free radical damage.^428^ Fatty acid metabolism is important for tumor growth and metastasis. Acetylation of FASN in the fatty acid synthesis pathway promotes its degradation. The deacetylation process is regulated by HDAC3, which functions in the initiation and development of liver cancer.^429^ Furthermore, SIRT4 can regulate branched‐chain amino acid catabolism by deacetylating BCAT2 and promote PDAC growth (Table 3).^430^

### 3.7 Acetylation‐associated targeted therapies

Due to the important functions of acetylation in diseases, HDACIs have now shown good application prospects in the treatment of various diseases, such as heart disease, diabetes, and cancers.^446^ Currently, HDACIs can be divided into four classes, including short‐chain fatty acids (SCFAs) predominantly inhibiting class I HDACs (e.g., butyrate, phenylbutyrate, and valproate), hydroxamic acids inhibiting class I and II HDACs (e.g., trichostatin A (TSA) and suberoylanilide hydroxamic acid (SAHA)), cyclic tetrapeptides displaying class I HDAC selectivity in vitro, and benzamides inhibiting class I HDACs (e.g., RGFP136 and MS‐275).^447^ Restoring normal protein acetylation may be a new approach for the treatment of malignant tumors.^448^

Upregulation of HDAC expression is a characteristic of various malignant cancers,^449^ such as prostate cancer,^450^ gastric cancer,^451^ breast cancer,^452^ renal cancer,^453^ and Hodgkin's lymphoma.^454^ HDACIs can inhibit tumor cell proliferation by inducing cell differentiation, growth arrest, and apoptosis.^455^, ^456^ A variety of HDACIs have been approved or entered clinical trials. HDACIs not only show direct inhibitory effects on tumor cells but also overcome the resistance of tumors to other drugs, which makes the combination of HDACIs and other antitumor drugs possible.^457^

HDACIs have bidirectional effects on inflammation and anti‐inflammatory effects. HDAC inhibition may not only increase inflammation but also attenuate the expression of specific genes to reduce infiltrating inflammatory cells, leading to a beneficial result.^458^ Additionally, HDACIs can reverse neuronal degeneration and aging and enhance synaptic plasticity in mouse models. On the one hand, HDACIs have a direct influence on gene transcription by remolding histone acetylation. On the other hand, HDACIs can increase the acetylation of transcriptional regulators such as HNF4a to indirectly regulate gene expression.^459^ HDACIs have been found to exhibit neuroprotective effects on neurological diseases such as PD, AD, amyotrophic lateral sclerosis (ALS), and HD.^460^ For example, the class I HDACI valproic acid may exert neuroprotective effects by regulating the BDNF/TrkB signaling axis.^461^ In a cellular model of PD patients, selective inhibition of SIRT2 increases tubulin acetylation and improves microtubule‐mediated transport.^462^ Moreover, SIRT2 inhibitors such as AGK2, AK‐7, and AK1 have been demonstrated to decrease neuroinflammation and cytotoxicity induced by toxins or mutant protein aggregation.^463^ Additionally, the HDAC6 inhibitor tubastatin A, which increases autophagic flux and protects neurons in HD patients, is a potential drug for the treatment of HD.^464^

Deacetylase inhibitors are also used to treat metabolic disorders. SAHA has been shown to target eNOS uncoupling and oxidative stress in diabetes.^465^ TSA can prevent ischemia‐induced left ventricular remodeling by inhibiting TNF‐α transcription. In addition, it also enhances AKT phosphorylation to promote angiogenesis and cardiomyocyte survival.^466^ However, a SIRT6 inhibitor aggravates diabetes‐induced cardiomyocyte apoptosis and fibrosis in mice by increasing the levels of inflammatory factors and ROS.^467^

## 4 OTHER SCFA MODIFICATIONS

**FIGURE 6:**
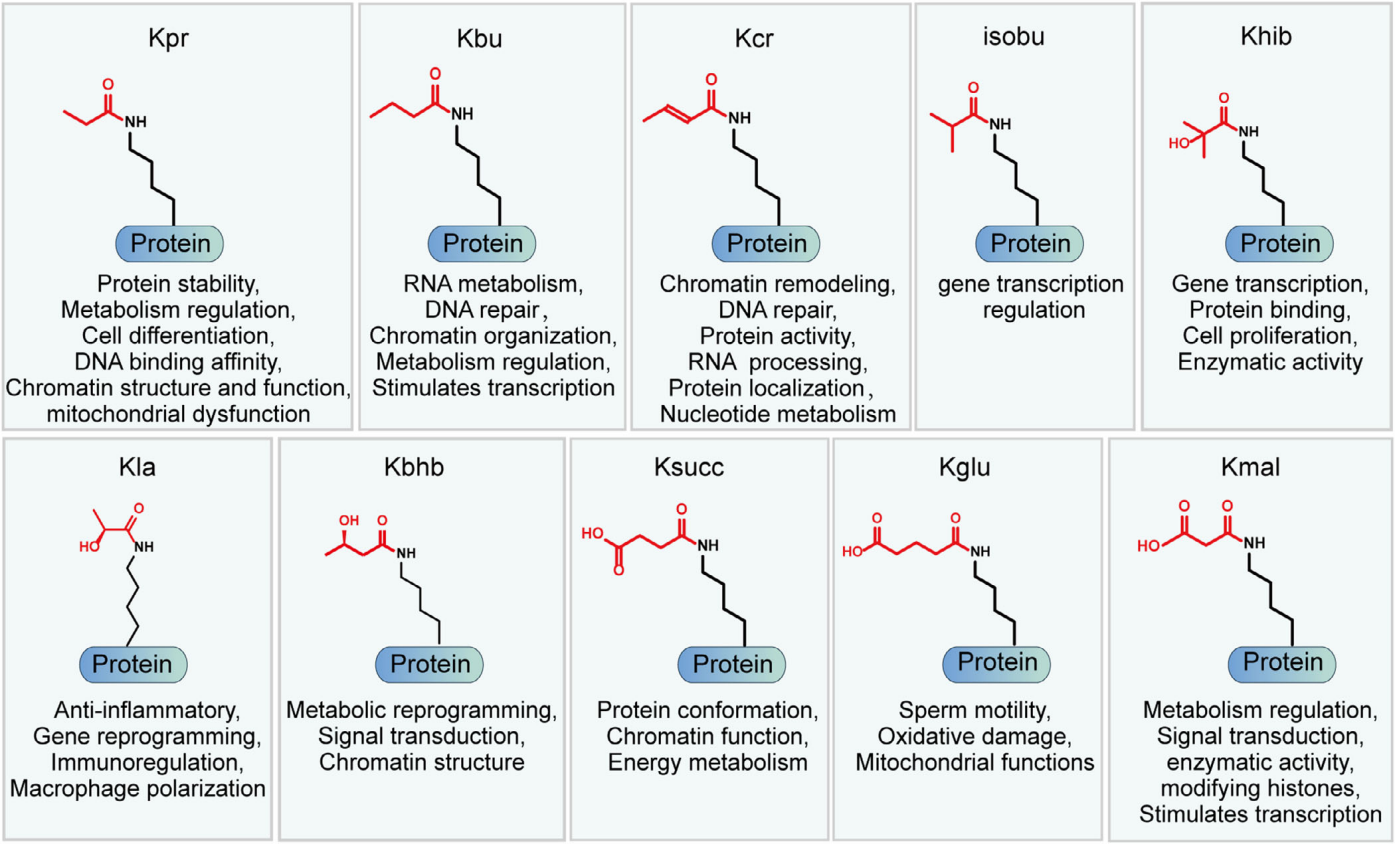
Chemical structures and representative biological functions of SCFA‐derived lysine acylation modifications, including propionylation (Kpr), butyrylation (Kbu), succinylation (Ksucc), 2‐hydroxyisobutyrylation (Khib), isobutyrylation (Kisobu), malonylation (Kmal), glutarylation (Kglu), crotonylation (Kcr), β‐hydroxybutyrylation (Kbhb), and lactylation (Kla).

**FIGURE 7:**
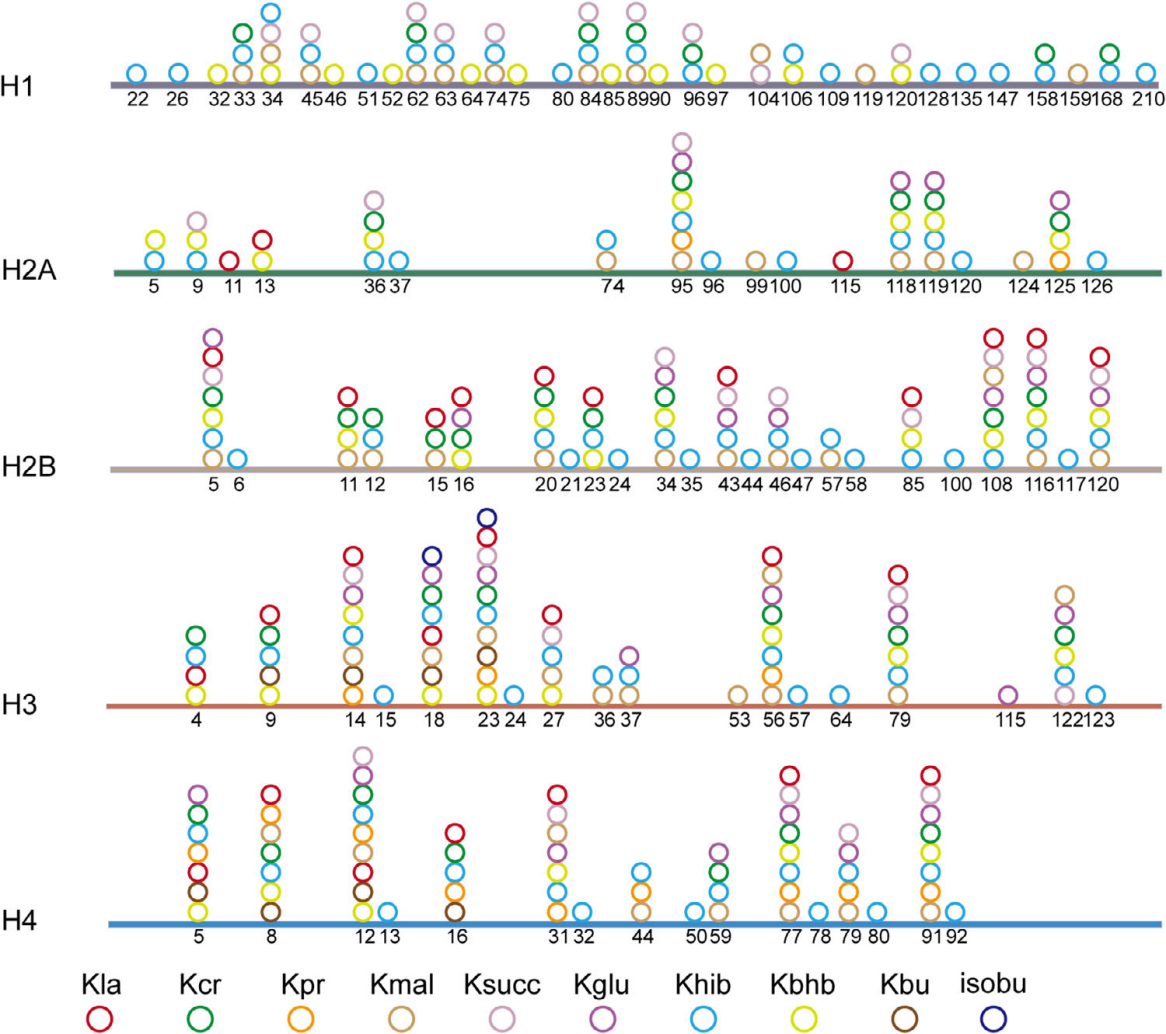
Distribution of reported lysine acylation modifications on histones.

SCFAs are products of food digestion and dietary fiber fermentation in the gut containing fewer than six carbons.^468^ Microbial‐derived metabolites have deleterious and beneficial effects on human health.^468^ They have a wide range of functions in signaling, cellular metabolism, and immunity.^469^ SCFAs can be transformed into acyl‐CoAs,^470^ which act as donors of protein lysine acylation.^471^ In addition to acetylation, the most extensively studied SCFA modification, introduced above, many other types of SCFA‐derived modifications have been identified, including propionylation (Kpr),^472^ butyrylation (Kbu),^472^ 2‐hydroxyisobutyrylation (Khib),^473^ succinylation (Ksucc),^474^ isobutyrylation (Kisobu),^475^ malonylation (Kmal),^476^ glutarylation (Kglu),^477^ crotonylation (Kcr),^478^ β‐hydroxybutyrylation (Kbhb),^479^ and lactylation (Kla) (Figure 6).^480^ To date, hundreds of histone acylation sites have been identified (Figure 7), and numerous studies have demonstrated the important roles that SCFA modifications play in both health and disease.^481^

**FIGURE 8:**
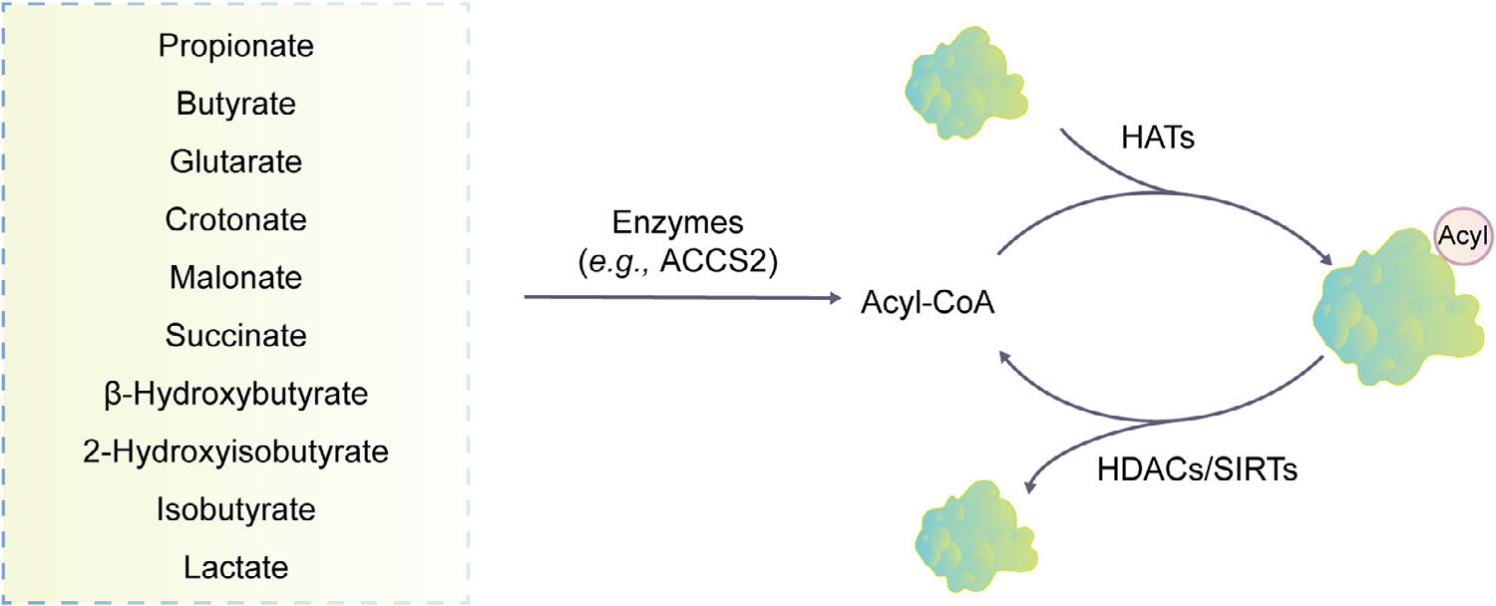
Origin and regulation of SCFA. SCFA‐derived lysine acylation modifications. The SCFA is converted into the corresponding acyl‐CoA in the presence of enzymes such as ACCS2. The acyl groups can be transferred onto proteins to modify the side chain of lysine residues by HATs. In addition, the acylated proteins can be deacylated by HDACs and sirtuins.

The acylation modifications derived from SCFAs are regulated by “writers” and “erasers.” Writers are enzymes that promote lysine acylation modifications, and erasers are enzymes that remove acylation modifications (Figure 8).^482^ In the past few years, an increasing number of studies have shown that classic acetyltransferases and deacetylases, such as p300, TIP60, HDACs, and SIRTs, also regulate other types of acylation modifications (Table 4).^34^, ^483^, ^484^

**TABLE 4:** The enzymatic specificities of different writers and erasers.

Acylations from SCFAs	Writers	Erasers
Propionylation	GCN5,^485^ PCAF,^486^ P300/CBP,^472^ MYST (MOF, MOZ, HBO1),^487^ KAT6A^488^	SIRT1, SIRT2, SIRT3, SIRT5^489^
Butyrylation	p300,^472^, ^490^ P300/CBP,^472^ GNAT^491^	SIRT3,^492^ SIRT5,^493^
Malonylation	NA	SIRT3,^493^ SIRT2, SIRT5^494^
Succinylation	KAT2A^495^	SIRT5,^483^ SIRT7^484^
2‐Hydroxyisobutyrylation	P300,^487^ Tip60, EP300^496^	SIRT5,^497^ HDAC2,^496^ HDAC3^498^
β‐Hydroxybutyrylation	P300/CBP, MYST, GNAT^499^	HDAC1, HDAC2, SIRT1–3^500^
Crotonylation	P300/CBP,^501^ GNAT, MYST,^502^ HBO1, KAT6A, MOF, PCAF, TIP60^503^	SIRT1–3, HDAC1–3, HDAC8^502^
Glutarylation	P300,^479^ KAT2A^504^	SIRT5,^477^, ^483^ SIRT7^505^
Lactylation	p300^480^	HDAC1–3^506^

Kpr is a widely distributed PTM. The propionyl‐CoA donor for Kpr is derived from odd‐chain fatty acid oxidation (FAO) and branched‐chain amino acid catabolism.^507^ Kpr is mainly found in proteins involved in energy production and conversion, participates in various metabolic processes, and plays an important role in protein breakdown.^508^, ^509^, ^510^ Histone propionylation, acetylation, and butyrylation levels also change in response to cellular metabolic changes, and these modifications regulate chromatin structure and function as important markers of such changes.^491^

Butyryl‐CoA is a donor of Kbu derived from even‐chain fatty acids.^511^ Kbu not only regulates transcription but also regulates RNA metabolism, chromatin organization, and DNA repair.^512^ The binding of a testis‐specific member, Brdt, can be inhibited by histone butyrylation, which affects the differentiation of male germ cells.^513^

Kisobu is a recently discovered isomeric modification of Kbu. Isobutyrylation and butyrylation are derived from different donors, isobutyryl‐CoA and butyryl‐CoA, whose biosyntheses are different in mammalian cells. Butyryl‐CoA is derived from the metabolism of fatty acids, while valine metabolism contributes to isobutyryl‐CoA production. Kisobu is also involved in the regulation of gene transcription.^475^

The crotonylation donor crotonyl‐CoA can be produced by ACCS2‐catalyzed crotonic acid metabolism^514^ or converted by the butyrate β‐oxidation pathway.^515^ During FAO, ACADS and ACOX3 are key enzymes that catalyze the conversion of butyryl‐CoA to crotonyl‐CoA,^516^ and Kcr is involved in several physiological processes in humans, such as DNA damage repair,^517^ chromatin reorganization, RNA processing, and regulation of protein activity and localization.^518^

Lactic acid serves as a carbon source in organisms and is the precursor of Kla,^480^, ^519^ which is derived from lactyl‐CoA generated by glycolytic conversion of glucose.^480^ Lactyl‐CoA is then transferred to the lysine side chain of proteins through transferases.^480^ Kla can be inhibited by glycolysis inhibitors and boosted by mitochondrial inhibitors or hypoxia, all of which affect lactate production.^492^Lactate stimulates histone Kla and influences gene transcription^480^ and is involved in important life activities such as anti‐inflammation,^520^ immune regulation,^521^ and gene reprogramming.^522^ In addition, histone Kla also inhibits the activation of inflammatory macrophages by promoting M2‐like polarization.^492^

Kmal refers to the addition of malonyl groups to lysine side chains. ^483^ Malonyl‐CoA, as a reactive donor of Kmal, can inhibit glycolysis‐related enzyme activities by modifying them.^523^ In addition, Kmal can modify many proteins and affect the related signaling pathways, including fatty acid synthesis and oxidation,^34^, ^524^ mitochondrial respiration,^524^ glycolysis,^524^, ^525^ and histones.^526^ Kmal also acts as a signal to regulate macrophage mRNA binding to promote inflammation. ^483^ In cells lacking FASN, malonyl‐CoA accumulation can lead to mTOR malonylation and affect mTORC1 signaling.^527^

Ksucc is a process of covalently attaching a succinyl group to the lysine side chain in an enzymatic or nonenzymatic manner.^479^ Ksucc occurs mainly in the mitochondria,^528^ where succinyl‐CoA is produced by amino acid metabolism or the TCA cycle.^529^ Moreover, succinyl‐CoA and Ksucc are highly abundant in tissues, such as the heart, brown adipose tissue and liver, with greater numbers of mitochondria.^530^ Ksucc participates in energy metabolism in vivo,^481^ causes protein conformation changes,^17^ and regulates nuclear function.^531^

Kglu, a reversible, dynamic and conserved modification, is produced by covalently binding glutaryl groups to lysine residues^504^ and occurs mainly in mitochondria.^532^ Kglu plays an important role in regulating protein structural changes,^532^ oxidative damage,^533^ mitochondrial functions,^534^ and sperm motility.^535^

2‐Hydroxyisobutyryl‐CoA is a potential donor for Khib. Tip60 and p300 are identified as 2‐hydroxyisobutyryltransferases, while HDAC2 and HDAC3 are de‐2‐hydroxyisobutyrylases.^473^, ^496^ Khib plays critical roles in the regulation of gene transcription, cell growth and cellular metabolism. It not only affects the binding interaction between histones and DNA^536^, ^537^ but also participates in the regulation of metabolic pathways such as glycolysis/gluconeogenesis and the TCA cycle. In addition, Khib also affects the motility of human sperm.^538^

3‐Hydroxybutyrate is a metabolic component of ketone bodies that provides energy for the heart and brain during periods of starvation.^499^, ^539^ Hypoglycemia leads to ketogenesis, producing β‐hydroxybutyrate.^479^ β‐Hydroxybutyrate forms covalent bonds with lysine side chains in proteins during ketogenesis, leading to Kbhb.^536^, ^540^ Kbhb is a sensitive indicator of changes in energy metabolism. Under starvation conditions, histone Kbhb levels in the mouse liver are significantly elevated, which can impact metabolic pathways such as amino acid catabolism.^536^ Histone Kbhb can promote the transcription of the *BDNF* gene.^536^ In addition, Kbhb also contributes to the regulation of chromatin structure.^512^

**FIGURE 9:**
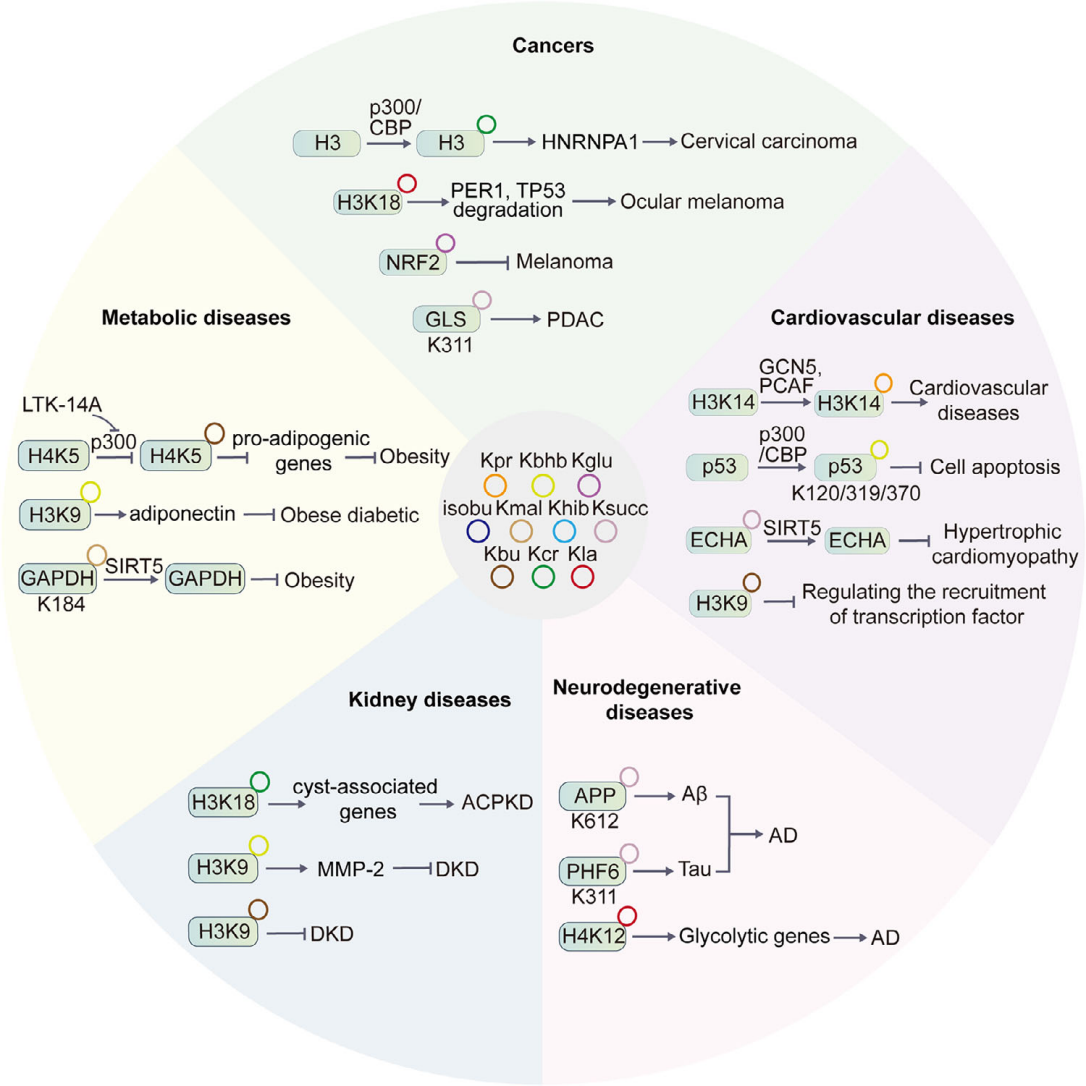
The functions of SCFA‐derived acylation modifications in various human diseases. Representative examples are shown.

Protein acylation not only regulates cellular processes such as gene transcription and cellular metabolism, but also plays a role in the regulation of health and disease.^541^ The microbiota‐dependent synthesis of many metabolites, particularly SCFAs, affects human health.^542^ SCFA modification, as an epigenetic mechanism, regulates key functions of various proteins related to growth, metabolism, cell differentiation and apoptosis, inflammation, aging, and angiogenesis. It plays a role in many diseases, including cancer, neurological and psychiatric disorders, CVD, diabetes, hepatitis, and kidney disease (Figure 9).^479^, ^503^, ^543^, ^544^

### 4.1 CVDs

SCFA modifications play a role in regulating the progression of CVDs through enzymatic switches and oxidative stress. Additionally, they can affect the cellular localization and PPIs of many cardioprotective proteins.^545^ Maintaining metabolic stability of SCFAs is essential for cardiac and vascular function.^543^ p53 Kbhb mediates the protective effect of β‐hydroxybutyrate on vascular cell senescence, possibly by reducing the expression of p21 and PUMA, which leads to cell growth arrest and reduced apoptosis.^499^ Kpr can cause FAO disorders and impair mitochondrial functions.^524^ H3K14pr is highly expressed in promoter regions of transcriptionally activating genes, including FAO‐related genes associated with CVD progression.^491^ SIRT5 defects lead to an increase in succinyl‐coA in the heart.^504^ Elevated levels of Ksucc can lead to hypertrophic obstructive cardiomyopathy,^528^ while oxidative stress caused by Kglu may be an important mechanism for inducing CVDs.^546^ A high‐fat diet causes adverse effects on cardiovascular health, especially under stressful conditions. The levels of H3k9bu affected by ACADS can moderate the expression of stress‐regulated genes.^547^

### 4.2 Metabolism‐associated diseases

Metabolic disorders cause dysregulated SCFA modifications, which can lead to various metabolic diseases.^17^, ^548^ Obesity can lead to sperm DNA damage and decreased sperm quality,^549^ and SCFA modifications cause male reproductive dysfunction in obese men. In high‐fat diet mice, acetylation and crotonylation decrease in the testes, while other metabolism‐related lysine acylations, including propionylation, malonylation, succinylation, glutarylation, 2‐hydroxyisobutyrylation, and benzoylation, increase.^550^ Obesity and metabolic syndrome accelerate the occurrence of osteoarthritis during the aging process, and SIRT5‐regulated malonylation may impair chondrocyte metabolism.^551^ The mechanism by which energy restriction improves fat metabolism may be involved in Ksucc. Acute fasting regulates Ksucc through SIRT5 to modulate lipid metabolism in adipose tissues and improve obesity.^552^ In addition, specific inhibition of p300‐mediated butyrylation at H4K5 by LTK‐14A in adipocytes and liver improves obesity.^490^

Decreased SCFA modifications, such as malonylation, butyrylation, and propionylation, are found in liver histones of obese mice induced by a high‐fat diet.^553^ Dapagliflozin treatment leads to elevated 3‐hydroxybutyrate in the plasma and adipose tissues of obese diabetic mice, which further induces H3K9 3‐hydroxybutyrylation to promote the expression of apolipoproteins in adipocytes. Apolipoproteins are anti‐inflammatory and antiatherosclerotic, indicating that 3‐hydroxybutyrate is protective against obesity‐associated diabetes by modulating H3K9 3‐hydroxybutyrylation.^554^ In addition, the dysregulation of Kmal in fatty acid oxidative metabolism can also lead to mitochondrial fatty acid metabolism diseases such as malonyl‐CoA synthetase ACSF3 deficiency.^532^ These studies provide additional evidence for the link between metabolic disorders and epigenetic regulation by SCFA modifications.

### 4.3 Kidney diseases

Kidney disease includes acute kidney injury (AKI) and chronic kidney disease (CKD), some of which progress to end‐stage renal disease (ESRD). SCFA modification, as a type of epigenetic mechanism, is involved in the progression of kidney disease.^481^ Histone lysine crotonylation was observed in mouse and human renal tubular cells, and histone crotonylation was observed to be increased in renal tissues during AKI. Crotonate supplementation can increase overall histone crotonylation and have a protective effect on the kidneys.^555^ Crotonyl‐CoA hydratase CDYL regulates the crotonylation of histone H3K18 and affects the disease process of ADPKD.^556^ Diabetic kidney disease (DKD) is the main cause of ESRD, and inflammation and fibrosis are key processes in its development. Butyrate inhibits the expression of renal inflammation and fibrosis genes through p300‐mediated histone Kbu and improves DKD.^557^ Other studies have found that crotonylation and 2‐hydroxyisobutyrylation also play a significant role in ESRD. By analyzing crotonylation and 2‐hydroxyisobutyrylation in PBMCs of patients with ESRD, it is speculated that crotonylation and 2‐hydroxyisobutyrylation may affect immune cell numbers and induce immune senescence, which may be due to regulation of the glycolytic/gluconeogenesis pathway and protein processing.^558^ In STZ‐induced diabetic SD rats, 3‐hydroxybutyrate treatment increases H3K9 3‐hydroxybutyrylation in the gene promoter to upregulate MMP‐2, which reduces collagen IV content and glomerular fibrosis.^559^

### 4.4 Cancers

Metabolic reprogramming is a common feature of cancer.^560^ The “Warburg effect” is an important feature of tumor cell metabolism.^548^ Tumor cells predominantly rely on aerobic glycolysis for energy production and metabolite synthesis, resulting in extracellular acidification due to lactate accumulation, which is a hallmark of cancer.^561^ Lactate is involved in the regulation of the tumor microenvironment, which promotes macrophage polarization into an M2‐like phenotype, thereby inhibiting the immune response in the tumor microenvironment.^480^ Histone Kla has been proven to promote the development of tumors. Histone Kla regulates the transcription of the m6A reader YTHDF2, which can recognize the m6A modification on the 3'UTR of the tumor suppressor genes *PER1* and *TP53* mRNA, resulting in their degradation and further impacting the development of melanoma.^562^ Kla is more abundant in gastric cancer tumor tissues than in adjacent normal tissues, indicating its potential as a prognostic indicator for gastric cancer.^563^ In PDAC, elevated tumor‐mediated Kla levels can enhance the expression of cancer‐associated fibroblasts (CAFs) to promote cancer cell invasiveness.^522^

Besides lactylation, other SCFA modifications also contribute to cancer development. In PDAC, SUCLA2‐coupled regulation of GLS succinylation promotes tumor growth.^564^ Lower levels of the mitochondrial protein GCDH result in Kglu of the TF NRF2, leading to cell death, indicating that GCDH pathway inhibition is a potential therapeutic strategy for melanoma treatment.^565^ Khib is widely distributed in PC, and treatment with the TIP60 inhibitor MG149 can significantly reduce the total Khib level in PC, which leads to the inhibition of PC cell proliferation, migration, and invasion.^498^ Kcr is also involved in cancer regulation, with decreased levels observed in liver, gastric, and kidney cancers and increased levels in thyroid, esophageal, and PC.^566^ In addition, p300‐mediated crotonylation enhances the expression of HNRNPA1, promoting HeLa cell malignancies.^567^ By knocking out HDACs or adding the HDACI TSA, the level of Kcr is increased, which inhibits the motility and proliferation of HCC cells.^566^ The level of Kcr in prostate cancer tissue is higher than that in adjacent tissues, and its level increases as the malignancy increases.^568^ Hyperpropionylation of H3K23 in the leukemia cell line U937 may serve as a stage‐specific biomarker in hematopoiesis and leukemogenesis.^569^ Moreover, propionate can induce global Kpr, which inhibits colon cancer development by upregulating the expression of MICA/B.^570^ Ksucc can affect the synthesis of thyroid hormones. Radiation‐induced thyroid cancer and cancer cell metastasis in apoptotic cell lines can be inhibited by Ksucc.^571^ Downregulation of TFAM can induce Kmal of mDia2 to promote its nuclear translocation, which further induces lung metastasis of mouse liver cancer.^572^

### 4.5 Neurological diseases

SCFA modifications are closely related to neurological function. In cases of syndromic intellectual disability, there is an alteration in histone H3K23 propionylation.^488^ The level of Kbu is significantly changed in the brains of rats with vascular dementia compared to the control group.^573^ Crotonylation and succinylation levels are increased in the cerebral cortex of mice with developmental disorders of the central nervous system (CNS).^574^ Neural excitation can modulate Kla levels in brain cells, suggesting that protein Kla in the brain may be associated with neuropsychiatric disorders.^575^ CDYL mediates histone crotonylation, which regulates gene transcription and promotes the development of stress‐induced depression in rodents, providing a potential therapeutic target for major depression.^576^ Lysine succinylation and malonylation are related to protein regulation, glycolysis, and energy metabolism. Mitochondrial dysfunction causes an imbalance in succinylation, which in turn leads to schizophrenia and other psychiatric disorders.^577^ Histone Kbhb is enriched in the promoter region of active genes and affects the organism by reprogramming the epigenetic map.^536^ Kbhb plays an important role in the development of neurological diseases, and 3‐hydroxybutyrate can be used to treat certain neurological diseases, such as epilepsy and AD.^578^, ^579^ Experiments in mice have shown that 3‐hydroxybutyrate may alleviate depressive behavior by increasing histone H3k9 3‐hydroxybutyrylation.^580^

In patients with AD, the succinylation levels of various mitochondrial proteins are decreased, while the succinylation of APP is increased. This disrupts the normal proteolytic process of APP and leads to abnormal protein deposition in the brain.^581^ In the AD mouse model, elevated levels of H4K12 lactylation in microglia activate the transcription of glycolytic genes, resulting in proinflammatory activation of microglia.^582^ During mammalian development, histone crotonylation and lactylation are widely distributed in the brain and play important roles in neurodevelopmental processes by contributing to transcriptome remodeling.^583^

## 5 LONG‐CHAIN FATTY ACID MODIFICATIONS

Straight‐chain fatty acids with 12 or more carbon atoms are referred to as long‐chain fatty acids (LCFAs), such as myristic acid, oleic acid, linoleic acid and palmitic acid.^584^ Similar to SCFAs, LCFAs can also be attached to the N‐terminus and amino acid side chains of proteins through the action of enzymes. The most commonly modified proteins by LCFAs are palmitoylated and myristoylated.^585^ Palmitoylation, the covalent attachment of the palmitoyl group to protein amino acid side chains, is a widespread modification in organisms^586^ and plays a crucial role in regulating protein translocation, localization and stability.^587^ Myristoylation, also known as N‐myristoylation, is an important PTM resulting from the covalent attachment of myristic acids to the N‐terminus of proteins catalyzed by N‐myristoyltransferase (NMT).^588^ It plays significant roles in innate immunity,^589^, ^590^ signal transduction,^591^ and cancer progression.^592^

### 5.1 Palmitoylation

**FIGURE 10:**
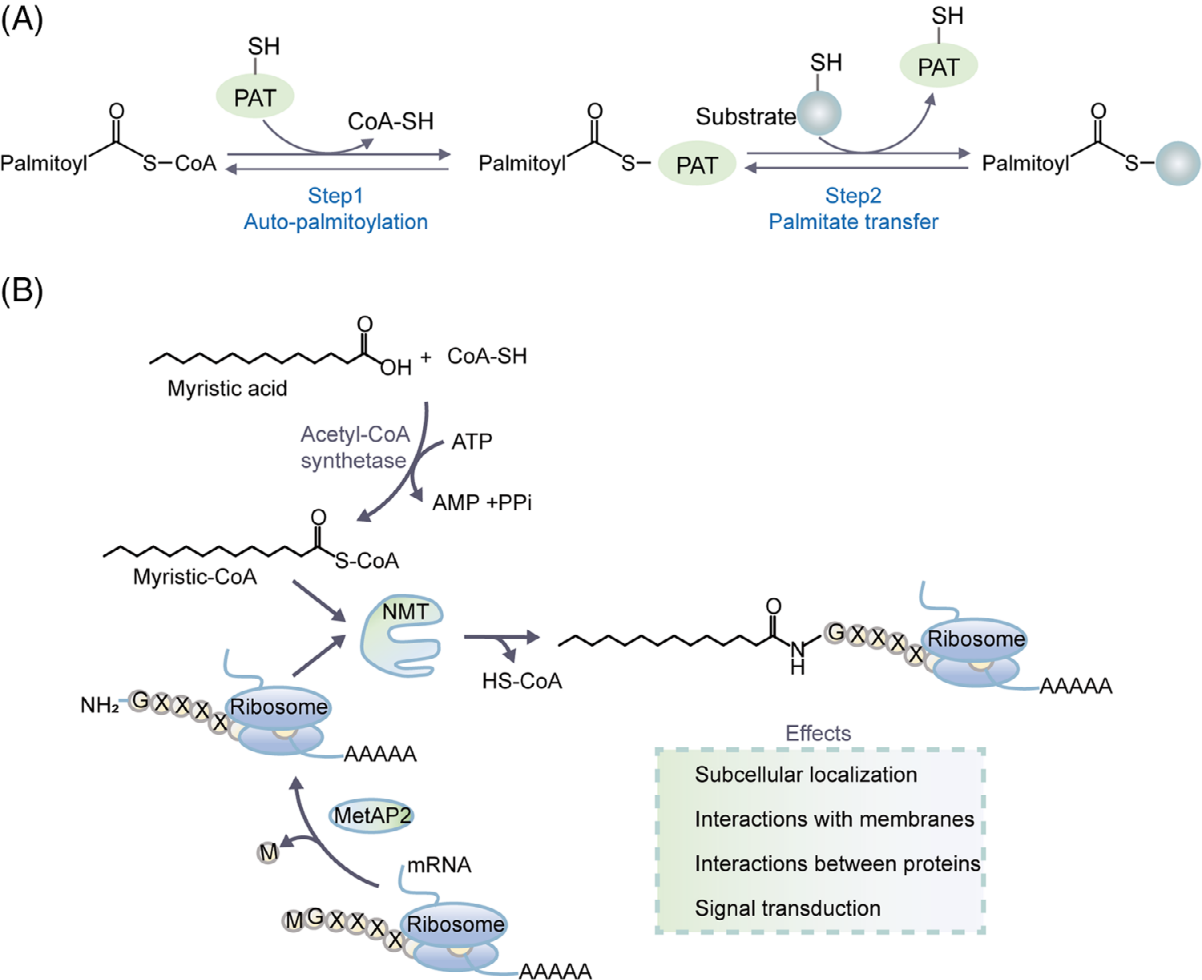
The process of S‐palmitoylation and N‐myristoylation. (A) The S‐palmitoylation and depalmitoylation process. Step1, palmitoyl acyltransferases (PATs) undergo auto‐palmitoylation, and the palmitoyl group is transferred to PAT; Step2, the palmitoyl group is transferred from PAT to protein substrates. (B) The process and functions of N‐myristoylation. Myristic acid and coenzyme A are converted to myristic acid coenzyme A by acetyl‐CoA synthetase. If the starting amino acid of the protein is methionine, it needs to be removed by methionyl aminopeptidase (MetAP2) before N‐myristoylation. N‐myristoyltransferase (NMT) is responsible for the addition of the myristoyl group to the glycine residue at the N‐terminal of the protein.

The regulation of protein palmitoylation balance is carried out by palmitoyl acyltransferases (PATs) and depalmitoylating enzymes (Figure 10A).^587^ PATs belong to the PAT family and contain a conserved DHHC (aspartic acid‐histidine‐histidine‐cysteine) motif, hence, they are referred to as DHHC‐PAT .^593^ These enzymes are also known as zinc finger‐containing DHHCs (ZDHHCs) as the DHHC motifs form zinc finger domains. Palmitoylation can be categorized into three types based on the way palmitoyl groups are attached to proteins: S‐type, N‐type, and O‐type.^594^ S‐type palmitoylation refers to the attachment of palmitoyl groups to the cysteine residues of proteins through an unstable thioester bond. N‐type palmitoylation refers to the attachment of palmitoyl groups to the amino groups of various amino acids (e.g., glycine, cysteine, lysine), while O‐type palmitoylation is the attachment of a few palmitoyl groups to the hydroxyl groups of serine or threonine. S‐palmitoylation, which dominates the majority of palmitoylated proteins and is a reversible process, represents typical palmitoylation. To date, 23 PATs have been discovered (Table 5).^595^ The thioester bond is hydrolyzed by depalmitoylases, leading to the dissociation of palmitoyl groups from cysteine residues. Five depalmitoylases have been found to date,^596^, ^597^, ^598^, ^599^, ^600^ including PPT1, PPT2, APT1, APT2, and ABHD17. PPT1, which is located mainly in lysosomes, is a thioesterase that mediates the depalmitoylation of various palmitoylated proteins in neurodegenerative diseases.^596^ PPT2,which has a different crystal structure from PPT1, is essential for depalmitoylation in protein degradation.^601^ APT1, which is mainly localized in the cytoplasm of yeast and mammalian cells, is a highly conserved α/β hydrolase containing the S‐H‐D catalytic triad and the G‐X‐S‐X‐G motif, with palmitoylated Ras proteins being its main substrates.^602^ APT2 is highly homologous to APT1.^603^ ABHD17 is essential for N‐Ras depalmitoylation and the relocalization of N‐Ras to internal cellular membranes.^604^

**TABLE 5:** Twenty‐three palmitoyl acyltransferases and their localization and functions.

Gene	Localization	Biological functions
ZDHHC1	ER	Ablation of ZDHHC1‐mediated p53 palmitoylation help cancer cells escape from the suppression of p53.^605^
ZDHHC2	ER, Golgi, dendritic vesicle in neuron	ZDHHC2 plays a critical role in inflammatory response of psoriasis^606^; ZDHHC2 is critical for the proliferation and the survival of B cells^607^; ZDHHC2 shows association with neurological diseases^608^, ^609^, ^610^; The C‐terminal domain of ZDHHC2 can regulate intracellular localization in neurons^611^; palmitoylation of SARS‐CoV‐2 spike protein is critical for virus entry.^612^
ZDHHC3	Golgi	Palmitoylation of PD‐L1 by ZDHHC3 inhibits antitumor immunity in vitro^613^; palmitoylation of ACE2 by ZDHHC3 is critical for the membrane‐targeting of extracellular vesicles secretion^614^; elevated expression of ZDHHC3 is correlated with poor survival in breast cancer^615^; high ZDHHC3 levels inhibits synaptic plasticity and memory in high‐fat diet (HFD) mice^616^; ZDHHC3 regulates the infection of primary and latent herpes simplex Virus 1.^617^
ZDHHC4	ER, Golgi	ZDHHC4 palmitoylates KAI1 and affects its localization, inhibiting angiogenesis^618^; GSK3β palmitoylation mediated by ZDHHC4 promotes tumorigenicity of GBM stem cells^619^; palmitoylation of D2R by ZDDHC4 is important for cell surface expression of the receptor.^620^
ZDHHC5	Plasma membrane, endosomes in dendritic shafts	ZDHHC5 plays important role in synaptic plasticity, cardiac function, cell adhesion, and fatty acid uptake; ZDHHC5 interacts with SARS‐CoV‐2 spike protein and affects their subcellular localization and pseudovirus entry^621^; Circ‐ZDHHC5 accelerates esophageal squamous cell carcinoma progression in vitro.^621^
ZDHHC6	ER	ZDHHC6‐mediated palmitoylation restrains FLT3‐ITD surface expression, signaling, and colonogenic growth in AML^622^; intracellular MYD88 palmitoylation by ZDHHC6 is a therapeutic target of sepsis^623^; ZDHHC6 palmitoylates NRas, contributing to its subcellular localization, and improves the downstream proproliferative signaling cascades in cancers.^624^
ZDHHC7	Golgi	ZDHHC7 regulates neuronal development and plasticity and modulates structural connectivity between hippocampus and medial prefrontal cortex in mice^625^; ZDHHC7 palmitoylates sex steroid hormone receptors and correlates with mental disorders^626^; palmitoylation of CD36 by ZDHHC7 are critical in NASH development.^627^
ZDHHC8	Golgi, dendritic vesicles, spines in neuronal cells	ZDHHC8 regulates seizure susceptibility in epilepsy^628^; ZDHHC8 palmitoylates scribble and Ras64B and controls growth and viability in *Drosophila* ^629^; ZDHHC8 and ZDHHC5 are present in dorsal root ganglion (DRG) axons and control retrograde signaling via the Gp130/JAK/STAT3 pathway.^630^
ZDHHC9	ER, Golgi	ZDHHC9‐mediated GLUT1 S‐palmitoylation promotes GBM glycolysis and tumorigenesis^595^; ZDHHC9 is essential for dendrite outgrowth and inhibitory synapse formation^631^; ZDHHC9 plays a critical role in intellectual disability.^632^
ZDHHC11	ER	ZDHHC11 is a positive modulator in NF‐κB signaling^633^; ZDHHC11 mediates MITA‐dependent innate immune responses against DNA viruses^634^; ZDHHC11 is a critical novel component of the oncogenic Myc‐miR‐150‐MYB network in Burkitt lymphoma.^635^
ZDHHC12	ER, Golgi	CLDN3 palmitoylated by ZDHHC12 contributes to plasma membrane localization and protein stability of CLDN3, thus promoting the progression of ovarian cancer^636^; ZDHHC12 can promote the proliferation and migration of glioma cells^637^; palmitoylation of gephyrin by ZDHHC‐12 contributes to coordinated neurotransmission.^638^
ZDHHC13	ER, Golgi	MC1R palmitoylation mediated by ZDHHC13 activates MC1R signaling, and affects senescence and melanomagenesis^639^; Drp1 palmitoylation by ZDHHC13 impacts brain bioenergetics and anxiety^640^; ZDHHC13 regulates skin barrier development by controlling protein stability^641^; ZDHHC13 regulates mitochondrial functions and metabolism in liver.^642^
ZDHHC14	ER	Palmitoylation induced by ZDHHC14 is of vital importance in control of neuronal excitability^643^; the expression of ZDHHC14 is inhibited, leading to increased proliferation and decreased apoptosis in coronary artery disease^644^; ZDHHC14 is involved in the palmitoylation of SARS‐CoV‐2 spike protein and contributes to virus entry.^612^
ZDHHC15	Golgi	ZDHHC15 mutations lead to psychiatric diseases^645^; ZDHHC15‐mediated palmitoylation may be a novel regulatory mechanism of dopamine in the striatum of mice^646^; ZDHHC15 regulates the formation of dendrite morphology and excitatory synapse^647^; ZDHHC15‐mediated GP130 palmitoylation is critical in the growth and self‐renewal of GBM stem cells.^648^
ZDHHC16	ER	Reduced ZDHHC16 contributes to p53 activation in GBM^649^; ZDHHC16 plays a crucial role in regulating neural stem/progenitor cells proliferation during zebrafish telencephalic development^650^; ZDHHC16 is involved in early stages of DNA damage response.^651^
ZDHHC17	Golgi, intracellular vesicles, presynaptic terminals	ZDHHC17 activates JNK and p38 MAPK and drives multiforme development and malignant progression in GBM^652^; ZDHHC17 interacts with CALCOCO1 and mediates selective Golgi autophagy^653^; ZDHHC17 is involved in the control of somal and distal axon integrity.^654^
ZDHHC18	Golgi	ZDHHC18 negatively regulates cGAS‐mediated innate immunity through palmitoylation^655^; MDH2 palmitoylation by ZDHHC18 sustains mitochondrial respiration and promotes the progress of ovarian cancer^656^; ZDHHC18 can regulate the cellular plasticity of glioma stem cells and contributes to their survival.^657^
ZDHHC19	ER	Zdhhc19 is dispensable for spermatogenesis and sperm functions in mice^658^, ^659^; ZDHHC19 accelerates tumor progression through wnt/β‐catenin pathway in osteosarcoma^660^; Flotillin‐1 palmitoylation turnover by APT‐1 and ZDHHC‐19 promotes cervical cancer progression.^661^
ZDHHC20	Plasma membrane	ZDHHC20‐mediated palmitoylation controls SARS‐CoV‐2 membrane lipid organization and enhances its fusion capacity^662^; palmitoylation by ZDHHC20 targets ORAI1 channels to lipid rafts for efficient Ca^2+^ signaling in immune responses^663^; palmitoylation of IFITM3 by ZDHHC20 enhances its antiviral activity.^664^
ZDHHC21	Golgi, plasma membrane	5‐HT1AR is palmitoylated by ZDHHC21 and reduced 5‐HT1AR palmitoylation is involved in depression^665^; ZDHHC21 mediates signaling events required for gut hyperpermeability induced by inflammation^666^; DHHC21 can palmitoylate α1 adrenergic receptor and regulate vascular functions^667^; DHHC21 mediates endothelial dysfunction in systemic inflammatory response syndrome.^668^
ZDHHC22	ER, Golgi	Palmitoylation of mTOR by ZDHHC22 can restrain breast cancer growth^669^; ZDHHC22 interacts with CCN3 and affects neuronal axon growth.^670^
ZDHHC23	ER, Plasma membrane	ZDHHC23 dynamically regulates the functional coupling with β1‐subunits and may be involved in cell‐specific control of ion‐channel physiology^671^; ZDHHC23 acts as potential regulators of tumor‐infiltrating immune cells and glioma progression.^672^
ZDHHC24	ER	High mRNA expression is an unfavorable prognostic marker in GBM.

Abbreviation: ER: endoplasmic reticulum

Palmitoylation is essential for protein localization. The palmitoylation of Cdc42 at Cys188 plays a crucial role in its localization to the plasma membrane, which regulates gene transcription and neuronal morphology in hippocampal neurons.^673^ The precise localization of calcineurin CNAβ1 and CD36 is also regulated by palmitoylation.^674^ Palmitoylation enhances the hydrophobicity of CD36,^675^ increasing its ability to bind to the membrane and absorb fatty acids.^676^ In contrast, inhibiting CD36 palmitoylation reduces its hydrophobicity and localization to the cytoplasmic membrane.^677^ The palmitoylation of CD36 is precisely regulated by DHHC4 and DHHC5.^678^ In addition, the palmitoyltransferase ZDHHC5 mediates the palmitoylation of NOD1/2 to promote its membrane recruitment and immune signaling, which are extremely important for microorganisms to establish an effective immune response.^679^

**FIGURE 11:**
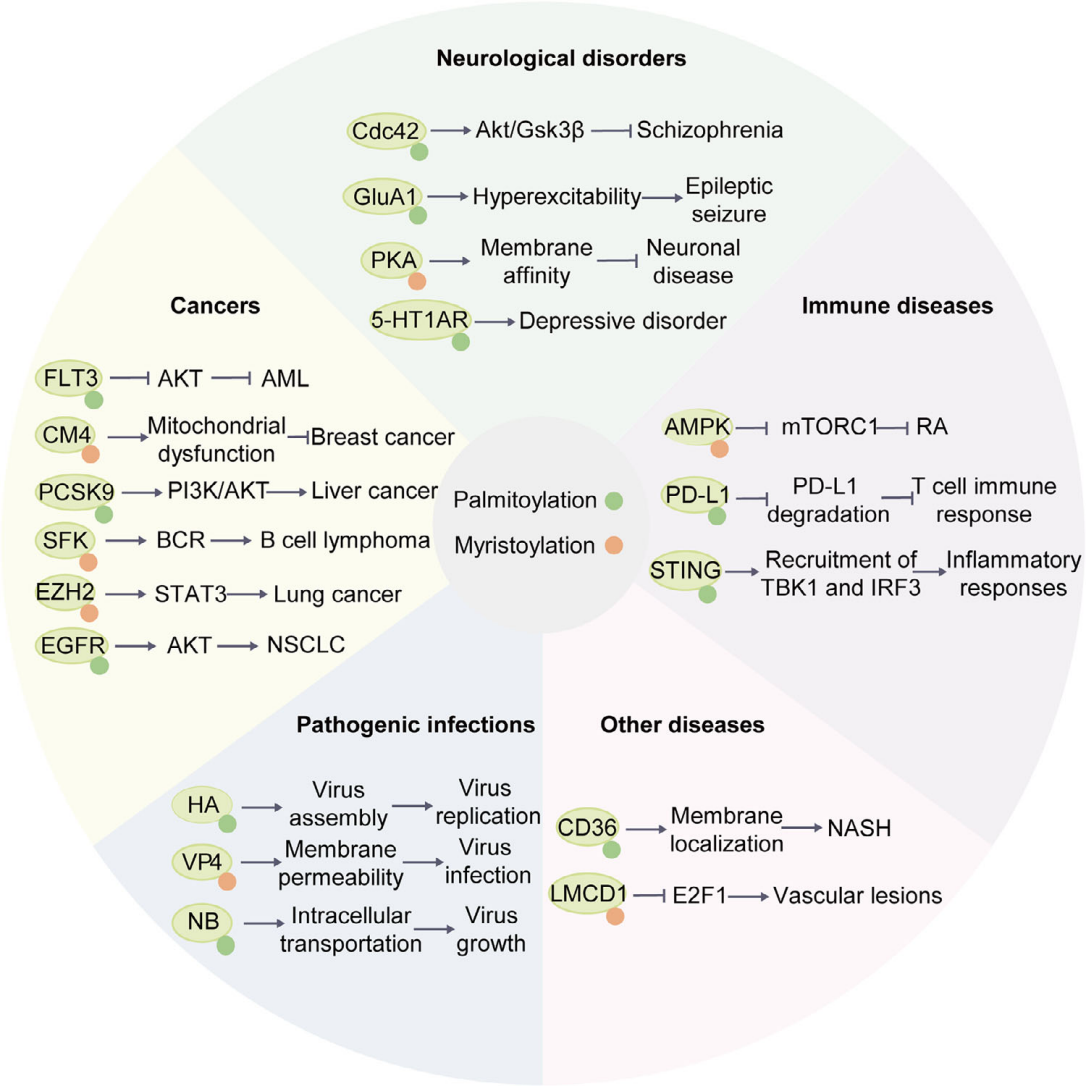
The functions of LCFA‐derived acylation modifications in various diseases, including palmitoylation and myristoylation. Representative examples are shown.

Cellular palmitoylation maintains a dynamic balance to ensure normal life activities, and any disruptions of this balance can lead to various diseases, such as autoimmune diseases,^613^ neurodegenerative diseases,^593^ T2DM,^33^ and nonalcoholic fatty liver disease,^677^ tumors,^680^, ^681^, ^682^, ^683^ Friedreich ataxia, and peripheral artery disease.^684^, ^685^ Elevated palmitoylation of NOD2 mutants in autoinflammatory diseases leads to inflammation and inhibits autophagic degradation (Figure 11).^686^ In autoimmune diseases, palmitoylation activates the STING signal associated with the type I IFN response and induces the expression of inflammatory genes through recruitment of TBK1 and IRF3. Treatment with the palmitoylation inhibitor 2‐bromopalmitate (2‐BP) can abolish the type I IFN response by inhibiting the palmitoylation of STING.^687^ In major depressive disorder, deletion of ZDHHC21 reduces palmitoylation of 5‐HT1AR and affects its signaling function.^665^ Reduced palmitoylation of Cdc42 in ZDHHC8‐deficient neurons interferes with Akt/Gsk3β signaling, leading to schizophrenia.^688^ Increased palmitoylation in the brains of HD mice can alleviate the anxiety and depression behaviors of mice^689^ and reduce cytotoxicity in YAC128 neurons.^32^ CD36 palmitoylation on the plasma membrane in nonalcoholic steatohepatitis (NASH) is significantly increased, but inhibition of CD36 palmitoylation protects against NASH in mice.^677^ Aberrant protein palmitoylation mediates cell barrier disruption and leads to spermatogenesis dysfunction in spermatodysplastic patients.^690^ ZDHHC17 mediates palmitoylation of Oct4A in human GBM and protects it from lysosomal degradation, which maintains tumorigenicity.^691^ Palmitoylated PCSK9 can activate the PI3K/AKT pathway, confer drug resistance in HCC cells, and promote cancer cell proliferation.^692^ In addition, palmitoylated EGFR in TKI‐resistant EGFR‐mutant NSCLC cells positively regulates FASN and further promotes cancer cell growth through the Akt pathway.^693^

At present, various therapeutic strategies have been developed to address diseases caused by imbalanced palmitoylation. FLT3 is palmitoylated in primary human AML cells, which hinders the activation of AKT signaling and AML progression. A novel therapeutic strategy has been developed for FLT3‐ITD^+^ leukemia by promoting FLT3 depalmitoylation.^622^ The palmitoyltransferase ZDHHC3 mediates the palmitoylation of PD‐L1, which further inhibits PD‐L1 ubiquitination and degradation. The palmitoylation inhibitor 2‐BP can lower PD‐L1 palmitoylation and boost PD‐L1 degradation through the lysosomal pathway, thereby increasing the immune response of T cells against tumors.^613^ Furthermore, 2‐BP can block STING palmitoylation and impair the type I IFN response.^687^ ABD957 acts as a potent and selective inhibitor of ABHD17 depalmitoylase. Specifically, it inhibits N‐Ras depalmitoylation in AML cells and disrupts the balance of N‐Ras palmitoylation, suggesting ABHD17 as a promising target for the treatment of N‐Ras mutant tumors.^604^

### 5.2 Myristoylation

Protein myristoylation is a process mediated by NMT, with the majority of this transfer occurring at the amino group of glycine.^585^, ^694^ On rare occasions, it takes place at the side chain of lysine.^695^ Until now, no demyristoylase has been discovered, but a study has shown that a cysteine protease IpaJ expressed by Shigella bacteria can cleave myristoylglycine from the N‐terminus of host proteins, serving a similar function as a hypothetical demyristoylase.^696^ The N‐terminus of the myristoylated protein contains a conserved sequence Met‐Gly‐XXX‐Ser/Thr (XXX could be any natual amino acid). The N‐terminal amino acid methionine must be removed by methionine aminopeptidase to expose glycine before myristoylation (Figure 10B).^697^ There are two NMTs (NMT1 and NMT2) in humans, NMT1 and NMT2, which have 77% sequence similarity and partially overlapping substrates and biological functions.^695^, ^698^

Myristoylation affects the subcellular localization of proteins and regulates PPIs and protein–membrane interactions. Myristoylated proteins located on the membrane can trigger subsequent cellular responses by sensing and transmitting signals.^699^, ^700^ For example, the myristoylation of the signal peptide of the virus envelope glycoproteins is essential for the fusion of the virus with the cell membrane and promotes the virus infection of host cells.^701^ EV71 has a myristoylation modification site on the glycine residue of VP4, which improves membrane permeability. When this modification is missing, the viral genome replication is impaired.^702^ ZYG11B and ZER1 are E3 ligase complexes that control the quality of N‐myristoylated proteins.^585^

N‐myristoylation endows proteins with stronger hydrophobicity, and the disruption of cellular N‐myristoylation balance may lead to the occurrence of malignant tumors, CVDs, and immune diseases.^699^, ^703^, ^704^ N‐myristoylation of EZH2 promotes phase separation of EZH2 with its substrate STAT3, leading to the activation of STAT3 signaling and growth of lung cancer cells, making N‐myristoylation of EZH2 a potential target for lung cancer therapy.^705^ In rheumatoid arthritis (RA), T cell deficits in NMT1 can lead to inflammation in synovial tissue due to impaired lysosomal transfer and AMPK activation.^704^ During vascular lesions, N‐myristoylation of LMCD1 specifically suppresses E2F1 and NFATc1, resulting in increased CDC6 and IL‐33, which further affects VSMC proliferation and migration.^706^

A variety of treatments have also been developed for diseases caused by N‐myristoylation imbalance. NMT1 is significantly upregulated in bladder cancer, and high NMT1 expression is linked to poor patient prognosis. NMT1 mediates the myristoylation of LAMTOR1 at Gly2 to increase LAMTOR1 stability and lysosomal localization, which is critical for amino acid sensing and mTORC1 activation. The inhibitor B13 can abrogate the functions of NMT1 and suppress tumor growth, suggesting that targeting NMT1 is a potential treatment for bladder cancer.^707^ B13 inhibits NMT1 activity by blocking Src myristoylation and reducing its cytoplasmic membrane localization, which inhibits prostate cancer cell proliferation.^708^

## 6 METHYLATION

Protein methylation, formed by transferring a methyl group from S‐adenosylmethionine (SAM) to a specific methyl acceptor, usually occurs at the side chains of lysine, arginine, histidine, asparagine, and glutamine, among which the methylation of lysine and arginine is the most common.^709^, ^710^

**FIGURE 12:**
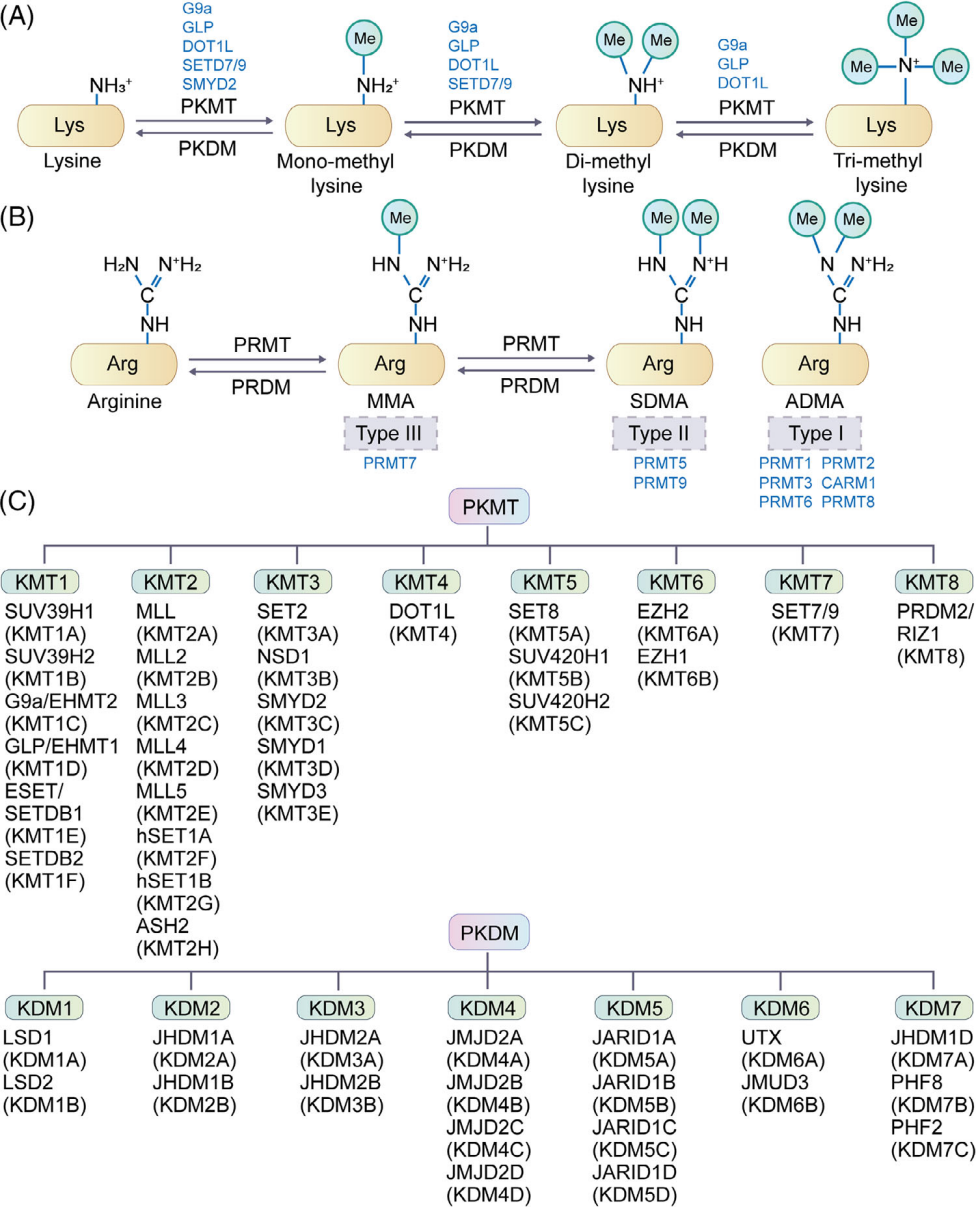
The methylation and demethylation process. (A) Lysine residues undergo mono‐, di‐ or trimethylation through the addition of a methyl group to its side chain. (B) There are three types of methylation occurring at the side chain of arginine, including monomethylated arginine (MMA), asymmetric dimethylated arginine (ADMA), symmetric dimethylated arginine (SDMA). (C) The common lysine methyltransferases and demethylases are listed.

Lysine methylation, mediated by protein lysine methyltransferases (PKMTs), has three different methylation forms, including monomethylation (Kme1), dimethylation (Kme2), and trimethylation (Kme3) (Figure 12A), linked to heterochromatin formation, X chromosome inactivation, and transcriptional silencing or activation.^711^, ^712^ PKMTs can be divided into two broad categories. One category is methyltransferases with a conserved SET domain, and most of the known PKMTs belong to this broad category.^713^ Another class is methyltransferases without the SET conserved domain, and most of this class of methyltransferases belongs to the seven‐β‐strand methyltransferase family. This PKMT family is characterized by a twisted β‐fold structure and can affect chromatin structure and gene regulation expression by modifying lysine sites at specific positions of proteins.^714^

Lysine methylation is a reversible modification. According to the different types of catalytic reactions, lysine demethylases can be mainly divided into two families: LSDs and Jumonji C (JmjC) domain‐containing demethylases (Figure 12C). The discovery of the first lysine demethylase LSD1 in 2004, also known as KDM1A or AOF2, was a milestone.^715^ In 2005, the first Jumonji C domain‐containing lysine demethylase KDM2A was reported and was mainly responsible for the demethylation of H3K36.^716^, ^717^ There are two LSD enzymes (LSD1 and LSD2) encoded in the human genome. LSD enzymes have the amine oxidase catalytic domain commonly found in metabolic enzymes and the SWIRM domain associated with chromatin binding that stabilizes the overall structure of the protein.^718^, ^719^ LSD1/2 can only demethylate monomethylated and dimethylated lysines. The demethylation of trimethylated lysines requires demethylases containing the Jumonji C domain.^720^ To date, approximately 30 Jumonji C domain‐containing proteins have been identified in the human genome.^721^ The family of demethylases containing the Jumonji C domain can hydrolyze methyl groups on monomethylated, demethylated, and trimethylated lysines.^720^

Protein arginine methyltransferases (PRMTs) mediate arginine methylation^722^ and can be divided into three types: monomethylarginine (MMA), asymmetric dimethylarginine (ADMA), and symmetric dimethylarginine (SDMA).^723^ To date, nine PRMTs have been identified, among which type I arginine methylases can catalyze the formation of MMA and ADMA, including PRMT1, PRMT2, PRMT3, PRMT4, PRMT6, and PRMT8; type II arginine methyltransferases include PRMT5 and PRMT9, which can catalyze the formation of MMA and SDMA; and the type III arginine methyltransferase is PRMT7, which only catalyzes the formation of MMA (Figure 12B).^23^

Histone arginine demethylation is mainly accomplished by two enzymes. One is PAD4, which plays a critical role in regulating the methylation of arginine residues on histones by catalyzing the conversion of methyl‐arginine to citrulline, resulting in the release of methylamine. PAD4 targets various sites in histones H3 and H4, including those that are methylated by coactivators CARM1 (such as H3 Arg17) and PRMT1 (such as H4 Arg3).^724^ Another enzyme is a JmjC domain‐containing JMJD6. JMJD6 is a specific histone arginine demethylase dependent on Fe^2+^ and ketoglutarate^725^. JMJD6 can catalyze the demethylation of histones H3R2 and H4R3 and convert them into formaldehyde through hydroxylation^726^. JMJD6 affects the demethylation of monomethylated, symmetric dimethylated and asymmetric dimethylated arginine residues.^711^, ^727^

**FIGURE 13:**
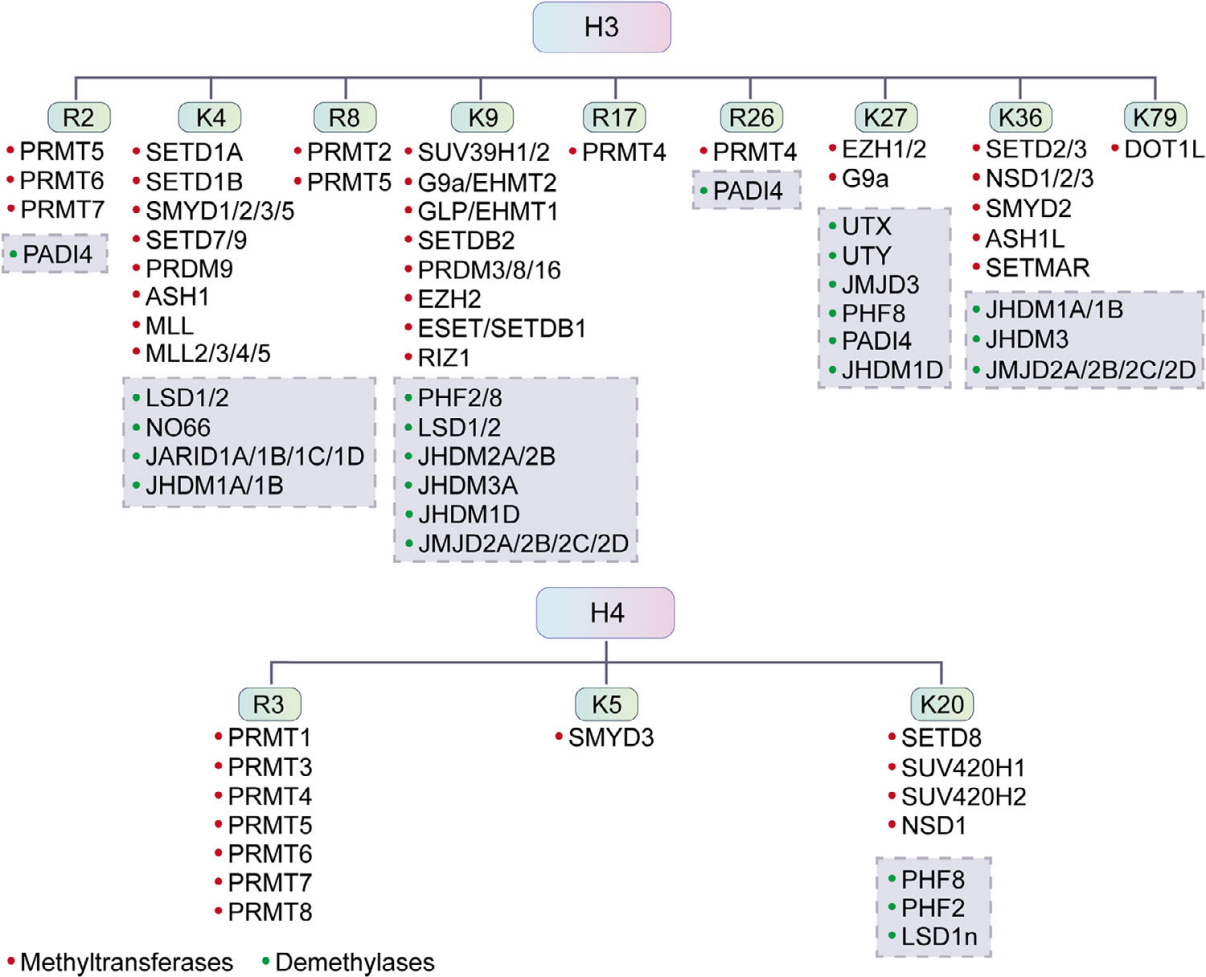
Protein methylation on histone H3 and H4 and their regulatory enzymes. The same residues can be regulated by multiple methylases and demethylases.

Histone methylation refers to the methylation of the lysine or arginine side chains of histone H3 or H4 mediated by histone methyltransferase (HMT), and some of them also occur on histidine residues.^728^ The most widely studied histone lysine methylation sites include H3K4, H3K9, H3K27, H3K36, H3K79, and H4K20, and the most widely studied histone arginine methylation sites include H3R2, H3R8, H3R17, H3R26, and H4R3 (Figure 13).^728^ Methylation of H3K4, H3K36, and H3K79 is generally associated with transcriptional activation of genes, whereas methylation of H3K9, H3K23, H3K27, H3K56, and H4K20 is generally related to transcriptional repression of genes.^729^, ^730^ Notably, different methylation states of the same lysine residue, such as mono‐, di‐, and trimethylation, may play different roles in chromatin state and gene transcriptional regulation.^731^

PRMT and PKMT can not only catalyze the methylation of histones, but also catalyze the methylation of nonhistone proteins.^732^ For example, the DNA damage response proteins MRE11 and 53BP1 can be methylated by PRMT1 to regulate its DNA exonuclease activity and localization at DNA damage sites.^733^ Some immunomodulatory proteins, such as Vav1 and NIP45, can also be modified by arginine methylation.^734^ In addition, KMT1C, KMT1D, and KMT1E in the KMT1 family; KMT2F in the KMT2 family; KMT3B, KMT3C, and KMT3E in the KMT3 family; KMT5A in the KMT5 family; and KMT7 have also been reported to catalyze nonhistone lysine methylation.^735^, ^736^, ^737^, ^738^ For example, KMT3C/SMYD2 methylates Rb at Lys860.^739^

**FIGURE 14:**
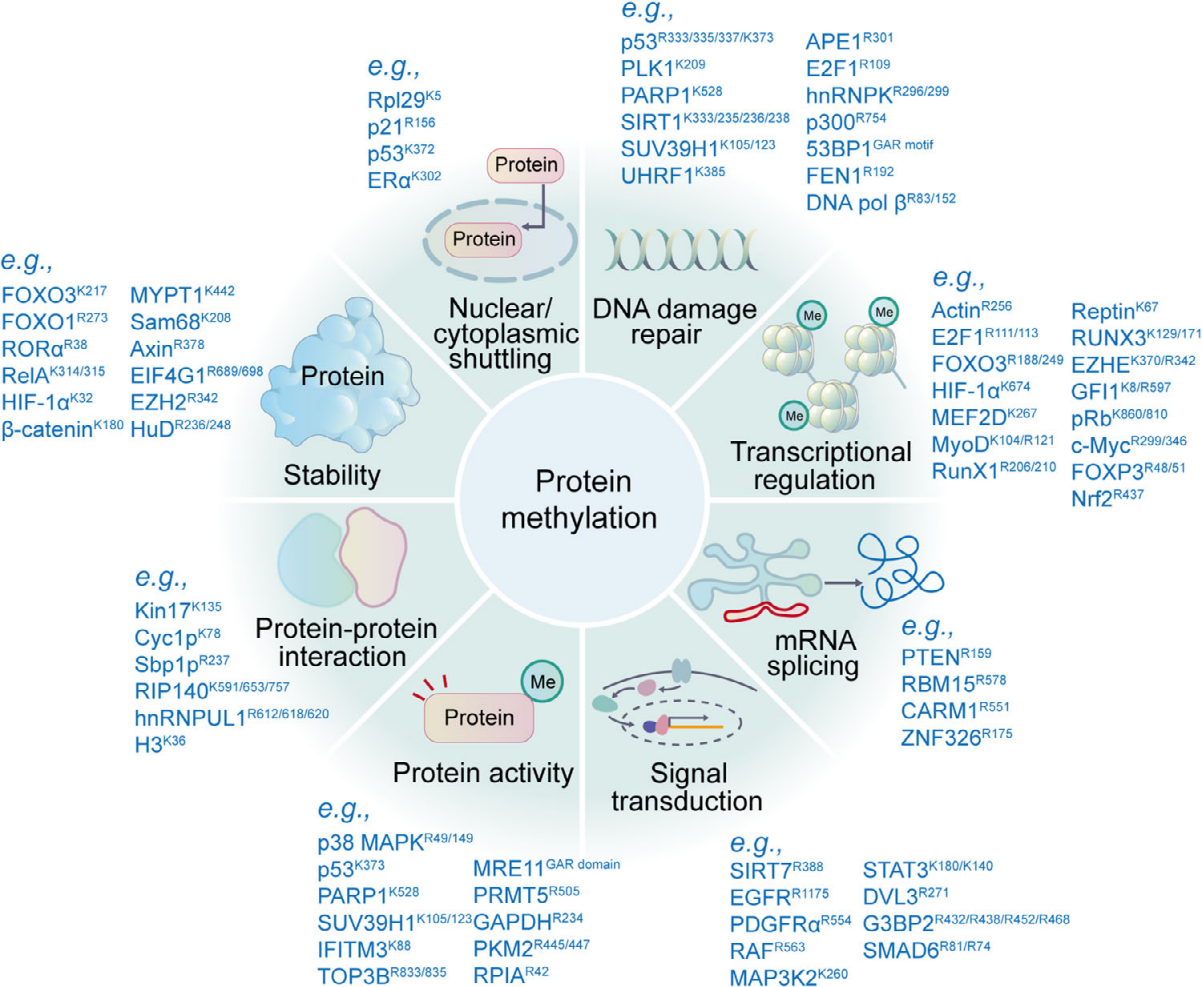
Functions of protein methylation. Representative methylation substrates are presented. Protein methylation is extensively involved in signal transduction, protein stability, protein activity, protein–protein interaction, mRNA splicing, transcriptional regulation, DNA damage repair, nuclear, and cytoplasmic shuttling.

Methylation is involved in the regulation of protein stability, protein activity, PPIs, nuclear‐cytoplasmic shuttling, DNA damage repair, transcriptional regulation, ribosome assembly, RNA processing and trafficking, heterogeneous RNA ribosomal protein maturation, protein translation and processing, and intracellular signal transduction (Figure 14).^27^, ^740^ Normal methylation modification is of great significance to the growth and development of cells and organisms. For example, EHMT2, also known as G9A, has a SET domain and acts as a transcriptional cooperator or a corepressor.^741^, ^742^ EHMT2 can catalyze the mono‐ or di‐methylation of H3K9, which is involved in the regulation of embryonic development and DNA replication.^743^ The *Ehmt2^−/−^* SET domain deletion mutation is embryonic lethal.^744^ G9a‐mediated nonhistone MyoD methylation plays a key regulatory role during muscle development. G9a methylates MyoD at Lys104 to limit its transcriptional activity. Mutation of Lys104 makes MyoD activity refractory to G9a transferase inhibition, resulting in enhanced myogenic activity.^745^

### 6.1 Methylation in aging

Histone methylation and methylated proteins have recently been shown to play a role in regulating lifespan and tissue aging in organisms. EZH2 is involved in the regulation of aging. Compared with young mice, more EZH2 was recruited to the SDF1 promoter region in aged mice to inhibit SDF1 expression and promote skin tissue regeneration and repair in aged mice.^746^ KDM2B is a regulator of mouse embryonic fibroblast (MEF) lifespan.^747^ KDM2B inhibits MEF senescence through demethylation of H3K36me2, resulting in cell immortalization (Table 6).^748^

**TABLE 6:** Representative methylation events in health and diseases.

Diseases and biological processes		Substrates	Effects
Aging		H3K27	Pharmacologic inhibition of EZH2 restores SDF1 induction and prevents tissue regeneration.^746^
		H3K36	KDM2B inhibits MEF senescence by demethylating H3K36me2.^748^
Metabolic disorders	Metabolic syndrome	SHP	PRMT5 catalyzes Arg57 methylation of SHP to augments the SHP repression function and mitigates the risk of metabolic syndrome.^790^
	Vascular dysfunction in T2DM	H3K4	In endothelial cells, high glucose induced sustained expression of NF‐κB p65 subunit and inflammatory genes by increasing H3K4me1 via SETD7 activation.^764^
	Diabetes	H3K4, H3K9	Hyperglycemia induces aberrant changes in H3K4me2 and H3K9me2 in human monocytes.^791^
	Obesity	H3K36	Nsd2‐mediated H3K36 methylation affects adipose tissue development and function.^792^
	DN	H4K20	H4K20 methylation is a direct target of KMT5A. KMT5A and RFX1 modulate ENO1, and are involved in hyperglycemia‐mediated EndMT in glomeruli of DN.^793^
Immune diseases	Autoimmunity	FOXP3	FOXP3 is dimethylated by PRMT5 (or PRMT1, PRMT6) at R48 and R51, which attenuates the expression of immunosuppressive genes and may lead to autoimmunity.^27^
	Secondary bacterial infection	H3K9	Setdb2 is upregulated and induces the repressive H3K9me3 of at Cxcl1 promoter, leading to reduced neutrophil infiltration and attenuated host defense against secondary bacterial infection.^794^
	Innate antiviral immunity	TBK1	PRMT1 interacts with TBK1 and catalyzes asymmetric methylation of R54, R134, and R228 on TBK1. Myeloid‐specific Prmt1‐knockout mice are more susceptible to infection with DNA and RNA viruses than Prmt1^fl/fl^ mice.^795^
Neurodegenerative diseases	HD	H2A, H4	Mutant HTT inhibits the activity of PRMT5 and reduces symmetrical dimethylation of H2A and H4 in HD brain.^775^
		H3K27	Reduced PRC2 in adult neurons inhibits the expression of PRC2 target genes. Loss of neuronal function and survival enhances the ongoing dysregulation of PRC2, as well as other H3K27me3‐regulated enzymes, likely leading to systemic neurodegeneration in HD.^796^
	AD	H3K4	KMT2A can monomethylate and trimethylate H3K4 to promote neuronal gene expression. Mice heterozygous for loss‐of‐function mutations in the *KMT2A* gene exhibit learning and memory deficits.^771^
		H3K9	EHMT1/2 inhibitors can reverse histone hyper‐methylation and lead to the recovery of glutamate receptor expression and excitatory synaptic function in prefrontal cortex and hippocampus in FAD mice.^797^
	Neurodevelopmental disorders	H4K20	Demethylation of H4K20me1 by Phf8 results in transcriptional suppression of RSK1 and homeostasis of mTOR signaling and causes cognitive impairments.^774^
	PD	H3K9	α‐Synuclein overexpression enhances H3K9me2 level in SNAP25 promoter region by ΕΗΜΤ2 to affect α‐synuclein‐regulated synaptic vesicle fusion events and leads to synaptic dysfunction in PD.^776^
		H3K4	Increase of H3K4me3 at the SNCA promoter reverts the deregulated expression of α‐synuclein in neurons in the context of PD.^798^
		H3K4	Upregulating H3K4me3 by GSK‐J4 confers neuroprotection from oxidative stress and alleviate motor deficits in PD.^799^
	ALS	H3K9, H3K27, H3K79, H4K20	Reduced mRNA levels from pathogenic C9orf72 are associated with enhanced binding of trimethylated lysine residues in histones H3 and H4.^800^
CVDs	Coronary and ventricular defects	H3K36	SETD2 deletion reduces H3K36me3 and affects the expression of cardiac development‐related genes Rspo3 and Flrt2, resulting in coronary and ventricular defects.^749^
	Cardiac malformations	H3K4	H3K4 methyltransferases, SETD7 and SMYD3, are highly expressed during the development of zebrafish heart. Knockout or overexpression of both Setd7 and Smyd3 can induce cardiac malformations.^752^
	Diabetic vascular complications	H3K4, H3K9	H3K4me1, H3K9me2, and H3K9me3 promote endothelial dysfunction in diabetic vasculature by inducing ROS.^801^
	CAD	H3K4	ANRIL can promote the combination of WDR5 and HDAC3 complexes and active histone marks such as H3K4me3 to upregulate the ROS level and promote the transformation of HASMC phenotype^802^
	Cardiac hypertrophy	H3K9	G9a mediates cardiomyocyte homeostasis by repressing antihypertrophic genes through H3K9 methylation and interaction with EZH2 and MEF2C.^803^
		H4R3	PRMT5 ameliorates cardiomyocyte hypertrophy and induces the methylation of H4R3me2 via the transcriptional activation of Filip1L and subsequent enhancement of β‐catenin degradation.^804^
Cancers	Breast cancer	EZH2	EZH2 R342 methylation by PRMT1 increases EMT of breast cancer cells.^780^
		H3K9	G9a exerts its oncogenic function in breast cancer by repressing hephaestin and destruction cellular iron homeostasis.^805^
	PT	H3K36	Alteration of SETD2 and downstream H3K36me3 may be involved in the development of PT.^806^
	Neuroblastoma	H4R3	Downregulation of PRMT1 in neuroblastoma leads to decreased expression of H4R3me2a enrichment at *ATF5* promoter and inhibits tumor cell growth.^781^
	ccRCC	H4R3	DCPT1061 inhibits ccRCC cell proliferation and induces G1 phase arrest by decreasing the expression of ADMA and PRMT1‐mediated H4R3me2a.^782^
	HCC	H3K9	Knockdown of G9a reduces H3K9me2 and impairs HCC cell growth and sphere formation.^807^
	Leukemogenesis	H3K36	SETD2 mutations affect the expression of leukemigenic genes, hinder the repair of H3K36me3‐mediated DNA damage.^808^
	CRC	H3K36, H3K9	Overexpression of KDM4C reduces H3K36me3 and H3K9me3 at the promoter of MALAT1, thereby up‐regulating MALAT1 expression and enhancing β‐catenin signaling pathway.^809^
	PDAC	H3K36	SETD2 loss reduces H3K36me3 occupancy at Fbxw7, leading to decreased Fbxw7 expression and increased Myc protein.^810^
	LUAD	H3K36	SETD2 inhibits CXCL1 expression by promoting H3K36me3 within the promoter of CXCL1 to reduce the proliferation of LUAD cells and the growth of tumors.^811^

Abbreviations: AD, Alzheimer's disease; ALS, amyotrophic lateral sclerosis; CAD, coronary atherosclerotic heart disease; ccRCC, clear cell renal cell carcinoma; CRC, colorectal cancer; CVDs, cardiovascular diseases; DN, diabetic nephropathy; HCC, hepatocellular carcinoma; HD, Huntington's disease; LUAD, lung adenocarcinoma; PD, Parkinson's disease; PDAC, pancreatic ductal adenocarcinoma; PT, phyllodes tumor of the breast.

### 6.2 Methylation in heart development and CVDS

Abnormal protein methylation or mutations in methyltransferases often lead to many diseases, such as CVDs, metabolic diseases, immune diseases, neurodegenerative diseases, and tumors. Mutations in multiple SETD family members have been associated with abnormal development of the cardiovascular system, mainly involving SETD2,^749^ SETD5,^750^ and SETD7.^751^ For example, the loss of *Setd2* in cardiac progenitor cells leads to obvious coronary vascular defects and ventricular noncompaction, which causes the fetus to die in mid‐gestation. The mechanism may be that *Setd2* deletion significantly reduces the level of H3K36me3 and affects the expression of the heart development‐related genes *Rspo3* and *Flrt2*.^749^ Both SETD7 and SMYD3 are H3K4 methyltransferases that are highly expressed during heart development in zebrafish. Knockout and overexpression of both *Setd7* and *Smyd3* induce severe defects in cardiac morphogenesis, suggesting that SETD7 and SMYD3 have a synergistic effect on heart development.^752^ Moreover, abnormal expression of SETD family members is potentially related to pulmonary hypertension. SETD3 may be protective factors against hypoxic pulmonary hypertension,^753^ while SETD2, SETD8, and SETD9 may be pathogenic factors in hypoxic pulmonary hypertension or pulmonary fibrosis.^754^, ^755^, ^756^ In human and mouse hypertrophic hearts, the expression of JMJD1C is increased, and the methylation level of H3K9 is decreased. Knockdown of *Jmjd1c* can inhibit Ang II‐induced expression of hypertrophy‐related genes and cardiomyocyte hypertrophy, while overexpression of JMJD1C can promote cardiomyocyte hypertrophy. Elevated JMJD1C expression induced by pathological conditions reduces the levels of H3K9me1/2/3 at the CaMKK2 promoter, which was associated with CaMKK2 gene silence. CAMKK2 could facilitate the development of metabolic dysfunction and cardiac hypertrophy.^757^

### 6.3 Methylation in metabolic disorders

Histone methylation may be responsible for diabetic complications, including diabetic neuropathy, and the phenomenon of “metabolic memory” of long‐term changes, and plays a crucial role in pathways of fibrosis, inflammation, and oxidative stress.^758^ PRMT1 is associated with abnormal glucose tolerance by affecting hepatic glucose metabolism and insulin secretion. PRMT1 knockdown reduced the activation of insulin signaling and inhibited the expression of gluconeogenic genes in hepatocytes.^759^ High expression of SHP can inhibit the activity of some metabolic enzymes, which increases glucose tolerance and reduces the levels of bile acid and triglycerides. PRMT5 catalyzes Arg57 methylation of SHP to augments the SHP repression function and reduce the occurrence of metabolic syndrome.^760^ Increased expression of SETD7 is one of the mechanisms of vascular dysfunction in T2DM.^761^, ^762^ In endothelial cells, high glucose induced sustained expression of NF‐κB p65 subunit and inflammatory genes by increasing H3K4me1 via SETD7 activation.^763^, ^764^ Moreover, high‐glucose stimulation can reduce the level of H3K9me3 and increase the expression of inflammatory genes in normal human vascular smooth muscle cells (VSMCs).^758^ On the contrary, SETD8 is a protective factor against endothelial damage in hyperglycemic patients (Table 6).^765^

### 6.4 Methylation in immune diseases

PRMTs play a critical role in the establishment and maintenance of lymphoid and myeloid cell lines. PRMT1 is essential for lymphocyte development, proliferation, and differentiation in vivo, as well as for cytokine production by Th cells. CARM1 regulates the differentiation of early thymocyte progenitors by methylating the T cell‐specific factor TARPP at R650, while PRMT5‐mediated arginine methylation is crucial for the recruitment of TFs during cytokine gene expression in activated T cells. PRMTs have a role in regulating inflammation, with PRMT1 acting as a negative regulator. It interacts with and methylates the NF‐κB subunit, RelA/p65, at R30 to suppress its activation by TNF‐α. Asymmetric dimethylation of RelA/p65 at R30 inhibits its function as a TF.^766^ On the other hand, PRMT5 is a positive regulator of inflammation, as it contributes to the activation of IKK and NF‐κB, and the induction of several NF‐κB target genes.^767^ Additionally, PRMT6 and CARM1 also positively regulate inflammation.^768^

PRMT1 is involved in both acute and chronic asthma in epithelial cells and fibroblasts.^768^ The PRMT5 inhibitor C220 can reduce T cell proliferation and cytokine production, thereby alleviating acute graft‐versus‐host disease.^769^ Moreover, selectively inhibiting PRMT5 may prove to be an effective therapeutic strategy for RA and ulcerative colitis.^768^ PRMT7 is an essential contributor to B cell lymphomagenesis.^768^ FOXP3, a TF critical for Treg cell identity and immunosuppressive function, is dimethylated by PRMT5 (or PRMT1, PRMT6) at R48 and R51. This methylation reduces the expression of immunosuppressive genes, consequently leading to autoimmunity, tumor shrinkage, and associated CD8^+^ T cell infiltration.^27^ Anti‐hnRNP reactivity in RA, systemic lupus erythematosus (SLE), and mixed connective tissue diseases (MCTD) is mainly derived from arginine‐methylated proteins such as hnRNP A1, A2, and K (Table 6).^770^

### 6.5 Methylation in neurogenerative diseases

Protein methylation is closely related to neurological diseases.^771^ PRMT5 is highly expressed in mammalian neurons. In neurons, β‐amyloid peptide (Aβ) deposition reduces PRMT5 expression, increasing E2F‐1 expression and activating GSK‐3β and NF‐κB, leading to caspase‐3‐dependent neuronal apoptosis.^772^ KMT2A can monomethylate and trimethylate H3K4 to promote neuronal gene expression. Mice heterozygous for loss‐of‐function mutations in the *KMT2A* gene exhibit learning and memory deficits.^771^ The histone demethylase KDM2B is a candidate gene associated with intellectual disability, autism, epilepsy, and craniofacial abnormalities.^773^ KDM7B is a key factor in learning and memory. *KDM7B*‐knockout mice show impaired learning and memory, accompanied by abnormal long‐term potentiation in the hippocampus.^774^

The abnormal protein interactions of mutant HTT have been implicated in the pathogenesis of HD. Normal HTT stimulates PRMT5 activity in vitro. However, the presence of mutant HTT reduced the symmetrical dimethylation of arginine (sDMA) of histones H2A and H4 in primary cultured neurons and in HD brain, consistent with impaired gene transcription and RNA splicing in HD.^775^ In terms of PD, increased levels of α‐synuclein can boost the H3K9 methylation activity of EHMT2, which may affect SNARE complex assembly and effectively vesicle fusion events.^776^ Methyltransferase KMT2A (MLL1) and G9a are associated with AD. KMT2A plays a protective role in AD, while G9a plays a harmful role. Inhibition of the G9a/GLP complex promotes long term potentiation and synaptic tagging/capture in the hippocampus (Table 6).^771^

### 6.6 Methylation in cancers

Aberrant methylation is closely associated with the occurrence and development of cancer.^734^ Compared with normal tissues, PRMT1, PRMT4, and PRMT6 showed higher expression in lung cancer tissues.^777^, ^778^ Similarly, PRMT1, PRMT2, PRMT3, PRMT4, and PRMT7 are highly expressed in breast cancer tissues.^779^ EZH2 R342 can be methylated by PRMT1, which increases epithelial–mesenchymal transition (EMT) in breast cancer cells and predicts poor prognosis in breast cancer patients.^780^ Downregulation of PRMT1 expression in neuroblastoma results in reduced activity of the prosurvival factor ATF5 and inhibits tumor cell growth.^781^ In clear cell RCC (ccRCC), a novel potent inhibitor, DCPT1061, was found to induce G1 cell cycle arrest by targeting PRMT1 activity.^782^ G9a is highly expressed in diverse tumors and indicates poor prognosis. G9a can induce H3K9me2 to affect cancer cell growth and apoptosis.^783^ Set7‐mediated methylation of Gli3 at K436 and K595 in the Sonic Hedgehog pathway promotes NCSLC.^784^ Aberrant SMYD3 expression may contribute to carcinogenesis.^785^, ^786^, ^787^ In PDAC, SMYD3‐catalyzed MAP3K2 methylation at lysine 260 is involved in the regulation of oncogenic Ras signaling.^788^ SMYD3 also exhibits a proto‐oncogenic role in prostate cancer due to its methyltransferase enzymatic activity.^789^ Moreover, key methylation sites, such as H3K27me3, have also been found to be upregulated in many cancers including prostate cancer, breast cancer, and lymphoma, indicating their involvement in tumor progression (Table 6).^728^

### 6.7 Methylation‐associated targeted therapies

EZH2, a key histone methyltransferase and EMT inducer, is overexpressed in diverse carcinomas. Given its role in tumorigenesis and progression, EZH2 has emerged as a potential antitumor therapeutic target.^812^, ^813^ It has been reported that EZH2 can enhance adhesion turnover and accelerate tumorigenesis by increasing cytoskeletal regulatory protein, Talin1 methylation and cleavage. However, this capacity is abolished by targeted disruption of the EZH2 interaction with cytoskeleton remodeling effector, VAV. The interaction of EZH2 with VAV family proteins in the cytoplasm contributes to initial tumor transformation and may maintain cancer stem cells by regulating adhesion dynamics and STAT3 signaling pathways.^814^ Some anticancer drugs targeting mutant or wild‐type EZH2, such as GSK126, were used to inhibit EZH2‐mutant lymphoma cells,^815^ and EPZ‐6438 in a phase I/II clinical trial was designed for treating patients with relapsed or refractory B‐cell non‐Hodgkin lymphoma or advanced solid tumors.^812^ Many G9a inhibitors, such as diazepinquinazolin‐amines and benzimidazoles, have also been developed. The current G9a inhibitors are roughly classified into three types according to their binding modes, including substrate competitive inhibitors, SAM cofactor competitive inhibitors, and inhibitors whose mechanism of inhibition remains elusive. In general, substrate‐competitive inhibitors show better selectivity for G9a than SAM inhibitors.^783^ In addition, the PRMT1 inhibitor GSK3368715 has also entered phase I clinical trials,^816^ and other inhibitors, including AMI‐1, allantodapsone and furamidine, have also started preclinical studies.^817^

## 7 UBIQUITINATION

**FIGURE 15:**
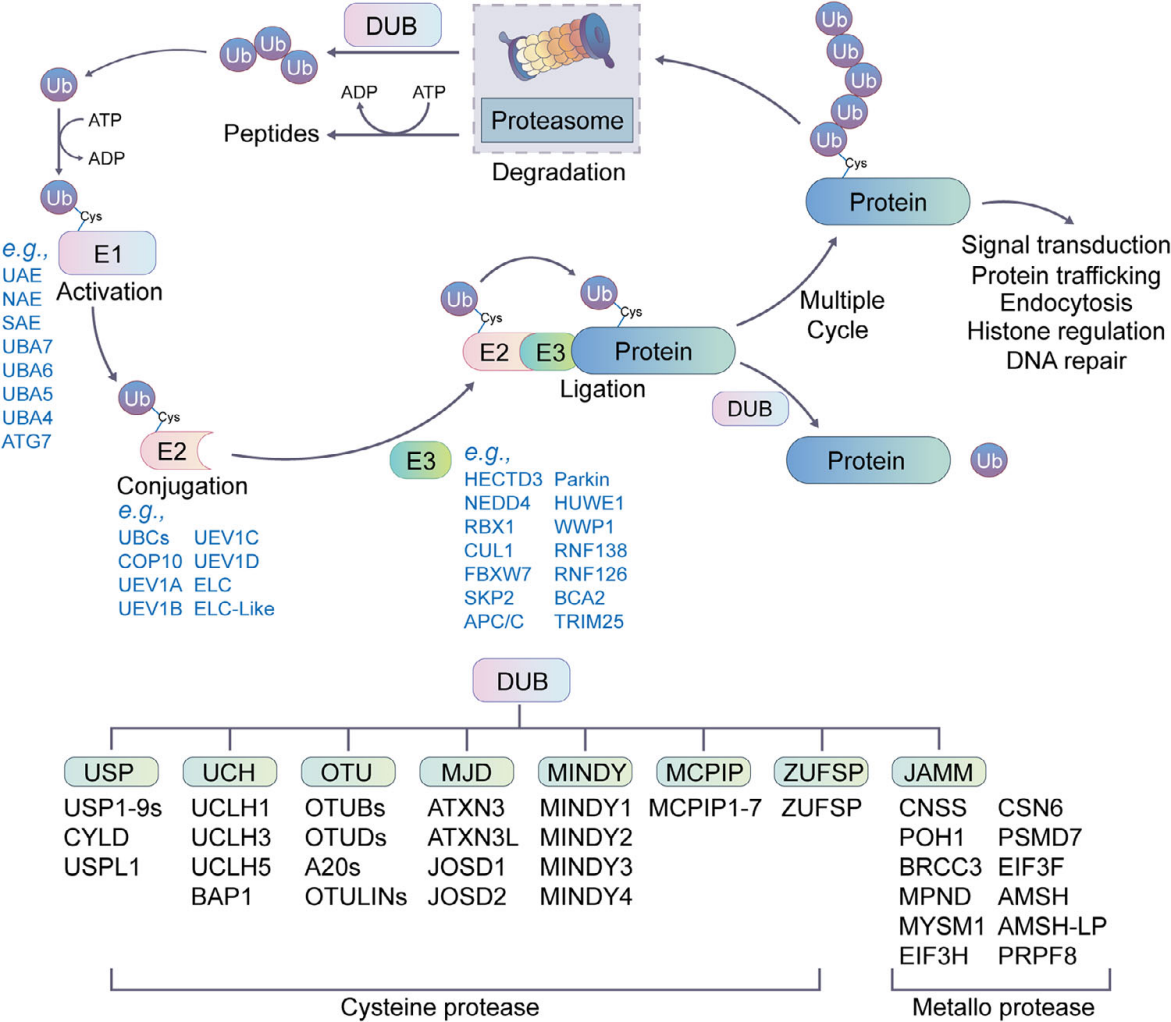
The protein ubiquitination pathway. The ubiquitin (Ub) moiety is activated by E1 through the cysteine (Cys) residue of E1. Ub at E1 is transferred to the Cys residue of E2. Ub conjugated with E2 is transferred to the lysine residue of a substrate protein by E3. Ubiquitinated proteins with ubiquitination are subjected to 26S proteasome‐dependent degradation or execute other functions and activities. Deubiquitination is the opposite mechanism of ubiquitination mediated by DUBs. The process includes reversing ubiquitin conjugation and recycling ubiquitin molecules through the UPS. Based on the enzymatic cleavage mechanism, the DUB family is divided into two subfamilies. The cysteine protease family consists of USP, UCH, OTH, MJD, MINDY, MCPIP, and ZUFSP. Metalloprotease family includes JAMM.

Ubiquitination (also termed ubiquitylation) is the covalent attachment of Ub monomers or Ub chains to lysine residues of proteins. In addition to lysines, the side chains of serine, threonine and cysteine can also undergo ubiquitination.^818^ Ub, a protein of 76 amino acids that is highly conserved in eukaryotes, contains seven lysine residues K6, K11, K27, K29, K33, K48, and K63 through which the ubiquitination chain extends.^819^ Protein modification can occur through monoubiquitination (a single Ub moiety) or polyubiquitination (Ub chains) via the isopeptide linkage between two Ub moieties.^820^ Monoubiquitination refers to the modification of a target protein by a single Ub molecule, while multimonoubiquitination involves the simultaneous modification of multiple lysine residues of a target protein by a single Ub molecule. If a single lysine residue of the target protein is labeled by Ub chains, polyubiquitination occurs.^821^ The N‐terminal methionine residue (Met1) can also be modified by Ub molecules, further increasing the diversity and complexity of ubiquitination.^822^ Ubiquitination is regulated by three enzymes: Ub‐activating enzyme (E1), Ub‐conjugating enzyme (E2), and Ub‐ligase enzyme (E3).^823^ First, E1 (UBA1, UBA6) forms a high‐energy thioester bond with the Ub molecule to activate it. Then, the activated Ub is covalently attached to the cysteine residue of E2 (UBE2B, UBE2D2) through a thioesterification reaction. Finally, activated Ub is either directly attached to substrates through E2 or transferred to substrates in the presence of E3 (RNF6, TRAF6, SKP2, and Nedd4).^824^ Ubiquitination is a reversible process. The removal of ubiquitination is mainly carried out by deubiquitinases (DUBs). Both Ub‐modifying enzymes and deubiquitinating enzymes work together to regulate the transmission of intracellular Ub signaling to maintain normal cellular activities (Figure 15).^825^

Thus far, only two human E1 Ub‐activating enzymes, UBA1 and UBA6, have been identified.^826^ However, in the human proteome, eight E1 enzymes are known to activate Ub‐like proteins (UBLs), including UBA1 (UAE), NAE, SAE, UBA6, UBA7, UBA4, UBA5, and ATG 7.^827^ In contrast, more than 40 human E2 Ub‐conjugating enzymes have been reported.^828^ There are more than 600 E3 Ub ligases, which can be roughly divided into three categories: RING (truly interesting new gene) E3 ligases, HECT (homologous to E6AP C‐terminus) E3 ligases, and RBR (RING‐between‐RING) E3 ligases.^829^ Ring E3 ligases are the most predominant Ub ligases in the human body.^830^ There are few identified DUBs, and more than 100 DUBs have been identified to date.^818^ Based on sequence homology, deubiquitinating enzymes can be divided into eight classes (Figure 15), including Ub C‐terminal hydrolases (UCHs), Ub‐specific proteases (USPs), Machado‐Joseph domain‐containing proteases (MJDs), ovarian tumor proteases (OTUs), motif interacting with Ub‐containing novel DUB family (MINDYs), JAMMs (JAB1/MPN/MOV34), monocyte chemotactic protein‐induced protein (MCPIP) families, and ZUFSP DUB family.^831^ According to the mechanisms of action, deubiquitinating enzymes can also be divided into cysteine proteases (including UCHs, USPs, MJDs, OTUs, and MINDYs and MCPIPs) and metalloprotease JAMMs.^832^

**FIGURE 16:**
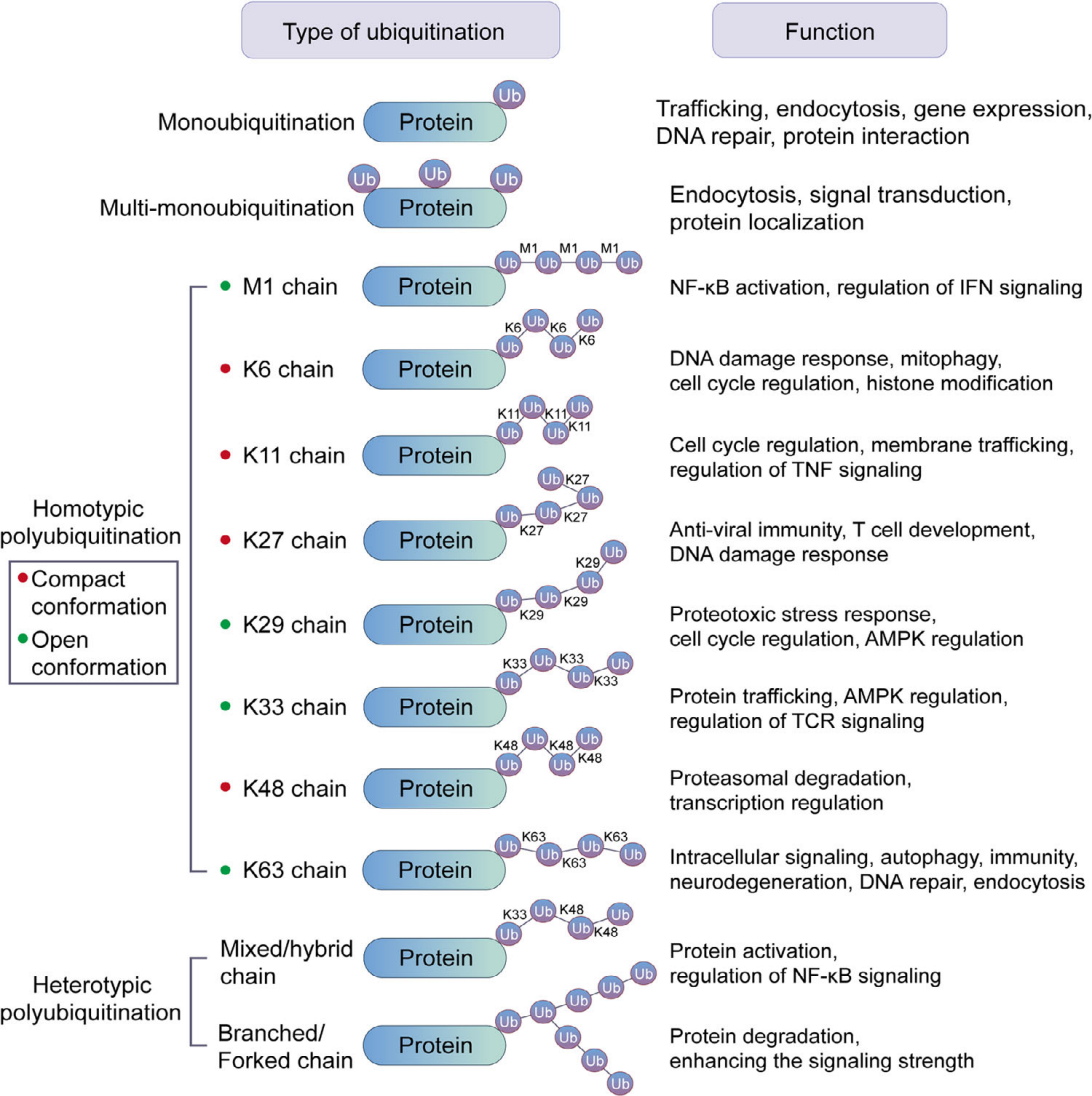
Ubiquitin linkage types and their roles. Ubiquitination can occur as single or multiple monoubiquitin or as homotypic/heterotypic/branched chains linked through K6, K11, K27, K29, K33, K48, or K63, as well as M1. The functional consequences of ubiquitin signals are determined by cellular and substrate‐context, ubiquitin chain position, linkage type and conformation, ranging from proteasomal degradation to nonproteolytic functions.

A variety of Ub combinations form a variety of structures and are involved in different physiological functions.^833^, ^834^, ^835^ Among the seven types of polyubiquitin chains, the most common K48 and K63 polyubiquitin chains mainly regulate proteasomal degradation of substrates and intracellular signaling, respectively.^836^, ^837^ K48‐linked ubiquitination is the most prevalent signal for proteasomal degradation, although other ubiquitination types such as K11‐ or K29‐linked ubiquitination and multiple monoubiquitination are also signals for proteasomal degradation.^838^, ^839^ In contrast, K63‐linked ubiquitination regulates “proteasome‐independent” processes such as inflammatory signal transduction, neurodegeneration, DNA repair, endocytosis, and selective autophagy.^840^, ^841^ K27 is critical for cellular immunity^842^ and the DNA damage response.^843^ The M1 chain (linear chain) is a positive regulator of NF‐κB signaling^844^ and a negative regulator of type I IFN signaling.^845^ The K6 chain is involved in the regulation of the UV‐induced DNA damage response and mitochondrial homeostasis.^846^, ^847^ The K11 chain is another proteasomal degradation signal involved in cell cycle regulation.^848^ K11‐linkages are also implicated in regulating membrane trafficking and the innate immune response.^845^ The K29 chain is involved in proteotoxic stress responses, cell cycle and AMPK regulation.^845^, ^849^ The K33 chain acts on protein exchange at the Golgi membrane and is related to the regulation of the innate immune response.^850^ However, polyubiquitin chains can be heterogeneous, consisting of more than one type of connection, and are divided into branched/forked chains and mixed (hybrid) chains.^837^, ^851^ A large proportion (10–20%) of branched chains are present in the aggregated form of ubiquitination.^852^ Branched Ub chains have two degradative linkages, including K11/K48 or K29/K48. The K48/K63 branched Ub chain can enhance NF‐κB signaling.^837^, ^853^ Similarly, mixed chains consisting of two NF‐κB‐associated junctions (M1 and K63) are formed during NF‐κB activation.^851^ The discovery that the K63 chain is modified by the M1 chain in a hybrid or branched structure addresses the question of whether the K63 chain acts on inflammatory signaling or NF‐κB activation.^851^ In contrast to polyubiquitination, monoubiquitination plays critical roles in DNA repair, receptor endocytosis, vesicle sorting, and gene silencing,^854^ and multimonoubiquitination is involved in the regulation of receptor endocytosis, protein interactions and localization (Figure 16).^855^

### 7.1 Ubiquitination in development

An increasing number of studies have shown that members of various E2 and E3 families are involved in sperm capacitation, oocyte maturation, and embryonic development in mammals.^856^ For example, the absence of UCH‐L1, an neuronal deubiquitinating enzyme, impacts the maintenance of spermatogonial stem cells homeostasis and metabolism and impacts the differentiation competence.^857^ Testicular macrophage USP2 promotes sperm motility, activation, and capacitation.^858^ Polycomb repressive complex 1 (PRC1) is known to play a crucial role in stem cell and tissue development. It has been demonstrated that PRC1, in conjunction with H2AK119ub, influences early embryonic development. High levels of expression of Polycomb repressive DUB complex (PR–DUB) in zygotes can rapidly reduce H2AK119ub levels, resulting in developmental arrest at the 4‐cell stage.^859^ Furthermore, Cbls play a role in promoting the ubiquitination and degradation of FLT3, which results in the inhibition of FLT3 signaling and limits the development of CD8α^+^/CD103^+^ DC1 (cDC1). When Cbls are absent, activated FLT3 cannot be efficiently removed, leading to constant FLT3 signaling that favors cDC1 development and expansion.^860^

### 7.2 Ubiquitination in aging

During aging, there is a buildup of damaged and aggregated proteins, which can lead to a decline in cellular function. Ub‐dependent proteolytic pathways are critical for the efficient turnover of defective proteins.^861^ However, age‐related impairment of these pathways can lead to a greater accumulation of damaged proteins, thereby exacerbating the aging process.^861^, ^862^ For example, in aged *C. elegans*, 192 proteins with low levels of ubiquitination accumulate, further contributing to the decline in cellular function.^863^ Notably, ageing causes a global loss of ubiquitination that is triggered by increased DUB activity. Parkin‐mediated mitophagy is essential to ensure mitochondrial quality control in myocardium. The main mechanism of action is that the interaction between Parkin and TBKI promotes the K63 polyubiquitination of TBK1, which in turn promotes TBK1 phosphorylation to enhance mitophagy and alleviate cardiac aging.^864^ However, excessive or inappropriate protein ubiquitination may also shorten longevity. For example, the Ub ligase RLE‐1 selectively polyubiquitinates daf‐16, a key component in the insulin/IGF signaling pathway, leading to its degradation by the proteasome. As a result, inhibition of RLE‐1 extends lifespan in *C. elegans*.^865^ In human fibroblasts, the degradation of BMAL1 is mediated by the E3 Ub ligase STUB1. Reduced BMAL1 can attenuate cellular senescence induced by hydrogen peroxide.^866^ Nrf2 is an important regulator in healthy aging, and its activity is also affected by ubiquitination. For example, p62 prevents Nrf2 from being ubiquitinated by combining with Keap1. Some studies have found that the expression of p62 decreases with age. Hrd1 is a negative regulator of Nrf2. It interacts with Nrf2 through its Neh4–5 domain to enhance its ubiquitination.^867^, ^868^ Stem cell dysfunction and reduced regenerative capacity are hallmarks of aging. NANOG, one of homeobox proteins, plays a crucial role in regulating self‐renewal and pluripotency for embryonic stem cells (ESCs).The deubiquitinating enzyme USP21 increases NANOG levels to maintain pluripotency in ESCs by deubiquitinating NANOG to reduce NANOG proteasomal degradation (Table 7).^869^

**TABLE 7:** Representative ubiquitination events in health and diseases.

Diseases and biological processes		Protein substrates	Effects
Aging		TBK1	K63 polyubiquitination of TBK1 promotes TBK1 phosphorylation to enhance mitophagy and attenuate cardiac aging.^864^
		daf‐16	The Ub ligase RLE‐1 selectively poly‐ubiquitinates daf‐16 and promotes proteasomal degradation. Inhibition of RLE‐1 prolongs lifespan in *C. elegans*.^865^
		BMAL1	STUB1 ubiquitinates and degrades the substrate BMAL1, attenuating hydrogen peroxide‐induced cellular senescence.^938^
Immune regulation	Viral infection	IRF7	NEURL3 promotes innate antiviral responses by catalyzing K63‐linked poly‐ubiquitination of IRF7 at K375.^872^
	Viral infection	RIG‐I	The CoV nucleocapsid (N) protein of SADS‐CoV interacts with RIG‐I and promotes its K27‐, K48‐, and K63‐linked ubiquitination to induce RIG‐I degradation, which further inhibits the host IFN‐β response.^879^
	Viral infection	RIG‐I	lncRNAs can promote influenza A virus (IAV) replication and immune evasion by restricting RIG‐I K63 ubiquitination mediated by TRIM25.^880^
	Viral infection	PPE	Mycobacterial PPE protein ubiquitination mediated by MKRN1 suppresses the innate immune response.^881^
	Viral infection	MAVS	Viral infection enhances the interaction between USP18 and MAVS and promotes the K63‐linked ubiquitination of MAVS to upregulate the production of IFN‐I.^882^ RNF115 interacts with MAVS to promote K48 ubiquitination of MAVS, and loss of RNF115 enhances antiviral signaling triggered by RNA viruses.^883^
	Autoimmune disease	c‐Rel	Peli1 negatively regulates T cell activation and inhibit the development of autoimmunity through K48 ubiquitination dependent degradation of c‐Rel.^875^
Metabolic disorders	Insulin resistance	MG53	MG53 acts as an E3 ligase targeting insulin receptor and IRS1 for Ub‐dependent degradation. Overexpression of MG53 is sufficient to induce muscle insulin resistance and metabolic syndrome.^887^
	Diabetes	EZH2	Suppressive role of E3 Ub ligase FBW7 in type I diabetes in nonobese diabetic mice through mediation of ubiquitination of EZH2.^886^
Cancers	Multiple cancers	p53	The tumor suppressor p53 is degraded by ubiquitination mediated by MDM2, resulting in immortal cancer cell proliferation.^895^
	Liver cancer	Smad4	USP10 stabilizes Smad4 by ubiquitinating it, activates TGF‐β signaling, and promotes liver cancer metastasis.^900^
	PC	TRAF6	USP4 is highly expressed in PC. It stabilizes TRAF6 and activates the NF‐κB signaling pathway to enhance the proliferation, migration and invasion of PC cells.^902^
	Ovarian cancer	BCL6	USP14 expression is increased in cisplatin‐resistant ovarian cancer cells. It inhibits ovarian cancer cell apoptosis by stabilizing BCL6, which increases ovarian cancer cisplatin resistance.^904^
	PDAC	DRYK1A	USP22 is highly expressed in PDAC. It enhances the growth and colony formation ability of cancer cells by regulating DRYK1A.^905^
	Gastric cancer	SMAD2	USP32 is highly expressed in gastric cancer and is closely related to the stage and prognosis of gastric cancer patients. Downregulation of USP32 significantly reduces SMAD2 expression, thereby inhibiting the proliferation, migration, and resistance to cisplatin of gastric cancer cells.^906^
	Liver cancer	TBLR1	USP1 promotes the survival of liver circulating tumor cells in the bloodstream by deubiquitinating and stabilizing TBLR1.^907^
	Liver cancer	RAB1A	USP2a is highly expressed in HCC tissues and is positively correlated with poor prognosis. USP2a can deubiquitinate and stabilize RAB1A to promote HCC progression.^908^
	Multiple cancers	K‐Ras	Ubiquitination of K‐Ras can enhance its interaction with PI3K, leading to abnormal activation of the PI3K/AKT signaling pathway. This is one of the mechanisms by which the G12V mutation of K‐Ras causes malignant cell proliferation.^909^
CVDs	Cardiac fibrosis	RIP1	Peli1 silencing abrogates mechanical stretch‐induced polyubiquitination of TRAF6 and RIP1 and consequently decreases the DNA binding activity of NF‐κB in neonatal rat cardiac fibroblasts.^913^
	Vascular lesions	HIF	The loss‐of‐function mutation of *VHL* can inhibit the normal degradation of its downstream substrate HIF. Accumulated HIF activates downstream target genes such as *VEGF*, leading to the formation of vascular lesions.^939^
Neurodegenerative diseases	AD	C/EBPβ	Peli1 can directly ubiquitinate and degrade C/EBPβ, inhibits the phagocytosis of microglial cells, thus slowing down Aβ clearance in the brain of AD mice.^929^
	AD	C/EBPβ	Loss of COP1 results in rapid accumulation of the transcription factor C/EBPβ, which drives the expression of proinflammatory and neurodegeneration‐related genes and accelerates the neurodegeneration of AD.^928^
	AD	TAp73	Aβ42 can lead to hyperphosphorylation of Itch by abnormally activating the JNK signaling pathway. Hyperphosphorylated Itch ubiquitinates and degrades TAp73, leading to abnormal expression of important neuronal cyclins and causing neuronal apoptosis, which accelerates AD progression.^926^
	AD	ESR2	The HECT family protein E6AP can activate the transcription of ESR2, which reduces Aβ deposition in the hippocampus and improves learning and memory in AD rats.^927^
	PD	Synphilin 1	The UbcH7‐parkin complex promotes the ubiquitination and degradation of several proteins via the 26S proteasome. Cellular accumulation of the UbcH7‐parkin targets, α‐synuclein and synphilin‐1, has been associated with PD.^940^
	ALS	TDP‐43	The occurrence of ALS is related to neuronal cell death caused by the abnormal aggregation of highly phosphorylated and ubiquitinated pathological TDP‐43.^941^ Insufficient degradation of abnormally aggregated TDP‐43 protein leads to cell death and inflammation, which is one of the important mechanisms in the pathogenesis of ALS.^935^

Abbreviations: AD, Alzheimer's disease; ALS, amyotrophic lateral sclerosis; PC, pancreatic cancer; PDAC, pancreatic ductal adenocarcinoma; PD, Parkinson's disease.

### 7.3 Ubiquitination in immune regulation

Ubiquitination modulates the function of immune cells by regulating biological processes such as protein degradation and signal pathway transduction.^870^ K63‐ or K48‐linked ubiquitination is common in these processes.^871^ For example, the E3 Ub ligase NEURL3 promotes host antiviral immune response by catalyzing K63‐linked polyubiquitination of IRF7 at K375.^872^ The removal of K63 ubiquitination of TBK1 by USP15 negatively regulates TBK1 activity to suppress macrophage antiviral innate immune responses.^873^ Pellino 1 (Peli1) can mediate TLR‐stimulated K63 ubiquitination of c‐IAP2 in microglia to trigger the Ub ligase activity of c‐IAP2, which catalyzes the K48 ubiquitination and degradation of TRAF3 and consequently induces proinflammatory cytokine production and the recruitment of autoimmune T cells in peripheral lymphoid organs to the CNS.^874^ Peli1 also negatively regulates T‐cell activation and inhibits the development of autoimmunity through K48 ubiquitination‐dependent degradation of c‐Rel.^875^ The unanchored K48‐polyubiquitin chain synthesized by the E3 ligase TRIM6 can promote the binding of DHX16 to RIG‐I and mediate the production of IFN‐I and the expression of IFN‐stimulated genes (ISGs).^876^

During thymocyte development, multiple E3 ligases have been shown to play a role in T cell development. Protein ubiquitination regulates T cell development and differentiation. For example, Ub ligases, including Itch, LNX, DTX, Mib1, Mib2, Neur1, and Neur2, catalyze Notch ubiquitination, which is critical to the early stage of T cell development.^877^ Ubiquitination also affects the proliferation and development of B cells by regulating NF‐κB signaling and participating in BCR and BAFFR signal transduction. E3 Ub ligase Hrd1 mediates the downregulation of pre‐BCR through ubiquitination and promotes the maturation of pre‐B cells.^878^

Viruses can escape from immunity by degrading target proteins. For example, the CoV nucleocapsid (N) protein of SADS‐CoV interacts with RIG‐I and promotes its K27‐, K48‐, and K63‐linked ubiquitination to induce RIG‐I degradation, which further inhibits the host IFN‐β response.^879^ lncRNAs can promote influenza A virus (IAV) replication and immune evasion by restricting RIG‐I K63 ubiquitination mediated by TRIM25.^880^ Mycobacterial PPE proteins are ubiquitinated by MKRN1, which suppresses the innate immune response.^881^ Additionally, ubiquitination plays a role in regulating early innate immune responses triggered by human respiratory syncytial virus. RIG‐I, MAVS, TRAF3/6, and NEMO are the main proteins involved in these processes.^871^ Activation of MAVS is indispensable for antiviral immunity. Viral infection enhances the interaction between USP18 and MAVS and promotes the K63‐linked ubiquitination and subsequent aggregation of MAVS to upregulate the production of IFN‐I.^882^ RNF115 interacts with MAVS to promote K48 ubiquitination of MAVS, and loss of RNF115 enhances antiviral signaling triggered by RNA viruses (Table 7).^883^

### 7.4 Ubiquitination in metabolic disorders

Dysfunction of the Ub‐proteasomal system can lead to obesity‐related metabolic disorders such as diabetes and fatty liver. Chronic insulin stimulation inhibits hepatocyte ubiquitination by activating USP14, which also increases the nuclear translocation of the lipogenic TF SREBP‐1c to inhibit mature SREBP‐1c.^884^ USP7 is increased in diabetic foot ulcers and human umbilical vein endothelial cells (HUVECs). USP7 inhibition can suppress AGEs‐induced cell cycle arrest and cellular senescence in HUVECs by promoting p53 ubiquitination.^885^ E3 Ub ligase FBW7 prevents type I diabetes in nonobese diabetic mice by mediating EZH2 ubiquitination.^886^ MG53 acts as an E3 ligase targeting insulin receptor and IRS1 for Ub‐dependent degradation. Overexpression of MG53 is sufficient to induce muscle insulin resistance and metabolic syndrome.^887^ Diabetic cataract is also a common complication of diabetes. The E3 Ub ligase MDM2 may promote high glucose‐induced EMT and oxidative stress damage by downregulating LKB1. The EMT of lens epithelial cells is an important step in the development of diabetic cataracts.^888^ Pregnant women with obesity or gestational diabetes have reduced blood levels of adiponectin, which is thought to be associated with an increased risk of obesity or obesity‐related insulin resistance and fetal overgrowth. Adiponectin ubiquitination is increased in the visceral fat of obese pregnant women compared to their lean counterparts, and it is a key mechanism through which obesity curtails adiponectin secretion during pregnancy (Table 7).^889^

### 7.5 Ubiquitination in cancers

Dysregulation of ubiquitination may cause a range of adverse consequences, such as abnormal activation or inactivation of signaling pathways, abnormal protein complex formation, accumulation of misfolded proteins and mislocalization of proteins,^890^ and even cancers.^834^ Due to the specificity of E3 in recognizing protein substrates, E3 has received increasing attention.^891^ Some E3 ligases are carcinogenic factors, some are tumor suppressors, and some have both functions dependent on the context.^892^ E3 usually participates in tumorigenesis and development by regulating the stability of oncoproteins and tumor suppressors. Members of the Cbl family of E3 ligases are involved in tumorigenesis and development by mediating lysosomal sorting and degradation of activated RTKs.^893^ Mutations and aberrant expression of *C‐CBL* are most common in myelodysplastic syndromes.^894^ The tumor suppressor p53 is degraded by ubiquitination mediated by the E3 ligase MDM2, resulting in immortal cancer cell proliferation.^895^

DUBs also affect cancer signaling pathways by deubiquitination. The changes in DUBs may cause continuous activation or abnormal blockade of downstream signal transduction molecules such as PI3K/AKT^896^ and NF‐κB^897^ to affect the progression of malignant tumors. USP7 can deubiquitinate and stabilize MDM2 (Murine double minute 2) oncoproteins, which is the major negative regulator of the p53 tumor suppressor, thereby inducing the initiation, progression, and metastasis of human cancers.^898^ On the other hand, USP10 can counteract the effects of MDM2‐induced p53 nuclear export and degradation by deubiquitinating p53.^899^ Additionally, USP10 can also stabilize Smad4 by ubiquitinating it, which contributes to liver cancer metastasis.^900^ USP4 can interact directly with and deubiquitinates ARF‐BP1, leading to the stabilization of ARF‐BP1 and subsequent reduction of p53 levels.^901^ In addition, USP4 is highly expressed in PC tumors. It stabilizes TRAF6 and activates the NF‐κB signaling pathway to enhance the proliferation, migration and invasion of PC cells.^902^ In cervical cancer cell lines SiHa and Caski, silencing USP18 resulted in the inhibition of cell proliferation, induction of apoptosis, and promotion of cleaved caspase‐3 expression.^903^ USP14 has increased expression in cisplatin‐resistant ovarian cancer cells. It inhibits ovarian cancer cell apoptosis by stabilizing the level of BCL6, which increases ovarian cancer cisplatin resistance.^904^ USP22 is highly expressed in human PDAC tissues. It enhances the growth and colony formation ability of cancer cells by regulating the expression of DRYK1A.^905^ USP32 is highly expressed in gastric cancer and is closely related to the high T‐staging and poor prognosis of gastric cancer patients. Downregulation of USP32 can significantly inhibit the expression of SMAD2, thereby inhibiting the proliferation, migration, and chemoresistance to cisplatin of gastric cancer cells.^906^ TBLR1 plays an important role in regulating the Wnt signaling pathway. USP1 promotes the survival of liver circulating tumor cells in the bloodstream by deubiquitinating and stabilizing TBLR1.^907^ USP2a is highly expressed in HCC tissues and is positively correlated with poor prognosis. USP2a can deubiquitinate and stabilize RAB1A to promote HCC progression.^908^

In addition to impacting protein activity and degradation, ubiquitination is also implicated in cancer signaling regulation by modulating PPIs. Monoubiquitination of K‐Ras at K147 can lead to enhanced GTP loading and increases its affinity for specific downstream effectors PI3K and Raf, which results in anomalous activation of the PI3K–AKT signaling pathway. This is one of the mechanisms by which the G12V‐K‐Ras mutant spurs malignant cell proliferation (Table 7).^909^

### 7.6 Ubiquitination in CVDS

Ubiquitination is also critical in the development of CVDs. Elevated levels of myocardial ubiquitinated proteins have been observed in most primary causes of heart failure, such as cardiac muscle loss^910^ and cardiomyopathy.^911^ Changes in Ub protein ligases targeting myofibrillar and other cardiac proteins, such as atrogin‐1 (MAFbx) and MURF‐1, are associated with pathological cardiac remodeling.^912^ Moreover, ubiquitination is linked to atherosclerosis, with Ub–proteasome system (UPS) regulating eNOS activity and oxidative stress in the initiation and development of atherosclerosis. It also activates the NF‐κB pathway, affecting adhesion molecule expression, cytokine release, and proliferation. The UPS also influences foam cell formation and maintenance, which can impact atherosclerosis progression.^912^ Ubiquitination also plays an important role in the development of myocardial fibrosis. By inhibiting the expression of the E3 Ub ligase Pellino1, it is possible to prevent the production of α‐SMA, collagen I and collagen III, and thereby attenuate myocardial interstitial fibrosis. Pellino1 has been shown to facilitate the binding of NF‐κB and AP‐1 to the TGF‐β promoter, which regulates the fibrogenic capability of cardiac fibroblast cells and contributes to the development of fibrosis in the heart (Table 7).^913^

### 7.7 Ubiquitination in neurodegenerative diseases

Abnormal UPS function is closely related to the formation of protein aggregates in neurodegenerative diseases.^914^ The accumulation of insoluble Aβ in extracellular plaques and hyperphosphorylated tau protein (P‐tau) in NFTs within neuronal cytoplasm is a significant pathological factor observed in the brains of AD patients.^915^ Reduced UPS efficiency and the inhibited autophagy‐lysosomal pathway are significantly positively correlated with the abnormal accumulation of Tau at synaptic terminals.^916^, ^917^ Molecular misreading allows the formation of mutant proteins in the absence of gene mutations. Ubb^+1^, a frameshift mutation product of Ub protein in the brains of AD patients, can inhibit the function of the 26S proteasome and lead to an accumulation of a large number of pathogenic proteins, such as Aβ.^918^, ^919^ In addition, many ubiquitination‐related enzymes are abnormally expressed in AD, such as increased E2 ligase E2‐25K/Hip‐2^920^ and E3 ligase CHIP^921^ and RNF182,^922^ and decreased E3 ligases Parkin,^923^ HRD1,^924^ and the DUB UCHL1.^925^

Aβ42 can lead to hyperphosphorylation of the E3 Ub ligase Itch by abnormally activating the JNK signaling pathway. Hyperphosphorylated Itch ubiquitinates and degrades TAp73, leading to abnormal expression of important neuronal cyclins and causing neuronal apoptosis, which accelerates AD progression.^926^ The HECT family protein E6AP can activate the transcription of the *ESR2* gene encoding ER‐β, which reduces Aβ deposition in the hippocampus and improves learning and memory in AD rats.^927^ Loss of the E3 Ub ligase COP1 results in rapid accumulation of the TF C/EBPβ, which drives the expression of proinflammatory and neurodegeneration‐related genes and accelerates the neurodegeneration of AD.^928^ However, C/EBPβ is also the main TF responsible for the transcription of the scavenger receptor CD36. The E3 Ub ligase Peli1 can directly ubiquitinate and degrade C/EBPβ, which further reduces CD36 and inhibits the phagocytosis of microglial cells, slowing the clearance of Aβ in the brains of AD mice.^929^

While the precise mechanisms of PD are not yet entirely clear, it is widely acknowledged that α‐synuclein plays key pathophysiological roles as the main constituent of the cytoplasmic inclusions known as Lewy bodies. SIAH is an E3 Ub ligase that plays a key role in stress‐induced cell death and α‐synuclein degradation. This suggests that ubiquitination could protect against PD.^930^ However, some studies have found that SIAH can actually promote α‐synuclein aggregation and enhance its toxicity,^931^, ^932^ leading to more inclusions in dopaminergic neurons. Inhibiting SIAH could prevent Lewy bodies formation and be a potential therapy for PD.^933^

The accumulation of RNA‐binding protein TDP‐43 in neuronal cytoplasmic and intranuclear aggregates is a defining feature of neurodegenerative disorders, including ALS and frontotemporal lobar degeneration. TDP‐43 is typically modified with polyubiquitin chains that are mainly K48‐ or K63‐linked.^934^ Insufficient degradation of abnormally aggregated TDP‐43 protein leads to cell death and inflammation, which is an critical mechanism in the pathogenesis of ALS.^935^ Several studies have examined the potential therapeutic value of targeting TDP‐43 ubiquitination by preventing the removal of Ub chains, with conflicting results. Inhibition of the DUB USP14 promotes TDP‐43 clearance by maintaining Ub chains.^936^ However, in *Drosophila*, knockdown of the DUB UBPY increased TDP‐43 toxicity, despite retaining Ub chains (Table 7).^937^

### 7.8 Ubiquitination‐associated targeted therapies

An increasing number of studies have shown that ubiquitination‐related enzymes are a class of important drug targets. For example, the E3 ligase inhibitors thalidomide, lenalidomide, and permadomide have been used to treat multiple myeloma.^942^ These inhibitors bind to CRBN, activate the activity of CRL4^CRBN^ E3 Ub ligase, and induce the degradation of two important TFs, Ikaros/Aiolos, to kill cancer cells.^943^ With the continuous advancement of drug screening technology and chemical synthesis technology, an increasing number of novel small molecules targeting ubiquitination have been discovered, such as the E2 ligase UBE2 inhibitor NSC697923,^944^ E3 ligase MDM2 inhibitor BI‐0252,^945^ CRL inhibitors 33‐11 and KH‐4‐43,^946^ XIAP and cIAP1 inhibitor AT‐IAP,^947^ VHL inhibitor VH298,^948^ DUB USP2 inhibitor 6TG,^949^ USP7 inhibitor FT671,^950^ USP9X inhibitor G9,^951^ USP14 inhibitor IU1–47,^952^ and PSMD14 inhibitor THL.^953^ For proteins that pose challenges for targeting, regulating their upstream ubiquitination‐related enzymes has emerged as a novel drug development strategy.^954^

In 2001, the proteolysis‐targeting chimera (PROTAC) was first proposed as a chemical biology tool for targeted therapies. After 20 years of development, PROTAC technology has matured.^955^ The structures of PROTACs are similar to a dumbbell, connecting the “ligand of target protein” and “recruitment ligand of E3 ubiquitin ligase” through a linker.^691^, ^692^, ^956^ The tagged target proteins are recognized and degraded by the intracellular 26S proteasome.^955^ The most significant advantage of PROTAC technology is its ability to convert potentially undruggable targets into druggable ones.^957^ Moreover, PROTACs overcome drug resistance.^958^, ^959^, ^960^ Some PROTACs developed against cancer‐associated proteins outperform traditional small‐molecule inhibitors for cancer therapy. For example, oral PROTACs targeting ER and AR (ARV‐110 and ARV‐471) are used for the treatment of breast and prostate cancer, respectively.^961^, ^962^, ^963^

## 8 SUMOYLATION

SUMOylation is a PTM that covalently attaches small Ub‐like modifiers (SUMOs) to specific lysine residues in proteins.^964^ SUMO is a family of highly conserved small‐molecule proteins widely found in eukaryotes. There are currently five SUMO proteins (SUMO1–5) found in eukaryotes.^964^ The size of SUMOs is approximately 11 kDa. SUMO1, SUMO2, and SUMO3 contain 101, 103, and 95 amino acid residues, respectively.^965^ SUMO2 and SUMO3 cannot be distinguished by antibodies because their sequence similarity is as high as 97%, so they are usually collectively referred to as SUMO2/3. However, the sequence similarity between SUMO2/3 and SUMO1 is only 46%, and they usually show different biological functions in the body.^966^, ^967^ For example, SUMO1 mainly modifies some proteins in the physiological state, and SUMO2/3 mainly modifies stress proteins.^968^ SUMO1, SUMO2, and SUMO3 are widely expressed in all cells and organs.^969^ In contrast, SUMO4 is specifically expressed only in certain organs, such as the kidney, lymph nodes, and spleen,^968^ and SUMO5 is mainly expressed in the lung and spleen.^970^

**FIGURE 17:**
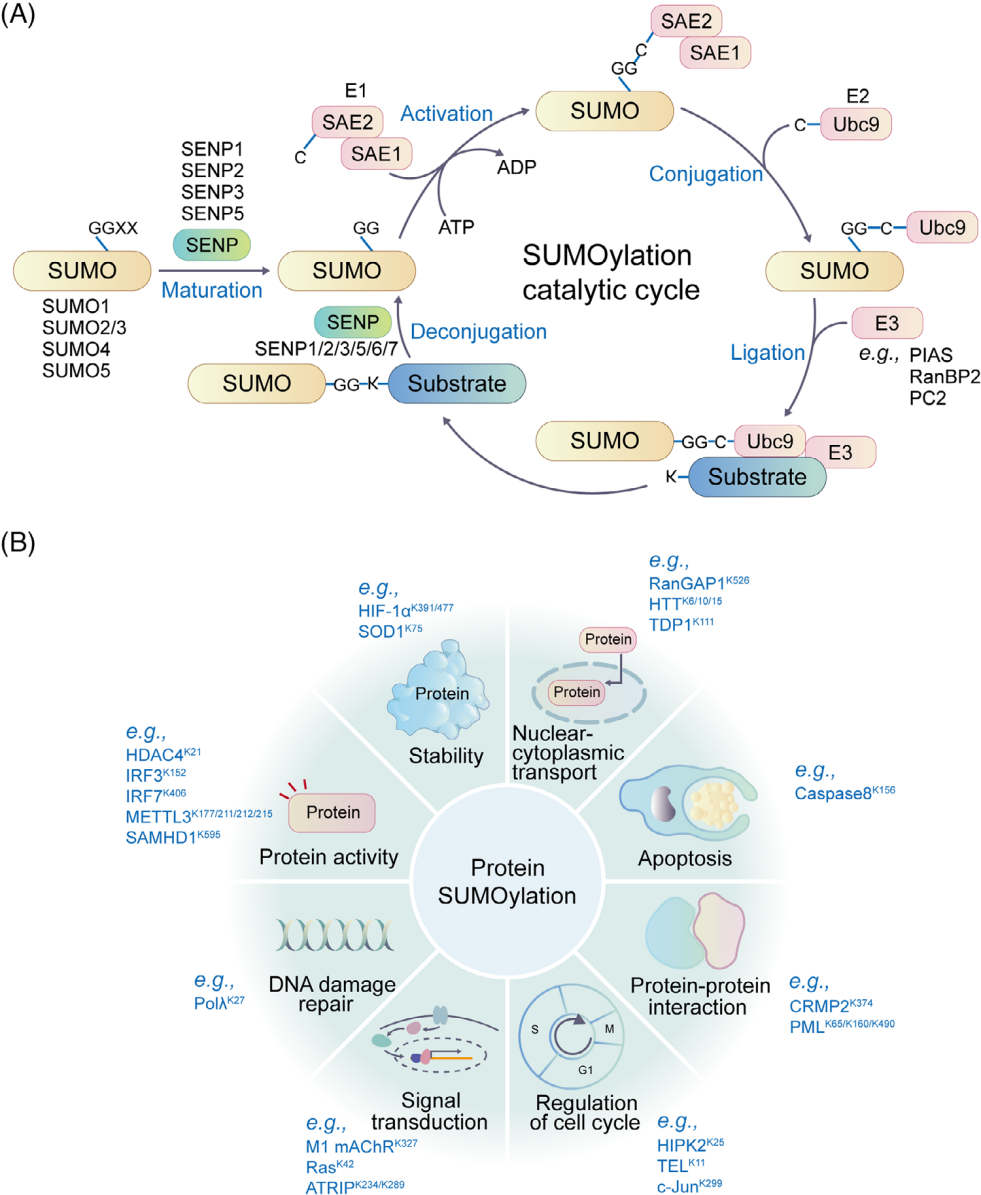
SUMOylation process and its functions. (A) The catalytic cycle of SUMOylation. SENP have endopeptidase activity to cleave SUMO precursors by exposing the carboxy‐terminal diglycine motifs essential for the conjugation to lysine residues in target proteins. SUMOylation is catalyzed by SAE1‐SAE2 (E1) and UBC9 (E2). The E3 ligases can facilitate the last step of SUMO conjugation. SENPs isopeptidase activity allows for the release of SUMO from target proteins. (B) Functions of SUMOylation. SUMOylation is extensively involved in signal transduction, and the regulation of protein activity, protein stability, protein–protein interaction, cell apoptosis, translocation, and DNA damage repair. Representative SUMOylation proteins and substrates are shown.

SUMOylation is similar to the Ub modification process, requiring SUMO‐activation enzyme (E1), SUMO‐conjugating enzyme (E2), and SUMO‐ligating enzyme (E3).^966^ The process of protein SUMOylation includes four steps. (1) *Maturation of SUMO proteins*. In this process, several amino acids of the C‐terminal sequence of SUMO precursor proteins are excised by SENP to expose the diglycine GG motif, which matures the SUMO proteins. (2) *Activation*. Under the action of ATP, mature SUMO is linked to the cysteine of the E1 activating enzyme (SAE1/SAE2 heterodimer in humans, called AOS1/Uba2 in yeast) through a thioester bond to activate the SUMO molecule. (3) *Conjugation*. The SUMO–E1 complex transfers SUMO to the E2 ligase Ubc9. (4) *Ligation*. SUMO is transferred from E2 to the lysine residues of protein substrates under the action of ligase E3 (Figure 17A).^968^ To date, the reported E3 ligases include protein PIAS family members (PIAS1, PIAS3, PIASxα, PIASxβ, PIASy), hPC2 (also known as PC2 and CBX4) and RanBP2.^967^, ^971^ SUMOylation is a highly dynamic and reversible process. The process of deSUMOylation is mainly regulated by SENPs.^972^ There are seven SENPs, namely, SENP1, SENP2, SENP3, SENP5, SENP6, SENP7, and SENP8.^973^, ^974^ The seven SENPs are divided into three families. The first family includes SENP1 and SENP2, which have broad substrate specificity and can bind to SUMO1/2/3. The second family includes SENP3 and SENP5, located in the nucleolus, and is mainly responsible for the removal of SUMO2/3. The third family includes SENP6 and SENP7, which remove SUMO2/3 from poly‐SUMO chains and are mainly localized in the nucleoplasm (Figure 17A).^966^, ^975^

SUMOylation is associated with the regulation of protein expression, localization, stability, and activity and is involved in various cellular processes, such as PPIs, intracellular localization, DNA repair, nucleocytoplasmic transport, TF activation, apoptosis, cell cycle, and gene transcription (Figure 17B).^964^, ^976^ The way SUMOylation works can be divided into two categories. First, SUMOylation directly affects protein functions by covalently modifying the protein. Second, the target protein indirectly regulates the biological function of the protein through noncovalent binding of the SUMO interact motif (SIM).^977^ Although SUMO and Ub are very similar in structure and occurrence process, their effects are often opposite. Ubiquitinated proteins are usually sent to the proteasome for degradation, while SUMOylated proteins are often more stable and less susceptible to degradation.^978^ For example, IκBα K21 can be modified by both SUMO and Ub, and SUMOylation can antagonize ubiquitination.^979^

### 8.1 Sumoylation in immune regulation

SUMOylation plays a significant role in the host immune response, as numerous SUMOylated proteins are involved in the development and activation of various immune cells.^980^ For example, SUMOylation of PKC‐θ is required for T cell activation and formation of a mature immunological synapse.^981^ Viruses can manipulate the process of SUMOylation through the SUMO pathway, while SUMOylation can also eliminate viral infections by regulating host antiviral immune components.^982^ Additionally, SUMOylation has also been linked to autoimmune diseases, especially RA. SUMOylation bidirectionally regulates immune pathways, which can prevent hyperresponsiveness of the immune system and inhibit the development of inflammatory and autoimmune diseases.^983^ In RA patients, the expression of SUMO1 and SUMO2 is elevated in fibroblast‐like synoviocytes (FLSs), especially SUMO1, and FLSs are important for promoting RA pathogenesis. SUMO‐1 suppression could be protective against joint destruction in RA by inhibiting aggressive behavior of RA FLSs.^984^ Moreover, SUMOylation is crucial in maintaining the stability of the intestinal epithelial barrier by changing the intestinal flora, regulating immune cells, and regulating cytokines, such as IL‐6, TNF‐α, and IFN‐γ. SUMOylation of intestinal epithelial cells (IECs) can reduce the severity of inflammatory bowel disease (IBD) by inhibiting the activity of master regulators, including the serine‐threonine kinase AKT1 (Table 8).^985^

**TABLE 8:** Representative SUMOylation events in health and diseases.

Diseases		Protein substrates	Effects
Immune diseases	Inflammation	NLRP3	TRIM28 binds to NLRP3, catalyzes SUMO1, SUMO2, and SUMO3 modification of NLRP3, promotes NLRP3 expression, and enhances NLRP3 inflammasome activation.^1057^
	RA	IκB‐α	SUMOylation of IκB‐α prevents NF‐κB from ubiquitination, which further inhibit nuclear migration and production of inflammatory mediators.^983^
	IBD	IECs	SUMOylation of IECs reduces the severity of IBD by inhibiting the activity of master regulators.^985^
Neurodegenerative diseases	AD	Tau	SUMOylation promote Tau phosphorylation and inhibit Tau degradation to promote NFT formation.^1058^
		APP	SUMO1 modification of APP promotes the generation of Aβ plaques in AD mouse models.^1042^
	PD	α‐Synuclein	SUMO1 modification promotes its aggregation to form Lewis bodies.^1045^
	ALS	TDP‐43	SUMOylation promotes the formation of TDP‐43 aggregates and affects the nuclear localization of TDP‐43, involving in the pathological process of ALS.^1049^
	HD	HTT	SUMOylation of HTT increases its insolubility and toxicity, leading to the accumulation of HTT.^1059^
CVDs	Ischemia	Drp1	DUSP6 SUMOylation at K234 is antiapoptotic during reperfusion.^1060^
	Myocardial IRI	PPAR‐γ	Overexpression of PIAS1 alleviates injury of myocardial I/R by increasing SUMOylation of PPAR‐γ at K365 and downregulating NF‐κB pathway.^1061^
	IRI	SERCA2a	SERCA2a SUMOylation at K585 enhances intracellular mitochondrial membrane potential and reduces cell apoptosis, which promotes the recovery of cardiac function and reduces the infarct area in vivo.^1062^
Cancers	Various cancers	β‐catenin	SUMOylation of β‐catenin prevents its ubiquitination and degradation.^1063^
	Prostate cancer	AR	Modification by SUMO1 attenuates AR's transcriptional activity.^1064^
	Prostate cancer	HK2	SUMOylation‐deficient HK2 promotes the growth of prostate cancer cells that resist chemotherapeutic drug‐induced apoptosis.^1028^
	APL	PML/RARA	Arsenic‐enhanced PML/RARA SUMOylation promotes degradation.^1065^
	Breast cancer	hTERT	CBX4 regulates SUMOylation of hTERT to promote the migration and invasion of breast cancer cells.^1036^
	HCC	METTL3	SUMOylation of METTL3 regulates HCC progression by controlling Snail mRNA homeostasis in a m6A methyltransferase activity dependent manner.^1037^
Aging	Aging	UBC9	The SUMOylation of UBC9 at K49 is conducive to its relocation to PML‐NBs and promotes the translocation of target proteins into nucleus, which can transmit the antiaging phenotype.^1012^
		Sp1	A gradual decrease in Prdx6 expression is associated with increased Sp1 SUMOylation and decreased Sp1 expression during aging.^1013^, ^1014^
Metabolic disorders	Diabetes	ICA512	PIASy reduces the interaction between ICA512 and STAT5 through SUMOylation of ICA512 and inhibits insulin secretion.^1019^
	Diabetes mellitus and myocardial infarction	ERK5	ERK5 SUMOylation enhances the inhibition of ROS‐mediated ERK5 transcription, which leads to the deterioration of left ventricular function after myocardial infarction in diabetic patients.^1021^
	Obesity	ERp44	SUMOylation of ERp44 enhances Ero1α ER retention, thereby resulting in ER stress associated with aberrant lipid metabolism and obesity.^1066^

RA, rheumatoid arthritis; IBD, inflammatory bowel disease; IECs, intestinal epithelial cells; AD, Alzheimer's disease; NFT, neurofibrillary tangle; APP, amyloid precursor protein; PD, Parkinson's disease; ALS, amyotrophic lateral sclerosis; HD, Huntington's disease; HTT, huntingtin; IRI, ischemia–reperfusion injury; AR, androgen receptor; APL, acute promyelocytic leukemia; HCC, hepatocellular carcinoma.

### 8.2 Sumoylation in development

The role of SUMOylation in embryonic development has been confirmed in *Drosophila*,^986^ nematodes,^987^ zebrafish,^988^ Xenopus laevis,^989^ silkworms,^990^ and various plants.^991^ For example, an imbalance of SUMO leads to defects in embryonic patterning in *Drosophila*,^992^ while the absence of SUMO activity disrupts multiple signaling pathways and causes neural tube and heart defects in Xenopus embryos.^989^ In mammals, dysregulation of SUMO leads to defects in embryonic development, craniofacial defects,^993^ and even embryonic lethality. SUMO‐deficient mice suffer from severe developmental disabilities, and mice die during embryonic development. Further studies have found that only SUMO2‐deficient mice die earlier in embryonic development, while SUMO1‐ or SUMO3‐deficient mice survive and reproduce well.^969^, ^994^, ^995^

The balance of SUMOylation in organisms is critical to the development of tissues and organs such as the heart,^996^ blood vessels,^997^ reproductive system,^998^ lung,^999^ and nervous system.^1000^ Too high or too low SUMO levels may lead to organ dysfunction. SUMO1 has important and specific functions in normal heart development. Both hetero‐ and homozygous SUMO‐1 knockout mice exhibited atrial septal defects and ventricular septal defects with high mortality rates, which were rescued by cardiac reexpression of the SUMO‐1 transgene.^1001^ Similarly, high expression of SENP2 can enhance deSUMOylation in the mouse heart, leading to congenital heart defects and cardiac dysfunction in mice.^1002^ Angiogenesis is essential for embryonic development and tissue growth, and the NOTCH pathway is a significant negative regulator of endothelial sprouting and vascular growth. SUMOylation negatively regulates angiogenesis by targeting endothelial NOTCH signaling. Endothelial SENP1 deletion in newly generated mice significantly delayed retinal vascularization by maintaining prolonged NOTCH1 signaling.^997^ The dynamic SUMOylation of endothelial FGFR1 regulates the balance of the angiogenesis core pathways VEGF/VEGFR and FGF/FGFR and enables the body to complete angiogenesis in different microenvironments.^1003^ SUMOylation also plays an important role in the generation of germ cells. SUMO1 and SUMO2/3 function at different stages of male meiosis and precisely regulate the formation of sex chromosomes.^1004^ C/EBPα is a core TF that regulates cell growth and differentiation. During lung development, C/EBPα is SUMOylated and participates in C/EBPα‐mediated lung growth and differentiation.^1005^ Utf1 is a key SUMOylation target during neurogenesis and determines normal neurogenesis.^1006^ SENP2 can regulate the calcium homeostasis of mouse neurons through the SENP2–PLCβ4 signaling axis and then regulate neurogenesis in the hippocampus.^1007^ Furthermore, SUMOylation is important to maintain the development of human induced pluripotent stem cells.^1008^

### 8.3 Sumoylation in aging

During normal aging, SUMOylation is very important.^1009^, ^1010^ In *C. elegans*, enhanced insulin/IGF signaling activity promotes SUMOylation of the germ cell protein CAR‐1, resulting in shortened lifespan and impaired proteostasis.^1011^ The degree of SUMOylation at Lys49 of UBC9 increases during aging. It has been found that the SUMOylation of UBC9 at Lys49 is conducive to its relocation to PML‐NBs and promotes the translocation of target proteins into the nuclear bodies, which can transmit the antiaging phenotype. Whereas SUMOylation of proteins by the non‐SUMOylated UBC9 promotes senescence.^1012^ Persistent DNA damage triggers cells to undergo apoptosis or senescence to prevent replicating a damaged genome. Sp1, a protein involved in double‐strand break (DSB) repair, has been linked to aging, with Sp1 levels decreasing with age. Proteasomal degradation of Sp1 in senescent cells is mediated via SUMOylation, where SUMOylation of Sp1 on lysine 16 is increased in senescent cells. Prdx6 is important in maintaining redox homeostasis and localizes to ROS‐producing organelles. A gradual decrease in Prdx6 expression is associated with increased Sp1 SUMOylation and decreased Sp1 expression during aging.^1013^, ^1014^ SUMOylation of KLF1 at K74 regulates its transcriptional activity during erythroid differentiation. K74 SUMOylation deficiency contributes to health and longevity in mice.^1015^, ^1016^ SUMO can also affect lifespan by affecting the mitochondrial unfolded protein response (UPR). During mitochondrial stress, ULP4 prolongs lifespan in *C. elegans* by removing SUMOylated DVE1 and ATFS1 (Table 8).^1017^

### 8.4 Sumoylation in metabolic disorders

SUMOylation also plays an important role in the regulation of cellular metabolism.^966^ SUMOylation acts on factors related to cholesterol homeostasis, including SREBPs and members of the nuclear receptor superfamily, such as LXR, FXR, LRH1, and PPAR. These receptors are potential therapeutic targets for lipid metabolism disorders.^1018^ Studies have found that insufficient insulin secretion or output disorders will eventually lead to diabetes. The E3 SUMO ligase PIASy can inhibit insulin secretion by reducing the interaction between ICA512 and STAT5 through SUMOylation of ICA512.^1019^ Furthermore, SUMOylation can prevent stress‐induced β‐cell apoptosis by upregulating the levels of antioxidant genes, including *Ho‐1*, *Cat*, and *Nqo‐1*.^1020^ ERK5 is one of the major targets of SUMOylation in diabetic hearts. ERK5 SUMOylation enhances the inhibition of ROS‐mediated ERK5 transcription, which leads to the deterioration of left ventricular function after myocardial infarction in diabetic. The phenotype can be significantly reversed by inhibiting ERK5 SUMOylation.^1021^ Overexpression of SUMO4 promotes SUMOylation of IκBα, which inhibits the activation of NF‐κB by external stimuli. This modification is considered to be related to type 1 diabetes (Table 8).^1022^

### 8.5 Sumoylation in cancers

The important role of SUMOylation in human tumorigenesis has gradually emerged. SUMOylation enzymes E1, E2 and E3 are highly expressed in many types of tumors. For example, Ubc9 is associated with the occurrence and development of ovarian carcinoma, advanced melanomas, colon cancer and primary prostate cancer.^968^ SUMO E3 ligase PIAS1 has been implicated in the regulation of several oncogenes and tumor suppressors. In B‐cell lymphoma, PIAS1‐mediated SUMOylation of Myc leads to a longer half‐life of the protein and increased oncogenic activity, contributing to the development of B‐cell lymphoma.^1023^ PIAS1 is highly expressed in prostate cancer and leads to accelerated tumor cell proliferation by inhibiting p21 expression.^1024^ High expression of PIAS3 is very common in CRC.^1025^

SUMOylation has an impact on cancer cell signaling and gene networks that regulate DNA damage, metabolism, inflammation and immunity, which provides link with carcinogenesis, proliferation, metastasis and apoptosis. SUMOylation is important for mammalian DNA damage response. BRCA1 participates in the DNA damage response and mutations in BRCA1 are associated with a high risk of breast and ovarian cancer. Hybrid SUMO‐Ub chains are synthesized by RNF4, a SUMO‐targeted Ub E3 ligase, and recognized by RAP80 to promote BRCA1 recruitment and repair of DNA DSBs. PIAS1 and PIAS4 are necessary for efficient Ub‐adduct formation by RNF8, RNF168, and BRCA1 at DNA damage sites.^968^, ^1026^

SUMOylation is also involved in the regulation of cancer metabolism. For example, K270 of PKM2 can be modified by SUMO1, which promotes its transformation from a tetramer to a dimer. After entering the nucleus, it binds to the SIM on RUNX1, recruits RUNX1, and regulates the differentiation process of leukemia cells.^1027^ K315 and K492 are the SUMOylation sites of HK2. SUMOylation‐deficient HK2 enhances its binding to mitochondria, which reduces mitochondrial oxidative phosphorylation and increases glycolysis and lactic acid production. This process promotes the growth of prostate cancer cells that resist chemotherapeutic drug‐induced apoptosis.^1028^

SENP3 is involved in the regulation of immune cell function, which in turn affects the progression of tumor development.^1029^ Knockdown of SENP3 in DCs inactivates the STING‐dependent type‐I IFN signaling pathway and weakens the antitumor immune response. In the tumor microenvironment, SENP3 senses oxidative stimulation from DCs by deSUMOylating IFI204 and activating the STING signaling pathway.^1030^ SENP7 can regulate the metabolic homeostasis and antitumor activity of CD8^+^ T cells in response to oxidative stress. The key deSUMOylation substrate of SENP7 in this process is PTEN.^1031^

SUMOylation plays a role in cell differentiation and carcinogenesis. *Myc* mutation results in the constitutive expression of Myc protein, which leads to the uncontrolled expression of various genes, including those that drive cell proliferation, ultimately leading to cancer. Loss of SAE1/2 enzymatic activity is synthetically lethal with Myc. SUMOylation‐dependent Myc switchers (SMS genes) are necessary for Myc‐driven tumorigenesis. Patients with breast cancer who have *Myc* overexpression and low expression of SAE1/2 show significantly reduced cancer cell metastasis and improved survival compared with those with high SAE1/2 expression. Similarly, in a *Myc*‐overexpressing PDAC model, the highly selective SAE small‐molecule inhibitor ML‐93 significantly inhibited protein SUMOylation and tumor growth.^1032^ In addition, BIRC5, EG5, and TPX2 are synthetic lethal partners of Myc. The functions of these three proteins are also dependent on SUMOylation.^1033^, ^1034^ Moreover, SUMOylation is associated with tumor metastasis.^1035^ hTERT is the catalytic component of human telomerase, and SUMOylation of hTERT promotes the migration and invasion of breast cancer cells.^1036^ The SUMOylated E2‐conjugating enzyme Ubc9 modifies METTL3 through SUMO1, and the SUMOylated METTL3/Snail axis is correlated with high metastatic potential of liver cancer (Table 8).^1037^

### 8.6 Sumoylation in neurodegenerative diseases

Tight control of the CNS by SUMOylation is critical for maintaining neuronal cell viability, function, and connectivity.^1038^ SUMOylation plays important roles in the repair of DNA damage in neurons, axonal mRNA transport, and the regulation of synaptic plasticity.^1039^ Dysregulation of SUMOylation has been observed in the AD brain, with increased levels of hippocampal SUMO1 transcription possibly contributing to Aβ aggregations and impaired learning and memory abilities.^1040^ In fact, several proteins involved in the physiopathological process of AD, such as BACE1, GSK3‐β tau, Aβ precursor protein (AβPP), and JNK, are in fact subject to protein SUMOylation or interactions.^1041^ Mature Aβ is produced by hydrolysis of APP. SUMO1 modification of APP promotes the generation of Aβ plaques in AD mouse models.^1042^ In addition, melatonin can induce the SUMOylation of the APP intracellular domain (AICD). SUMOylation of AICD activates the transcription of two Aβ‐degrading enzymes, which promotes Aβ degradation and delays the occurrence of AD.^1043^ SUMO can also modify Tau. SUMOylation and phosphorylation of Tau promote each other and inhibit ubiquitination‐dependent Tau degradation, thereby promoting NFT formation.^1044^ α‐synuclein has two SUMOylation sites, Lys96/102, which are modified by SUMO1.^1044^ SUMOylation may regulate the normal and pathological functions of α‐synuclein, including degradation, intracellular distribution, and PPIs and aggregation.^1045^ SUMOylation promotes PD onset by preventing proteasomal degradation of α‐synuclein.^1046^ SUMOylation is also involved in regulating the pathogenesis of ALS.^1047^ Aggregation of SOD1 is characteristic of patients with SOD1 variant‐induced ALS. The modification of SOD1 by SUMO3 enhances the aggregation of familial ALS (fALS)‐linked SOD1 mutants, while SENP1 decreases the number of cells exhibiting SOD1‐mutant aggregation.^1048^ TDP‐43 can also be SUMOylated. SUMOylation promotes the formation of TDP‐43 aggregates and affects the nuclear localization of TDP‐43, which is involved in the pathological process of ALS (Table 8).^1049^

### 8.7 Sumoylation in CVDS

SUMOylation is also closely related to the development, metabolism, and pathology of the heart. For example, SUMO1 is essential for normal cardiac development, and cardiac‐specific overexpression of SUMO1 improves cardiac functions. SUMO1 mutant or knockout mice are more prone to congenital heart defects.^1050^ Specifically, UBC9 is the sole E2 enzyme essential for GATA4's role in cardiac development and function. It boosts GATA4's transcriptional activity and affects its nuclear localization. UBC9 adds a SUMO group to GATA4 at K366, which activates particular gene expression in pluripotent cardiac cells.^1051^

Protein SUMOylation and its dysregulation are implicated in various CVDs, including atherosclerosis, heart failure, and ischemic cardiomyopathy. Several factors, including ERK5, NF‐κB, p53, and PKC, undergo SUMOylation, which contributes to atherosclerosis progression.^1051^ For example, SUMO1‐mediated SUMOylation of NF‐κB inhibits IκBα degradation and reduces NF‐κB activation.^1052^ In contrast, SUMO2/3 modification promotes IκBα detachment from NF‐κB and enhances NF‐κB activation, ultimately inducing atherosclerosis.^1052^, ^1053^ Additionally, SUMOylation of HSF2, myocardin, PARIS, PPARγ1, and SERCA2a plays crucial roles in heart failure progression.^1051^ The protein levels of SUMO1 and SUMOylated SERCA2a are significantly decreased in failing hearts, whereas increased SUMOylation of SERCA2a improves myocardial contractility and ventricular function in heart failure mice.^1054^ Conversely, hypoxia induces SUMOylation of HIF‐1α, which promotes HIF‐1α degradation, whereas SENP1 deSUMOylates HIF‐1α and enhances its stability.^1055^ Reduced SENP1 levels exacerbate ischemia/reperfusion (I/R) injury in cardiomyocytes through the HIF‐1α pathway, while HIF‐1α overexpression counteracts the detrimental impact of SENP1 downregulation on cell death (Table 8).^1056^

### 8.8 Sumoylation‐associated targeted therapies

Targeted cancer therapy may be achieved by inhibiting the SUMO pathway.^1067^ In 2021, Bellail's team screened the hit compound CPD1 that can specifically target and degrade SUMO1 from the drug‐like compound library of the National Institutes of Health (NCI). CPD1 specifically reduces SUMO1 protein levels without affecting SUMO1 mRNA levels. Further druggability optimization identifies the first highly selective SUMO1 degrader, HB007. HB007 inhibits the proliferation of various tumor cells by selectively degrading SUMO1.^1068^ The highly selective SAE inhibitor TAK‐981 can significantly upregulate the expression of IFN1 and activate IFN1‐dependent innate immune cells, including macrophages, NK cells, DCs, and T cells, promoting antitumor immune responses.^1069^ Moreover, TAK‐981 is currently in phase 1 clinical trials in patients with solid tumors and lymphomas.^1069^ Although there are still many blind spots on the mechanism of SUMOylation involved in the occurrence and development of tumors, AD/PD, and CVDs at this stage, a large number of experiments have confirmed the role of SUMOylation in these diseases. Therefore, drugs targeting the SUMOylation mechanism may represent a promising treatment strategy.^1070^

## 9 GLYCOSYLATION

Protein glycosylation is a process in which sugar groups are transferred to proteins catalyzed by glycosyltransferases, which predominantly occurs in the ER and Golgi apparatus. Most glycans exist on the surface of cells and secreted proteins, with intricate and varied structures.^1071^, ^1072^, ^1073^ In contrast, the types of glycosylation existing in the nucleus and cytoplasm have simple structures and are highly dynamic.^1074^, ^1075^ The structural diversity and extensive distribution of protein glycosylation make it one of the most prevalent forms of PTMs in humans.^1076^

Protein glycosylation is a complicated process that involves multiple steps. The human genome contains approximately 700 genes related to glycosylation and deglycosylation, including enzymes, transporters, and chaperones.^1077^, ^1078^, ^1079^ Of these genes, approximately 200 encode glycosyltransferases involved in the construction of complex glycans on proteins.^1078^ These glycans are assembled from ten monosaccharides, including N‐acetylgalactosamine (GalNAc), N‐acetylglucosamine (GlcNAc), xylose (Xyl), fucose (Fuc), galactose (Gal), mannose (Man), glucose (Glc), glucuronic acid (GlcA), iduronic acid (IdoA), and sialic acid (SA).^1076^, ^1077^ The monosaccharides are linked to nucleotides or lipids to form an activated donor substrate, which is then extended through the action of glycosyltransferases to form approximately 10^12^ different glycan structures.^1077^, ^1080^, ^1081^ There are four ways of linking glycans to proteins, including N‐glycosylation to asparagine (Asn) residues, O‐glycosylation to serine (Ser), threonine (Thr) or tyrosine (Tyr) residues (O‐linked monosaccharides include GalNAc, GlcNAc, Gal, Glc, Man, Fuc, and Xyl), C‐mannosylation to tryptophan (Trp) residues, and glypiation. Based on these linking methods, protein glycosylation is divided into 14 different types, including N‐glycosylation, 11 types of O‐glycosylation, C‐mannosylation, and glypiation.^1076^, ^1082^ These glycosylation modifications play important roles in regulating various intracellular and extracellular protein functions and are involved in a variety of biological processes in humans (Table 9).

**TABLE 9:** Protein glycosylation through different linkages and their function.

Types	Linkages	Enzymes	Modified sequence in glycoproteins		Functions of glycosylation
			Sequence motifs	Specific domain	
N‐glycosylation	GlcNAc‐β‐Asn	OST complex (STT3A/STT3B)	N‐X‐T/S, X≠P N‐G; N‐X‐C/V, X≠P	None	Protein stability,^1118^ protein folding and quality control,^1119^ self/nonself recognition,^1120^ cell adhesion^1121^, immunotherapy,^1122^ receptor activation and endocytosis,^1123^ glycoediting and drug delivery.^1124^
O‐glycosylation	GalNAc‐α‐Ser/Thr	GALNT1–20	Weak isoform specific motifs^1093^	None	O‐glycan shielding is essential for secretion of an active protein,^1125^ participates in immunological recognition of the immune system,^1082^ protects membrane proteins from ectodomain shedding,^1090^ increases the half‐life of peptide hormones in circulation,^1126^ modulates the interaction between viral proteins and host surface receptors.^1127^ O‐glycosylated mucins expressed at mucosal surfaces such as the respiratory and GI tract can form an effective barrier against pathogens.^1128^ The particular glycosylation of LDLR class A repeat linker regions by GalNAc‐T11 alters the rate of uptake by cargo receptors.^1129^ O‐glycan is also important for leukocyte extravasation.^1130^
		GALNT11	C^6^‐X_3‐5_‐T‐C^1^ ^1131^	LA	
	GlcNAc‐β‐Ser/Thr	EOGT	C^5^‐X_2_‐(G/P/S)‐(Y/F/W)‐(T/S)‐G‐X_2_‐C^6^ ^1132^	EGF	
	GlcNAc‐β‐Ser/Thr	OGT	None	None	
	Gal‐β‐Hyl	COLGALT1–2	X‐Hyl‐Gly	Collagen repeats	
	Glc‐α‐Tyr	GYG	Tyr194 of GYG	None	
	Glc‐β‐Ser	POGLUT1	C^1^‐X‐S‐X‐(A/P)‐C^2^	EGF	
		POGLUT2–3	C^3^‐X‐N‐T‐X‐G‐S‐(F/Y)‐X‐C^4^		
	Fuc‐α‐Ser/Thr	POFUT1	C^2^‐X‐X‐X‐X‐(S/T)‐C^3^	EGF	
		POFUT2	C‐X‐X‐(S/T)‐C‐X‐X‐G	TSR	
	Man‐α‐Ser/Thr	POMT1–2	None	None	
		TMTC1–4	None	EC	
		Unknown	None	IPT	
	Xyl‐β‐Ser	XYLT1–2	a‐a‐a‐a‐G‐S‐G‐a‐(a/G)‐a (“a” represents Asp or Glu)	None	
C‐mannosylation	Man‐α‐Trp	DPY19L1–4	W‐X‐X‐W	TSR	Support folding, enhance stability of thrombospondin repeats,^1106^ regulate protein folding and maturation,^1133^ play a key role in the folding, sorting and secretion of substrate proteins.^1107^, ^1108^
Glypiation	Pr‐C(O)EthN‐6‐P‐Man	Transamidase	Carboxy‐terminal hydrophic segment	None	Regulate protein location on cell membrane,^1116^ cell signal transduction, cell adhesion, and immune recognition.^1117^

**FIGURE 18:**
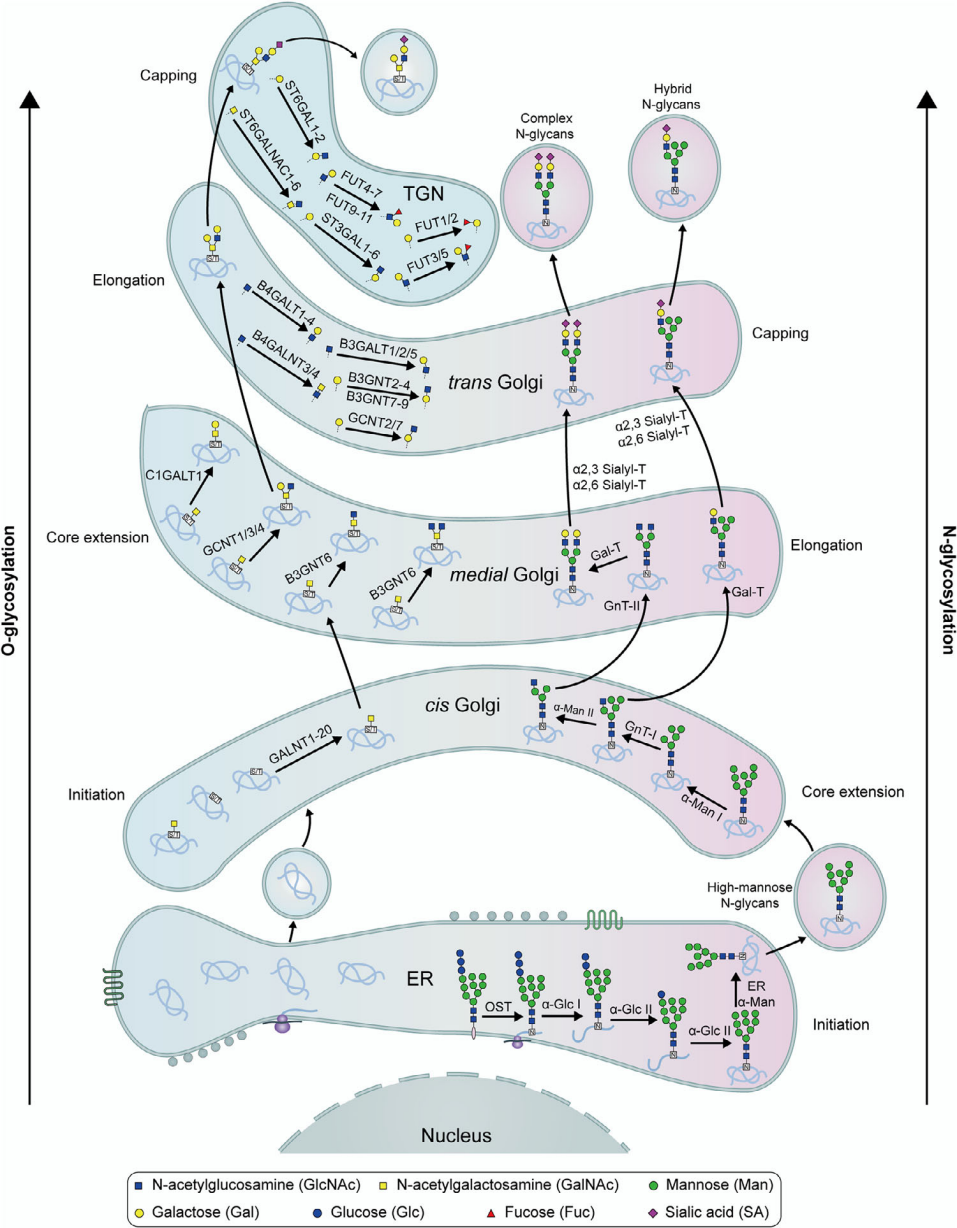
Overview of human N‐ and O‐glycosylation in the ER and Golgi apparatus. On the right side, the biosynthesis of complex‐type N‐glycans is shown. On the left side, the biosynthesis O‐glycosylation is shown.

N‐glycosylation occurs on Asn residues of proteins. The sugar complex with GlcNAc2Man3 as the core is linked to the N atom on the Asn side chain via GlcNAc, and various enzymes are recruited to remove or add monosaccharides. Based on the polysaccharide structure, glycans can be classified into high‐mannose N‐glycans, hybrid N‐glycans, and complex N‐glycans.^1083^ The oligosaccharyltransferase (OST) complex catalyzes the initiation of N‐glycosylation in the ER, transferring a 14‐saccharide precursor structure (GlcNAc2Man9Glc3) to the Asn‐X‐Ser/Thr (X represents other amino acids except Pro) motif. This glycosylation process can be cotranslational or posttranslational, and regulated by the STT3A and STT3B catalytic subunits of OST, respectively.^1084^, ^1085^ The OST–STT3A complex is mainly responsible for the cotranslational glycosylation of nascent peptides when they enter the ER cavity, while the OST–STT3B complex is mainly responsible for the release of the glycans on misfolded N‐glycoproteins into oligosaccharides, which are the main source of oligosaccharides.^1084^, ^1085^, ^1086^ After processing in the ER, the glycans in the precursor structures are moved to the *cis*‐Golgi and modified by a series of specific mannosidases and then transferred to the inside of the Golgi for further processing and maturation (Figure 18).

Protein O‐glycosylation mainly occurs on amino acids with functional hydroxyl groups on the side chains, such as Ser and Thr. The monosaccharides linked to Ser and Thr residues in humans are mainly GalNAc and GlcNAc.^1087^, ^1088^, ^1089^ GalNAc‐type O‐glycosylation is present on extracellular and secreted glycoproteins, such as mucins,^1090^, ^1091^ and this type of O‐glycosylation is initiated in the Golgi apparatus and regulated by up to 20 GalNActransferases (GALNTs), of which 15 isozymes have been demonstrated to be active enzymes.^1091^, ^1092^ GALNTs exhibit some specificity, but there is no specific amino acid motif for recognition in substrate proteins. The site and type of protein O‐glycosylation are coregulated by different transferases in a cooperative manner (Figure 18).^1091^, ^1093^ O‐glycosylation usually has the same glycan core structure (e.g., cores 1–4, sialyl‐Tn antigens and terminal GalNAc), and the glycan is further extended on the basis of the core structure, which protects glycoproteins and cell surfaces from external stress and microbial infection, which affects the self‐recognition process of the immune system.^1094^, ^1095^ GlcNAc‐type O‐glycosylation mainly exists on glycoproteins in the cytoplasm, mitochondria, and nucleus.^1096^, ^1097^, ^1098^ Unlike O‐GalNAcylation, O‐GlcNAcylation starts in the ER. It does not typically occur in the Golgi apparatus, and the glycan does not extend further. The formation of O‐GlcNAcylation is mainly regulated by O‐GlcNAc transferases (OGTs) and O‐GlcNAcases (OGAs).^1089^, ^1099^, ^1100^ O‐GlcNAcylation also plays an important role in cells and is closely associated with protein stability and localization, intracellular signal transduction, chromatin remodeling, and mitochondrial function.^1101^ Although OGTs and OGAs exist in different subcellular compartments in different forms, they share the same function of adding GlcNAc to protein substrates or removing GlcNAc from protein substrates. This maintains the homeostasis of O‐linked GlcNAc and plays an important role in regulating cellular functions.^1102^, ^1103^

C‐Mannosylation is a relatively rare form of protein glycosylation. The monomeric D‐mannopyranose bound with the C‐2 position of the pyrrole ring of the tryptophan residue to form a carbon—carbon bond.^1104^ C‐Mannosylation occurs on proteins in the ER and is regulated by four dpy‐19‐like C‐Man transferases (DPY19),^1105^ which transfer monomeric α‐mannose to the substrate on the first Trp residue in the Trp‐X‐X‐Trp/Cys motif.^1106^ C‐Mannosylation plays an important role in protein folding, sorting, and secretion,^1107^, ^1108^ and it has been determined that 18% of human proteins undergo C‐mannosylation during secretion and transmembrane transport.^1109^ The known protein substrates of C‐mannosyltransferases include the TSR superfamily and type I cytokine receptor family.

Glypiation is a special type of glycosylation that localizes proteins to the cell membrane via glycosylphosphatidylinositol (GPI). The GPI anchor contains a phosphoethanolamine linker, which binds to the C‐terminus of the target protein. The GPI sugar chain core structure contains a phospholipid tail, which anchors the structure to the membrane.^1110^ Similar to the synthesis of glycan precursors required for N‐glycosylation, the biosynthesis of GPI anchors begins on the cytoplasmic side of the ER. Glypiation‐modified proteins usually have two signal sequences. The N‐terminal and C‐terminal signal sequences determine transport into the ER and recognition by the GPI transamidase complex GPIT, respectively.^1111^ C‐terminal sequence recognized by GPIT for covalent binding to the GPI anchor. During the synthesis of GPI anchors, sugars on membrane‐embedded phosphatidylinositol (PI) molecules originate from sugar nucleotides and dolichol‐P‐mannose around the ER. The residues at the phosphoethanolamine (EtN‐P) linker are provided by phosphatidylethanolamine in the ER cavity.^1112^, ^1113^ GPI anchors recruit specific proteins to the cell membrane for their crucial roles, and enzymes such as phospholipase C are responsible for the cleavage of GPI anchors, which regulate cell membrane protein localization.^1114^, ^1115^, ^1116^ The diverse glypiation makes it critical in cell signaling, cell adhesion, and immune recognition.^1117^

### 9.1 Glycosylation in development

**FIGURE 19:**
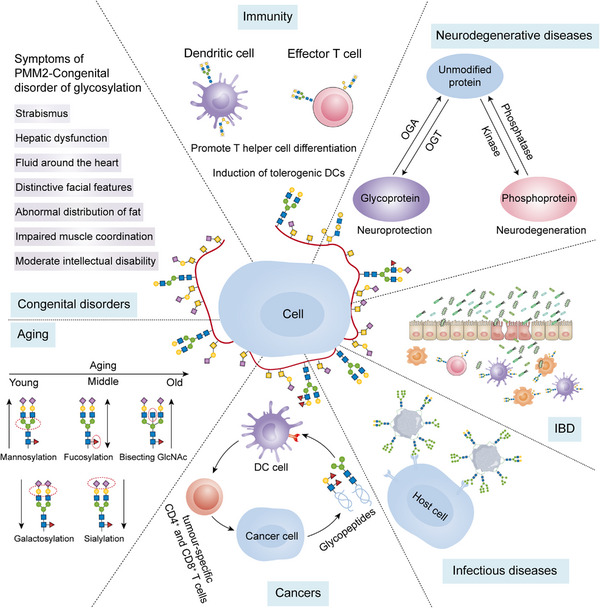
Functions of glycosylation in health and diseases, such as congenital diseases, immune regulation, neurodegenerative diseases, IBD, cancers, aging, and infectious diseases.

During the developmental process, protein glycosylation occurs at various times and locations, and these characteristic glycans cover the surface of nearly all cells. The numerous and complex structures of glycans provide strong support for cell‐to‐cell communication during growth and development.^1134^, ^1135^ Genetic defects in glycosylation are often embryonic lethal.^1136^ Congenital disorders of glycosylation (CDGs) are diseases caused by disorders of glycoprotein synthesis, with various clinical manifestations, such as the appearance of special facial features and lesions in the organs of the body, which can be divided into Type I and Type II CDGs.^1137^, ^1138^ The causative factors of CDGs include abnormal activation, presentation, or transport of glycolipid precursors, abnormal expression or activity of glycosidases or glycosyltransferases, and abnormal functions of proteins that control glycosylation or maintain the Golgi apparatus.^1082^ Furthermore, during embryonic development, N‐glycosylation plays a key role in the generation of hematopoietic stem cells (HSCs) from arterial endothelial cells through the process of endothelial to hematopoietic transition (EHT) and is a determinant of hematopoietic fate.^1139^ CDGs often have serious consequences, suggesting the important roles of protein glycosylation in maintaining the normal growth and development of individuals (Figure 19).

### 9.2 Glycosylation in aging

Protein glycosylation is a dynamic process highly sensitive to aging,^1140^ and acts as a potential important molecular effector in aging and age‐related diseases.^1141^ Numerous studies have shown that the glycan at Asn279 on IgG heavy chains undergoes changes during aging that are often similar to those found in inflammatory states,^1142^ but the mechanism by which the glycans at Asn279 on IgG heavy chains are altered is unclear (Figure 19).^1143^, ^1144^ In addition, reduced expression of the glycosyltransferase B4GALT1 can prevent senescence‐associated IgG glycan changes and improve the senescence phenotype.^1145^ The glycosyltransferase ST6GAL1 can alter the glycans of fibroblasts, leading to the transition of fibroblasts to proinflammatory cells.^1146^ Sialic acid‐binding immunoglobulin‐like lectins (Siglecs) act as inhibitory receptors on the immune cell surface.^1147^ The activity of Siglecs appears to correlate with longevity, possibly due to their ability to suppress aging‐related inflammation.^1148^

### 9.3 Glycosylation in immunity

A large number of glycoproteins on immune cell surfaces are extensively involved in many immune processes by receiving signals from the extracellular environment.^1149^ Neutrophils are the most abundant innate immune cells and act as the host's first line of defense against pathogen invasion, utilizing bioactive glycoproteins assembled in the cytoplasm to fight pathogenic infections.^1150^, ^1151^ N‐glycosylated and O‐glycosylated proteins exhibit structural and functional diversity in different life stages of neutrophils during bone marrow maturation, blood circulation, and sterilization of inflammatory peripheral tissues.^1152^ Numerous modified granule glycoproteins are present in neutrophils, including neutrophil elastase, myeloperoxidase, and cathepsin G.^1153^ In addition, neutrophils possess unique glycans that are not typically observed, such as hypertruncated chitobiose core‐ and paucimannosidic‐type N‐glycans and monoantennary complex‐type N‐glycans.^1154^, ^1155^ The glycoproteins major histocompatibility complex (MHC) classes I and II play key roles in adaptive immunity. They are closely associated with the presentation of cell surface antigen peptides and circulating T lymphocyte recognition and activation. Glycosylated protein antigens are critical for antigen uptake by cells, proteolysis, antigen presentation by MHC, and activation and initiation of T cells.^1156^, ^1157^ In the adaptive immune system, protein glycosylation also has multifaceted roles in the differentiation of B cells and T cells, cell–cell interactions and the recognition of glycosylated antigens (Figure 19).^1157^, ^1158^, ^1159^ Protein glycosylation has broad impacts on the function of the immune system.

### 9.4 Glycosylation in the gut

There are numerous glycosylation modifications on the surface proteins of IECs that form a physiological barrier to protect the intestinal tract from bacterial infection and invasion. For example, Golgi glycosyltransferases modify mucin in the gut to separate IECs from commensal microorganisms.^1160^ Changes in the glycosylation level of proteins on the surface of IECs can result in the destruction of the mucus layer and the occurrence of intestinal IBD (Figure 19).^1161^ Glycosylation of IEC surface proteins can also alter the structure and function of the microbiota.^1162^ IL‐22‐mediated glycosylation of intestinal cells facilitates the growth of succinate‐consuming Bacillus in the gut microbiome, which reduces the availability of succinate, a key metabolite for Clostridium difficile growth, and prevents Clostridium difficile infection.^1163^

### 9.5 Glycosylation in neurodegenerative diseases

A growing number of studies have shown significant differences in the levels of N‐glycosylation and O‐glycosylation in brains between AD patients and healthy individuals.^1164^, ^1165^, ^1166^, ^1167^, ^1168^ APP is concentrated at the synapses of neurons. After being cleaved by proteases, it produces toxic Aβ protein that accumulates in AD patients.^1169^ N‐glycosylation‐ and O‐glycosylation‐modified APP is found in the cerebrospinal fluid of AD patients.^1170^ The structure of N‐glycans can affect APP transport and Aβ production.^1171^ The modification of APP‐linked N‐glycans by sialylation may affect APP processing, resulting in increased APP secretion and Aβ production.^1172^ In addition, various O‐glycosylation sites have also been identified on APP in human cerebrospinal fluid,^1173^ and O‐glycosylation modification can also increase nonamyloidogenic α‐secretase processing, thus affecting APP processing and reducing Aβ secretion.^1174^ In AD patients, Tau proteins modified by N‐glycosylation and O‐GlcNAcylation can also be detected,^1175^, ^1176^ and the levels of N‐glycosylation in AD patients are higher than those in healthy people,^1177^ which may affect the aggregation of Tau proteins.^1175^ Interestingly, the level of O‐GlcNAcylation is reduced in AD patients.^1178^

TREM2 expressed on myeloid cells is a PD‐related protein that has multiple ligands, including APOE and lipids. By interacting with DNAX activating protein to transduce signals, TREM2 plays an anti‐inflammatory role in various diseases, including PD,^1179^ and can be modified by sialylated and fucosylated complex glycans. N‐glycan changes alter TREM2 conformation and affect the stability and antioxidant capacity of the protein.^1180^ α‐Synuclein can also be modified by O‐GlcNAcylation.^1181^ O‐GlcNAcylation of α‐synuclein affects its phosphorylation and blocks the toxicity of α‐synuclein, suggesting that an increase in O‐GlcNAcylation may prevent α‐synuclein aggregation (Figure 19).^1182^

### 9.6 Glycosylation in viruses

Protein glycosylation is involved in the regulation of host–pathogen interactions and mediates the adhesion, recognition, invasion and immune evasion of pathogens in host cells, which affects pathogen virulence or host cell resistance.^1183^, ^1184^, ^1185^, ^1186^, ^1187^, ^1188^ COVID‐19 caused by SARS‐CoV‐2 is currently a major global health problem.^1189^ Protein glycosylation affects the toxicity and viability of SARS‐CoV‐2, which utilizes its highly glycosylated modified spike (S) protein to interact with the glycosylated host receptor ACE2 and to facilitate SARS‐CoV‐2 invasion of host cells.^1127^

Acquired immune deficiency syndrome caused by human immunodeficiency virus (HIV) seriously endangers human health. On the HIV‐1 envelope (Env), there are approximately 25 and 4 glycosites on each gp120 monomer and gp41 subunit, respectively, and 18–33 glycans on each gp120 monomer. These glycans are dominated by the high mannose type and sialylated complex type.^1190^, ^1191^ which can bind to the chemokine receptors CD4 and DC‐SIGN of host cells or mannose receptors of macrophages and regulate HIV‐1 invasion of host cells (Figure 19).^1192^, ^1193^ Highly glycosylated HIV binds to C‐type lectin receptors (CLRs) of different antigen‐presenting cell subsets, possibly regulating T cell priming and B cell activation.^1194^, ^1195^ N‐glycosylation of gp120 in HIV‐1 is critical for CD4^+^ T cell recognition.^1196^ HIV infection also leads to altered glycosylation of host IgG. The levels of galactosylation in HIV^+^ patients are lower than those in healthy people, and it is more pronounced in the IgG1 subtype.^1197^ Low sialylation of IgG is also found in HIV‐infected patients.^1197^ During the evolution of HIV, the glycans on the HIV‐1 envelope become more complicated under the guidance of natural selection, which also makes HIV more diverse.^1198^

### 9.7 Glycosylation in cancer

O‐GlcNAc of the key cell cycle regulators participates in the processes of cell division, DNA repair, and cell death and is dynamically changed in a cell cycle stage‐dependent manner.^1199^ MUC1, a transmembrane glycoprotein associated with the cell cycle,^1200^ is overexpressed and aberrantly glycosylated in a variety of epithelial cancers and plays an important role in disease progression.^1201^ Dysregulation of glycosyltransferases such as ST6GalNAcI, C1GalT1, and ST3GalI alters the level of O‐glycosylation of MUC1.^1202^, ^1203^, ^1204^ In addition, changes in glycosylation motifs on MUC1 also affect cancer immune surveillance. For example, the binding of sialylated MUC1 to the cell surface lectin CD169 can enhance macrophage activation and promote tumor growth.^1205^

The growth of tumor cells is accompanied by immune escape. There are ligands of programmed death receptors on the surface of tumor cells, which can bind to programmed death receptors (such as PD1) on the surface of T cells, making T cells exhausted and unable to kill tumor cells. Aberrant glycosylation on the surface of tumor cells can alter how the immune system senses tumors and induce immunosuppressive signals. Thus, specific glycans on tumor cells represent a novel immune checkpoint. Aberrant glycosylation on the surface of tumor cells may affect antitumor responses. Changing the level of glycosylated proteins on the surface of tumor cells can enhance the killing effect of CAR‐T cells on solid malignant tumors.^1206^ Moreover, the glycosylation of tumor cell surface proteins also provides new antigen targets for tumor‐specific T cells (Figure 19).^1207^ The most common tumor‐associated glycans include sialylated glycans, Tn antigen and Lewis antigen. The level of sialylation on melanoma cells correlates with the level of tumor growth in vivo, which is associated with the accumulation of Treg cells, reduction of effector T cells, and decreased activity of NK cells.^1208^ Poor survival in patients with stage III colon cancer is associated with BRAF mutation and increased Tn antigen.^1209^ In addition, increased Lewis antigen in the tumor microenvironment can drive innate immune suppression.^1207^

### 9.8 Therapeutic glycosylated proteins

Almost all therapeutic proteins are glycosylated, such as EPO, ENPP1, and IgG antibodies. Carbohydrate components play an important role in the safety and pharmacokinetic properties of these protein‐based drugs.^1210^ Rapid advances in the field of glycobiology have provided more opportunities for the development of glycoprotein therapeutics.^1211^ Technologies are being developed for glycan processing of therapeutic proteins using chemical, chemoenzymatic and genetic approaches in different cell types.^1210^, ^1212^, ^1213^ In addition, engineered cells provide a more powerful tool for developing more complex protein glycan structures and improving the pharmacodynamic properties of therapeutic proteins.^1211^

EPO is an endogenous glycosylated hormone that can stimulate erythropoiesis and has a variety of important physiological functions.^1214^ It can be used to treat anemia caused by CKD and cancer. The recombinant EPO used for clinical treatment can be divided into four types according to their different glycosylation levels. Sialylation and branching N‐glycans on EPO can prolong its half‐life in serum,^1215^, ^1216^ while EPO lacking sialylation exhibits neuroprotective effects in vivo.^1217^ This shows the importance of the type of glycosylation for therapeutic EPO.

Mutations in ENPP1 cause generalized arterial calcification in infancy (GACI), an extremely rare neonatal disease associated with extensive arterial calcification and narrowing. ENPP1 cleaves ATP into PPi and AMP extracellularly. Recombinant ENPP1‐Fc protein has preventive and therapeutic effects on GACI animal models.^1218^ Enhancing the sialylation level of recombinant ENPP1‐Fc protein has a significant effect on prolonging the protein half‐life and improving drug efficacy.^1219^

Glycosylation‐dependent IgGs are therapeutic antibodies for cancers.^1220^, ^1221^ Glycosylation of the IgG Fc region affects the safety and clinical efficacy of therapeutic antibodies. Biantennary complex oligosaccharide modification of Fc at Asn297 is essential for the effector function of antibodies. Fucose and outer arm sugars linked to the core heptasaccharide create structural heterogeneity that also exhibits unique biological activities.^1222^ Clarifying the glycosylation profile of Fc is the key to the development and quality control of therapeutic antibodies.

Glycosylated vaccines also have broad application prospects. For example, changes in the glycans of the glycoprotein gp120 expressed on the HIV‐1 envelope may lead to immune escape of the virus.^1223^ In the process of vaccine design, adding a new glycosylated epitope on recombinant gp120 is beneficial to improve the ability of neutralizing monoclonal antibodies to recognize HIV‐1, thereby optimizing the design of viral vaccines.

## 10 CITRULLINATION

**FIGURE 20:**
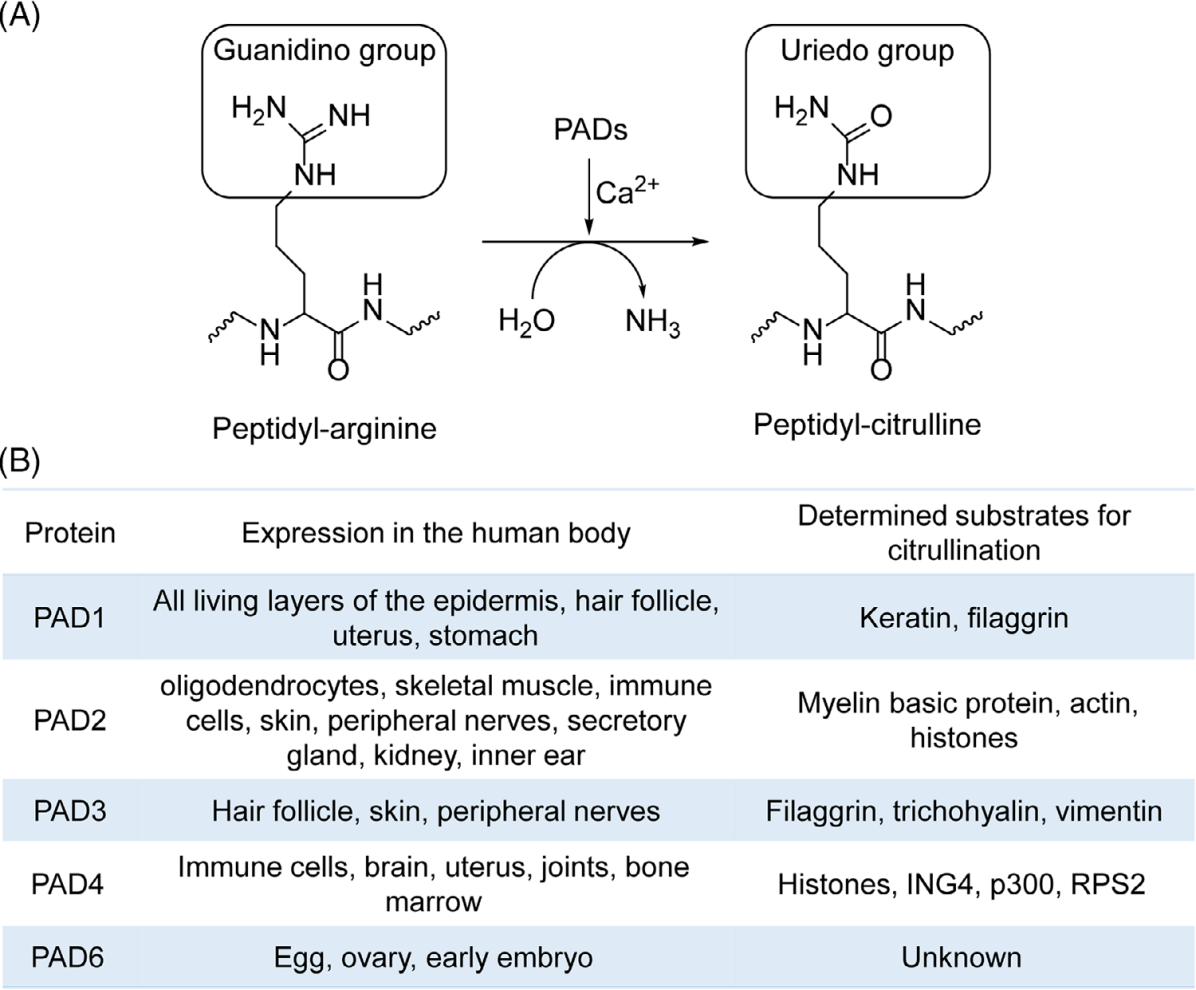
The schematic reaction for citrullination regulated by PADs. (A) Cartoon depicting PAD‐mediated conversion of arginine to citrulline. (B) Table listing the tissue distribution of PADs and their representative citrullination substrates.

Citrullination refers to the irreversible process of converting arginine residues in proteins into citrulline residues under the action of protein arginine deiminases (PADs) (Figure 20A).^1224^ The conversion of positively charged arginine to charged citrulline affects hydrogen bond formation, protein folding, hydrophobicity, and protein interactions, ultimately leading to protein denaturation.^1225^, ^1226^ For example, the change of 5% of arginine residues into citrullination in hyalin or fibroin can affect its tertiary structure, and the citrullination of more than 10% of arginine residues may completely destroy the protein structure and denature it.^1227^ PAD includes five isozymes (PAD1–4 and PAD6) with tissue specificities (Figure 20B). PADs 1–4 are catalytically active, while PAD6 is not because of active site mutations.^1228^ The most common citrullination substrates of PADs are keratin, fibroin, vimentin, actin, histones, collagen, and myelin basic protein (Figure 20B).^1229^, ^1230^ Notably, free arginine cannot be citrullinated by PADs.^1228^ PADs have a high degree of sequence homology but differ in their tissue distribution. Both PAD1 and PAD3 are mainly in the hair follicle and epidermis, and PAD1 is also present in the uterus.^1231^, ^1232^ PAD2 and PAD4 are widely distributed throughout tissues. For example, PAD2 is expressed in skeletal muscle, brain, spleen, and secretory glands and is the most widely expressed PAD in the human body.^1233^, ^1234^ There are two main substrates of PAD2: myelin basic protein in the CNS and glial fibrillary acidic protein (GFAP). Citrullination of the former is involved in the pathogenesis of multiple sclerosis (MS), while the latter may be associated with senile dementia.^1235^ In contrast, PAD4 is present in neutrophils, macrophages, mammary cells, and tumor cells.^1236^, ^1237^, ^1238^ PAD4 mainly catalyzes histones,^1239^ whose citrullination not only disrupts their structures but also causes them to lose many positive charges, thereby depolymerizing nucleosomes, breaking DNA, and ultimately leading to apoptosis. Notably, PAD4 is involved in histone citrullination in neutrophils. After translocation of activated PAD4 to the nucleus of neutrophils, neutrophil extracellular traps (NETs) are produced to trap bacteria and other pathogens.^1240^, ^1241^ Histone modifications control NETosis, which is linked to the development of autoimmune diseases such as RA, ulcerative colitis, and SLE.^1242^ PAD6 was originally discovered through sequence alignment and is located in early embryos, eggs, and ovaries.^1238^, ^1243^ PAD6 expression is correlated with the degree of citrullination in female germ cells despite lacking catalytic activity,^1244^ and its function may be involved in early embryonic development.^1245^

PAD‐mediated citrullination is regulated by various factors, such as Ca^2+^ concentration and amino acid sequence^1246^. The intracellular Ca^2+^ concentration is maintained at low levels to keep PAD inactive under physiological conditions.^1247^ However, certain PAD‐mediated processes are operated under physiological Ca^2+^ concentrations, implying other unknown mechanisms of PAD action.^1248^, ^1249^ Apoptosis is dependent on high intracellular Ca^2+^ concentrations,^1250^, ^1251^ and protein citrullination is increased in apoptotic cells.^1247^

Protein citrullination has diverse biological functions. For example, citrullination is involved in cell apoptosis, which may be related to cell morphological changes and DNA fragmentation during apoptosis. The elevation of Ca^2+^ is involved in the early signal transduction and execution stage of apoptosis.^1265^, ^1266^ PAD2‐mediated vimentin citrullination may result in changes in cell morphology.^1247^ Activated nuclear PAD4 induces nonspecific citrullination of histones, which disrupts protein structure, depolymerizes nucleosomes, and makes DNA more susceptible to nuclease cleavage, ultimately leading to apoptosis.^1252^ Citrullination can promote terminal differentiation of cells, and the expression of keratin changes as the epidermis progresses to terminal differentiation. Keratin 1, keratin 10, keratin 5, and keratin 14 are four major keratins expressed in the human epidermis. Keratin citrullination in the epidermis promotes terminal differentiation of keratinocytes.^1244^ Citrullination can regulate gene expression.^1253^ PAD2‐mediated citrullination of histone H3 is associated with the regulation of the expression of more than 200 ER‐related genes, such as *HER2*.^1254^ Moreover, *PAD2* expression is significantly higher in blood and tissues from breast cancer patients than in those from normal controls.^1255^ When *PAD2* is inhibited, the expression of *ACSL4* and baculovirus‐containing IAP repeats is reduced in the breast cancer cell line MCF‐7.^1256^ Abnormal expression of these genes is related to dysregulated lipid metabolism and tumor cell invasion.^1257^

### 10.1 Citrullination in immune diseases

**FIGURE 21:**
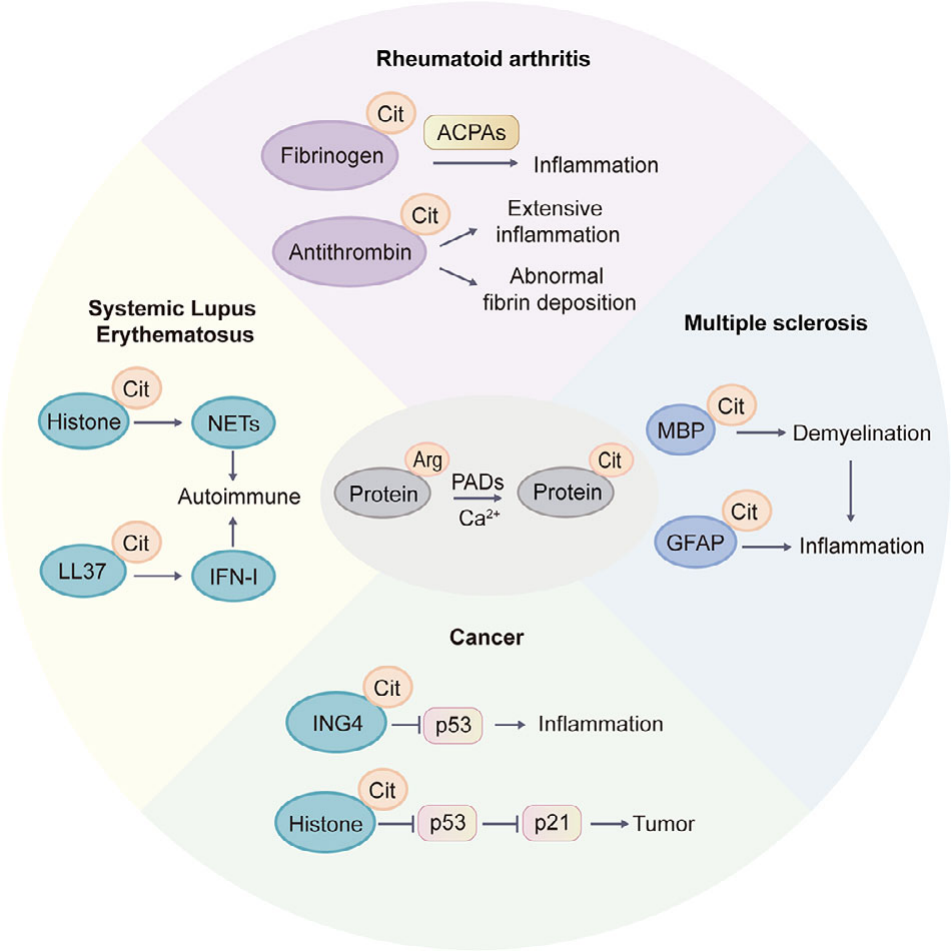
Representative citrullination substrates and their functions in various diseases, such as RA, cancer, MS, and SLE.

Inflammation, chronic pain, and polyarthritis are the main features of RA.^1258^ A growing amount of evidence shows that the autoimmune reaction to RA is driven by dysregulation of protein citrullination mediated by PAD4, as 75% of patients have anticitrullinated protein antibodies (ACPAs).^1239^ ACPAs are the most specific autoantibodies in RA serum.^1259^ Most of these autoantibodies can be detected early in the plasma, making them useful diagnostic markers for RA.^1260^, ^1261^ In inflammatory synovial tissues, macrophages and neutrophils express more PAD2 and PAD4, respectively. Both PAD2 and PAD4 are released into joint citrullinated proteins, such as fibrin, fibrinogen and vimentin,^175^ which further initiate immune responses and induce autoantibodies (Figure 21).^1262^, ^1263^ Blood coagulation factor citrullination is also important in RA.^1264^ Thrombin can generate inflammatory mediators that promote inflammation, resulting in excessive capillary formation and fibrin deposition in synovial tissues (Figure 21). PAD4‐mediated citrullination of antithrombin inhibits thrombin activity, resulting in an increase in the coagulation rate.^1265^ The levels of citrullinated antithrombin are significantly elevated in RA patients compared to healthy controls.^1229^ Inhibiting hyperactivated PAD enzymes to reduce citrullination is a promising therapeutic strategy for RA patients.^1266^

MS is characterized by inflammatory demyelinating lesions of the CNS. The pathogenesis of MS is complex and is currently thought to be caused by a combination of genetic and environmental factors.^1264^ MS is primarily associated with PAD2‐mediated over‐citrullination of myelin basic protein and GFAP, leading to demyelination and affecting nerve signal transduction (Figure 21).^1267^, ^1268^ Overexpression of PAD2 in transgenic mice increases the amount of citrullinated myelin basic protein and accelerates demyelinating changes.^1269^ Citrullinated myelin basic protein alters its processing and presentation by T cells. Despite the presence of T cells specific for citrullinated myelin peptides, no autoantibodies against citrullinated proteins have been found in the serum of MS patients.^1270^ Furthermore, the upregulation of PAD2 and inflammatory signaling may locally increase PAD4 to further aggravate inflammatory disease. Collectively, myelin basic protein citrullination mediated by PAD2 and PAD4 promotes proteolysis, demyelination, and signaling blockade, ultimately leading to MS.

SLE is manifested by the immune system attacking healthy cells and tissues throughout the body. SLE immune system activation is characterized by B cell and T cell hyperreactivity and loss of immune tolerance to self‐antigens.^1271^ Autoantigens in SLE patients mainly come from apoptosis and the formation of NETs, and citrullination is involved in these processes and affects the occurrence and development of SLE. For example, LL37 binds to self‐DNA/RNA and stimulates plasmacytoid dendritic cells to produce IFN‐I, leading to an autoimmune response. In the skin and kidney of SLE, citrullinated LL37 (cit‐LL37) is significantly increased, and LL37‐specific T cells show a significant response to cit‐LL37 (Figure 21).^1272^ Many NETs‐related proteins are posttranslationally modified, especially histones found to be methylated, acetylated, and citrullinated, indicating that NETs may be a source of self‐antigens in autoimmune diseases (Figure 21).^1273^ Aberrant apoptotic pathways prevent immune cell clearance, which prolongs the exposure of self‐antigens and induces the production of autoantibodies.^1264^, ^1274^ Among the autoantibodies associated with SLE, many antigens, including nuclear DNA and proteins, can be detected in NETs.^1283^, ^1293^

### 10.2 Citrullination in cancers

p53 is a well‐known tumor suppressor and TF.^1275^ Based on pathological studies of patient samples, PAD4 is highly expressed in a variety of tumors, including colon cancer, esophageal cancer, ovarian cancer, PC, and gastric cancer,^1237^, ^1276^ suggesting possible involvement of PAD4 in tumorigenesis. PAD4 expression is regulated by p53. PAD4 citrullinates the growth inhibitor ING4, which subsequently prevents the binding of p53 to ING4 to inhibit p53 expression, further inhibiting downstream p21 expression and promoting tumor growth (Figure 21). Notably, PAD4 forms negative feedback to regulate p53 through histone citrullination.^1277^ PAD inhibitors may also prevent the expression of genes related to cancer cell invasion and metastasis.^1256^

### 10.3 Citrullination in inflammatory diseases

Ulcerative colitis is a chronic inflammatory disease occurring in the colonic mucosa. Ulcerative colitis patients often suffer from serious complications, the most common of which is peripheral arthropathy.^1278^ Immunohistochemical analysis shows increased PAD2 and PAD4 in damaged tissues in ulcerative colitis patients.^1279^ The upregulation of PAD4 is associated with neutrophils and NETs in the colonic mucosa of ulcerative colitis patients, even in remission.^1280^ Proteins that promote NETs formation are potential therapeutic targets to reduce inflammation in ulcerative colitis.^1281^ The severity of acute colitis in PAD4‐deficient mice with considerable rectal bleeding is evidence that PAD4 is essential for maintaining colitis mucosal homeostasis and regulating rectal bleeding.^1282^

## 11 CARBAMYLATION

Protein carbamylation is a nonenzymatic modification mediated by cyanate. Cyanate reacts with the amino groups of proteins to form carbamylation.^1283^ Spontaneous carbamylation can alter the molecular weight, isoelectric point, and other physical properties of the protein and can also lead to an irreversible decrease in protein activity.^1284^ In theory, all proteins in the human body are prone to carbamylation. However, the probability of carbamylation of each protein depends on the number and activity of amino groups and the lifetime of the protein. Protein carbamylation preferentially occurs at the side chains of lysine residues, also known as homocitrullination.^1285^

**FIGURE 22:**
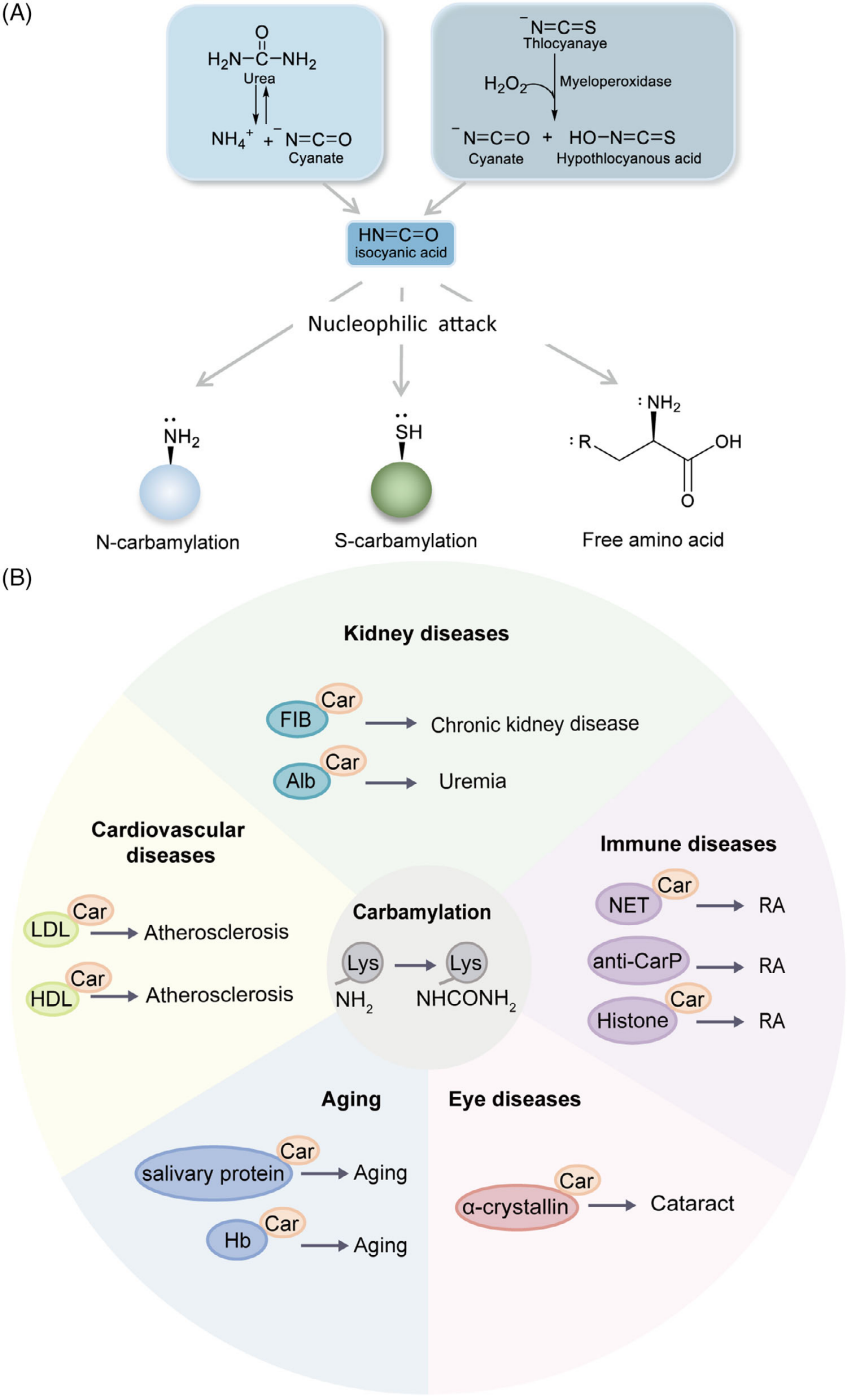
The different pathways of carbamylation and its roles in several diseases. (A) There are two main pathways for the production of isocyanic acid for carbamylation. (B) Representative protein substrates and roles of carbamylation in various diseases.

There are two main pathways for the formation of cyanate (Figure 22A). First, urea is a product of protein catabolism that decomposes slowly and spontaneously in aqueous solution to form cyanic acid and cyanate.^1286^ Under normal physiological conditions, the cyanate concentrations in body fluids are also extremely low and do not cause extensive carbamylation in the body.^1287^ Second, under certain inflammatory conditions, MPO alters the balance between cyanate and thiocyanate, causing an increase in cyanate synthesis and leading to carbamylation in the body.^1288^ In addition, smoking also increases the concentration of cyanate and carbamylation in the body, which leads to the occurrence of diseases.^1289^, ^1290^

Protein carbamylation can alter protein structures, PPIs, and protein–cell interactions. By removing the positive charges on proteins, carbamylation can alter the protein's interaction with water and disrupt ionic interactions on the protein's surface. As these interactions are able to stabilize the secondary and tertiary structures of proteins, their absence may result in dramatic changes in protein conformation.^1291^ Changes in protein structure will affect its functions and biological activities and cause diseases in the body. Protein carbamylation alters the native structure of plasma proteins, which participates in the pathogenesis of diabetes.^1292^ In addition, there are also proteins that acquire new functions after carbamylation. Carbamylation of LL‐37 reduces its bactericidal properties, and converts anti‐inflammatory LL‐37 to proinflammatory LL‐37.^1293^ Moreover, carbamylation affects the assembly of homologous or heterologous protein monomers into fibers or filaments.^1294^ For example, actin or collagen cannot form intact filaments or fibers after carbamylation.^1295^

### 11.1 Carbamylation in aging

Studies have found that various physiological and pathological processes are related to protein carbamylation, such as aging, cataracts, CKD, atherosclerosis, RA, and neurological diseases (Figure 22B).^1283^ By measuring the changes in homocitrulline, a typical carbamylation derivative product (CDP), with age, carbamylation occurs throughout the life cycle and contributes to carbamylated protein accumulation in organs. The accumulation rate of CDPs is negatively correlated with lifespan, suggesting that longer‐lived species may have efficient turnover, repair, or degradation systems to limit carbamylated protein accumulation in organs.^1296^ Modifications of salivary proteins increase with age, as evidenced by decreased total thiol levels and increased carbamylated proteins in the saliva of older adults.^1297^ Therefore, protein carbamylation may serve as a marker of mammalian aging.^1285^ In addition, protein carbamylation in peripheral blood is associated with age‐related oxidative damage.^1298^ In elderly cataract patients, crystallins, including α‐, β‐, and γ‐crystallin, can be modified by carbamylation, with seven lysine residues modified in α‐crystallin.^1299^ An important function of α‐crystallin in the lens is to ensure the activity of its chaperones, which limit protein aggregation and thus keep the lens transparent. However, α‐crystallin carbamylation significantly affects its chaperone activity.^1300^

### 11.2 Carbamylation in kidney diseases

Carbamylated protein levels have been found to be significantly elevated in CKD (Figure 22B), and carbamylated albumin is considered an important biomarker for risk of death.^1301^ Carbamylated proteins may cause CKD complications such as atherosclerosis. Higher levels of carbamylated proteins are detected in the plasma of 75% of kidney‐removed CKD mice.^1302^ C‐Alb carbamylation levels are associated with higher mortality in diabetic patients with ESRD.^1303^ Carbamylated HDL but not carbamylated LDL in plasma is independently related to CKD progression in T2DM patients.^1304^ By investigating the levels of fibrinogen carbamylation in patients with renal disease and the effect of carbamylation on thrombin fibrinogen cleavage, fibrin polymerization and in vitro crosslinking, it has been found that although carbamylation itself does not affect thrombin cleavage, it alters fibrin polymerization kinetics and impairs cross‐linking and clot degradation.^1295^

### 11.3 Carbamylation in atherosclerosis

Protein carbamylation is closely associated with atherosclerosis (Figure 22B).^1305^ “Uremic dyslipidemia” in CKD patients is characterized by normal low‐density lipoprotein cholesterol, low high‐density lipoprotein cholesterol, and high triglyceride plasma levels.^1306^ cLDL induces the prothrombotic effects of vascular cells and platelets by activating LOX‐1 receptors and promotes thrombus formation.^1307^ An increased incidence of acute thrombosis has been observed in patients with CKD. In addition, the slight carbamylation of LDL leads to a decrease in the uptake of LDL by fibroblasts, which results in a lower clearance rate and prolongs the residence time of these particles in the blood circulation. This consequently increases the chance of further carbamylation of LDL. High cLDL leads to the accumulation of cholesteryl esters in macrophages.^1308^, ^1309^ Meanwhile, cLDL causes damage to vascular endothelial cells and induces abnormal proliferation of VSMCs, eventually leading to atherosclerosis.^1310^ Compared with cLDL, HDL has antiatherosclerotic properties and protective effects on the heart. However, carbamylation of HDL is involved in the occurrence and development of atherosclerotic CVD.^1311^ Carbamylated HDL engages in the formation of foam cells and becomes proatherosclerotic lipoproteins.^1312^ In addition, after carbamylation, vascular elastin fiber morphology and susceptibility to elastase degradation remain unchanged, but elastic fiber stiffness increases. These changes in the mechanical properties of the vascular wall may lead to aortic stiffness.^1313^

### 11.4 Carbamylation in immune diseases

In addition to ACPAs,^1314^ autoantibodies against carbamylated proteins (anti‐CarP) have been detected in the serum of RA patients (Figure 22B).^1315^ Anti‐CarP has important implications in the pathophysiology of RA and can be used to assess the risk level of RA patients. RA patients also develop autoantibodies against carbamylated NET (cNET) antigens, and the levels of these antibodies correlate with anti‐CarP levels, making them a new biomarker for RA.^1316^, ^1317^ In addition, carbamylated histone‐IgG immune complexes can promote osteoclast differentiation and enhance the matrix resorption of osteoclasts, suggesting that carbamylated proteins in NETs can increase pathogenic immune responses and bone destruction. This explains the link between anti‐CarP levels and erosive arthritis in RA patients.^1317^

### 11.5 Carbamylation in neuropathy

Carbamylation has also been related to neuropathy (Figure 22B). The higher the level of carbamylation in the rat brain, the more severe the memory loss.^1283^ Carbamylation has also been implicated in AD,^1318^ and prevention of carbamylation may protect against cyanate‐induced neuropathy.^1319^ Collectively, protein carbamylation is a potential biomarker for various human diseases and has important clinical significance.

## 12 REDOX MODIFICATIONS

**FIGURE 23:**
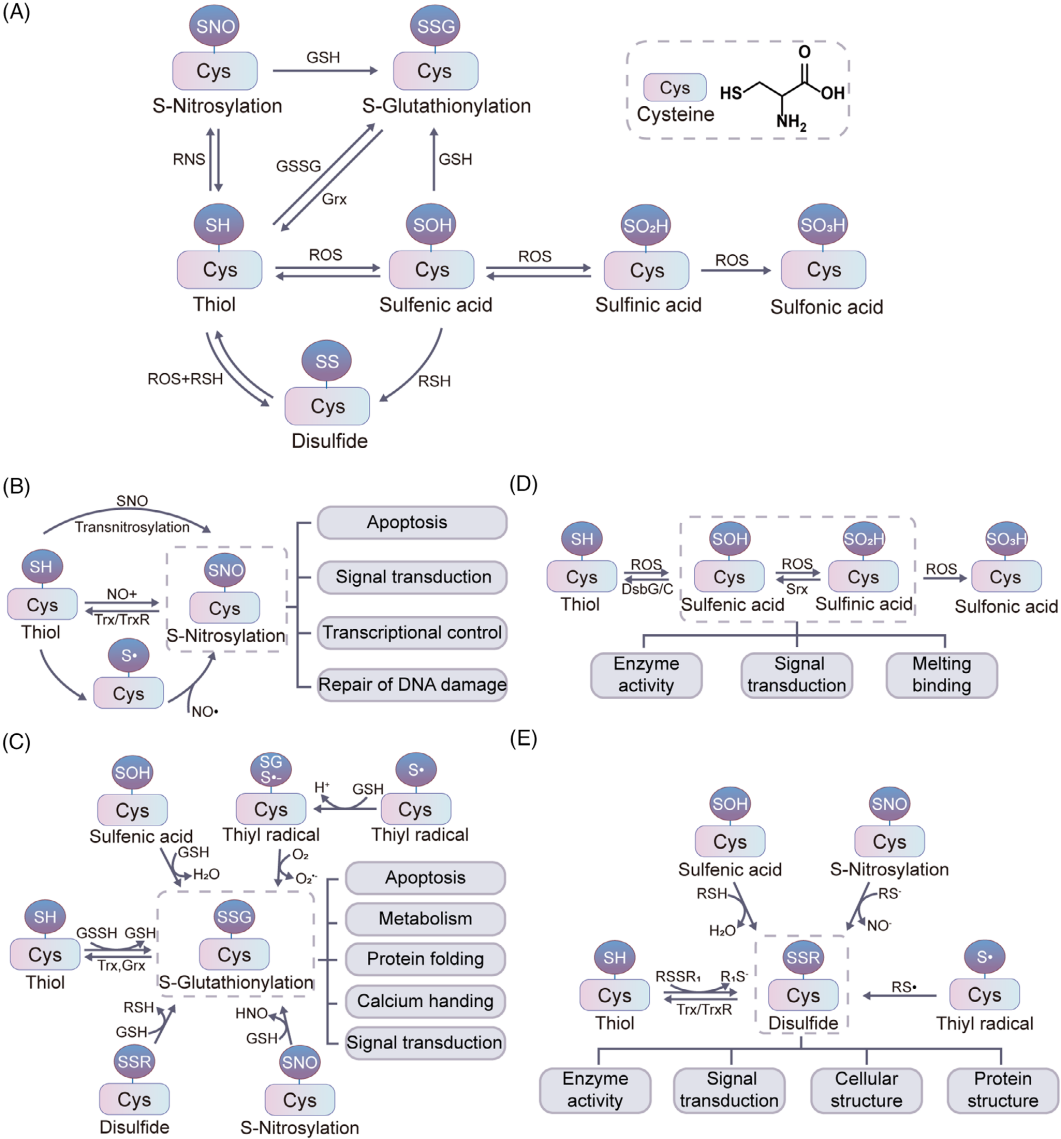
Redox modifications of cysteine. (A) Different‐types‐of‐redox modifications of cysteine. (B–E) The mechanisms of different types of redox modifications and associated physiological functions.

As a main element of life, sulfur has multiple oxidation states (from −2 to +6) and participates in various redox reactions. Cysteine is a sulfur‐containing amino acid in proteins, and its sulfur atom provides a wide range of chemical reactivity and structural flexibility for proteins.^1320^ Although the theoretical abundance of cysteine distribution in the human proteome is only 3.3%, as much as 22% of protein active sites are formed.^1321^ Redox is the main pathway for the regulation of cysteine functions, with two main classes of drivers.^1322^ The first is ROS/RNS/RSS generated by endogenous metabolism or exogenous stimuli, which mediate various redox modifications on cysteine, such as the oxidation of sulfhydryl (‐SH) on the side chain of cysteine to sulfenylation (‐SOH), which not only participates in the formation of intramolecular or intermolecular disulfide bonds (‐SS‐) or undergoes S‐glutathionylation (‐SSG) but can also be further oxidized to generate sulfinylation (‐SO_2_H) and sulfonylation (‐SO_3_H) modifications. The second driver is the complex and diverse reductase system in cells, which can reduce modifications other than sulfonylation. For instance, glutathionylation can be reduced by GRX/GST,^1323^ and sulfenylation can be specifically reduced by two isomerases, DsbG/C.^1324^ Currently, a number of oxidative PTMs (oxPTMs) on the thiols of cysteines, including SNO, sulfenylation, sulfinylation, sulfonylation, S‐glutathionylation, and disulfide bonds, have been described (Figure 23A).

### 12.1 S‐Nitrosylation

The formation of SNO is a reversible nonenzymatic catalyzed reaction in which NO is covalently bound to the thiols of cysteines to form SNO.^1325^ In general, there are three pathways for the synthesis of SNO (Figure 23B). NO reacts directly with the thiol group of the cysteine through NO^+^,^1326^ the SNO‐modified small molecule or the proximal SNO‐modified protein provides NO^+^,^1327^ or the thiol of cysteine can be activated to form a sulfur radical (S•), which then reacts with a NO radical (NO•) to generate SNO.^1326^ S‐nitrosothiols are biologically stable reservoirs of NO,^1328^ have selective and transient modification properties and are excellent signal sensors.^1329^ For each protein, SNO can occur at a single cysteine or multiple cysteines.^1330^ There are more than 3000 proteins that are regulated by SNO and are involved in the regulation of protein stability, DNA damage repair, transcriptional regulation, cell growth, differentiation, and apoptosis (Figure 23B).^1330^ In addition, SNO also regulates the protein conformation of overlapping or nonoverlapping residues, PPIs, and other PTMs (e.g., phosphorylation, acetylation, ubiquitination, and disulfide linkage).^1331^ Dysregulation of SNO contributes to many diseases.^1332^, ^1333^, ^1334^

### 12.2 S‐Glutathionylation

Glutathione is covalently bound to reactive cysteines in proteins through disulfide bonds, termed S‐glutathionylation.^1335^ S‐Glutathionylation can occur via nucleophilic sulfur, where the thioanion (S‐) reacts with oxidized glutathione (GSSG), or via the reaction between GSH and electrophilic sulfur intermediates such as sulfenic acid, S‐nitrosothiol, and thiol radical (Figure 23C).^1336^ S‐glutathionylation is involved in redox signaling and protects the thiols of cysteines from irreversible oxidation during oxidative stress. Many enzymes are known to catalyze glutathionylation/deglutathionylation reactions, including GSTP and GRX.^1335^ GSTP possesses both molecular chaperone and catalytic properties and controls the redox balance in the oxidative endoplasmic reticulum. It catalyzes the S‐glutathionylation of target proteins and impacts the function of unfolded proteins.^1337^ GRX plays a crucial role in removing S‐glutathionylation, preserving cellular redox homeostasis by regulating the S‐glutathionylation of essential proteins such as phosphatases, kinases, and TFs.^1338^ Therefore, S‐glutathionylation balance serves as a feature of normal cellular redox homeostasis.^1335^ Moreover, S‐glutathionylation also regulates the activities of mitochondrial enzymes, heat shock proteins, TFs, and cytoskeletal proteins.^1339^ Hsp90 is a widely distributed molecular chaperone that interacts with a variety of proteins and regulates a variety of cellular processes. S‐glutathionylation of Hsp90 results in inactivation of ATPase.^1340^ S‐glutathionylation of C/EBPβ stabilizes the protein and increases its levels, promoting 3T3l1 cell differentiation.^1341^ Additionally, S‐glutathionylation plays a role in regulating apoptosis. For example, S‐glutathionylation of GADPH may transmit signals to the nucleus where GADPH trans‐glutathionylates nuclear proteins such as Sirt1 to trigger apoptosis. GRX removes S‐glutathionylation of GAPDH and prevents its nuclear transport.^1342^ FASL‐induced activation of airway epithelial cell apoptosis is accompanied by an increase in protein S‐glutathionylation.^1343^

### 12.3 Sulfenylation

Cysteine thiols are oxidized by hydrogen peroxide to produce sulfenic acids. Sulfenylation is a nonenzymatic modification that can also be converted from other oxidized forms of cysteine, such as SNO (Figure 23D).^1326^ Sulfenic acids have long been considered transient reaction intermediates formed by cysteine thiols under oxidative stress.^1344^ However, they have been discovered to play much more significant roles in cellular biology. These sulfenic acids serve not only as indicators of oxidation‐sensitive cysteines and intermediate oxidation states, but also as key regulators of protein function, signal transduction, and initiators of disulfide bond formation.^1345^, ^1346^ For example, the formation of sulfenic acid is associated with hydrogen peroxide‐mediated inactivation of PTPs, and the oxidation of cysteine thiol affects cellular PTP activity.^1344^ Sulfenylation increases the kinase activity of EGFR by oxidizing the active site Cys797.^1347^ The activity of Src is regulated by a redox‐dependent mechanism, in which sulfenylation at Cys185 and Cys277 can enhance its activity.^1348^ Platelet CD36 signaling can promote hydrogen peroxide‐mediated sulfenylation of Src family kinases, which is critical for oxLDL/CD36 proaggregation and procoagulant functions.^1349^ UCP1 is a protein required for thermogenesis in adipose tissue. Cys253 of UCP1 is sulfenylated during thermogenesis and affects sensitivity to UCP1‐dependent thermogenesis.^1350^ The redox state of a single cysteine can alter biological processes in response to changes in cellular redox homeostasis.^1351^ For example, ROS‐induced sulfonylation of C663 inhibits the UPR and stimulates the antioxidant response mediated by p38 MAPK signaling.^1352^ Reversible sulfenylation can also regulate the enzymatic activity of transcription and transduction factors, such as Mfn2, which undergoes sulfenylation after inflammatory stimulation and negatively regulates the transcriptional activity of β‐catenin.^1353^ In addition, protein sulfenylation can also directly or indirectly affect many PTMs in cells, such as the oxidation of specific cysteines in PTPs, PTK, and cysteine proteases.^1354^

### 12.4 Sulfinylation and sulfonylation

Cysteine sulfinic and sulfonic acids are the peroxidation products of cysteine.^1345^ In the presence of excess oxidants, sulfenic acid can be oxidized to sulfinic acids or even sulfonic acids (Figure 23D). Sulfonic acids are the most oxidized form of cysteine. Although sulfinic acid was once thought to be an irreversible oxidative modification, the oxidation of the active site cysteine of Prx I or Prx II to sulfinic acids is reversible.^1355^ With the discovery of sulfiredoxin,^1356^, ^1357^ the regulation of sulfinylation in biology has also received attention. By using electrophilic diazene probes (DiaAlk), hundreds of previously unreported protein sulfinylation sites have been identified in mammalian cells.^1358^ Prx is an important class of human antioxidant enzymes that are present in high concentrations in human cells such as erythrocytes^1359^ and can rapidly oxidize sulfenic to sulfinic acids.^1360^ Under normal conditions, Prx utilizes the thiol/sulfenic acid oxidative cycle to detoxify hydrogen peroxide. However, under conditions of oxidative stress, the hydrogen peroxide concentration exceeds the reducing power of Prx, resulting in the formation of sulfinic acids. Cysteine residues in Prx sense the intracellular hydrogen peroxide concentration.^1360^ DJ‐1 is a protein associated with hereditary Parkinson's syndrome. Sulfinylation at Cys106 regulates the protective function of DJ‐1.^1361^ Cysteine residues usually exist in metal‐binding motifs and form coordinate bonds with metal ions, such as zinc, copper, and iron. However, sulfinylation of these proteins can lead to the release of zinc ions and changes in protein conformation, which in turn alter protein function.^1362^

### 12.5 Disulfides

Disulfide bonds in proteins are a widespread cysteine modification that plays an important role in protein folding and stability. Disulfide bonds form intermolecularly or intramolecularly, depending on the accessibility and proximity to other cysteine groups. Disulfide bonds can be produced intracellularly through thiol‐disulfide exchange, coupling between thiol radicals, and thiol reaction with nitrosylated cysteine or sulfenic acids (Figure 23E).^1345^ Thiol‐disulfide exchange can be achieved by nonenzymatic or enzymatic reactions, such as Trx, Grx, and PDI, which can accelerate disulfide bond formation.^1345^ Disulfide bonds can introduce conformational constraints in peptides and proteins. Peptides containing disulfide bonds are expected to be used as drug leads or scaffold materials.^1363^ Hinge disulfide bonds in the human IgG2 CD40 antibody regulate receptor signaling by modulating conformation and flexibility.^1364^ Kinases are now also thought to be regulated by redox. The formation of intermolecular disulfide bonds between homodimers activates PKG1α and ATM, while intermolecular disulfide bonds between Src monomers inhibit kinase activity.^1365^ MLL1 is a redox‐regulated HMT, and peroxide‐induced intramolecular disulfide bond formation results in inactivation of the HMT SET‐1/MLL1.^1366^ The protease activity of human ATG4B is also affected by reversible redox modification. A previous study found that C292 and C361 of ATG4B form a disulfide bond, which affects autophagy by regulating the activity of ATG4B.^1367^ Disulfide bonds also affect the subcellular localization of proteins, PPIs,^1368^ and the activity of cytokines. HMGB1 is a nuclear protein with extracellular inflammatory cytokine activity. HMGB1 requires mild oxidation to form a C23–C45 disulfide bond and unoxidized C106 to induce phosphorylation of the NF‐κB p65 subunit and TNF‐α production.^1369^ Oxidative stress induces the intermolecular disulfide bond formation of TFEB/TFE3 in mammals, which enhances the activity of the TF.^1370^ STING is a key regulator in the innate immune type I IFN pathway, and its C206 oxidation to form intermolecular disulfide bonds leads to a conformational change in the protein that prevents excessive activation of STING.^1371^ In addition, cellular structure is also dependent on cysteine disulfides, as microtubule assembly is partly mediated by disulfide bonds.^1345^

### 12.6 Redox modifications in health and diseases

**FIGURE 24:**
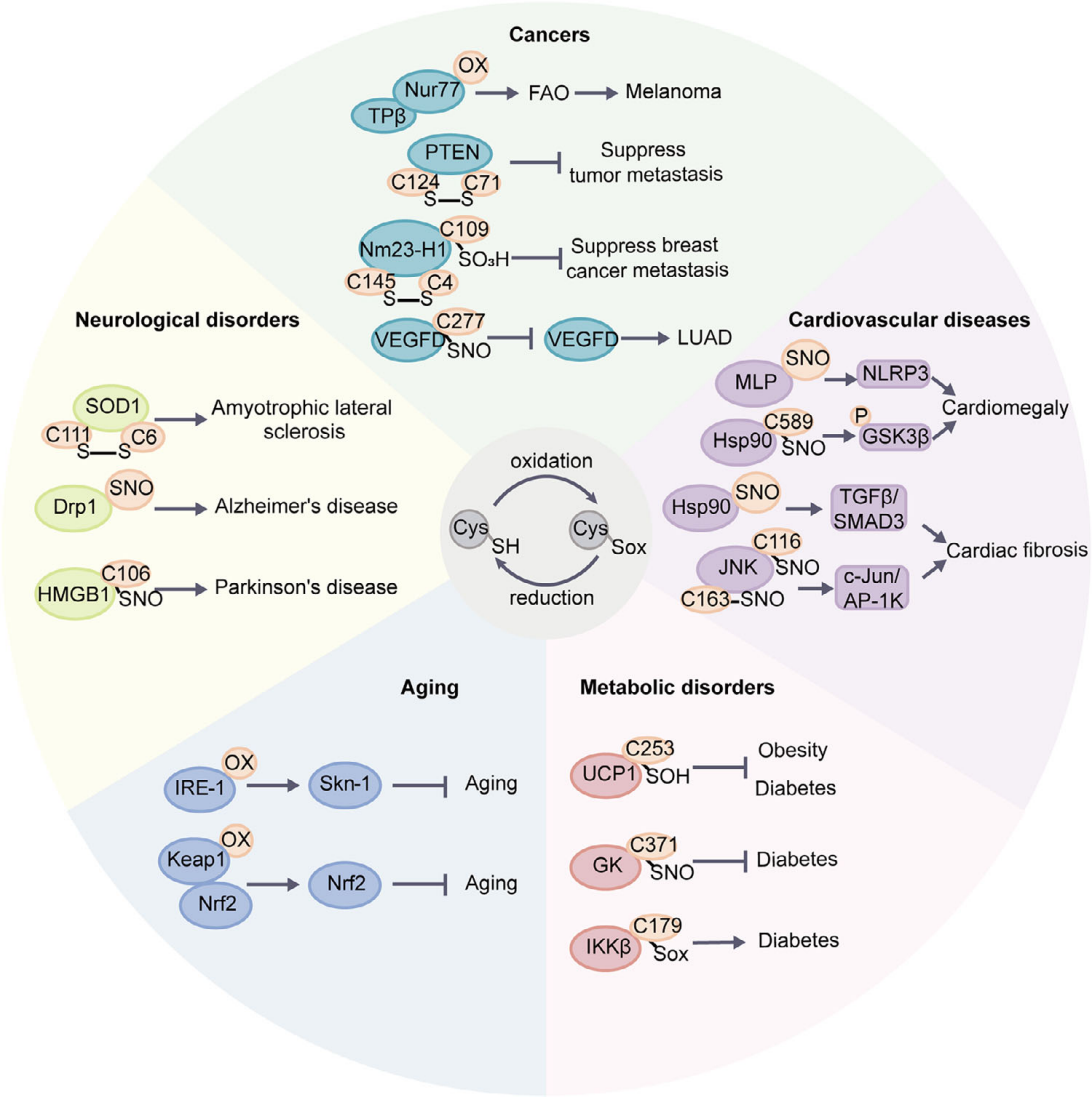
Representative redox modification events in aging, metabolic disorders, neurological disorders, cancers, and CVDs are shown.

Redox‐associated PTMs are a part of normal cell signaling and can regulate the activity of a wide variety of proteins involved in energy metabolism, protein folding and degradation, and gene transcription. Imbalances in redox homeostasis are associated with aging^1372^ and various diseases, such as neurodegenerative diseases,^1373^ CVDs,^1374^ and cancers (Figure 24).^1334^

### 12.7 Redox modifications in aging

Aging‐caused imbalance between RONS production and cellular antioxidant capacity may lead to oxidative stress. Proteins aberrantly modified by redox modifications become dysfunctional.^1372^ Protein cysteine redoxomics in various tissues of young and old mice has shown that many redox modifications in young tissues disappear in old tissues, but some new redox modifications also appear in old tissues.^1375^ Supplementation with antioxidants can neutralize ROS, and glutathione and its precursor N‐acetylcysteine (NAC) are common dietary supplements. However, long‐term use of GSH and NAC inhibits skn‐1‐mediated gene transcription and accelerates aging.^1376^ Endoplasmic reticulum sulfhydryl oxidase Ero1α produces SNO to reduce its activity, leading to reduced stress in the ER and compromised ER proteostasis and UPR, which in turn promotes cell senescence.^1377^ The KEAP1–NRF2 system is a key defense mechanism to prevent oxidative stress and aging.^1378^

Sarcopenia is a hallmark of human aging.^1379^ Muscle wasting is accompanied by decreased oxygen consumption and increased ROS production in sarcopenia.^1380^ The restoration of redox balance by elamipretide (SS‐31) in aged mice can enhance mitochondrial function and improve skeletal muscle function. S‐glutathionylation has been significantly reversed in aged mice, and the gastrocnemius muscle of SS‐31‐treated mice has greater fatigue resistance and mass.^1381^ S‐glutathionylation is significantly increased in mitochondrial complexes I, II and V, ACO2, GAPDH, and MDH1 after rest and fatigue contraction, indicating that redox plays important roles in the control of muscle physiology, metabolism, and exercise adaptation response.^1382^

### 12.8 Redox modifications in metabolic disorders

The production and metabolism of active substances and the recovery and removal of the antioxidant system are highly synchronized processes in normal physiological conditions. When these systems are disturbed, a series of metabolic diseases, such as obesity, metabolic syndrome, and T2DM, can occur (Figure 24).^1383^, ^1384^ Excessive production of ROS in vivo can lead to oxidation of proteins, leading to decreased insulin secretion and increased insulin resistance, and contributing to the development of diabetes.^1385^ For example, ROS‐induced oxidative stress can activate the IKKβ/NF‐kβ pathway, leading to pancreatic β‐cell dysfunction. Cys179 of IKKβ may be a vulnerable site to redox modification.^1386^ Oxidative modification of cysteine residues in Keap1 also affects the development of diabetes by regulating the Nrf2/Keap1/ARE pathway.^1385^

Increased intracellular NO production and reduced bioavailability are key factors leading to imbalances in redox homeostasis in metabolic diseases.^1387^ Protein SNO is important to the entire process of insulin action, including processing and secretion by pancreatic β cells, transport by endothelial cells, signal transduction, and degradation of insulin.^1388^, ^1389^ For example, glucokinase SNO at Cys371 dissociates glucokinase from ISG and facilitates its transition to the active conformation to increase insulin secretion.^1390^

Obesity is a metabolic disorder affected by oxidative stress.^1391^, ^1392^ SNO of IRE1α in obese mice leads to a decrease in glucose homeostasis.^1393^ ROS can activate thermogenesis through direct redox modification of UCP1. UCP1 Cys253 in brown and beige adipose tissues undergoes sulfenylation during thermogenesis, and the heat generated by these adipocytes can fight obesity and diabetes.^1394^, ^1395^

In addition to the influence of ROS/RNS, antioxidant enzymes also play a role in metabolic diseases. Numerous studies have demonstrated that the activity of antioxidant enzymes can be used as potential biomarkers of metabolic diseases. For example, elevated levels of glutathione S‐transferase have been observed in the blood plasma of individuals with T2DM.^1396^ The activities of enzymes such as superoxide dismutases, catalases, and glutathione peroxidases in PBMCs of obese patients are found to be significantly lower.^1397^ In addition, the regulation of the Trx/trxR system has been identified as a potential target for treating metabolic syndrome, T2DM and hypertension.^14^, ^1398^, ^1399^

### 12.9 Redox modifications in the cardiovascular system

The heart is one of the organs most severely affected by SNO, which plays a key role in regulating redox homeostasis in the stressed heart.^1400^ In mouse cardiac proteins, a total of 1974 SNO sites from 761 proteins have been identified. In the cardiovascular system, NO signaling regulates vasodilation and myocardial contraction, and SNO of proteins may represent the third functional dimension of NO signaling in the cardiovascular system to ensure optimal cardiac function.^1401^ SNO at Cys589 of Hsp90 promotes cardiac hypertrophy.^1402^ Moreover, the SNO levels of MLP are significantly elevated in patients with hypertrophic myocardium and in spontaneously hypertensive rats and mice with transverse aortic constriction.^1403^ Cardiac fibrosis is an irreversible pathological process, and inhibition of SNO of Hsp90 can alleviate myocardial fibrosis through the TGFβ/SMAD3 signaling pathway (Figure 24).^1404^ SNO at C116/C163 of JNK accelerates cardiac fibrosis.^1405^ SNO of Hsp90 affects cardiac hypertrophy and myocardial fibrosis. A recent study found that SNO of C521 of Hsp90 can inhibit the interaction between Hsp90 and AHA1, promote the recruitment of CDC37, and aggravate atherosclerosis.^1406^ In addition, SNO mediates the coupling of GNAI2 to CXCR5, activating YAP‐dependent endothelial inflammation to drive diabetes‐accelerated atherosclerosis.^1407^ SNO is also involved in calcific aortic valve diseases. USP9X SNO is reduced in calcified human aortic valves. SNO of USP9X can stabilize MIB1, which activates the NOTCH1 signaling pathway in adjacent cells to prevent calcification.^1408^ In addition to SNO, protein S‐glutathionylation also plays a role in CVDs. During the development of calcific aortic valve stenosis, abnormal S‐glutathionylation promotes tissue phenotypic switching in the aortic valve, eventually leading to calcium deposition.^1409^

### 12.10 Redox modifications in neurological disorders

Redox homeostasis is linked to neurological disorders. ALS is a motor neuron disease. The G93A mutation in the antioxidant enzyme SOD1 is a gain‐of‐function mutation that causes SOD1 aggregation and motor neuron degeneration. Aggregation of SOD1 is associated with the oxidation of cysteine residues on SOD1 and increases when Cys6 and Cys111 are oxidized (Figure 24).^1410^, ^1411^ The pathogenesis of AD is related to Aβ, neuronal hyperexcitability, and aging‐related neuroinflammation. Excessive NO production leads to aberrant SNO of various proteins.^1412^ Moreover, noncanonical transnitrosylation can lead to synaptic loss, which may be one of the pathological causes of cognitive decline in AD patients.^1412^ SNO also affects the occurrence and progression of PD. HMGB1 is a DNA‐binding protein that regulates gene transcription and genome stability in the nucleus. SNO in C106 of HMGB1 can promote the secretion of HMGB1 and enhance its binding force with microglial Mac1, which is one of the key mechanisms for the development of PD.^1413^ In addition, SNO of foreign α‐synuclein also promotes Parkinopathy.^1414^

### 12.11 Redox modifications in cancers

ROS‐induced oxidative stress is a fundamental feature of cancer. Tumor cells produce more ROS than normal cells, resulting in abnormal redox homeostasis.^1415^ Oxidative stress can regulate intracellular signaling pathways and promote the growth of tumor cells, while excessive oxidative stress may lead to oxidative damage and even tumor cell death.^1416^ Tumor cells can rely on their own powerful antioxidant systems to withstand oxidative stress with high levels of ROS at different stages. For example, in the early stages of tumors, they can adapt to oxidative stress by activating antioxidant TFs or increasing NADPH through the PPP. During tumor growth and metastasis, tumor cells can activate AMPK, PPP, and reductive glutamine to increase NADPH, allowing cells to survive under conditions of high ROS.^1417^ During the whole process, the redox modifications of proteins in tumors play a crucial role (Figure 24). For example, SNO at C277 of VEGFD inhibits its expression and promotes the occurrence and development of LUAD.^1418^ In early CRC, PTPS is highly expressed and phosphorylated at Thr58 under hypoxic conditions, which promotes binding to LTBP1 and drives LTBP1 SNO, thereby maintaining tumor cell growth under hypoxic conditions.^1419^ Tumor cells can resist high levels of ROS by activating the activity of antioxidant TFs such as Nrf2. Nrf2 deletion affects the redox proteome of PC and NCSLC, which has impacts on mRNA translation machinery and the glycolytic pathway.^1420^, ^1421^ ROS can also affect tumor cells by regulating the functions of metabolic enzymes through redox modification. For example, TPβ is a redox‐sensitive protein and a key rate‐limiting enzyme in the FAO process. Under the stimulation of ROS, C458 of TPβ will be oxidized and inactivated. In oxidative stress caused by glucose starvation, Nur77 can enter the mitochondria and be oxidized by ROS, protecting C458 of TPβ from oxidation, resulting in the production of FAO‐mediated NADPH to relieve intracellular oxidative stress and promote melanoma cell survival and metastasis. This suggests that Nur77 may be a potential target for the treatment of melanoma.^1422^ In the glycolysis pathway, PFKM is one of the most important regulatory enzymes, and SNO at C351 of PFKM can stabilize the tetramer of PFKM, which contributes to the metabolic reprogramming of ovarian cancer cells.^1423^ Additionally, elevated expression of Nm23‐H1 is linked to a favorable prognosis in patients with breast cancer, and activation of Nm23‐H1 through redox regulation can inhibit breast cancer metastasis.^1424^ PTEN is a tumor suppressor. The oxidative modification of the cysteine of PTEN makes it inactive. The recovery of its activity mainly depends on the availability of Trx and Prx.^1425^ Methionine is an amino acid residue prone to redox modifications that are regulated by MSRA. MSRA deletion promotes the oxidation of methionine on PKM2, which in turn promotes mitochondrial respiration and cell metastasis.^1426^

## 13 OTHER MODIFICATIONS

### 13.1 ADP‐ribosylation

ADP‐ribosylation is a process in which ADP‐ribosyl transferases transfer ADP‐ribosyl to the target protein's ADP‐ribose binding domain using NAD^+^ as the substrate.^1427^ Currently, over 800 proteins have been found to contain the ADP‐ribose binding domain.^1428^ The structure and function of many proteins, including nuclear proteins topoisomerase I, DNA ligase II, endonuclease, histones H1, H2B and H4, DNA polymerases and cytoplasmic proteins adenyl cyclase and elongation factor eEF‐2, are regulated by ADP‐ribosylation.^1429^ ADP‐ribosylation is involved in numerous physiological and pathological processes, such as signal transduction, protein transport, transcription, DNA damage repair, cell cycle regulation, apoptosis, and necrosis.^1427^ Poly‐ADP‐ribosylation (PAR) of proteins may decease during aging because the activity of poly‐ADP‐ribose polymerase (PARP) in senescent human fibroblasts is reduced with donor age and continuous passage in vitro.^1429^ PAR modification plays an important role in DNA damage repair,^1430^ and many DNA damage repair factors can recognize PAR signals, leading to the rapid recruitment of these factors for efficient repair.^1431^ Loss of PAR modification inhibits SSB and DSB repair.^1432^ Therefore, PARP inhibitors (PARPi), such as olaparib, rucaparib, niraparib, and talazoparib, have been developed as a class of targeted drugs for cancer treatment.^1433^ These drugs compete with PARP1/2 for intracellular NAD^+^ and inhibit its catalytic activity to block DNA damage repair signals. Additionally, PARP1/2 can become trapped in damaged DNA, forming a PARP–DNA complex and blocking its release, causing replication fork stagnation and leading to cancer cell death.^1434^, ^1435^

### 13.2 Benzoylation

Lysine benzoylation (Kbz) was the first discovered aromatic fatty acid modification and primarily occurs in the N‐terminal tails of histones.^1436^ Sodium benzoate (SB) is a widely used food additive and a clinical treatment for hyperammonemia.^1437^ It can be converted into benzoyl‐CoA in mammalian cells and as a precursor of Kbz.^1438^ Moreover, benzoyl‐CoA is also a central intermediate for the degradation of numerous aromatic growth substrates in bacteria and gut microbes.^1439^ Previous studies have showed that HBO1 and the Spt‐Ada‐Gcn5 acetyltransferase complex are writers of histone Kbz.^1440^, ^1441^ In vivo studies have found that SIRT2, unlike other sirtuins or histone deacetylases (HDACs), acts as an eraser of histone Kbz.^1436^ NAD^+^‐dependent histone deacetylase Hst2 has debenzoylase activity in yeast.^1440^ Human DPF and YEATS but not BRD domains are the readers for histone benzoylation.^1442^

Histone Kbz is a mark enriched in gene promoters and is associated with gene expression.^1436^ Furthermore, it has a different physiological relevance than histone acetylation. Kbz primarily targets gene promoters and regulates glycerophospholipid metabolism, ovarian steroid synthesis, and hydrolytic phospholipase signaling pathways.^1438^ Excessive intake of SB increases Kbz levels, leading to motor coordination disorders and increased risk of diseases such as ADHD.^1443^

### 13.3 Neddylation

Neddylation is a PTM that can conjugate the Ub‐like protein NEDD8 to target proteins.^1444^ Neddylation has a broad range of functions and can alter various aspects of protein function, including protein conformation, stability, subcellular localization, affinity for DNA, and binding of protein substrates.^1445^, ^1446^ NEDD8 is a highly conserved protein composed of 81 amino acids. Of all the Ub‐like protein families, NEDD8 is the molecule with the highest sequence and structural similarity to Ub.^1444^ After passing through the E1–E2–E3 cascade, NEDD8 covalently binds to the substrate and undergoes Neddylation on the lysine of the substrate protein clock, thereby regulating the biological function of the substrate.^1444^

NEDD8 activating enzyme E1 is a heterodimer composed of APPBP1/UBA3.^1447^ Through its ATP‐dependent catalytic subunit, it catalyzes the formation of a high‐energy sulfolipid bond at the C‐terminal glycine of NEDD8 molecule and the cystine active site of UBA3 to activate the NEDD8 molecule.^1448^ The NEDD8‐loaded NEDD8‐activating enzyme (NAE) is transferred to E2‐conjugating enzymes UBC12/UBE2M or UBE2F via a trans‐thiolation reaction. Ultimately, substrate specific E3 ligases transfer NEDD8 from E2 to lysine residues in their target proteins by covalent attachment. NEDD8 ligase E3 can be divided into RING finger and HECT types.^1449^ Many neddylation E3s have been discovered, including RBX1/2, ROC1/2, SAG, c‐CBL, MDM2, FBXO11, c‐CBL, DCNL1–5, IAPs, RNF111, TFB3, and TRIM40.^1448^, ^1449^ Neddylation is a reversible process, and under the action of the de‐neddylation enzyme, NEDD8 can be dissociated from the substrate protein.^1450^

Similar to ubiquitination, the neddylation process also affects cell growth in multiple aspects such as proliferation, apoptosis, and migration in tumor cells. Many recent studies have shown that NEDD8 or enzymes related to the neddylation pathway are overexpressed in various cancers, including lung cancer,^1451^ liver cancer,^1452^ CRC,^1449^ and esophageal squamous cell carcinoma.^1453^ At present, the primary approach to studying the neddylation pathway involves interfering with the expression of particular genes through siRNA or inhibiting the activation enzyme NAE using small molecule inhibitors such as MLN4924 which deactivate the entire neddylation pathway.^1454^ The formation of a stable covalent bond with NEDD8 and competitive inhibition of NAE's activation of NEDD8 leads to inhibition of the neddylation pathway and partially blocks the ubiquitination pathway dependent on CRL. This inhibition results in the accumulation of substrates, triggering various cellular responses, such as cell cycle arrest, apoptosis, aging, and autophagy. By utilizing this mechanism, it is possible to achieve a therapeutic effect for treating tumors.^1455^ In addition, the dysregulation of neddylation is involved in the development of various diseases such as neurodegenerative diseases,^1456^ inflammation,^1457^ and CVDs,^1445^ suggesting that it can be a potential target for disease treatment. In fact, the NAE inhibitor MLN4924 not only has significant antitumor activity, but also has activities such as antiviral^1458^ and anti‐inflammatory effects.^1459^ Collectively, neddylation is a versatile PTM that can modulate a wide range of protein functions, with implications for many physiological and pathological processes. The neddylation pathway and its inhibitors hold significant potential for research into various disease treatments.

## 14 PERSPECTIVE

Protein modification signaling pathways play important roles in physiological and pathological processes.^10^ Compared with genomics, proteomics can instantly reflect the profiles of proteins and protein modifications of individuals in the disease state and normal state and has more prospects for diagnosis and treatment in the clinic.^1460^, ^1461^ Deciphering the complex biological functions of proteins requires a deep understanding of protein modification, referred to as the “PTM code.”^1462^, ^1463^, ^1464^ In recent years, the rapid development of MS‐based proteomics technology has greatly advanced the research progress of protein PTMs. Despite these advances, high‐throughput characterization of protein modifications remains a challenging task.^1465^, ^1466^, ^1467^ First, there is a lack of effective antibodies and reagents to enrich the modified peptides of some modification types, such as methylation.^1468^ Second, large‐scale PTMomics analysis of clinical samples can be difficult due to the limited availability of samples from clinical cohorts.^1469^ Third, the dynamic range of the human proteome exceeds several orders of magnitude, exacerbating the identification of low‐abundance modified proteins.^1470^ Fourth, complete structural characterization of proteins requires more substance and analysis time than “simple” identification based on some peptide fragments.^1470^ Finally, the modification groups on some proteins such as antibodies, often have poor chemical stability and low abundance, which also puts forward higher requirements for more sensitive MS detection.^1471^

When a protein PTM performs its function, it is often not the PTM at a single site that does it. In fact, crosstalk between protein modifications is common.^1472^, ^1473^, ^1474^ It includes not only the crosstalk of different modifications of the same protein at the same site but also the crosstalk of different modification sites of the same protein.^1475^, ^1476^ In addition, there is also extensive crosstalk between modifications on different proteins.^1477^ However, research on this PTM crosstalk is still in its infancy. The development of top‐down proteomics technology, high‐throughput PTM omics technology and novel PTM crosstalk analysis algorithm will provide the most powerful help to reveal the crosstalk of PTMs.^1478^, ^1479^, ^1480^

An increasing number of studies have demonstrated the close relationship between protein modifications and diseases.^1465^, ^1481^, ^1482^ Thanks to the rapid development of simple and efficient phosphorylation enrichment technology, the phosphoproteomes of some diseases, such as tumors and neurodegenerative diseases, have been revealed.^1483^, ^1484^, ^1485^ Some phosphosites are closely related to the occurrence and development of these diseases. In addition, preliminary progress has also been made in the acetylome, ubiquitinome and glycosylome in clinical samples.^140^, ^141^, ^148^, ^161^, ^1486^, ^1487^, ^1488^, ^1489^ However, studies of the relationship between many other types of protein modifications and diseases in large clinical cohorts remain lacking. The establishment of a multiomics molecular network based on the genome, proteome, PTMome, and metabolome will provide new breakthroughs for the discovery of new biomarkers and drug targets.^1490^, ^1491^ In addition, some irreversible protein modifications, such as citrullination and carbamylation, may generate neoantigens.^1492^, ^1493^, ^1494^ Uncovering the roles of these neoantigens in diseases will also be the focus of future research.

Although more than 650 types of protein modifications have been identified (http://www.uniprot.org/docs/ptmlist.txt), we believe that with the rapid development of new technologies and algorithms, the number of novel PTMs may still increase, which will help to elucidate the code of life.

## AUTHOR CONTRIBUTIONS

L.D. organized the team and revised the manuscript. Q.Z., X.X., Y.Q. Z.X., C.C., B.C. X.Z., S.H., S.L., and Z.A. drafted the manuscript and participated in the discussion. All authors have read and approved the final manuscript.

## CONFLICT OF INTEREST STATEMENT

The authors declare no competing interests.

## ETHICS STATEMENT

No ethical approval was needed.

## Data Availability

No additional data are included.

## References

[1] Millan‐Zambrano G , Burton A , Bannister AJ , Schneider R . Histone post‐translational modifications: cause and consequence of genome function. Nat Rev Genet. 2022;23(9):563‐580. 35338361 10.1038/s41576-022-00468-7

[2] Li W , Li F , Zhang X , Lin H‐K , Xu C . Insights into the post‐translational modification and its emerging role in shaping the tumor microenvironment. Signal Transduct Target Ther. 2021;6(1):422. 34924561 10.1038/s41392-021-00825-8 PMC8685280

[3] Müller MM . Post‐translational modifications of protein backbones: unique functions, mechanisms, and challenges. Biochemistry. 2018;57(2):177‐185. 29064683 10.1021/acs.biochem.7b00861 PMC5770884

[4] Keenan EK , Zachman DK , Hirschey MD . Discovering the landscape of protein modifications. Mol Cell. 2021;81(9):1868‐1878. 33798408 10.1016/j.molcel.2021.03.015 PMC8106652

[5] Prabakaran S , Lippens G , Steen H , Gunawardena J . Post‐translational modification: nature's escape from genetic imprisonment and the basis for dynamic information encoding. Wiley Interdiscip Rev Syst Biol Med. 2012;4(6):565‐583. 22899623 10.1002/wsbm.1185 PMC3473174

[6] López‐Maury L , Marguerat S , Bähler J . Tuning gene expression to changing environments: from rapid responses to evolutionary adaptation. Nat Rev Genet. 2008;9(8):583‐593. 18591982 10.1038/nrg2398

[7] Wang S , Osgood AO , Chatterjee A . Uncovering post‐translational modification‐associated protein‐protein interactions. Curr Opin Struct Biol. 2022;74:102352. 35334254 10.1016/j.sbi.2022.102352 PMC9464464

[8] Snider NT , Omary MB . Post‐translational modifications of intermediate filament proteins: mechanisms and functions. Nat Rev Mol Cell Biol. 2014;15(3):163‐177. 24556839 10.1038/nrm3753 PMC4079540

[9] Ramazi S , Zahiri J . Posttranslational modifications in proteins: resources, tools and prediction methods. Database (Oxford). 2021;2021:baab012. 33826699 10.1093/database/baab012 PMC8040245

[10] Wang H , Yang L , Liu M , Luo J . Protein post‐translational modifications in the regulation of cancer hallmarks. Cancer Gene Ther. 2022. 10.1038/s41417-022-00464-3 35393571

[11] Walsh CT , Garneau‐Tsodikova S , Gatto GJ, Jr. Protein posttranslational modifications: the chemistry of proteome diversifications. Angew Chem Int Ed Engl. 2005;44(45):7342‐7372. 16267872 10.1002/anie.200501023

[12] Xie H , Vucetic S , Iakoucheva LM , et al. Functional anthology of intrinsic disorder. 3. Ligands, post‐translational modifications, and diseases associated with intrinsically disordered proteins. J Proteome Res. 2007;6(5):1917‐1932. 17391016 10.1021/pr060394e PMC2588348

[13] Macek B , Forchhammer K , Hardouin J , Weber‐Ban E , Grangeasse C , Mijakovic I . Protein post‐translational modifications in bacteria. Nat Rev Microbiol. 2019;17(11):651‐664. 31485032 10.1038/s41579-019-0243-0

[14] Conibear AC . Deciphering protein post‐translational modifications using chemical biology tools. Nat Rev Chem. 2020;4(12):674‐695. 37127974 10.1038/s41570-020-00223-8

[15] Beltrao P , Albanese V , Kenner LR , et al. Systematic functional prioritization of protein posttranslational modifications. Cell. 2012;150(2):413‐425. 22817900 10.1016/j.cell.2012.05.036 PMC3404735

[16] Ahearn IM , Haigis K , Bar‐Sagi D , Philips MR . Regulating the regulator: post‐translational modification of RAS. Nat Rev Mol Cell Biol. 2011;13(1):39‐51. 22189424 10.1038/nrm3255 PMC3879958

[17] Humphrey SJ , James DE , Mann M . Protein phosphorylation: a major switch mechanism for metabolic regulation. Trends Endocrinol Metab. 2015;26(12):676‐687. 26498855 10.1016/j.tem.2015.09.013

[18] Liddy KA , White MY , Cordwell SJ . Functional decorations: post‐translational modifications and heart disease delineated by targeted proteomics. Genome Med. 2013;5(2):20. 23445784 10.1186/gm424 PMC3706772

[19] Chen L , Liu S , Tao Y . Regulating tumor suppressor genes: post‐translational modifications. Signal Transduct Target Ther. 2020;5(1):90. 32532965 10.1038/s41392-020-0196-9 PMC7293209

[20] Shvedunova M , Akhtar A . Modulation of cellular processes by histone and non‐histone protein acetylation. Nat Rev Mol Cell Biol. 2022;23(5):329‐349. 35042977 10.1038/s41580-021-00441-y

[21] Nozaki T , Kanai M . Chemical catalysis intervening to histone epigenetics. Acc Chem Res. 2021;54(9):2313‐2322. 33847478 10.1021/acs.accounts.1c00144

[22] Liu J , Qian C , Cao X . Post‐translational modification control of innate immunity. Immunity. 2016;45(1):15‐30. 27438764 10.1016/j.immuni.2016.06.020

[23] Guccione E , Richard S . The regulation, functions and clinical relevance of arginine methylation. Nat Rev Mol Cell Biol. 2019;20(10):642‐657. 31350521 10.1038/s41580-019-0155-x

[24] Polletta L , Vernucci E , Carnevale I , et al. SIRT5 regulation of ammonia‐induced autophagy and mitophagy. Autophagy. 2015;11(2):253‐270. 25700560 10.1080/15548627.2015.1009778 PMC4502726

[25] Ubersax JA , Ferrell JE, Jr. Mechanisms of specificity in protein phosphorylation. Nat Rev Mol Cell Biol. 2007;8(7):530‐541. 17585314 10.1038/nrm2203

[26] Choudhary C , Kumar C , Gnad F , et al. Lysine acetylation targets protein complexes and co‐regulates major cellular functions. Science. 2009;325(5942):834‐840. 19608861 10.1126/science.1175371

[27] Wu Q , Schapira M , Arrowsmith CH , Barsyte‐Lovejoy D . Protein arginine methylation: from enigmatic functions to therapeutic targeting. Nat Rev Drug Discov. 2021;20(7):509‐530. 33742187 10.1038/s41573-021-00159-8

[28] Ohtsubo K , Marth JD . Glycosylation in cellular mechanisms of health and disease. Cell. 2006;126(5):855‐867. 16959566 10.1016/j.cell.2006.08.019

[29] Meyer‐Schwesinger C . The ubiquitin–proteasome system in kidney physiology and disease. Nat Rev Nephrol. 2019;15(7):393‐411. 31036905 10.1038/s41581-019-0148-1

[30] Janssen SM , Lorincz MC . Interplay between chromatin marks in development and disease. Nat Rev Genet. 2022;23(3):137‐153. 34608297 10.1038/s41576-021-00416-x

[31] Li C , Gotz J . Tau‐based therapies in neurodegeneration: opportunities and challenges. Nat Rev Drug Discov. 2017;16(12):863‐883. 28983098 10.1038/nrd.2017.155

[32] Lemarie FL , Caron NS , Sanders SS , et al. Rescue of aberrant huntingtin palmitoylation ameliorates mutant huntingtin‐induced toxicity. Neurobiol Dis. 2021;158:105479. 34390831 10.1016/j.nbd.2021.105479

[33] Yang Q , Vijayakumar A , Kahn BB . Metabolites as regulators of insulin sensitivity and metabolism. Nat Rev Mol Cell Biol. 2018;19(10):654‐672. 30104701 10.1038/s41580-018-0044-8 PMC6380503

[34] Du Y , Cai T , Li T , et al. Lysine malonylation is elevated in type 2 diabetic mouse models and enriched in metabolic associated proteins. Mol Cell Proteomics. 2015;14(1):227‐236. 25418362 10.1074/mcp.M114.041947 PMC4288257

[35] Ma W , Liu Y , Lei P , Zhu M , Pan X . Novel compound, ND‐17, regulates the JAK/STAT, PI3K/AKT, and MAPK pathways and restrains human T‐lymphoid leukemia Development. Curr Cancer Drug Targets. 2022;22(5):404‐413. 35249489 10.2174/1568009622666220304202116

[36] Vucic D , Dixit VM , Wertz IE . Ubiquitylation in apoptosis: a post‐translational modification at the edge of life and death. Nat Rev Mol Cell Biol. 2011;12(7):439‐452. 21697901 10.1038/nrm3143

[37] Wang ZJ , Hanet A , Weishaupl D , et al. Divalproex sodium modulates nuclear localization of ataxin‐3 and prevents cellular toxicity caused by expanded ataxin‐3. CNS Neurosci Ther. 2018;24(5):404‐411. 29318784 10.1111/cns.12795 PMC6489778

[38] Fraga MF , Ballestar E , Villar‐Garea A , et al. Loss of acetylation at Lys16 and trimethylation at Lys20 of histone H4 is a common hallmark of human cancer. Nat Genet. 2005;37(4):391‐400. 15765097 10.1038/ng1531

[39] Krivtsov AV , Feng Z , Lemieux ME , et al. H3K79 methylation profiles define murine and human MLL‐AF4 leukemias. Cancer Cell. 2008;14(5):355‐368. 18977325 10.1016/j.ccr.2008.10.001 PMC2591932

[40] Wang P , Lin C , Smith ER , et al. Global analysis of H3K4 methylation defines MLL family member targets and points to a role for MLL1‐mediated H3K4 methylation in the regulation of transcriptional initiation by RNA polymerase II. Mol Cell Biol. 2009;29(22):6074‐6085. 19703992 10.1128/MCB.00924-09 PMC2772563

[41] Shi Y . Serine/threonine phosphatases: mechanism through structure. Cell. 2009;139(3):468‐484. 19879837 10.1016/j.cell.2009.10.006

[42] Ardito F , Giuliani M , Perrone D , Troiano G , Lo Muzio L . The crucial role of protein phosphorylation in cell signaling and its use as targeted therapy (Review). Int J Mol Med. 2017;40(2):271‐280. 28656226 10.3892/ijmm.2017.3036 PMC5500920

[43] Bilbrough T , Piemontese E , Seitz O . Dissecting the role of protein phosphorylation: a chemical biology toolbox. Chem Soc Rev. 2022;51(13):5691‐5730. 35726784 10.1039/d1cs00991e

[44] Chevalier D , Morris ER , Walker JC . 14‐3‐3 and FHA domains mediate phosphoprotein interactions. Annu Rev Plant Biol. 2009;60:67‐91. 19575580 10.1146/annurev.arplant.59.032607.092844

[45] Gong W , Zhou D , Ren Y , et al. PepCyber:P∼PEP: a database of human protein protein interactions mediated by phosphoprotein‐binding domains. Nucleic Acids Res. 2008;36(Database issue):D679‐D683. 18160410 10.1093/nar/gkm854 PMC2238930

[46] Zhao L , Yuan X , Wang J , et al. A review on flavones targeting serine/threonine protein kinases for potential anticancer drugs. Bioorg Med Chem. 2019;27(5):677‐685. 30733087 10.1016/j.bmc.2019.01.027

[47] Qu N , Li F , Shao B , et al. The unexpected and exceptionally facile chemical modification of the phenolic hydroxyl group of tyrosine by polyhalogenated quinones under physiological conditions. Chem Res Toxicol. 2016;29(10):1699‐1705. 27611113 10.1021/acs.chemrestox.6b00217

[48] Wolanin PM , Thomason PA , Stock JB . Histidine protein kinases: key signal transducers outside the animal kingdom. Genome Biol. 2002;3(10):REVIEWS3013. 12372152 10.1186/gb-2002-3-10-reviews3013 PMC244915

[49] Gotink KJ , Verheul HM . Anti‐angiogenic tyrosine kinase inhibitors: what is their mechanism of action? Angiogenesis. 2010;13(1):1‐14. 20012482 10.1007/s10456-009-9160-6 PMC2845892

[50] Yin Z , Zou Y , Wang D , et al. Regulation of the Tec family of non‐receptor tyrosine kinases in cardiovascular disease. Cell Death Dis. 2022;8(1):119. 10.1038/s41420-022-00927-4 PMC8927484 35296647

[51] Goel HL , Mercurio AM . VEGF targets the tumour cell. Nat Rev Cancer. 2013;13(12):871‐882. 24263190 10.1038/nrc3627 PMC4011842

[52] Turner N , Grose R . Fibroblast growth factor signalling: from development to cancer. Nat Rev Cancer. 2010;10(2):116‐129. 20094046 10.1038/nrc2780

[53] Siveen KS , Prabhu KS , Achkar IW , et al. Role of non receptor tyrosine kinases in hematological malignances and its targeting by natural products. Mol Cancer. 2018;17(1):31. 29455667 10.1186/s12943-018-0788-y PMC5817858

[54] Chartier M , Chenard T , Barker J , Najmanovich R . Kinome Render: a stand‐alone and web‐accessible tool to annotate the human protein kinome tree. PeerJ. 2013;1:e126. 23940838 10.7717/peerj.126 PMC3740139

[55] Perrotti D , Neviani P . Protein phosphatase 2A: a target for anticancer therapy. Lancet Oncol. 2013;14(6):e229‐e238. 23639323 10.1016/S1470-2045(12)70558-2 PMC3913484

[56] Amoozadeh M , Behbahani M , Mohabatkar H , Keyhanfar M . Analysis and comparison of alkaline and acid phosphatases of Gram‐negative bacteria by bioinformatic and colorimetric methods. J Biotechnol. 2020;308:56‐62. 31705933 10.1016/j.jbiotec.2019.11.002

[57] Jin J , Pawson T . Modular evolution of phosphorylation‐based signalling systems. Philos Trans R Soc Lond B Biol Sci. 2012;367(1602):2540‐2555. 22889906 10.1098/rstb.2012.0106 PMC3415845

[58] Sacco F , Perfetto L , Castagnoli L , Cesareni G . The human phosphatase interactome: an intricate family portrait. FEBS Lett. 2012;586(17):2732‐2739. 22626554 10.1016/j.febslet.2012.05.008 PMC3437441

[59] Li X , Wilmanns M , Thornton J , Köhn M . Elucidating human phosphatase‐substrate networks. Sci Signal. 2013;6(275):rs10. 23674824 10.1126/scisignal.2003203

[60] Das AK , Helps NR , Cohen PT , Barford D . Crystal structure of the protein serine/threonine phosphatase 2C at 2.0 A resolution. EMBO J. 1996;15(24):6798‐6809. 9003755 PMC452505

[61] Alonso A , Sasin J , Bottini N , et al. Protein tyrosine phosphatases in the human genome. Cell. 2004;117(6):699‐711. 15186772 10.1016/j.cell.2004.05.018

[62] Pils B , Schultz J . Evolution of the multifunctional protein tyrosine phosphatase family. Mol Biol Evol. 2004;21(4):625‐631. 14739250 10.1093/molbev/msh055

[63] Chen MJ , Dixon JE , Manning G . Genomics and evolution of protein phosphatases. Sci Signal. 2017;10(474):eaag1796. 28400531 10.1126/scisignal.aag1796

[64] Tarrant MK , Cole PA . The chemical biology of protein phosphorylation. Annu Rev Biochem. 2009;78:797‐825. 19489734 10.1146/annurev.biochem.78.070907.103047 PMC3074175

[65] Lu KP , Zhou XZ . The prolyl isomerase PIN1: a pivotal new twist in phosphorylation signalling and disease. Nat Rev Mol Cell Biol. 2007;8(11):904‐916. 17878917 10.1038/nrm2261

[66] Guo X , Wang X , Wang Z , et al. Site‐specific proteasome phosphorylation controls cell proliferation and tumorigenesis. Nat Cell Biol. 2016;18(2):202‐212. 26655835 10.1038/ncb3289 PMC4844191

[67] Iyer SC , Casas‐Pastor D , Kraus D , et al. Transcriptional regulation by σ factor phosphorylation in bacteria. Nat Microbiol. 2020;5(3):395‐406. 31988380 10.1038/s41564-019-0648-6

[68] Hossain MB , Shifat R , Johnson DG , et al. TIE2‐mediated tyrosine phosphorylation of H4 regulates DNA damage response by recruiting ABL1. Sci Adv. 2016;2(4):e1501290. 27757426 10.1126/sciadv.1501290 PMC5065225

[69] Yogosawa S , Yoshida K . Tumor suppressive role for kinases phosphorylating p53 in DNA damage‐induced apoptosis. Cancer Sci. 2018;109(11):3376‐3382. 30191640 10.1111/cas.13792 PMC6215896

[70] Acconcia F , Barnes CJ , Singh RR , Talukder AH , Kumar R . Phosphorylation‐dependent regulation of nuclear localization and functions of integrin‐linked kinase. Proc Natl Acad Sci U S A. 2007;104(16):6782‐6787. 17420447 10.1073/pnas.0701999104 PMC1871862

[71] Zhang J , Wang S , Jiang B , et al. c‐Src phosphorylation and activation of hexokinase promotes tumorigenesis and metastasis. Nat Commun. 2017;8:13732. 28054552 10.1038/ncomms13732 PMC5227066

[72] Harsha HC , Pandey A . Phosphoproteomics in cancer. Mol Oncol. 2010;4(6):482‐495. 20937571 10.1016/j.molonc.2010.09.004 PMC3030978

[73] Lahiry P , Torkamani A , Schork NJ , Hegele RA . Kinase mutations in human disease: interpreting genotype‐phenotype relationships. Nat Rev Genet. 2010;11(1):60‐74. 20019687 10.1038/nrg2707

[74] Pike KA , Tremblay ML . Protein tyrosine phosphatases: regulators of CD4 T cells in inflammatory bowel disease. Front Immunol. 2018;9:2504. 30429852 10.3389/fimmu.2018.02504 PMC6220082

[75] Xu K , Zhao X , Fu X , et al. Gender effect of hyperuricemia on the development of nonalcoholic fatty liver disease (NAFLD): a clinical analysis and mechanistic study. Biomed Pharmacother. 2019;117:109158. 31252266 10.1016/j.biopha.2019.109158

[76] Lassen PS , Thygesen C , Larsen MR , Kempf SJ . Understanding Alzheimer's disease by global quantification of protein phosphorylation and sialylated N‐linked glycosylation profiles: a chance for new biomarkers in neuroproteomics? J Proteomics. 2017;161:11‐25. 28396268 10.1016/j.jprot.2017.04.003

[77] Chou J , Quigley DA , Robinson TM , Feng FY , Ashworth A . Transcription‐associated Cyclin‐dependent kinases as targets and biomarkers for cancer therapy. Cancer Discov. 2020;10(3):351‐370. 32071145 10.1158/2159-8290.CD-19-0528

[78] Hanahan D , Weinberg RA . Hallmarks of cancer: the next generation. Cell. 2011;144(5):646‐674. 21376230 10.1016/j.cell.2011.02.013

[79] Su K‐H , Cao J , Tang Z , et al. HSF1 critically attunes proteotoxic stress sensing by mTORC1 to combat stress and promote growth. Nat Cell Biol. 2016;18(5):527‐539. 27043084 10.1038/ncb3335 PMC5341796

[80] Krentz NAJ , van Hoof D , Li Z , et al. Phosphorylation of NEUROG3 links endocrine differentiation to the cell cycle in pancreatic progenitors. Dev Cell. 2017;41(2):129‐142. e6. 28441528 10.1016/j.devcel.2017.02.006 PMC5517315

[81] Shyh‐Chang N , Daley GQ , Cantley LC . Stem cell metabolism in tissue development and aging. Development. 2013;140(12):2535‐2547. 23715547 10.1242/dev.091777 PMC3666381

[82] Hassan AM , Mancano G , Kashofer K , et al. High‐fat diet induces depression‐like behaviour in mice associated with changes in microbiome, neuropeptide Y, and brain metabolome. Nutr Neurosci. 2019;22(12):877‐893. 29697017 10.1080/1028415X.2018.1465713

[83] Svoboda P , Franke V , Schultz RM . Chapter Nine ‐ Sculpting the transcriptome during the oocyte‐to‐embryo transition in mouse. In: Lipshitz HD , ed. Curr Top Dev Biol. Academic Press; 2015:305‐349. 10.1016/bs.ctdb.2015.06.004 26358877

[84] Zhang Y , Wang HH , Wan X , Xu Y , Pan MH , Sun SC . Inhibition of protein kinase D disrupts spindle formation and actin assembly during porcine oocyte maturation. Aging. 2018;10(1945‐4589 (Electronic)):3736‐3744. 30555056 10.18632/aging.101667 PMC6326681

[85] Yaeger R , Corcoran RB . Targeting alterations in the RAF‐MEK pathway. Cancer Discov. 2019;9(2159‐8290 (Electronic)):329‐341. 30770389 10.1158/2159-8290.CD-18-1321 PMC6397699

[86] Voisin L , Julien C , Duhamel S , et al. Activation of MEK1 or MEK2 isoform is sufficient to fully transform intestinal epithelial cells and induce the formation of metastatic tumors. BMC Cancer. 2008;8(1471‐2407 (Electronic)):337. 19014680 10.1186/1471-2407-8-337 PMC2596176

[87] Battaglioni S , Benjamin D , Wälchli M , Maier T , Hall MN . mTOR substrate phosphorylation in growth control. Cell. 2022;185(11):1814‐1836. 35580586 10.1016/j.cell.2022.04.013

[88] Chu C , Geng Y , Zhou Y , Sicinski P . Cyclin E in normal physiology and disease states. Trends Cell Biol. 2021;31(1879‐3088 (Electronic)):732‐746. 34052101 10.1016/j.tcb.2021.05.001 PMC8364501

[89] Parisi T , Beck Ar Fau ‐ Rougier N , Rougier N Fau ‐ McNeil T , et al. Cyclins E1 and E2 are required for endoreplication in placental trophoblast giant cells. EMBO J. 2003;22(0261‐4189 (Print)):4794‐803. 12970191 10.1093/emboj/cdg482 PMC212738

[90] Hernandez‐Segura A , Nehme J , Demaria M . Hallmarks of cellular senescence. Trends in Cell Biol. 2018;28(6):436‐453. 29477613 10.1016/j.tcb.2018.02.001

[91] Muñoz‐Espín D , Serrano M . Cellular senescence: from physiology to pathology. Nat Rev Mol Cell Biol. 2014;15(7):482‐496. 24954210 10.1038/nrm3823

[92] López‐Otín C , Blasco MA , Partridge L , Serrano M , Kroemer G . The hallmarks of aging. Cell. 2013;153(6):1194‐1217. 23746838 10.1016/j.cell.2013.05.039 PMC3836174

[93] Harada H , Farhani N , Wang X‐F , et al. Extracellular phosphorylation drives the formation of neuronal circuitry. Nat Chem Biol. 2019;15(11):1035‐1042. 31451763 10.1038/s41589-019-0345-z

[94] Heydari AR , Butler JA , Waggoner SM , Richardson A . Age‐related changes in protein phosphorylation by rat hepatocytes. Mech Ageing Dev. 1989;50(3):227‐248. 2561003 10.1016/0047-6374(89)90102-4

[95] Bakthisaran R , Akula KK , Tangirala R , Rao CM . Phosphorylation of αB‐crystallin: role in stress, aging and patho‐physiological conditions. BBA. 2016;1860(1, Part B):167‐182. 26415747 10.1016/j.bbagen.2015.09.017

[96] Doran P , Gannon J , O'Connell K , Ohlendieck K . Aging skeletal muscle shows a drastic increase in the small heat shock proteins αB‐crystallin/HspB5 and cvHsp/HspB7. Eur J Cell Biol. 2007;86(10):629‐640. 17761354 10.1016/j.ejcb.2007.07.003

[97] Ueda Y , Duncan MK , Fau‐David LL , David LL . Lens proteomics: the accumulation of crystallin modifications in the mouse lens with age. Invest Ophthalmol Vis Sci. 2002;43(0146‐0404 (Print)):205‐215. 11773033

[98] Timofeev O , Koch L , Niederau C , et al. Phosphorylation control of p53 DNA‐Binding cooperativity balances tumorigenesis and aging. Cancer Res. 2020;80(23):5231‐5244. 32873634 10.1158/0008-5472.CAN-20-2002

[99] Liu Y , Tavana O , Gu W . p53 modifications: exquisite decorations of the powerful guardian. J Mol Cell Biol. 2019;11(7):564‐577. 31282934 10.1093/jmcb/mjz060 PMC6736412

[100] Chibaya L , Karim B , Zhang H , Jones SN . Mdm2 phosphorylation by Akt regulates the p53 response to oxidative stress to promote cell proliferation and tumorigenesis. Proc Natl Acad Sci U S A. 2021;118(4):e2003193118. 33468664 10.1073/pnas.2003193118 PMC7848548

[101] Ferrer I , Andres‐Benito P , Ausin K , et al. Dysregulated protein phosphorylation: a determining condition in the continuum of brain aging and Alzheimer's disease. Brain Pathol. 2021;31(6):e12996. 34218486 10.1111/bpa.12996 PMC8549032

[102] van der Zee EA . Synapses, spines and kinases in mammalian learning and memory, and the impact of aging. Neurosci Biobehav Rev. 2015;50:77‐85. 24998408 10.1016/j.neubiorev.2014.06.012

[103] Tagawa K , Homma H , Saito A , et al. Comprehensive phosphoproteome analysis unravels the core signaling network that initiates the earliest synapse pathology in preclinical Alzheimer's disease brain. Hum Mol Genet. 2015;24(2):540‐558. 25231903 10.1093/hmg/ddu475

[104] Torres AK , Rivera BI , Polanco CM , Jara C , Tapia‐Rojas C . Phosphorylated tau as a toxic agent in synaptic mitochondria: implications in aging and Alzheimer's disease. Neural Regen Res. 2022;17(8):1645‐1651. 35017410 10.4103/1673-5374.332125 PMC8820692

[105] Ryall JG , Schertzer JD , Lynch GS . Cellular and molecular mechanisms underlying age‐related skeletal muscle wasting and weakness. Biogerontology. 2008;9(4):213‐228. 18299960 10.1007/s10522-008-9131-0

[106] Cruz‐Jentoft AJ , Bahat G , Bauer J , et al. Sarcopenia: revised European consensus on definition and diagnosis. Age Ageing. 2019;48(1):16‐31. 30312372 10.1093/ageing/afy169 PMC6322506

[107] Markofski MM , Dickinson JM , Drummond MJ , et al. Effect of age on basal muscle protein synthesis and mTORC1 signaling in a large cohort of young and older men and women. Exp Gerontol. 2015;65(1873‐6815 (Electronic)):1‐7. 25735236 10.1016/j.exger.2015.02.015 PMC4397165

[108] Gregorich ZR , Peng Y , Cai WX , et al. Top‐down targeted proteomics reveals decrease in myosin regulatory light‐chain phosphorylation that contributes to sarcopenic muscle dysfunction. J Proteome Res. 2016;15(8):2706‐2716. 27362462 10.1021/acs.jproteome.6b00244 PMC4975644

[109] Pao LI , Badour K , Siminovitch KA , Neel BG . Nonreceptor protein‐tyrosine phosphatases in immune cell signaling. Annu Rev Immunol. 2007;25:473‐523. 17291189 10.1146/annurev.immunol.23.021704.115647

[110] Mustelin T , Vang T , Bottini N . Protein tyrosine phosphatases and the immune response. Nat Rev Immunol. 2005;5(1):43‐57. 15630428 10.1038/nri1530

[111] Chuang HC , Wang X , Tan TH . MAP4K family kinases in immunity and inflammation. Adv Immunol. 2016;129:277‐314. 26791862 10.1016/bs.ai.2015.09.006

[112] Lim PS , Sutton CR , Rao S . Protein kinase C in the immune system: from signalling to chromatin regulation. Immunology. 2015;146(4):508‐522. 26194700 10.1111/imm.12510 PMC4693901

[113] Torgersen KM , Vang T , Abrahamsen H , Yaqub S , Taskén K . Molecular mechanisms for protein kinase A‐mediated modulation of immune function. Cell Signal. 2002;14(1):1‐9. 11747983 10.1016/s0898-6568(01)00214-5

[114] Veillette A , Latour S , Davidson D . Negative regulation of immunoreceptor signaling. Annu Rev Immunol. 2002;20:669‐707. 11861615 10.1146/annurev.immunol.20.081501.130710

[115] Shuai K . Serine phosphorylation: arming Stat1 against infection. Immunity. 2003;19(6):771‐772. 14670293 10.1016/s1074-7613(03)00329-7

[116] Panne D , McWhirter SM , Maniatis T , Harrison SC . Interferon regulatory factor 3 is regulated by a dual phosphorylation‐dependent switch. J Biol Chem. 2007;282(31):22816‐22822. 17526488 10.1074/jbc.M703019200

[117] Liu S , Cai X , Wu J , et al. Phosphorylation of innate immune adaptor proteins MAVS, STING, and TRIF induces IRF3 activation. Science. 2015;347(6227):aaa2630. 25636800 10.1126/science.aaa2630

[118] Liu R , Li J , Shao J , et al. Innate immune response orchestrates phosphoribosyl pyrophosphate synthetases to support DNA repair. Cell Metab. 2021;33(10):2076‐2089. e9. 34343500 10.1016/j.cmet.2021.07.009

[119] Chen H , Sun H , You F , et al. Activation of STAT6 by STING is critical for antiviral innate immunity. Cell. 2011;147(2):436‐446. 22000020 10.1016/j.cell.2011.09.022

[120] Steen HC , Kotredes KP , Nogusa S , Harris MY , Balachandran S , Gamero AM . Phosphorylation of STAT2 on serine‐734 negatively regulates the IFN‐α‐induced antiviral response. J Cell Sci. 2016;129(22):4190‐4199. 27802159 10.1242/jcs.185421 PMC6518330

[121] Wang S , Xie F , Chu F , et al. YAP antagonizes innate antiviral immunity and is targeted for lysosomal degradation through IKKɛ‐mediated phosphorylation. Nat Immunol. 2017;18(7):733‐743. 28481329 10.1038/ni.3744

[122] Wang Y , Tian Q , Hao Y , et al. The kinase complex mTORC2 promotes the longevity of virus‐specific memory CD4(+) T cells by preventing ferroptosis. Nat Immunol. 2022;23(2):303‐317. 34949833 10.1038/s41590-021-01090-1

[123] Yokosuka T , Takamatsu M , Kobayashi‐Imanishi W , Hashimoto‐Tane A , Azuma M , Saito T . Programmed cell death 1 forms negative costimulatory microclusters that directly inhibit T cell receptor signaling by recruiting phosphatase SHP2. J Exp Med. 2012;209(6):1201‐1217. 22641383 10.1084/jem.20112741 PMC3371732

[124] Zhang J , Bu X , Wang H , et al. Cyclin D‐CDK4 kinase destabilizes PD‐L1 via cullin 3‐SPOP to control cancer immune surveillance. Nature. 2018;553(7686):91‐95. 29160310 10.1038/nature25015 PMC5754234

[125] Zhang Y , Zhao M , Gao H , et al. MAPK signalling‐induced phosphorylation and subcellular translocation of PDHE1α promotes tumour immune evasion. Nat Metab. 2022;4(3):374‐388. 35315437 10.1038/s42255-022-00543-7

[126] Guo D , Tong Y , Jiang X , et al. Aerobic glycolysis promotes tumor immune evasion by hexokinase2‐mediated phosphorylation of IκBα. Cell Metab. 2022;34(9):1312‐1324. e6. 36007522 10.1016/j.cmet.2022.08.002

[127] American Diabetes A. 2. Classification and diagnosis of diabetes: standards of medical care in diabetes‐2019. Diabetes Care. 2019;42(Suppl 1):S13‐S28. 30559228 10.2337/dc19-S002

[128] Sacco F , Seelig A , Humphrey SJ , et al. Phosphoproteomics reveals the GSK3‐PDX1 axis as a key pathogenic signaling node in diabetic islets. Cell Metab. 2019;29(6):1422‐1432. e3. 30879985 10.1016/j.cmet.2019.02.012

[129] Yadav Y , Dey CS . Ser/Thr phosphatases: one of the key regulators of insulin signaling. Rev Endocr Metab Disord. 2022;23(5):905‐917. 35697962 10.1007/s11154-022-09727-8

[130] Batista TM , Jayavelu AK , Wewer Albrechtsen NJ , et al. A cell‐autonomous signature of dysregulated protein phosphorylation underlies muscle insulin resistance in type 2 diabetes. Cell Metab. 2020;32(5):844‐859. e5. 32888406 10.1016/j.cmet.2020.08.007 PMC7875546

[131] Zhang Y , Zhang Y , Yu Y . Global phosphoproteomic analysis of insulin/Akt/mTORC1/S6K signaling in rat hepatocytes. J Proteome Res. 2017;16(8):2825‐2835. 28689409 10.1021/acs.jproteome.7b00140 PMC5557384

[132] Huang B , Zhao Z , Zhao Y , Huang S . Protein arginine phosphorylation in organisms. Int J Biol Macromol. 2021;171:414‐422. 33428953 10.1016/j.ijbiomac.2021.01.015

[133] Hall JA , Ramachandran D , Roh HC , et al. Obesity‐linked PPARgamma S273 phosphorylation promotes insulin resistance through growth differentiation factor 3. Cell Metab. 2020;32(4):665‐675. e6. 32941798 10.1016/j.cmet.2020.08.016 PMC7543662

[134] Tozzi M , Brown EL , Petersen PSS , et al. Dynamic interplay between Afadin(S1795) phosphorylation and diet regulates glucose homeostasis in obese mice. J Physiol. 2022;600(4):885‐902. 34387373 10.1113/JP281657

[135] Yao J , Wu D , Zhang C , et al. Macrophage IRX3 promotes diet‐induced obesity and metabolic inflammation. Nat Immunol. 2021;22(10):1268‐1279. 34556885 10.1038/s41590-021-01023-y

[136] Yi SA , Um SH , Lee J , et al. S6K1 phosphorylation of H2B mediates EZH2 trimethylation of H3: a determinant of early adipogenesis. Mol Cell. 2016;62(3):443‐452. 27151441 10.1016/j.molcel.2016.03.011 PMC5325705

[137] Su H , Meng C , Xu J , Su Z , Xiao C , Yang D . Histone methyltransferase Smyd2 drives adipogenesis via regulating STAT3 phosphorylation. Cell Death Dis. 2022;13(10):890. 36270984 10.1038/s41419-022-05321-7 PMC9586978

[138] Yang N , Wang Y , Tian Q , et al. Blockage of PPARgamma T166 phosphorylation enhances the inducibility of beige adipocytes and improves metabolic dysfunctions. Cell Death Differ. 2022. 10.1038/s41418-022-01077-x PMC9984430 36329235

[139] Wu C‐J , Cai T , Rikova K , Merberg D , Kasif S , Steffen M . A predictive phosphorylation signature of lung cancer. PloS One. 2009;4(11):e7994. 19946374 10.1371/journal.pone.0007994 PMC2777383

[140] Gillette MA , Satpathy S , Cao S , et al. Proteogenomic characterization reveals therapeutic vulnerabilities in lung adenocarcinoma. Cell. 2020;182(1):200‐225. e35. 32649874 10.1016/j.cell.2020.06.013 PMC7373300

[141] Satpathy S , Krug K , Jean Beltran PM , et al. A proteogenomic portrait of lung squamous cell carcinoma. Cell. 2021;184(16):4348‐4371. e40. 34358469 10.1016/j.cell.2021.07.016 PMC8475722

[142] Xu J‐Y , Zhang C , Wang X , et al. Integrative proteomic characterization of human lung adenocarcinoma. Cell. 2020;182(1):245‐261. e17. 32649877 10.1016/j.cell.2020.05.043

[143] Li C , Sun Y‐D , Yu G‐Y , et al. Integrated omics of metastatic colorectal cancer. Cancer Cell. 2020;38(5):734‐747. e9. 32888432 10.1016/j.ccell.2020.08.002

[144] Vasaikar S , Huang C , Wang X , et al. Proteogenomic analysis of human colon cancer reveals new therapeutic opportunities. Cell. 2019;177(4):1035‐1049. e19. 31031003 10.1016/j.cell.2019.03.030 PMC6768830

[145] Gao L , Lu Y , Chen H‐N , et al. Deciphering the clinical significance and kinase functions of GSK3α in colon cancer by proteomics and phosphoproteomics. Mol Cell Proteom. 2023; 100545. 10.1016/j.mcpro.2023.100545 PMC10196724 37031867

[146] Zhang R , Hu M , Chen H‐N , et al. Phenotypic heterogeneity analysis of APC‐mutant colon cancer by proteomics and phosphoproteomics identifies RAI14 as a key prognostic determinant in East Asians and Westerners. Mol Cell Proteom. 2023; 100532. 10.1016/j.mcpro.2023.100532 PMC10148045 36934880

[147] Gao Q , Zhu H , Dong L , et al. Integrated proteogenomic characterization of HBV‐related hepatocellular carcinoma. Cell. 2019;179(5):1240. 31730861 10.1016/j.cell.2019.10.038

[148] Krug K , Jaehnig EJ , Satpathy S , et al. Proteogenomic landscape of breast cancer tumorigenesis and targeted therapy. Cell. 2020;183(5):1436‐1456. e31. 33212010 10.1016/j.cell.2020.10.036 PMC8077737

[149] Wong MT , Ong DEH , Lim FSH , et al. A high‐dimensional atlas of human T cell diversity reveals tissue‐specific trafficking and cytokine signatures. Immunity. 2016;45(2):442‐456. 27521270 10.1016/j.immuni.2016.07.007

[150] Mun D‐G , Bhin J , Kim S , et al. Proteogenomic characterization of human early‐onset gastric cancer. Cancer Cell. 2019;35(1):111‐124. e10. 30645970 10.1016/j.ccell.2018.12.003

[151] Li Y , Xu C , Wang B , et al. Proteomic characterization of gastric cancer response to chemotherapy and targeted therapy reveals new therapeutic strategies. Nat Commun. 2022;13(1):5723. 36175412 10.1038/s41467-022-33282-0 PMC9522856

[152] Huang C , Chen L , Savage SR , et al. Proteogenomic insights into the biology and treatment of HPV‐negative head and neck squamous cell carcinoma. Cancer Cell. 2021;39(3):361‐379. e16. 33417831 10.1016/j.ccell.2020.12.007 PMC7946781

[153] Liu W , Xie L , He YH , et al. Large‐scale and high‐resolution mass spectrometry‐based proteomics profiling defines molecular subtypes of esophageal cancer for therapeutic targeting. Nat Commun. 2021;12(1):4961. 34400640 10.1038/s41467-021-25202-5 PMC8368010

[154] Chen PY , Muzumdar MD , Dorans KJ , et al. Adaptive and reversible resistance to kras inhibition in pancreatic cancer cells. Cancer Res. 2018;78(4):985‐1002. 29279356 10.1158/0008-5472.CAN-17-2129 PMC5837062

[155] Cao L , Huang C , Cui Zhou D , et al. Proteogenomic characterization of pancreatic ductal adenocarcinoma. Cell. 2021;184(19):5031‐5052. e26. 34534465 10.1016/j.cell.2021.08.023 PMC8654574

[156] Clark DJ , Dhanasekaran SM , Petralia F , et al. Integrated proteogenomic characterization of clear cell renal cell carcinoma. Cell. 2019;179(4):964‐983. e31. 31675502 10.1016/j.cell.2019.10.007 PMC7331093

[157] Qu Y , Feng J , Wu X , et al. A proteogenomic analysis of clear cell renal cell carcinoma in a Chinese population. Nat Commun. 2022;13(1):2052. 35440542 10.1038/s41467-022-29577-x PMC9019091

[158] Basken J , Stuart SA , Kavran AJ , et al. Specificity of phosphorylation responses to mitogen activated protein (MAP) kinase pathway inhibitors in melanoma cells. Mol Cell Proteomics. 2018;17(4):550‐564. 29255136 10.1074/mcp.RA117.000335 PMC5880111

[159] Pastushenko I , Mauri F , Song Y , et al. Fat1 deletion promotes hybrid EMT state, tumour stemness and metastasis. Nature. 2021;589(7842):448‐455. 33328637 10.1038/s41586-020-03046-1 PMC7612440

[160] Kramer MH , Zhang Q , Sprung R , et al. Proteomic and phosphoproteomic landscapes of acute myeloid leukemia. Blood. 2022;140(13):1533‐1548. 35895896 10.1182/blood.2022016033 PMC9523374

[161] Tong Y , Sun M , Chen L , et al. Proteogenomic insights into the biology and treatment of pancreatic ductal adenocarcinoma. J Hematol Oncol. 2022;15(1):168. 36434634 10.1186/s13045-022-01384-3 PMC9701038

[162] Zhang F , Zhang Q , Zhu J , et al. Integrated proteogenomic characterization across major histological types of pituitary neuroendocrine tumors. Cell Res. 2022;32(12):1047‐1067. 36307579 10.1038/s41422-022-00736-5 PMC9715725

[163] Deng M , Ran P , Chen L , et al. Proteogenomic characterization of cholangiocarcinoma. Hepatology. 2022. 10.1002/hep.32624 PMC9869950 35716043

[164] Xu N , Yao Z , Shang G , et al. Integrated proteogenomic characterization of urothelial carcinoma of the bladder. J Hematol Oncol. 2022;15(1):76. 35659036 10.1186/s13045-022-01291-7 PMC9164575

[165] Yang T , Ren C , Qiao P , et al. PIM2‐mediated phosphorylation of hexokinase 2 is critical for tumor growth and paclitaxel resistance in breast cancer. Oncogene. 2018;37(45):5997‐6009. 29985480 10.1038/s41388-018-0386-x PMC6224402

[166] Li H , Lu S , Chen Y , et al. AKT2 phosphorylation of hexokinase 2 at T473 promotes tumorigenesis and metastasis in colon cancer cells via NF‐κB, HIF1α, MMP2, and MMP9 upregulation. Cell Signal. 2019;58:99‐110. 30877036 10.1016/j.cellsig.2019.03.011

[167] Lee J‐H , Liu R , Li J , et al. Stabilization of phosphofructokinase 1 platelet isoform by AKT promotes tumorigenesis. Nat Commun. 2017;8(1):949. 29038421 10.1038/s41467-017-00906-9 PMC5643558

[168] Houles T , Gravel S‐P , Lavoie G , et al. RSK regulates PFK‐2 activity to promote metabolic rewiring in melanoma. Cancer Res. 2018;78(9):2191‐2204. 29440170 10.1158/0008-5472.CAN-17-2215

[169] Li F‐L , Liu J‐P , Bao R‐X , et al. Acetylation accumulates PFKFB3 in cytoplasm to promote glycolysis and protects cells from cisplatin‐induced apoptosis. Nat Commun. 2018;9(1):508. 29410405 10.1038/s41467-018-02950-5 PMC5802808

[170] Hitosugi T , Kang S , Vander Heiden MG , et al. Tyrosine phosphorylation inhibits PKM2 to promote the Warburg effect and tumor growth. Sci Signal. 2009;2(97):ra73. 19920251 10.1126/scisignal.2000431 PMC2812789

[171] Cai Z , Li C‐F , Han F , et al. Phosphorylation of PDHA by AMPK drives TCA cycle to promote cancer metastasis. Mol Cell. 2020;80(2):263‐278. 33022274 10.1016/j.molcel.2020.09.018 PMC7534735

[172] Butt U , Khan MH , Pouwels J , Westermarck J . SHARPIN S146 phosphorylation mediates ARP2/3 interaction, cancer cell invasion and metastasis. J Cell Sci. 2022;135(20):jcs260627. 36148554 10.1242/jcs.260627

[173] Li GM , Li L , Li MQ , et al. DAPK3 inhibits gastric cancer progression via activation of ULK1‐dependent autophagy. Cell Death Differ. 2021;28(3):952‐967. 33037394 10.1038/s41418-020-00627-5 PMC7937684

[174] Ciraku L , Bacigalupa ZA , Ju J , et al. O‐GlcNAc transferase regulates glioblastoma acetate metabolism via regulation of CDK5‐dependent ACSS2 phosphorylation. Oncogene. 2022;41(14):2122‐2136. 35190642 10.1038/s41388-022-02237-6 PMC9410282

[175] Li Z , Ge Y , Dong J , et al. BZW1 facilitates glycolysis and promotes tumor growth in pancreatic ductal adenocarcinoma through potentiating eIF2α phosphorylation. Gastroenterology. 2022;162(4):1256‐1271. e14. 34951995 10.1053/j.gastro.2021.12.249 PMC9436032

[176] Xiong X , Hasani S , Young LEA , et al. Activation of Drp1 promotes fatty acids‐induced metabolic reprograming to potentiate Wnt signaling in colon cancer. Cell Death Differ. 2022;29(10):1913‐1927. 35332310 10.1038/s41418-022-00974-5 PMC9525627

[177] Huang Y , Liu S , Shan M , et al. RNF12 is regulated by AKT phosphorylation and promotes TGF‐β driven breast cancer metastasis. Cell Death Dis. 2022;13(1):44. 35013159 10.1038/s41419-021-04493-y PMC8748510

[178] Xiong Y , Ju L , Yuan L , et al. KNSTRN promotes tumorigenesis and gemcitabine resistance by activating AKT in bladder cancer. Oncogene. 2021;40(9):1595‐1608. 33452459 10.1038/s41388-020-01634-z

[179] Li M , Zhao X , Yong H , et al. Transketolase promotes colorectal cancer metastasis through regulating AKT phosphorylation. Cell Death Dis. 2022;13(2):99. 35110545 10.1038/s41419-022-04575-5 PMC8810869

[180] Apostolidi M , Vathiotis IA , Muthusamy V , et al. Targeting pyruvate kinase M2 phosphorylation reverses aggressive cancer phenotypes. Cancer Res. 2021;81(16):4346‐4359. 34185676 10.1158/0008-5472.CAN-20-4190 PMC8373815

[181] Zhang K , Zhang D , Wang J , et al. Aloe gel glucomannan induced colon cancer cell death via mitochondrial damage‐driven PINK1/Parkin mitophagy pathway. Carbohydr Polym. 2022;295:119841. 35989033 10.1016/j.carbpol.2022.119841

[182] Miyazaki N , Shiratori R , Oshima T , et al. PINK1‐dependent and Parkin‐independent mitophagy is involved in reprogramming of glycometabolism in pancreatic cancer cells. Biochem Biophys Res Commun. 2022;625:167‐173. 35963163 10.1016/j.bbrc.2022.08.004

[183] Sun X , Shu Y , Ye G , et al. Histone deacetylase inhibitors inhibit cervical cancer growth through Parkin acetylation‐mediated mitophagy. Acta Pharm Sin B. 2022;12(2):838‐852. 35256949 10.1016/j.apsb.2021.07.003 PMC8897022

[184] Gan ZY , Callegari S , Cobbold SA , et al. Activation mechanism of PINK1. Nature. 2022;602(7896):328‐335. 34933320 10.1038/s41586-021-04340-2 PMC8828467

[185] Kazlauskaite A , Martínez‐Torres RJ , Wilkie S , et al. Binding to serine 65‐phosphorylated ubiquitin primes Parkin for optimal PINK1‐dependent phosphorylation and activation. EMBO Rep. 2015;16(8):939‐954. 26116755 10.15252/embr.201540352 PMC4552487

[186] El Manaa W , Duplan E , Goiran T , et al. Transcription‐ and phosphorylation‐dependent control of a functional interplay between XBP1s and PINK1 governs mitophagy and potentially impacts Parkinson disease pathophysiology. Autophagy. 2021;17(12):4363‐4385. 34030589 10.1080/15548627.2021.1917129 PMC8726674

[187] Wahabi K , Perwez A , Rizvi MA . Parkin in Parkinson's disease and cancer: a double‐edged sword. Mol Neurobiol. 2018;55(8):6788‐6800. 29349575 10.1007/s12035-018-0879-1

[188] Xia Y , Prokop S , Giasson BI . “Don't Phos Over Tau”: recent developments in clinical biomarkers and therapies targeting tau phosphorylation in Alzheimer's disease and other tauopathies. Mol Neurodegener. 2021;16(1):37. 34090488 10.1186/s13024-021-00460-5 PMC8180161

[189] Alavi Naini SM , Soussi‐Yanicostas N . Tau hyperphosphorylation and oxidative stress, a critical vicious circle in neurodegenerative tauopathies? Oxid Med Cell Longev. 2015;2015:151979. 26576216 10.1155/2015/151979 PMC4630413

[190] Luo HB , Xia YY , Shu XJ , et al. SUMOylation at K340 inhibits tau degradation through deregulating its phosphorylation and ubiquitination. Proc Natl Acad Sci U S A. 2014;111(46):16586‐16591. 25378699 10.1073/pnas.1417548111 PMC4246270

[191] Signaevsky M , Prastawa M , Farrell K , et al. Artificial intelligence in neuropathology: deep learning‐based assessment of tauopathy. Lab Invest. 2019;99(7):1019‐1029. 30770886 10.1038/s41374-019-0202-4 PMC7684013

[192] Thijssen EH , La Joie R , Strom A , et al. Plasma phosphorylated tau 217 and phosphorylated tau 181 as biomarkers in Alzheimer's disease and frontotemporal lobar degeneration: a retrospective diagnostic performance study. Lancet Neurol. 2021;20(9):739‐752. 34418401 10.1016/S1474-4422(21)00214-3 PMC8711249

[193] Gianotti C , Porta A , De Graan PN , Oestreicher AB , Nunzi MG . B‐50/GAP‐43 phosphorylation in hippocampal slices from aged rats: effects of phosphatidylserine administration. Neurobiol Aging. 1993;14(5):401‐406. 8247222 10.1016/0197-4580(93)90098-v

[194] Zhou Z , Hong EJ , Cohen S , et al. Brain‐specific phosphorylation of MeCP2 regulates activity‐dependent Bdnf transcription, dendritic growth, and spine maturation. Neuron. 2006;52(2):255‐269. 17046689 10.1016/j.neuron.2006.09.037 PMC3962021

[195] Li X , Wang Y , Zhu Q , et al. Epidermal growth factor regulates the development of stem and progenitor Leydig cells in rats. J Cell Mol Med. 2020;24(13):7313‐7330. 32441057 10.1111/jcmm.15302 PMC7339176

[196] Svoboda P , Franke V , Schultz RM . Sculpting the transcriptome during the oocyte‐to‐embryo transition in mouse. Curr Top Dev Biol. 2015;113:305‐349. 26358877 10.1016/bs.ctdb.2015.06.004

[197] Marinis JM , Hutti JE , Homer CR , et al. IκB kinase α phosphorylation of TRAF4 downregulates innate immune signaling. Mol Cell Biol. 2012;32(13):2479‐2489. 22547678 10.1128/MCB.00106-12 PMC3434482

[198] Konno H , Konno K , Barber GN . Cyclic dinucleotides trigger ULK1 (ATG1) phosphorylation of STING to prevent sustained innate immune signaling. Cell. 2013;155(3):688‐698. 24119841 10.1016/j.cell.2013.09.049 PMC3881181

[199] Kawai T , Akira S . Signaling to NF‐kappaB by toll‐like receptors. Trends Mol Med. 2007;13(11):460‐469. 18029230 10.1016/j.molmed.2007.09.002

[200] Zhong B , Yang Y , Li S , et al. The adaptor protein MITA links virus‐sensing receptors to IRF3 transcription factor activation. Immunity. 2008;29(4):538‐550. 18818105 10.1016/j.immuni.2008.09.003

[201] Ritter JL , Zhu Z , Thai TC , et al. Phosphorylation of RAB7 by TBK1/IKKε regulates innate immune signaling in triple‐negative breast cancer. Cancer Res. 2020;80(1):44‐56. 31662325 10.1158/0008-5472.CAN-19-1310 PMC6942622

[202] Tong J , Tan X , Song X , et al. CDK4/6 inhibition suppresses p73 phosphorylation and activates DR5 to potentiate chemotherapy and immune checkpoint blockade. Cancer Res. 2022;82(7):1340‐1352. 35149588 10.1158/0008-5472.CAN-21-3062 PMC8983601

[203] Jing W , Wang G , Cui Z , et al. FGFR3 destabilizes PD‐L1 via NEDD4 to control T‐cell‐mediated bladder cancer immune surveillance. Cancer Res. 2022;82(1):114‐129. 34753771 10.1158/0008-5472.CAN-21-2362

[204] Chen J , Wei X , Wang X , et al. TBK1‐METTL3 axis facilitates antiviral immunity. Cell Rep. 2022;38(7):110373. 35172162 10.1016/j.celrep.2022.110373

[205] Zhang X , Huang X , Xu J , et al. NEK2 inhibition triggers anti‐pancreatic cancer immunity by targeting PD‐L1. Nat Commun. 2021;12(1):4536. 34315872 10.1038/s41467-021-24769-3 PMC8316469

[206] Im E , Chung KC . Dyrk1A phosphorylates parkin at Ser‐131 and negatively regulates its ubiquitin E3 ligase activity. J Neurochem. 2015;134(4):756‐768. 25963095 10.1111/jnc.13164

[207] Gharwan H , Groninger H . Kinase inhibitors and monoclonal antibodies in oncology: clinical implications. Nat Rev Clin Oncol. 2016;13(4):209‐227. 26718105 10.1038/nrclinonc.2015.213

[208] Gild ML , Tsang VHM , Clifton‐Bligh RJ , Robinson BG . Multikinase inhibitors in thyroid cancer: timing of targeted therapy. Nat Rev Endocrinol. 2021;17(4):225‐234. 33603220 10.1038/s41574-020-00465-y

[209] Roskoski R, Jr. Properties of FDA‐approved small molecule protein kinase inhibitors: a 2022 update. Pharmacol Res. 2022;175:106037. 34921994 10.1016/j.phrs.2021.106037

[210] Roskoski R, Jr. Classification of small molecule protein kinase inhibitors based upon the structures of their drug‐enzyme complexes. Pharmacol Res. 2016;103:26‐48. 26529477 10.1016/j.phrs.2015.10.021

[211] Lindauer M , Hochhaus A . Dasatinib. Recent Results Cancer Res. 2014;201:27‐65. 24756784 10.1007/978-3-642-54490-3_2

[212] Huang Z , Xiong Q , Cui Z , et al. Efficacy and safety of crizotinib plus bevacizumab in positive non‐small cell lung cancer: an open‐label, single‐arm, prospective observational study. Am J Transl Res. 2021;13(3):1526‐1534. 33841676 PMC8014364

[213] Hantschel O , Rix U , Superti‐Furga G . Target spectrum of the BCR‐ABL inhibitors imatinib, nilotinib and dasatinib. Leuk Lymphoma. 2008;49(4):615‐619. 18398720 10.1080/10428190801896103

[214] Choi HG , Ren P , Adrian F , et al. A type‐II kinase inhibitor capable of inhibiting the T315I “gatekeeper” mutant of Bcr‐Abl. J Med Chem. 2010;53(15):5439‐5448. 20604564 10.1021/jm901808w PMC4134510

[215] Bhullar KS , Lagarón NO , McGowan EM , et al. Kinase‐targeted cancer therapies: progress, challenges and future directions. Mol Cancer. 2018;17(1):48. 29455673 10.1186/s12943-018-0804-2 PMC5817855

[216] Ibrahim N , Yu Y , Walsh WR , Yang JL . Molecular targeted therapies for cancer: sorafenib mono‐therapy and its combination with other therapies (review). Oncol Rep. 2012;27(5):1303‐1311. 22323095 10.3892/or.2012.1675

[217] Kane RC , Farrell AT , Madabushi R , et al. Sorafenib for the treatment of unresectable hepatocellular carcinoma. Oncologist. 2009;14(1):95‐100. 19144678 10.1634/theoncologist.2008-0185

[218] Iyer R , Fetterly G , Lugade A , Thanavala Y . Sorafenib: a clinical and pharmacologic review. Expert Opin Pharmacother. 2010;11(11):1943‐1955. 20586710 10.1517/14656566.2010.496453

[219] Fasano M , Della Corte CM, Califano R , et al. Type III or allosteric kinase inhibitors for the treatment of non‐small cell lung cancer. Expert Opin Investig Drugs. 2014;23(6):809‐821. 10.1517/13543784.2014.902934 24673358

[220] Schoepfer J , Jahnke W , Berellini G , et al. Discovery of Asciminib (ABL001), an allosteric inhibitor of the tyrosine kinase activity of BCR‐ABL1. J Med Chem. 2018;61(18):8120‐8135. 30137981 10.1021/acs.jmedchem.8b01040

[221] Sharman JP , Egyed M , Jurczak W , et al. Acalabrutinib with or without obinutuzumab versus chlorambucil and obinutuzumab for treatment‐naive chronic lymphocytic leukaemia (ELEVATE‐TN): a randomised, controlled, phase 3 trial. Lancet. 2020;395(10232):1278‐1291. 32305093 10.1016/S0140-6736(20)30262-2 PMC8151619

[222] Deeks ED , Keating GM . Afatinib in advanced NSCLC: a profile of its use. Drugs Ther Perspect. 2018;34(3):89‐98. 29540977 10.1007/s40267-018-0482-6 PMC5840214

[223] Martin M , Holmes FA , Ejlertsen B , et al. Neratinib after trastuzumab‐based adjuvant therapy in HER2‐positive breast cancer (ExteNET): 5‐year analysis of a randomised, double‐blind, placebo‐controlled, phase 3 trial. Lancet Oncol. 2017;18(12):1688‐1700. 29146401 10.1016/S1470-2045(17)30717-9

[224] Bhanumathy KK , Balagopal A , Vizeacoumar FS , Vizeacoumar FJ , Freywald A , Giambra V . Protein tyrosine kinases: their roles and their targeting in leukemia. Cancers (Basel). 2021;13(2):184. 33430292 10.3390/cancers13020184 PMC7825731

[225] Jain N , Keating M , Thompson P , et al. Ibrutinib and venetoclax for first‐line treatment of CLL. N Engl J Med. 2019;380(22):2095‐2103. 31141631 10.1056/NEJMoa1900574 PMC11827445

[226] Jiao Q , Bi L , Ren Y , Song S , Wang Q , Wang YS . Advances in studies of tyrosine kinase inhibitors and their acquired resistance. Mol Cancer. 2018;17(1):36. 29455664 10.1186/s12943-018-0801-5 PMC5817861

[227] He J , Zhou Z , Sun X , et al. The new opportunities in medicinal chemistry of fourth‐generation EGFR inhibitors to overcome C797S mutation. Eur J Med Chem. 2021;210:112995. 33243531 10.1016/j.ejmech.2020.112995

[228] Girard N . Optimizing outcomes in EGFR mutation‐positive NSCLC: which tyrosine kinase inhibitor and when? Future Oncol. 2018;14(11):1117‐1132. 29336166 10.2217/fon-2017-0636

[229] Rosell R , Carcereny E , Gervais R , et al. Erlotinib versus standard chemotherapy as first‐line treatment for European patients with advanced EGFR mutation‐positive non‐small‐cell lung cancer (EURTAC): a multicentre, open‐label, randomised phase 3 trial. Lancet Oncol. 2012;13(3):239‐246. 22285168 10.1016/S1470-2045(11)70393-X

[230] Wang S , Li J . Second‐generation EGFR and ErbB tyrosine kinase inhibitors as first‐line treatments for non‐small cell lung cancer. Onco Targets Ther. 2019;12:6535‐6548. 31496745 10.2147/OTT.S198945 PMC6700283

[231] Eide IJZ , Helland A , Ekman S , et al. Osimertinib in T790M‐positive and ‐negative patients with EGFR‐mutated advanced non‐small cell lung cancer (the TREM‐study). Lung Cancer. 2020;143:27‐35. 32200138 10.1016/j.lungcan.2020.03.009

[232] Melosky B , Hirsh V . Management of common toxicities in metastatic NSCLC related to anti‐lung cancer therapies with EGFR‐TKIs. Front Oncol. 2014;4:238. 25279350 10.3389/fonc.2014.00238 PMC4165207

[233] Fountas A , Diamantopoulos LN , Tsatsoulis A . Tyrosine kinase inhibitors and diabetes: a novel treatment paradigm? Trends Endocrinol Metab. 2015;26(11):643‐656. 26492832 10.1016/j.tem.2015.09.003

[234] Hagerkvist R , Sandler S , Mokhtari D , Welsh N . Amelioration of diabetes by imatinib mesylate (Gleevec): role of beta‐cell NF‐kappaB activation and anti‐apoptotic preconditioning. FASEB J. 2007;21(2):618‐628. 17135364 10.1096/fj.06-6910com

[235] Mukai E , Ohta T , Kawamura H , et al. Enhanced vascular endothelial growth factor signaling in islets contributes to beta cell injury and consequential diabetes in spontaneously diabetic Torii rats. Diabetes Res Clin Pract. 2014;106(2):303‐311. 25262109 10.1016/j.diabres.2014.08.023

[236] Fitter S , Vandyke K , Gronthos S , Zannettino AC . Suppression of PDGF‐induced PI3 kinase activity by imatinib promotes adipogenesis and adiponectin secretion. J Mol Endocrinol. 2012;48(3):229‐240. 22474082 10.1530/JME-12-0003

[237] Prada PO , Ropelle ER , Mourao RH , et al. EGFR tyrosine kinase inhibitor (PD153035) improves glucose tolerance and insulin action in high‐fat diet‐fed mice. Diabetes. 2009;58(12):2910‐2919. 19696185 10.2337/db08-0506 PMC2780887

[238] Lin JJ , Zhu VW , Schoenfeld AJ , et al. Brigatinib in patients with alectinib‐refractory ALK‐positive NSCLC. J Thorac Oncol. 2018;13(10):1530‐1538. 29935304 10.1016/j.jtho.2018.06.005 PMC6341982

[239] Gover‐Proaktor A , Granot G , Pasmanik‐Chor M , et al. Bosutinib, dasatinib, imatinib, nilotinib, and ponatinib differentially affect the vascular molecular pathways and functionality of human endothelial cells. Leuk Lymphoma. 2019;60(1):189‐199. 29741440 10.1080/10428194.2018.1466294

[240] Byrd JC , Wierda WG , Schuh A , et al. Acalabrutinib monotherapy in patients with relapsed/refractory chronic lymphocytic leukemia: updated phase 2 results. Blood. 2020;135(15):1204‐1213. 31876911 10.1182/blood.2018884940 PMC7146022

[241] Abbas HA , Wierda WG . Acalabrutinib: a selective bruton tyrosine kinase inhibitor for the treatment of B‐cell malignancies. Front Oncol. 2021;11:668162. 34055635 10.3389/fonc.2021.668162 PMC8162209

[242] Elisei R , Schlumberger MJ , Müller SP , et al. Cabozantinib in progressive medullary thyroid cancer. J Clin Oncol. 2013;31(29):3639‐3646. 24002501 10.1200/JCO.2012.48.4659 PMC4164813

[243] Moro‐Sibilot D , Cozic N , Pérol M , et al. Crizotinib in c‐MET‐ or ROS1‐positive NSCLC: results of the AcSé phase II trial. Ann Oncol. 2019;30(12):1985‐1991. 31584608 10.1093/annonc/mdz407

[244] Sonpavde G , Hutson TE , Rini BI . Axitinib for renal cell carcinoma. Expert Opin Investig Drugs. 2008;17(5):741‐748. 10.1517/13543784.17.5.741 18447599

[245] Krajewska J , Olczyk T , Jarzab B . Cabozantinib for the treatment of progressive metastatic medullary thyroid cancer. Expert Rev Clin Pharmacol. 2016;9(1):69‐79. 26536165 10.1586/17512433.2016.1102052

[246] Schettino C , Bareschino MA , Ricci V , Ciardiello F . Erlotinib: an EGF receptor tyrosine kinase inhibitor in non‐small‐cell lung cancer treatment. Expert Rev Respir Med. 2008;2(2):167‐178. 20477246 10.1586/17476348.2.2.167

[247] Deremer DL , Ustun C , Natarajan K . Nilotinib: a second‐generation tyrosine kinase inhibitor for the treatment of chronic myelogenous leukemia. Clin Ther. 2008;30(11):1956‐1975. 19108785 10.1016/j.clinthera.2008.11.014

[248] Lee ATJ , Jones RL , Huang PH . Pazopanib in advanced soft tissue sarcomas. Signal Transduct Target Ther. 2019;4:16. 31123606 10.1038/s41392-019-0049-6 PMC6522548

[249] Sternberg CN , Davis ID , Mardiak J , et al. Pazopanib in locally advanced or metastatic renal cell carcinoma: results of a randomized phase III trial. J Clin Oncol. 2010;28(6):1061‐1068. 20100962 10.1200/JCO.2009.23.9764

[250] Benner B , Good L , Quiroga D , et al. Pexidartinib, a novel small molecule CSF‐1R inhibitor in use for tenosynovial giant cell tumor: a systematic review of pre‐clinical and clinical development. Drug Des Devel Ther. 2020;14:1693‐1704. 10.2147/DDDT.S253232 PMC7210448 32440095

[251] Wang JP , Wu C‐Y , Yeh Y‐C , et al. Erlotinib is effective in pancreatic cancer with epidermal growth factor receptor mutations: a randomized, open‐label, prospective trial. Oncotarget. 2015;6(20):18162‐18173. 26046796 10.18632/oncotarget.4216 PMC4627242

[252] Inno A , Di Noia V , Martini M , et al. Erlotinib for patients with EGFR wild‐type metastatic NSCLC: a retrospective biomarkers analysis. Pathol Oncol Res. 2019;25(2):513‐520. 29557085 10.1007/s12253-018-0404-x

[253] Zhang Z , Zeng K , Zhao S , et al. Pemetrexed/carboplatin plus gefitinib as a first‐line treatment for EGFR‐mutant advanced nonsmall cell lung cancer: a Bayesian network meta‐analysis. Ther Adv Med Oncol. 2019;11:1758835919891652. 31908655 10.1177/1758835919891652 PMC6937538

[254] Xuhong J‐C , Qi X‐W , Zhang Y , Jiang J . Mechanism, safety and efficacy of three tyrosine kinase inhibitors lapatinib, neratinib and pyrotinib in HER2‐positive breast cancer. Am J Cancer Res. 2019;9(10):2103‐2119. 31720077 PMC6834479

[255] Roubal K , Myint ZW , Kolesar JM . Erdafitinib: A novel therapy for FGFR‐mutated urothelial cancer. Am J Health Syst Pharm. 2020;77(5):346‐351. 32073123 10.1093/ajhp/zxz329

[256] Wollin L , Wex E , Pautsch A , et al. Mode of action of nintedanib in the treatment of idiopathic pulmonary fibrosis. Eur Respir J. 2015;45(5):1434‐45. 25745043 10.1183/09031936.00174914 PMC4416110

[257] Zhao J , Song Y , Liu D . Gilteritinib: a novel FLT3 inhibitor for acute myeloid leukemia. Biomark Res. 2019;7:19. 31528345 10.1186/s40364-019-0170-2 PMC6737601

[258] Kayser S , Levis MJ , Schlenk RF . Midostaurin treatment in FLT3‐mutated acute myeloid leukemia and systemic mastocytosis. Expert Rev Clin Pharmacol. 2017;10(11):1177‐1189. 28960095 10.1080/17512433.2017.1387051

[259] Valent P , Akin C , Hartmann K , et al. Midostaurin: a magic bullet that blocks mast cell expansion and activation. Ann Oncol. 2017;28(10):2367‐2376. 28945834 10.1093/annonc/mdx290 PMC7115852

[260] Bewersdorf JP , Jaszczur SM , Afifi S , Zhao JC , Zeidan AM . Beyond ruxolitinib: fedratinib and other emergent treatment options for myelofibrosis. Cancer Manag Res. 2019;11:10777‐10790. 31920387 10.2147/CMAR.S212559 PMC6935287

[261] Taylor PC . Clinical efficacy of launched JAK inhibitors in rheumatoid arthritis. Rheumatology (Oxford). 2019;58(Suppl 1):i17‐i26. 30806707 10.1093/rheumatology/key225 PMC6390878

[262] Suttle AB , Ball HA , Molimard M , et al. Relationships between pazopanib exposure and clinical safety and efficacy in patients with advanced renal cell carcinoma. Br J Cancer. 2014;111(10):1909‐1916. 25349968 10.1038/bjc.2014.503 PMC4229638

[263] Koshiyama M , Matsumura N , Baba T , Yamaguchi K , Yoshioka Y , Konishi I . Two cases of recurrent ovarian clear cell carcinoma treated with sorafenib. Cancer Biol Ther. 2014;15(1):22‐25. 24096267 10.4161/cbt.26608 PMC3938519

[264] Luo X‐Y , Wu K‐M , He X‐X . Advances in drug development for hepatocellular carcinoma: clinical trials and potential therapeutic targets. J Exp Clin Cancer Res. 2021;40(1):172. 34006331 10.1186/s13046-021-01968-w PMC8130401

[265] Gan HK , Seruga B , Knox JJ . Sunitinib in solid tumors. Expert Opin Investig Drugs. 2009;18(6):821‐834. 10.1517/13543780902980171 19453268

[266] Roskoski R . Sunitinib: a VEGF and PDGF receptor protein kinase and angiogenesis inhibitor. Biochem Biophys Res Commun. 2007;356(2):323‐328. 17367763 10.1016/j.bbrc.2007.02.156

[267] Felip E , Smit EF , Molina‐Vila MA , et al. Alectinib for the treatment of pretreated RET‐rearranged advanced NSCLC: results of the ETOP ALERT‐lung trial. Lung Cancer. 2022;172:94‐99. 36030612 10.1016/j.lungcan.2022.08.008

[268] Grüllich C . Cabozantinib: multi‐kinase inhibitor of MET, AXL, RET, and VEGFR2. Recent Results Cancer Res. 2018;211:67‐75. 30069760 10.1007/978-3-319-91442-8_5

[269] Cabanillas ME , Habra MA . Lenvatinib: Role in thyroid cancer and other solid tumors. Cancer Treat Rev. 2016;42:47‐55. 26678514 10.1016/j.ctrv.2015.11.003

[270] Tremblay G , Groff M , Iadeluca L , et al. Effectiveness of crizotinib versus entrectinib in ‐positive non‐small‐cell lung cancer using clinical and real‐world data. Future Oncol. 2022;18(17):2063‐2074. 35232230 10.2217/fon-2021-1102

[271] Cortes JE , Khoury HJ , Kantarjian HM , et al. Long‐term bosutinib for chronic phase chronic myeloid leukemia after failure of imatinib plus dasatinib and/or nilotinib. Am J Hematol. 2016;91(12):1206‐1214. 27531525 10.1002/ajh.24536 PMC5303616

[272] Paik J . Fostamatinib: a review in chronic immune thrombocytopenia. Drugs. 2021;81(8):935‐943. 33970459 10.1007/s40265-021-01524-y

[273] Doebele RC , Drilon A , Paz‐Ares L , et al. Entrectinib in patients with advanced or metastatic NTRK fusion‐positive solid tumours: integrated analysis of three phase 1–2 trials. Lancet Oncol. 2020;21(2):271‐282. 31838007 10.1016/S1470-2045(19)30691-6 PMC7461630

[274] Laetsch TW , Hawkins DS . Larotrectinib for the treatment of TRK fusion solid tumors. Expert Rev Anticancer Ther. 2019;19(1):1‐10. 30350734 10.1080/14737140.2019.1538796

[275] Albiges L , Gizzi M , Carton E , Escudier B . Axitinib in metastatic renal cell carcinoma. Expert Rev Anticancer Ther. 2015;15(5):499‐507. 25907705 10.1586/14737140.2015.1033408

[276] Hoy SM . Cabozantinib: a review of its use in patients with medullary thyroid cancer. Drugs. 2014;74(12):1435‐1444. 25056653 10.1007/s40265-014-0265-x

[277] Deng S , Solinas A , Calvisi DF . Cabozantinib for HCC treatment, from clinical back to experimental models. Front Oncol. 2021;11:756672. 34722310 10.3389/fonc.2021.756672 PMC8548824

[278] Tannir NM , Schwab G , Grünwald V . Cabozantinib: an active novel multikinase inhibitor in renal cell carcinoma. Curr Oncol Rep. 2017;19(2):14. 28247252 10.1007/s11912-017-0566-9 PMC5331092

[279] Zschäbitz S , Grüllich C . Lenvantinib: a tyrosine kinase inhibitor of VEGFR 1–3, FGFR 1–4, PDGFRα, KIT and RET. Recent Results Cancer Res. 2018;211:187‐198. 30069768 10.1007/978-3-319-91442-8_13

[280] Drabkin HA . Pazopanib and anti‐VEGF therapy. Open Access J Urol. 2010;2:35‐40. 24198612 PMC3818876

[281] Martinelli E , Troiani T , Morgillo F , et al. Emerging VEGF‐receptor inhibitors for colorectal cancer. Expert Opin Emerg Drugs. 2013;18(1):25‐37. 23216053 10.1517/14728214.2013.749856

[282] Flaherty KT . Sorafenib in renal cell carcinoma. Clin Cancer Res. 2007;13(2 Pt 2):747s‐752s. 17255304 10.1158/1078-0432.CCR-06-2063

[283] Llovet JM , Ricci S , Mazzaferro V , et al. Sorafenib in advanced hepatocellular carcinoma. N Engl J Med. 2008;359(4):378‐390. 18650514 10.1056/NEJMoa0708857

[284] Krajewska J , Handkiewicz‐Junak D , Jarzab B . Sorafenib for the treatment of thyroid cancer: an updated review. Expert Opin Pharmacother. 2015;16(4):573‐583. 25605317 10.1517/14656566.2015.1005601

[285] Hopkins TG , Marples M , Stark D . Sunitinib in the management of gastrointestinal stromal tumours (GISTs). Eur J Surg Oncol. 2008;34(8):844‐850. 18082353 10.1016/j.ejso.2007.10.011

[286] Rizzo M , Porta C . Sunitinib in the treatment of renal cell carcinoma: an update on recent evidence. Ther Adv Urol. 2017;9(8):195‐207. 29662544 10.1177/1756287217713902 PMC5896861

[287] Delbaldo C , Faivre S , Dreyer C , Raymond E . Sunitinib in advanced pancreatic neuroendocrine tumors: latest evidence and clinical potential. Ther Adv Med Oncol. 2012;4(1):9‐18. 22229044 10.1177/1758834011428147 PMC3244202

[288] Chau NG , Haddad RI . Vandetanib for the treatment of medullary thyroid cancer. Clin Cancer Res. 2013;19(3):524‐529. 23231950 10.1158/1078-0432.CCR-12-2353

[289] Rutkowski P , Blank C . Dabrafenib for the treatment of BRAF V600‐positive melanoma: a safety evaluation. Expert Opin Drug Saf. 2014;13(9):1249‐1258. 25014231 10.1517/14740338.2014.939954

[290] Planchard D , Kim TM , Mazieres J , et al. Dabrafenib in patients with BRAF(V600E)‐positive advanced non‐small‐cell lung cancer: a single‐arm, multicentre, open‐label, phase 2 trial. Lancet Oncol. 2016;17(5):642‐650. 27080216 10.1016/S1470-2045(16)00077-2 PMC5006181

[291] Liao Y , Gao Y , Chang A , et al. Melatonin synergizes BRAF‐targeting agent dabrafenib for the treatment of anaplastic thyroid cancer by inhibiting AKT/hTERT signalling. J Cell Mol Med. 2020;24(20):12119‐12130. 32935463 10.1111/jcmm.15854 PMC7579709

[292] Dummer R , Ascierto PA , Gogas HJ , et al. Encorafenib plus binimetinib versus vemurafenib or encorafenib in patients with BRAF‐mutant melanoma (COLUMBUS): a multicentre, open‐label, randomised phase 3 trial. Lancet Oncol. 2018;19(5):603‐615. 29573941 10.1016/S1470-2045(18)30142-6

[293] Foukakis T . Ribociclib in premenopausal women with advanced breast cancer. Lancet Oncol. 2018;19(7):850‐852. 29804904 10.1016/S1470-2045(18)30367-X

[294] Sendur MAN , Zengin N , Aksoy S , Altundag K . Everolimus: a new hope for patients with breast cancer. Curr Med Res Opin. 2014;30(1):75‐87. 24050600 10.1185/03007995.2013.846253

[295] Yao JC , Shah MH , Ito T , et al. Everolimus for advanced pancreatic neuroendocrine tumors. N Engl J Med. 2011;364(6):514‐523. 21306238 10.1056/NEJMoa1009290 PMC4208619

[296] Voss MH , Molina AM , Motzer RJ . mTOR inhibitors in advanced renal cell carcinoma. Hematol Oncol Clin North Am. 2011;25(4):835‐852. 21763970 10.1016/j.hoc.2011.04.008 PMC3587783

[297] Bissler JJ , Kingswood JC , Radzikowska E , et al. Everolimus for angiomyolipoma associated with tuberous sclerosis complex or sporadic lymphangioleiomyomatosis (EXIST‐2): a multicentre, randomised, double‐blind, placebo‐controlled trial. Lancet. 2013;381(9869):817‐824. 23312829 10.1016/S0140-6736(12)61767-X

[298] Franz DN , Agricola K , Mays M , et al. Everolimus for subependymal giant cell astrocytoma: 5‐year final analysis. Ann Neurol. 2015;78(6):929‐938. 26381530 10.1002/ana.24523 PMC5063160

[299] Ponticelli C . The pros and the cons of mTOR inhibitors in kidney transplantation. Expert Rev Clin Immunol. 2014;10(2):295‐305. 24377908 10.1586/1744666X.2014.872562

[300] Freitas CSG , Baldi BG , Araújo MS , Heiden GI , Kairalla RA , Carvalho CRR . Use of sirolimus in the treatment of lymphangioleiomyomatosis: favorable responses in patients with different extrapulmonary manifestations. J Bras Pneumol. 2015;41(3):275‐280. 26176526 10.1590/S1806-37132015000004553 PMC4541764

[301] Bergmann L , Maute L , Guschmann M . Temsirolimus for advanced renal cell carcinoma. Expert Rev Anticancer Ther. 2014;14(1):9‐21. 24313573 10.1586/14737140.2014.864562

[302] Villegas NC , Lee W‐S . Effectiveness of netarsudil as an additional therapy for glaucoma in patients already on maximally tolerated medical therapy. Clin Ophthalmol. 2021;15:4367‐4372. 34754176 10.2147/OPTH.S337105 PMC8572117

[303] van Herpen CML , Agarwala SS , Hauschild A , et al. Biomarker results from a phase II study of MEK1/2 inhibitor binimetinib (MEK162) in patients with advanced ‐ or ‐mutated melanoma. Oncotarget. 2019;10(19):1850‐1859. 30956763 10.18632/oncotarget.26753 PMC6442999

[304] Lian T , Li C , Wang H . Trametinib in the treatment of multiple malignancies harboring MEK1 mutations. Cancer Treat Rev. 2019;81:101907. 31715422 10.1016/j.ctrv.2019.101907

[305] Aksnes H , Ree R , Arnesen T . Co‐translational, post‐translational, and non‐catalytic roles of N‐terminal acetyltransferases. Mol Cell. 2019;73(6):1097‐1114. 30878283 10.1016/j.molcel.2019.02.007 PMC6962057

[306] Pehar M , Puglielli L . Lysine acetylation in the lumen of the ER: a novel and essential function under the control of the UPR. Biochim Biophys Acta. 2013;1833(3):686‐697. 23247107 10.1016/j.bbamcr.2012.12.004 PMC3556210

[307] Varland S , Osberg C , Arnesen T . N‐terminal modifications of cellular proteins: the enzymes involved, their substrate specificities and biological effects. Proteomics. 2015;15(14):2385‐2401. 25914051 10.1002/pmic.201400619 PMC4692089

[308] Choudhary C , Weinert BT , Nishida Y , Verdin E , Mann M . The growing landscape of lysine acetylation links metabolism and cell signalling. Nat Rev Mol Cell Biol. 2014;15(8):536‐550. 25053359 10.1038/nrm3841

[309] Li P , Ge J , Li H . Lysine acetyltransferases and lysine deacetylases as targets for cardiovascular disease. Nat Rev Cardiol. 2020;17(2):96‐115. 31350538 10.1038/s41569-019-0235-9

[310] Di Martile M , Del Bufalo D , Trisciuoglio D . The multifaceted role of lysine acetylation in cancer: prognostic biomarker and therapeutic target. Oncotarget. 2016;7(34):55789‐55810. 27322556 10.18632/oncotarget.10048 PMC5342454

[311] Verdin E , Ott M . 50 years of protein acetylation: from gene regulation to epigenetics, metabolism and beyond. Nat Rev Mol Cell Biol. 2015;16(4):258‐264. 25549891 10.1038/nrm3931

[312] Cameron AM , Lawless SJ , Pearce EJ . Metabolism and acetylation in innate immune cell function and fate. Semin Immunol. 2016;28(5):408‐416. 28340958 10.1016/j.smim.2016.10.003 PMC10911065

[313] Ali I , Conrad RJ , Verdin E , Ott M . Lysine acetylation goes global: from epigenetics to metabolism and therapeutics. Chem Rev. 2018;118(3):1216‐1252. 29405707 10.1021/acs.chemrev.7b00181 PMC6609103

[314] Fang Z , Wang X , Sun X , Hu W , Miao QR . The role of histone protein acetylation in regulating endothelial function. Front Cell Dev Biol. 2021;9:672447. 33996829 10.3389/fcell.2021.672447 PMC8113824

[315] Zhu X , Liu B , Carlsten JO , et al. Mediator influences telomeric silencing and cellular life span. Mol Cell Biol. 2011;31(12):2413‐2421. 21482672 10.1128/MCB.05242-11 PMC3133415

[316] Allahverdi A , Yang R , Korolev N , et al. The effects of histone H4 tail acetylations on cation‐induced chromatin folding and self‐association. Nucleic Acids Res. 2011;39(5):1680‐1691. 21047799 10.1093/nar/gkq900 PMC3061077

[317] Goodman RH , Smolik S . CBP/p300 in cell growth, transformation, and development. Genes Dev. 2000;14(13):1553‐1577. 10887150

[318] Viziteu E , Grandmougin C , Goldschmidt H , et al. Chetomin, targeting HIF‐1alpha/p300 complex, exhibits antitumour activity in multiple myeloma. Br J Cancer. 2016;114(5):519‐523. 26867162 10.1038/bjc.2016.20 PMC4782210

[319] Ogiwara H , Sasaki M , Mitachi T , et al. Targeting p300 addiction in CBP‐deficient cancers causes synthetic lethality by apoptotic cell death due to abrogation of MYC expression. Cancer Discov. 2016;6(4):430‐445. 26603525 10.1158/2159-8290.CD-15-0754

[320] Menzies KJ , Zhang H , Katsyuba E , Auwerx J . Protein acetylation in metabolism ‐ metabolites and cofactors. Nat Rev Endocrinol. 2016;12(1):43‐60. 26503676 10.1038/nrendo.2015.181

[321] Zhao S , Xu W , Jiang W , et al. Regulation of cellular metabolism by protein lysine acetylation. Science. 2010;327(5968):1000‐1004. 20167786 10.1126/science.1179689 PMC3232675

[322] Qiu X , Brown K , Hirschey MD , Verdin E , Chen D . Calorie restriction reduces oxidative stress by SIRT3‐mediated SOD2 activation. Cell Metab. 2010;12(6):662‐667. 21109198 10.1016/j.cmet.2010.11.015

[323] Chen Y , Zhang J , Lin Y , et al. Tumour suppressor SIRT3 deacetylates and activates manganese superoxide dismutase to scavenge ROS. EMBO Rep. 2011;12(6):534‐541. 21566644 10.1038/embor.2011.65 PMC3128277

[324] Yu W , Dittenhafer‐Reed KE , Denu JM . SIRT3 protein deacetylates isocitrate dehydrogenase 2 (IDH2) and regulates mitochondrial redox status. J Biol Chem. 2012;287(17):14078‐14086. 22416140 10.1074/jbc.M112.355206 PMC3340192

[325] Zou X , Zhu Y , Park SH , et al. SIRT3‐mediated dimerization of IDH2 directs cancer cell metabolism and tumor growth. Cancer Res. 2017;77(15):3990‐3999. 28536275 10.1158/0008-5472.CAN-16-2393 PMC5540757

[326] Wang YP , Zhou LS , Zhao YZ , et al. Regulation of G6PD acetylation by SIRT2 and KAT9 modulates NADPH homeostasis and cell survival during oxidative stress. EMBO J. 2014;33(12):1304‐1320. 24769394 10.1002/embj.201387224 PMC4194121

[327] Lin HP , Cheng ZL , He RY , et al. Destabilization of fatty acid synthase by acetylation inhibits de novo lipogenesis and tumor cell growth. Cancer Res. 2016;76(23):6924‐6936. 27758890 10.1158/0008-5472.CAN-16-1597 PMC5135623

[328] Min SW , Cho SH , Zhou Y , et al. Acetylation of tau inhibits its degradation and contributes to tauopathy. Neuron. 2010;67(6):953‐966. 20869593 10.1016/j.neuron.2010.08.044 PMC3035103

[329] Xu Y , Wan W . Acetylation in the regulation of autophagy. Autophagy. 2022:1‐9. 10.1080/15548627.2022.2062112 PMC9851266 35435793

[330] Son SM , Park SJ , Fernandez‐Estevez M , Rubinsztein DC . Autophagy regulation by acetylation‐implications for neurodegenerative diseases. Exp Mol Med. 2021;53(1):30‐41. 33483607 10.1038/s12276-021-00556-4 PMC8080689

[331] Son SM , Park SJ , Stamatakou E , Vicinanza M , Menzies FM , Rubinsztein DC . Leucine regulates autophagy via acetylation of the mTORC1 component raptor. Nat Commun. 2020;11(1):3148. 32561715 10.1038/s41467-020-16886-2 PMC7305105

[332] Bi G , Jiang G . The molecular mechanism of HDAC inhibitors in anticancer effects. Cell Mol Immunol. 2006;3(4):285‐290. 16978537

[333] Sen P , Lan Y , Li CY , et al. Histone Acetyltransferase p300 Induces De Novo Super‐Enhancers to Drive Cellular Senescence. Molecular cell. 2019;73(4):684‐698. e8. 30773298 10.1016/j.molcel.2019.01.021 PMC6688479

[334] Peleg S , Feller C , Ladurner AG , Imhof A . The metabolic impact on histone acetylation and transcription in ageing. Trends Biochem Sci. 2016;41(8):700‐711. 27283514 10.1016/j.tibs.2016.05.008

[335] Lavu S , Boss O , Elliott PJ , Lambert PD . Sirtuins–novel therapeutic targets to treat age‐associated diseases. Nat Rev Drug Discov. 2008;7(10):841‐853. 18827827 10.1038/nrd2665

[336] Ansari A , Rahman MS , Saha SK , Saikot FK , Deep A , Kim KH . Function of the SIRT3 mitochondrial deacetylase in cellular physiology, cancer, and neurodegenerative disease. Aging Cell. 2017;16(1):4‐16. 27686535 10.1111/acel.12538 PMC5242307

[337] Podobinska M , Szablowska‐Gadomska I , Augustyniak J , Sandvig I , Sandvig A , Buzanska L . Epigenetic modulation of stem cells in neurodevelopment: the role of methylation and acetylation. Front Cell Neurosci. 2017;11:23. 28223921 10.3389/fncel.2017.00023 PMC5293809

[338] Tang T , Zhang Y , Wang Y , et al. HDAC1 and HDAC2 regulate intermediate progenitor positioning to safeguard neocortical development. Neuron. 2019;101(6):1117‐1133. 30709655 10.1016/j.neuron.2019.01.007

[339] Stengel KR , Zhao Y , Klus NJ , et al. Histone deacetylase 3 is required for efficient T cell development. Mol Cell Biol. 2015;35(22):3854‐3865. 26324326 10.1128/MCB.00706-15 PMC4609739

[340] Stengel KR , Bhaskara S , Wang J , et al. Histone deacetylase 3 controls a transcriptional network required for B cell maturation. Nucleic Acids Res. 2019;47(20):10612‐10627. 31586401 10.1093/nar/gkz816 PMC6847391

[341] Goldfarb Y , Kadouri N , Levi B , et al. HDAC3 is a master regulator of mTEC development. Cell Rep. 2016;15(3):651‐665. 27068467 10.1016/j.celrep.2016.03.048 PMC5849426

[342] Witkowski JM , Bryl E , Fulop T . Proteodynamics and aging of eukaryotic cells. Mech Ageing Dev. 2021;194:111430. 33421431 10.1016/j.mad.2021.111430

[343] Zhu D , Wu X , Zhou J , et al. NuRD mediates mitochondrial stress‐induced longevity via chromatin remodeling in response to acetyl‐CoA level. Sci Adv. 2020;6(31):eabb2529. 32789178 10.1126/sciadv.abb2529 PMC7400466

[344] Hwangbo DS , Lee HY , Abozaid LS , Min KJ . Mechanisms of lifespan regulation by calorie restriction and intermittent fasting in model organisms. Nutrients. 2020;12(4):1194. 32344591 10.3390/nu12041194 PMC7230387

[345] Bradshaw PC . Acetyl‐CoA metabolism and histone acetylation in the regulation of aging and lifespan. Antioxidants (Basel). 2021;10(4):572. 33917812 10.3390/antiox10040572 PMC8068152

[346] Madeo F , Carmona‐Gutierrez D , Hofer SJ , Kroemer G . Caloric restriction mimetics against age‐associated disease: targets, mechanisms, and therapeutic potential. Cell Metab. 2019;29(3):592‐610. 30840912 10.1016/j.cmet.2019.01.018

[347] Zhang W , Qu J , Liu GH , Belmonte JCI . The ageing epigenome and its rejuvenation. Nat Rev Mol Cell Biol. 2020;21(3):137‐150. 32020082 10.1038/s41580-019-0204-5

[348] Lin SJ , Defossez PA , Guarente L . Requirement of NAD and SIR2 for life‐span extension by calorie restriction in Saccharomyces cerevisiae. Science. 2000;289(5487):2126‐2128. 11000115 10.1126/science.289.5487.2126

[349] Satoh A , Imai SI , Guarente L . The brain, sirtuins, and ageing. Nat Rev Neurosci. 2017;18(6):362‐374. 28515492 10.1038/nrn.2017.42

[350] Luo C , Ding W , Zhu S , Chen Y , Liu X , Deng H . Nicotinamide mononucleotide administration amends protein acetylome of aged mouse liver. Cells. 2022;11(10):1654. 35626691 10.3390/cells11101654 PMC9139684

[351] Cheng H , Xuan H , Green CD , et al. Repression of human and mouse brain inflammaging transcriptome by broad gene‐body histone hyperacetylation. Proc Natl Acad Sci U S A. 2018;115(29):7611‐7616. 29967166 10.1073/pnas.1800656115 PMC6055154

[352] He M , Chiang HH , Luo H , et al. An acetylation switch of the NLRP3 inflammasome regulates aging‐associated chronic inflammation and insulin resistance. Cell Metab. 2020;31(3):580‐591. e5. 32032542 10.1016/j.cmet.2020.01.009 PMC7104778

[353] Pouikli A , Parekh S , Maleszewska M , et al. Chromatin remodeling due to degradation of citrate carrier impairs osteogenesis of aged mesenchymal stem cells. Nature Aging. 2021;1(9):810‐825. 37117628 10.1038/s43587-021-00105-8 PMC10154229

[354] Yeo D , Kang C , Ji LL . Aging alters acetylation status in skeletal and cardiac muscles. Geroscience. 2020;42(3):963‐976. 32300965 10.1007/s11357-020-00171-7 PMC7286993

[355] Wang W , Zheng Y , Sun S , et al. A genome‐wide CRISPR‐based screen identifies KAT7 as a driver of cellular senescence. Sci Transl Med. 2021;13(575):eabd2655. 33408182 10.1126/scitranslmed.abd2655

[356] Yan P , Li Z , Xiong J , et al. LARP7 ameliorates cellular senescence and aging by allosterically enhancing SIRT1 deacetylase activity. Cell Rep. 2021;37(8):110038. 34818543 10.1016/j.celrep.2021.110038

[357] Jiang W , Wang S , Xiao M , et al. Acetylation regulates gluconeogenesis by promoting PEPCK1 degradation via recruiting the UBR5 ubiquitin ligase. Mol Cell. 2011;43(1):33‐44. 21726808 10.1016/j.molcel.2011.04.028 PMC3962309

[358] Li C , Xu J , Yu Q , et al. Mutation of the novel acetylation site at K414R of BECN1 is involved in adipocyte differentiation and lipolysis. J Cell Mol Med. 2021;25(14):6855‐6863. 34085745 10.1111/jcmm.16692 PMC8278081

[359] Kumar S , Kim YR , Vikram A , et al. Sirtuin1‐regulated lysine acetylation of p66Shc governs diabetes‐induced vascular oxidative stress and endothelial dysfunction. Proc Natl Acad Sci U S A. 2017;114(7):1714‐1719. 28137876 10.1073/pnas.1614112114 PMC5321021

[360] Kosanam H , Thai K , Zhang Y , et al. Diabetes induces lysine acetylation of intermediary metabolism enzymes in the kidney. Diabetes. 2014;63(7):2432‐2439. 24677711 10.2337/db12-1770

[361] Thorwald MA , Godoy‐Lugo JA , Rodriguez R , et al. Cardiac NF‐kappaB acetylation increases while Nrf2‐related gene expression and mitochondrial activity are impaired during the progression of diabetes in UCD‐T2DM rats. Antioxidants (Basel). 2022;11(5):927. 35624791 10.3390/antiox11050927 PMC9137621

[362] Lu J , Huang Y , Zhang X , Xu Y , Nie S . Noncoding RNAs involved in DNA methylation and histone methylation, and acetylation in diabetic vascular complications. Pharmacol Res. 2021;170:105520. 33639232 10.1016/j.phrs.2021.105520

[363] Karbasforooshan H , Karimi G . The role of SIRT1 in diabetic retinopathy. Biomed Pharmacother. 2018;97:190‐194. 29091865 10.1016/j.biopha.2017.10.075

[364] Lu J , McKinsey TA , Nicol RL , Olson EN . Signal‐dependent activation of the MEF2 transcription factor by dissociation from histone deacetylases. Proc Natl Acad Sci U S A. 2000;97(8):4070‐4075. 10737771 10.1073/pnas.080064097 PMC18151

[365] Czubryt MP , McAnally J , Fishman GI , Olson EN . Regulation of peroxisome proliferator‐activated receptor gamma coactivator 1 alpha (PGC‐1 alpha) and mitochondrial function by MEF2 and HDAC5. Proc Natl Acad Sci U S A. 2003;100(4):1711‐1716. 12578979 10.1073/pnas.0337639100 PMC149898

[366] Drazic A , Myklebust LM , Ree R , Arnesen T . The world of protein acetylation. Biochim Biophys Acta. 2016;1864(10):1372‐1401. 27296530 10.1016/j.bbapap.2016.06.007

[367] Mosley AL , Ozcan S . Glucose regulates insulin gene transcription by hyperacetylation of histone h4. J Biol Chem. 2003;278(22):19660‐19666. 12665509 10.1074/jbc.M212375200

[368] Moynihan KA , Grimm AA , Plueger MM , et al. Increased dosage of mammalian Sir2 in pancreatic beta cells enhances glucose‐stimulated insulin secretion in mice. Cell Metab. 2005;2(2):105‐117. 16098828 10.1016/j.cmet.2005.07.001

[369] Yuan ZL , Guan YJ , Chatterjee D , Chin YE . Stat3 dimerization regulated by reversible acetylation of a single lysine residue. Science. 2005;307(5707):269‐273. 15653507 10.1126/science.1105166

[370] Dubois‐Deruy E , El Masri Y , Turkieh A , Amouyel P , Pinet F , Annicotte JS . Cardiac acetylation in metabolic diseases. Biomedicines. 2022;10(8):1834. 36009379 10.3390/biomedicines10081834 PMC9405459

[371] Narita T , Weinert BT , Choudhary C . Functions and mechanisms of non‐histone protein acetylation. Nat Rev Mol Cell Biol. 2019;20(3):156‐174. 30467427 10.1038/s41580-018-0081-3

[372] Liu Y , Yang H , Liu X , Gu H , Li Y , Sun C . Protein acetylation: a novel modus of obesity regulation. J Mol Med (Berl). 2021;99(9):1221‐1235. 34061242 10.1007/s00109-021-02082-2

[373] Lee JE , Schmidt H , Lai B , Ge K . Transcriptional and epigenomic regulation of adipogenesis. Mol Cell Biol. 2019;39(11):e00601. 30936246 10.1128/MCB.00601-18 PMC6517598

[374] Ong BX , Brunmeir R , Zhang Q , et al. Regulation of thermogenic adipocyte differentiation and adaptive thermogenesis through histone acetylation. Front Endocrinol (Lausanne). 2020;11:95. 32174890 10.3389/fendo.2020.00095 PMC7057231

[375] Zhou Y , Peng J , Jiang S . Role of histone acetyltransferases and histone deacetylases in adipocyte differentiation and adipogenesis. Eur J Cell Biol. 2014;93(4):170‐177. 24810880 10.1016/j.ejcb.2014.03.001

[376] Duan Q , Mao X , Xiao Y , et al. Super enhancers at the miR‐146a and miR‐155 genes contribute to self‐regulation of inflammation. Biochim Biophys Acta. 2016;1859(4):564‐571. 26855180 10.1016/j.bbagrm.2016.02.004

[377] Li Q , Peng H , Fan H , et al. The LIM protein Ajuba promotes adipogenesis by enhancing PPARγ and p300/CBP interaction. Cell Death Differ. 2016;23(1):158‐168. 26113042 10.1038/cdd.2015.83 PMC4815986

[378] Takahashi N , Kawada T , Yamamoto T , et al. Overexpression and ribozyme‐mediated targeting of transcriptional coactivators CREB‐binding protein and p300 revealed their indispensable roles in adipocyte differentiation through the regulation of peroxisome proliferator‐activated receptor gamma. J Biol Chem. 2002;277(19):16906‐16912. 11884404 10.1074/jbc.M200585200

[379] Lai B , Lee JE , Jang Y , Wang L , Peng W , Ge K . MLL3/MLL4 are required for CBP/p300 binding on enhancers and super‐enhancer formation in brown adipogenesis. Nucleic Acids Res. 2017;45(11):6388‐6403. 28398509 10.1093/nar/gkx234 PMC5499743

[380] Brown JD , Feldman ZB , Doherty SP , et al. BET bromodomain proteins regulate enhancer function during adipogenesis. Proc Natl Acad Sci U S A. 2018;115(9):2144‐2149. 29444854 10.1073/pnas.1711155115 PMC5834672

[381] Namwanje M , Liu L , Chan M , Aaron N , Kraakman MJ , Qiang L . The depot‐specific and essential roles of CBP/p300 in regulating adipose plasticity. J Endocrinol. 2019;240(2):257‐269. 30530904 10.1530/JOE-18-0361 PMC6813822

[382] Gao S , Yang Q , Peng Y , et al. SIRT6 regulates obesity‐induced oxidative stress via ENDOG/SOD2 signaling in the heart. Cell Biol Toxicol. 2022. 10.1007/s10565-022-09735-z 35798905

[383] Louvet L , Leterme D , Delplace S , et al. Sirtuin 1 deficiency decreases bone mass and increases bone marrow adiposity in a mouse model of chronic energy deficiency. Bone. 2020;136:115361. 32289519 10.1016/j.bone.2020.115361

[384] Chatterjee TK , Idelman G , Blanco V , et al. Histone deacetylase 9 is a negative regulator of adipogenic differentiation. J Biol Chem. 2011;286(31):27836‐27847. 21680747 10.1074/jbc.M111.262964 PMC3149373

[385] Kuzmochka C , Abdou HS , Haché RJ , Atlas E . Inactivation of histone deacetylase 1 (HDAC1) but not HDAC2 is required for the glucocorticoid‐dependent CCAAT/enhancer‐binding protein α (C/EBPα) expression and preadipocyte differentiation. Endocrinology. 2014;155(12):4762‐4773. 25203139 10.1210/en.2014-1565

[386] Wallner M , Eaton DM , Berretta RM , et al. HDAC inhibition improves cardiopulmonary function in a feline model of diastolic dysfunction. Sci Transl Med. 2020;12(525):eaay7205. 31915304 10.1126/scitranslmed.aay7205 PMC7065257

[387] Yusoff SI , Roman M , Lai FY , et al. Systematic review and meta‐analysis of experimental studies evaluating the organ protective effects of histone deacetylase inhibitors. Transl Res. 2019;205:1‐16. 30528323 10.1016/j.trsl.2018.11.002 PMC6386580

[388] McKinsey TA , Olson EN . Toward transcriptional therapies for the failing heart: chemical screens to modulate genes. J Clin Invest. 2005;115(3):538‐546. 15765135 10.1172/JCI24144 PMC1052006

[389] Hsu A , Duan Q , McMahon S , et al. Salt‐inducible kinase 1 maintains HDAC7 stability to promote pathologic cardiac remodeling. J Clin Invest. 2020;130(6):2966‐2977. 32106109 10.1172/JCI133753 PMC7259992

[390] Lee GH , Hoang TH , Jung ES , et al. Anthocyanins attenuate endothelial dysfunction through regulation of uncoupling of nitric oxide synthase in aged rats. Aging Cell. 2020;19(12):e13279. 33274583 10.1111/acel.13279 PMC7744959

[391] Zhao Y , Ling S , Zhong G , et al. Casein kinase‐2 interacting protein‐1 regulates physiological cardiac hypertrophy inhibition of histone deacetylase 4 phosphorylation. Front Physiol. 2021;12:678863. 34211403 10.3389/fphys.2021.678863 PMC8239235

[392] Evans LW , Bender A , Burnett L , et al. Emodin and emodin‐rich rhubarb inhibits histone deacetylase (HDAC) activity and cardiac myocyte hypertrophy. J Nutr Biochem. 2020;79:108339. 32007664 10.1016/j.jnutbio.2019.108339 PMC7162729

[393] Na J , Jin H , Wang X , et al. The crosstalk of HDAC3, microRNA‐18a and ADRB3 in the progression of heart failure. Cell Biosci. 2021;11(1):31. 33549119 10.1186/s13578-020-00523-y PMC7866688

[394] Zhao T , Kee HJ , Bai L , Kim M‐K , Kee S‐J , Jeong MH . Selective HDAC8 inhibition attenuates isoproterenol‐induced cardiac hypertrophy and fibrosis via p38 MAPK pathway. Front Pharmacol. 2021;12:677757. 33959033 10.3389/fphar.2021.677757 PMC8093872

[395] Zhao T , Kee HJ , Kee S‐J , Jeong MH . Hdac8 inhibitor alleviates transverse aortic constriction‐induced heart failure in mice by downregulating Ace1. Oxid Med Cell Longev. 2022;2022:6227330. 35126818 10.1155/2022/6227330 PMC8813277

[396] Zhang H‐N , Dai Y , Zhang C‐H , et al. Sirtuins family as a target in endothelial cell dysfunction: implications for vascular ageing. Biogerontology. 2020;21(5):495‐516. 32285331 10.1007/s10522-020-09873-z

[397] Fan S , Hu Y , You Y , et al. Role of resveratrol in inhibiting pathological cardiac remodeling. Front Pharmacol. 2022;13:924473. 36120366 10.3389/fphar.2022.924473 PMC9475218

[398] Miyamoto S , Kawamura T , Morimoto T , et al. Histone acetyltransferase activity of p300 is required for the promotion of left ventricular remodeling after myocardial infarction in adult mice in vivo. Circulation. 2006;113(5):679‐690. 16461841 10.1161/CIRCULATIONAHA.105.585182

[399] Chelladurai P , Boucherat O , Stenmark K , et al. Targeting histone acetylation in pulmonary hypertension and right ventricular hypertrophy. Br J Pharmacol. 2021;178(1):54‐71. 31749139 10.1111/bph.14932

[400] Shin MK , Vazquez‐Rosa E , Koh Y , et al. Reducing acetylated tau is neuroprotective in brain injury. Cell. 2021;184(10):2715‐2732. e23. 33852912 10.1016/j.cell.2021.03.032 PMC8491234

[401] Ganai SA , Banday S , Farooq Z , Altaf M . Modulating epigenetic HAT activity for reinstating acetylation homeostasis: a promising therapeutic strategy for neurological disorders. Pharmacol Ther. 2016;166:106‐122. 27411674 10.1016/j.pharmthera.2016.07.001

[402] Gräff J , Rei D , Guan J‐S , et al. An epigenetic blockade of cognitive functions in the neurodegenerating brain. Nature. 2012;483(7388):222‐226. 22388814 10.1038/nature10849 PMC3498952

[403] Wu Y , Wang R , Liu R , Ba Y , Huang H . The roles of histone modifications in metal‐induced neurological disorders. Biol Trace Elem Res. 2023;201(1):31‐40. 35129806 10.1007/s12011-022-03134-5

[404] Pirooznia SK , Sarthi J , Johnson AA , et al. Tip60 HAT activity mediates APP induced lethality and apoptotic cell death in the CNS of a Drosophila Alzheimer's disease model. PloS One. 2012;7(7):e41776. 22848598 10.1371/journal.pone.0041776 PMC3406101

[405] Singh AK , Neo SH , Liwang C , et al. Glucose derived carbon nanosphere (CSP) conjugated TTK21, an activator of the histone acetyltransferases CBP/p300, ameliorates amyloid‐beta 1–42 induced deficits in plasticity and associativity in hippocampal CA1 pyramidal neurons. Aging Cell. 2022;21(9):e13675. 35962576 10.1111/acel.13675 PMC9470894

[406] Mahady L , Nadeem M , Malek‐Ahmadi M , Chen K , Perez SE , Mufson EJ . HDAC2 dysregulation in the nucleus basalis of Meynert during the progression of Alzheimer's disease. Neuropathol Appl Neurobiol. 2019;45(4):380‐397. 30252960 10.1111/nan.12518 PMC6433556

[407] Cho Y , Cavalli V . HDAC signaling in neuronal development and axon regeneration. Curr Opin Neurobiol. 2014;27:118‐126. 24727244 10.1016/j.conb.2014.03.008 PMC4122610

[408] Park J , Lee K , Kim K , Yi S‐J . The role of histone modifications: from neurodevelopment to neurodiseases. Signal Transduct Target Ther. 2022;7(1):217. 35794091 10.1038/s41392-022-01078-9 PMC9259618

[409] LoPresti P . HDAC6 in diseases of cognition and of neurons. Cells. 2020;10(1):12. 33374719 10.3390/cells10010012 PMC7822434

[410] Min SW , Sohn PD , Cho SH , Swanson RA , Gan L . Sirtuins in neurodegenerative diseases: an update on potential mechanisms. Front Aging Neurosci. 2013;5:53. 24093018 10.3389/fnagi.2013.00053 PMC3782645

[411] Lalla R , Donmez G . The role of sirtuins in Alzheimer's disease. Front Aging Neurosci. 2013;5:16. 23576985 10.3389/fnagi.2013.00016 PMC3620486

[412] Naia L , Carmo C , Campesan S , et al. Mitochondrial SIRT3 confers neuroprotection in Huntington's disease by regulation of oxidative challenges and mitochondrial dynamics. Free Radic Biol Med. 2021;163:163‐179. 33285261 10.1016/j.freeradbiomed.2020.11.031

[413] Luo H , Peng C , Xu X , et al. The protective effects of mogroside V against neuronal damages by attenuating mitochondrial dysfunction via upregulating sirtuin3. Mol Neurobiol. 2022;59(4):2068‐2084. 35040040 10.1007/s12035-021-02689-z

[414] Wang D , Cao L , Pan S , et al. Sirt3‐mediated mitochondrial dysfunction is involved in fluoride‐induced cognitive deficits. Food Chem Toxicol. 2021;158:112665. 34780879 10.1016/j.fct.2021.112665

[415] Shen Y , Wu Q , Shi J , Zhou S . Regulation of SIRT3 on mitochondrial functions and oxidative stress in Parkinson's disease. Biomed Pharmacother. 2020;132:110928. 33128944 10.1016/j.biopha.2020.110928

[416] Zhang X , Ren X , Zhang Q , et al. PGC‐1α/ERRα‐Sirt3 pathway regulates DAergic neuronal death by directly deacetylating SOD2 and ATP synthase β. Antioxid Redox Signal. 2016;24(6):312‐328. 26421366 10.1089/ars.2015.6403 PMC4761832

[417] Pasqualucci L , Dominguez‐Sola D , Chiarenza A , et al. Inactivating mutations of acetyltransferase genes in B‐cell lymphoma. Nature. 2011;471(7337):189‐195. 21390126 10.1038/nature09730 PMC3271441

[418] Mullighan CG , Zhang J , Kasper LH , et al. CREBBP mutations in relapsed acute lymphoblastic leukaemia. Nature. 2011;471(7337):235‐239. 21390130 10.1038/nature09727 PMC3076610

[419] Füllgrabe J , Lynch‐Day MA , Heldring N , et al. The histone H4 lysine 16 acetyltransferase hMOF regulates the outcome of autophagy. Nature. 2013;500(7463):468‐471. 23863932 10.1038/nature12313 PMC4006103

[420] Wang L , Li X , Zhang W , et al. miR24‐2 promotes malignant progression of human Liver cancer stem cells by enhancing tyrosine kinase Src epigenetically. Mol Ther. 2020;28(2):572‐586. 31732298 10.1016/j.ymthe.2019.10.015 PMC7001004

[421] Yang J , Song C , Zhan X . The role of protein acetylation in carcinogenesis and targeted drug discovery. Front Endocrinol (Lausanne). 2022;13:972312. 36171897 10.3389/fendo.2022.972312 PMC9510633

[422] Xu L , Chen Y , Song Q , Xu D , Wang Y , Ma D . PDCD5 interacts with Tip60 and functions as a cooperator in acetyltransferase activity and DNA damage‐induced apoptosis. Neoplasia. 2009;11(4):345‐354. 19308289 10.1593/neo.81524 PMC2657881

[423] Feng X , Zhang H , Meng L , et al. Hypoxia‐induced acetylation of PAK1 enhances autophagy and promotes brain tumorigenesis via phosphorylating ATG5. Autophagy. 2021;17(3):723‐742. 32186433 10.1080/15548627.2020.1731266 PMC8032228

[424] Lv L , Li D , Zhao D , et al. Acetylation targets the M2 isoform of pyruvate kinase for degradation through chaperone‐mediated autophagy and promotes tumor growth. Mol Cell. 2011;42(6):719‐730. 21700219 10.1016/j.molcel.2011.04.025 PMC4879880

[425] Zhao D , Zou S‐W , Liu Y , et al. Lysine‐5 acetylation negatively regulates lactate dehydrogenase A and is decreased in pancreatic cancer. Cancer Cell. 2013;23(4):464‐476. 23523103 10.1016/j.ccr.2013.02.005 PMC3885615

[426] Yang J , Jin X , Yan Y , et al. Inhibiting histone deacetylases suppresses glucose metabolism and hepatocellular carcinoma growth by restoring FBP1 expression. Sci Rep. 2017;7:43864. 28262837 10.1038/srep43864 PMC5338333

[427] Fan J , Shan C , Kang H‐B , et al. Tyr phosphorylation of PDP1 toggles recruitment between ACAT1 and SIRT3 to regulate the pyruvate dehydrogenase complex. Mol Cell. 2014;53(4):534‐548. 24486017 10.1016/j.molcel.2013.12.026 PMC3943932

[428] Shan C , Elf S , Ji Q , et al. Lysine acetylation activates 6‐phosphogluconate dehydrogenase to promote tumor growth. Mol Cell. 2014;55(4):552‐565. 25042803 10.1016/j.molcel.2014.06.020 PMC4142084

[429] Lin H‐P , Cheng Z‐L , He R‐Y , et al. Destabilization of fatty acid synthase by acetylation inhibits de novo lipogenesis and tumor cell growth. Cancer Res. 2016;76(23):6924‐6936. 27758890 10.1158/0008-5472.CAN-16-1597 PMC5135623

[430] Lei MZ , Li XX , Zhang Y , et al. Acetylation promotes BCAT2 degradation to suppress BCAA catabolism and pancreatic cancer growth. Signal Transduct Target Ther. 2020;5(1):70. 32467562 10.1038/s41392-020-0168-0 PMC7256045

[431] Castillo EC , Morales JA , Chapoy‐Villanueva H , et al. Mitochondrial hyperacetylation in the failing hearts of obese patients mediated partly by a reduction in SIRT3: the involvement of the mitochondrial permeability transition pore. Cell Physiol Biochem. 2019;53(3):465‐479. 31464387 10.33594/000000151

[432] Li P , Zhang L , Zhou C , Lin N , Liu A . Sirt 1 activator inhibits the AGE‐induced apoptosis and p53 acetylation in human vascular endothelial cells. J Toxicol Sci. 2015;40(5):615‐624. 26354378 10.2131/jts.40.615

[433] Vadvalkar SS , Matsuzaki S , Eyster CA , et al. Decreased mitochondrial pyruvate transport activity in the diabetic heart: role of mitochondrial pyruvate carrier 2 (MPC2) acetylation. J Biol Chem. 2017;292(11):4423‐4433. 28154187 10.1074/jbc.M116.753509 PMC5377762

[434] Gogna R , Madan E , Khan M , Pati U , Kuppusamy P . p53's choice of myocardial death or survival: oxygen protects infarct myocardium by recruiting p53 on NOS3 promoter through regulation of p53‐Lys(118) acetylation. EMBO Mol Med. 2013;5(11):1662‐1683. 24096875 10.1002/emmm.201202055 PMC3840484

[435] Leng Y , Wu Y , Lei S , et al. Inhibition of HDAC6 activity alleviates myocardial ischemia/reperfusion injury in diabetic rats: potential role of peroxiredoxin 1 acetylation and redox regulation. Oxid Med Cell Longev. 2018;2018:9494052. 30046381 10.1155/2018/9494052 PMC6036837

[436] Ooi JYY , Tuano NK , Rafehi H , et al. HDAC inhibition attenuates cardiac hypertrophy by acetylation and deacetylation of target genes. Epigenetics. 2015;10(5):418‐430. 25941940 10.1080/15592294.2015.1024406 PMC4622459

[437] Samant SA , Pillai VB , Sundaresan NR , Shroff SG , Gupta MP . Histone Deacetylase 3 (HDAC3)‐dependent reversible lysine acetylation of cardiac myosin heavy chain isoforms modulates their enzymatic and motor activity. J Biol Chem. 2015;290(25):15559‐15569. 25911107 10.1074/jbc.M115.653048 PMC4505469

[438] Wu X , Pan B , Liu L , et al. In utero exposure to PM2.5 during gestation caused adult cardiac hypertrophy through histone acetylation modification. J Cell Biochem. 2019;120(3):4375‐4384. 30269375 10.1002/jcb.27723

[439] Kang S‐H , Seok YM , Song M‐j , Lee H‐A , Kurz T , Kim I . Histone deacetylase inhibition attenuates cardiac hypertrophy and fibrosis through acetylation of mineralocorticoid receptor in spontaneously hypertensive rats. Mol Pharmacol. 2015;87(5):782‐791. 25667225 10.1124/mol.114.096974

[440] Dikalova AE , Pandey A , Xiao L , et al. Mitochondrial deacetylase Sirt3 reduces vascular dysfunction and hypertension while Sirt3 depletion in essential hypertension is linked to vascular inflammation and oxidative stress. Circ Res. 2020;126(4):439‐452. 31852393 10.1161/CIRCRESAHA.119.315767 PMC7035170

[441] Meraviglia V , Azzimato V , Colussi C , et al. Acetylation mediates Cx43 reduction caused by electrical stimulation. J Mol Cell Cardiol. 2015;87:54‐64. 26264759 10.1016/j.yjmcc.2015.08.001 PMC4637213

[442] Liu H‐Y , Liu Y‐Y , Yang F , et al. Acetylation of MORC2 by NAT10 regulates cell‐cycle checkpoint control and resistance to DNA‐damaging chemotherapy and radiotherapy in breast cancer. Nucleic Acids Res. 2020;48(7):3638‐3656. 32112098 10.1093/nar/gkaa130 PMC7144926

[443] Zhang Y , Liu Z , Yang X , et al. H3K27 acetylation activated‐COL6A1 promotes osteosarcoma lung metastasis by repressing STAT1 and activating pulmonary cancer‐associated fibroblasts. Theranostics. 2021;11(3):1473‐1492. 33391546 10.7150/thno.51245 PMC7738898

[444] Fan Y , Hou T , Gao Y , et al. Acetylation‐dependent regulation of TPD52 isoform 1 modulates chaperone‐mediated autophagy in prostate cancer. Autophagy. 2021;17(12):4386‐4400. 34034634 10.1080/15548627.2021.1917130 PMC8726735

[445] Kalinski AL , Kar AN , Craver J , et al. Deacetylation of Miro1 by HDAC6 blocks mitochondrial transport and mediates axon growth inhibition. J Cell Biol. 2019;218(6):1871‐1890. 31068376 10.1083/jcb.201702187 PMC6548128

[446] Haberland M , Montgomery RL , Olson EN . The many roles of histone deacetylases in development and physiology: implications for disease and therapy. Nat Rev Genet. 2009;10(1):32‐42. 19065135 10.1038/nrg2485 PMC3215088

[447] Graff J , Tsai LH . Histone acetylation: molecular mnemonics on the chromatin. Nat Rev Neurosci. 2013;14(2):97‐111. 23324667 10.1038/nrn3427

[448] Neganova ME , Klochkov SG , Aleksandrova YR , Aliev G . Histone modifications in epigenetic regulation of cancer: perspectives and achieved progress. Semin Cancer Biol. 2022;83:452‐471. 32814115 10.1016/j.semcancer.2020.07.015

[449] Benedetti R , Conte M , Altucci L . Targeting histone deacetylases in diseases: where are we? Antioxid Redox Signal. 2015;23(1):99‐126. 24382114 10.1089/ars.2013.5776 PMC4492558

[450] Song Y , Shiota M , Tamiya S , Kuroiwa K , Naito S , Tsuneyoshi M . The significance of strong histone deacetylase 1 expression in the progression of prostate cancer. Histopathology. 2011;58(5):773‐780. 21438903 10.1111/j.1365-2559.2011.03797.x

[451] Yu Z , Zeng J , Liu H , Wang T , Yu Z , Chen J . Role of HDAC1 in the progression of gastric cancer and the correlation with lncRNAs. Oncol Lett. 2019;17(3):3296‐3304. 30867763 10.3892/ol.2019.9962 PMC6396103

[452] Suzuki J , Chen YY , Scott GK , et al. Protein acetylation and histone deacetylase expression associated with malignant breast cancer progression. Clin Cancer Res. 2009;15(9):3163‐3171. 19383825 10.1158/1078-0432.CCR-08-2319 PMC3746548

[453] Fritzsche FR , Weichert W , Roske A , et al. Class I histone deacetylases 1, 2 and 3 are highly expressed in renal cell cancer. BMC Cancer. 2008;8:381. 19099586 10.1186/1471-2407-8-381 PMC2631013

[454] Adams H , Fritzsche FR , Dirnhofer S , Kristiansen G , Tzankov A . Class I histone deacetylases 1, 2 and 3 are highly expressed in classical Hodgkin's lymphoma. Expert Opin Ther Targets. 2010;14(6):577‐584. 20415600 10.1517/14728221003796609

[455] Mehnert JM , Kelly WK . Histone deacetylase inhibitors: biology and mechanism of action. Cancer J. 2007;13(1):23‐29. 17464243 10.1097/PPO.0b013e31803c72ba

[456] Bose P , Dai Y , Grant S . Histone deacetylase inhibitor (HDACI) mechanisms of action: emerging insights. Pharmacol Ther. 2014;143(3):323‐336. 24769080 10.1016/j.pharmthera.2014.04.004 PMC4117710

[457] Jones PA , Issa JP , Baylin S . Targeting the cancer epigenome for therapy. Nat Rev Genet. 2016;17(10):630‐641. 27629931 10.1038/nrg.2016.93

[458] Ito K , Charron CE , Adcock IM . Impact of protein acetylation in inflammatory lung diseases. Pharmacol Ther. 2007;116(2):249‐265. 17720252 10.1016/j.pharmthera.2007.06.009

[459] Liu D , Tang H , Li XY , et al. Targeting the HDAC2/HNF‐4A/miR‐101b/AMPK pathway rescues tauopathy and dendritic abnormalities in Alzheimer's disease. Mol Ther. 2017;25(3):752‐764. 28202389 10.1016/j.ymthe.2017.01.018 PMC5363202

[460] Burg T , Rossaert E , Moisse M , Van Damme P , Van Den Bosch L . Histone deacetylase inhibition regulates lipid homeostasis in a mouse model of amyotrophic lateral sclerosis. Int J Mol Sci. 2021;22(20):11224. 34681883 10.3390/ijms222011224 PMC8541517

[461] Dedoni S , Marras L , Olianas MC , Ingianni A , Onali P . Downregulation of TrkB expression and signaling by valproic acid and other histone deacetylase inhibitors. J Pharmacol Exp Ther. 2019;370(3):490‐503. 31308194 10.1124/jpet.119.258129

[462] Esteves AR , Arduíno DM , Silva DF , Viana SD , Pereira FC , Cardoso SM . Mitochondrial metabolism regulates microtubule acetylome and autophagy trough sirtuin‐2: impact for Parkinson's disease. Mol Neurobiol. 2018;55(2):1440‐1462. 28168426 10.1007/s12035-017-0420-y

[463] Zhang Y , Anoopkumar‐Dukie S , Arora D , Davey AK . Review of the anti‐inflammatory effect of SIRT1 and SIRT2 modulators on neurodegenerative diseases. Eur J Pharmacol. 2020;867:172847. 31812544 10.1016/j.ejphar.2019.172847

[464] Guedes‐Dias P , de Proença J , Soares TR , et al. HDAC6 inhibition induces mitochondrial fusion, autophagic flux and reduces diffuse mutant huntingtin in striatal neurons. Biochim Biophys Acta. 2015;1852(11):2484‐2493. 26300485 10.1016/j.bbadis.2015.08.012

[465] Advani A , Huang Q , Thai K , et al. Long‐term administration of the histone deacetylase inhibitor vorinostat attenuates renal injury in experimental diabetes through an endothelial nitric oxide synthase‐dependent mechanism. Am J Pathol. 2011;178(5):2205‐2214. 21514434 10.1016/j.ajpath.2011.01.044 PMC3081208

[466] Zhang L , Qin X , Zhao Y , et al. Inhibition of histone deacetylases preserves myocardial performance and prevents cardiac remodeling through stimulation of endogenous angiomyogenesis. J Pharmacol Exp Ther. 2012;341(1):285‐293. 22271820 10.1124/jpet.111.189910 PMC3310703

[467] Huang Y , Zhang J , Xu D , Peng Y , Jin Y , Zhang L . SIRT6specific inhibitor OSS128167 exacerbates diabetic cardiomyopathy by aggravating inflammation and oxidative stress. Mol Med Rep. 2021;23(5):367. 33760202 10.3892/mmr.2021.12006 PMC7986000

[468] Russell WR , Hoyles L , Flint HJ , Dumas M‐E . Colonic bacterial metabolites and human health. Curr Opin Microbiol. 2013;16(3):246‐254. 23880135 10.1016/j.mib.2013.07.002

[469] den Besten G , van Eunen K , Groen AK , Venema K , Reijngoud D‐J , Bakker BM . The role of short‐chain fatty acids in the interplay between diet, gut microbiota, and host energy metabolism. J Lipid Res. 2013;54(9):2325‐2340. 23821742 10.1194/jlr.R036012 PMC3735932

[470] Wagner GR , Bhatt DP , O Connell TM , et al. A class of reactive acyl‐coA species reveals the non‐enzymatic origins of protein acylation. Cell Metab. 2017;25(4):823‐837. e8. 28380375 10.1016/j.cmet.2017.03.006 PMC5399522

[471] Sinclair WR , Shrimp JH , Zengeya TT , et al. Bioorthogonal pro‐metabolites for profiling short chain fatty acylation. Chem Sci. 2018;9(5):1236‐1241. 29675169 10.1039/c7sc00247e PMC5885804

[472] Chen Y , Sprung R , Tang Y , et al. Lysine propionylation and butyrylation are novel post‐translational modifications in histones. Mol Cell Proteomics. 2007;6(5):812‐819. 17267393 10.1074/mcp.M700021-MCP200 PMC2911958

[473] Dai L , Peng C , Montellier E , et al. Lysine 2‐hydroxyisobutyrylation is a widely distributed active histone mark. Nat Chem Biol. 2014;10(5):365‐370. 24681537 10.1038/nchembio.1497

[474] Zhang Z , Tan M , Xie Z , Dai L , Chen Y , Zhao Y . Identification of lysine succinylation as a new post‐translational modification. Nat Chem Biol. 2011;7(1):58‐63. 21151122 10.1038/nchembio.495 PMC3065206

[475] Zhu Z , Han Z , Halabelian L , et al. Identification of lysine isobutyrylation as a new histone modification mark. Nucleic Acids Res. 2021;49(1):177‐189. 33313896 10.1093/nar/gkaa1176 PMC7797053

[476] Peng C , Lu Z , Xie Z , et al. The first identification of lysine malonylation substrates and its regulatory enzyme. Mol Cell Proteomics. 2011;10(12):M111.012658. 10.1074/mcp.M111.012658 PMC3237090 21908771

[477] Tan M , Peng C , Anderson KA , et al. Lysine glutarylation is a protein posttranslational modification regulated by SIRT5. Cell Metab. 2014;19(4):605‐617. 24703693 10.1016/j.cmet.2014.03.014 PMC4108075

[478] Tan M , Luo H , Lee S , et al. Identification of 67 histone marks and histone lysine crotonylation as a new type of histone modification. Cell. 2011;146(6):1016‐1028. 21925322 10.1016/j.cell.2011.08.008 PMC3176443

[479] Sabari BR , Zhang D , Allis CD , Zhao Y . Metabolic regulation of gene expression through histone acylations. Nat Rev Mol Cell Biol. 2017;18(2):90‐101. 27924077 10.1038/nrm.2016.140 PMC5320945

[480] Zhang D , Tang Z , Huang H , et al. Metabolic regulation of gene expression by histone lactylation. Nature. 2019;574(7779):575‐580. 31645732 10.1038/s41586-019-1678-1 PMC6818755

[481] Xu H , Wu M , Ma X , Huang W , Xu Y . Function and mechanism of novel histone posttranslational modifications in health and disease. Biomed Res Int. 2021;2021:1‐13. 33763479 10.1155/2021/6635225 PMC7952163

[482] Zhao S , Zhang X , Li H . Beyond histone acetylation—writing and erasing histone acylations. Curr Opin Struct Biol. 2018;53:169‐177. 30391813 10.1016/j.sbi.2018.10.001

[483] Galván‐Peña S , Carroll RG , Newman C , et al. Malonylation of GAPDH is an inflammatory signal in macrophages. Nat Commun. 2019;10(1):338. 30659183 10.1038/s41467-018-08187-6 PMC6338787

[484] Li L , Shi L , Yang S , et al. SIRT7 is a histone desuccinylase that functionally links to chromatin compaction and genome stability. Nat Commun. 2016;7(1):12235. 27436229 10.1038/ncomms12235 PMC4961794

[485] Ringel AE , Wolberger C . Structural basis for acyl‐group discrimination by human Gcn5L2. Acta Crystallogr D Struct Biol. 2016;72(7):841‐848. 27377381 10.1107/S2059798316007907 PMC4932917

[486] Leemhuis H , Packman LC , Nightingale KP , Hollfelder F . The human histone acetyltransferase P/CAF is a promiscuous histone propionyltransferase. ChemBioChem. 2008;9(4):499‐503. 18247445 10.1002/cbic.200700556

[487] Ding Q , Zhang Z , Li Y , et al. Propionate induces intestinal oxidative stress via Sod2 propionylation in zebrafish. iScience. 2021;24(6):102515‐102515. 34142031 10.1016/j.isci.2021.102515 PMC8188496

[488] Yan K , Rousseau J , Machol K , et al. Deficient histone H3 propionylation by BRPF1‐KAT6 complexes in neurodevelopmental disorders and cancer. Sci Adv. 2020;6(4):eaax0021. 32010779 10.1126/sciadv.aax0021 PMC6976298

[489] Du J , Zhou Y , Su X . Sirt5 is a NAD‐dependent protein lysine demalonylase and desuccinylase. Science. 2011;334(6057):806‐809. 22076378 10.1126/science.1207861 PMC3217313

[490] Bhattacharya A , Chatterjee S , Bhaduri U , et al. Butyrylation meets adipogenesis‐probed by a p300‐catalyzed acylation‐specific small molecule inhibitor: implication in anti‐obesity therapy. J Med Chem. 2022;65(18):12273‐12291. 36074919 10.1021/acs.jmedchem.2c00943

[491] Kebede AF , Nieborak A , Shahidian LZ , et al. Histone propionylation is a mark of active chromatin. Nat Struct Mol Biol. 2017;24(12):1048‐1056. 29058708 10.1038/nsmb.3490

[492] Zhang X , Cao R , Niu J , et al. Molecular basis for hierarchical histone de‐β‐hydroxybutyrylation by SIRT3. Cell Discov. 2019;5(1):35. 31636949 10.1038/s41421-019-0103-0 PMC6796883

[493] Rardin MJ , He W , Nishida Y , et al. SIRT5 regulates the mitochondrial lysine succinylome and metabolic networks. Cell Metab. 2013;18(6):920‐933. 24315375 10.1016/j.cmet.2013.11.013 PMC4105152

[494] Witkowski A , Thweatt J , Smith S . Mammalian ACSF3 protein is a malonyl‐CoA synthetase that supplies the chain extender units for mitochondrial fatty acid synthesis. J Biol Chem. 2011;286(39):33729‐33736. 21846720 10.1074/jbc.M111.291591 PMC3190830

[495] Wang Y , Guo YR , Liu K , et al. KAT2A coupled with the α‐KGDH complex acts as a histone H3 succinyltransferase. Nature. 2017;552(7684):273‐277. 29211711 10.1038/nature25003 PMC5841452

[496] Huang H , Luo Z , Qi S , et al. Landscape of the regulatory elements for lysine 2‐hydroxyisobutyrylation pathway. Cell Res. 2018;28(1):111‐125. 29192674 10.1038/cr.2017.149 PMC5752845

[497] Park J , Chen Y , Tishkoff DX , et al. SIRT5‐mediated lysine desuccinylation impacts diverse metabolic pathways. Mol Cell. 2013;50(6):919‐930. 23806337 10.1016/j.molcel.2013.06.001 PMC3769971

[498] Lu Y , Li X , Zhao K , et al. Global landscape of 2‐hydroxyisobutyrylation in human pancreatic cancer. Front Oncol. 2022;12:1001807. 36249039 10.3389/fonc.2022.1001807 PMC9563853

[499] Liu K , Li F , Sun Q , et al. p53 β‐hydroxybutyrylation attenuates p53 activity. Cell Death Dis. 2019;10(3):243. 30858356 10.1038/s41419-019-1463-y PMC6411878

[500] Zhou T , Cheng X , He Y , et al. Function and mechanism of histone β‐hydroxybutyrylation in health and disease. Front Immunol. 2022;13:981285. 36172354 10.3389/fimmu.2022.981285 PMC9511043

[501] Huang H , Wang D‐L , Zhao Y . Quantitative crotonylome analysis expands the roles of p300 in the regulation of lysine crotonylation pathway. Proteomics. 2018;18(15):e1700230. 29932303 10.1002/pmic.201700230 PMC6420807

[502] Narita T , Weinert BT , Choudhary C . Functions and mechanisms of non‐histone protein acetylation. Nat Rev Mol Cell Biol. 2019;20(8):508. 30467427 10.1038/s41580-018-0081-3

[503] Fu Y , Yu J , Li F , Ge S . Oncometabolites drive tumorigenesis by enhancing protein acylation: from chromosomal remodelling to nonhistone modification. J Exp Clin Cancer Res. 2022;41(1):144. 35428309 10.1186/s13046-022-02338-w PMC9013066

[504] Xie L , Xiao Y , Meng F , Li Y , Shi Z , Qian K . Functions and mechanisms of lysine glutarylation in eukaryotes. Front Cell Dev Biol. 2021;9:667684. 34249920 10.3389/fcell.2021.667684 PMC8264553

[505] Bao X , Liu Z , Zhang W , et al. Glutarylation of histone H4 lysine 91 regulates chromatin dynamics. Mol Cell. 2019;76(4):660‐675. e9. 31542297 10.1016/j.molcel.2019.08.018

[506] Moreno‐Yruela C , Zhang D , Wei W , et al. Class I histone deacetylases (HDAC1–3) are histone lysine delactylases. Sci Adv. 2022;8(3):eabi6696. 35044827 10.1126/sciadv.abi6696 PMC8769552

[507] Yang M , Huang H , Ge F . Lysine propionylation is a widespread post‐translational modification involved in regulation of photosynthesis and metabolism in cyanobacteria. Int J Mol Sci. 2019;20(19):4792. 31561603 10.3390/ijms20194792 PMC6801645

[508] Okanishi H , Kim K , Masui R , Kuramitsu S . Lysine propionylation is a prevalent post‐translational modification in thermus thermophilus. Mol Cell Proteomics. 2014;13(9):2382‐2398. 24938286 10.1074/mcp.M113.035659 PMC4159656

[509] Tang H , Zhan Z , Zhang Y , Huang X . Propionylation of lysine, a new mechanism of short‐chain fatty acids affecting bacterial virulence. Am J Transl Res. 2022;14(1943‐8141 (Print)):5773‐5784. 36105019 PMC9452321

[510] Trefely S , Huber K , Liu J , et al. Quantitative subcellular acyl‐CoA analysis reveals distinct nuclear metabolism and isoleucine‐dependent histone propionylation. Mol Cell. 2022;82(1097‐4164 (Electronic)):447‐462. e6. 34856123 10.1016/j.molcel.2021.11.006 PMC8950487

[511] Lin H , Su X , He B . Protein lysine acylation and cysteine succination by intermediates of energy metabolism. ACS Chem Biol. 2012;7(6):947‐960. 22571489 10.1021/cb3001793 PMC3376250

[512] Huang H , Zhang D , Weng Y , et al. The regulatory enzymes and protein substrates for the lysine β‐hydroxybutyrylation pathway. Sci Adv. 2021;7(9):eabe2771. 33627428 10.1126/sciadv.abe2771 PMC7904266

[513] Goudarzi A , Zhang D , Huang H , et al. Dynamic competing histone H4 K5K8 acetylation and butyrylation are hallmarks of highly active gene promoters. Mol Cell. 2016;62(2):169‐180. 27105113 10.1016/j.molcel.2016.03.014 PMC4850424

[514] Sabari BR , Tang Z , Huang H , et al. Intracellular crotonyl‐coA stimulates transcription through p300‐catalyzed histone crotonylation. Mol Cell. 2015;58(2):203‐215. 25818647 10.1016/j.molcel.2015.02.029 PMC4501262

[515] Wei W , Liu X , Chen J , et al. Class I histone deacetylases are major histone decrotonylases: evidence for critical and broad function of histone crotonylation in transcription. Cell Res. 2017;27(7):898‐915. 28497810 10.1038/cr.2017.68 PMC5518989

[516] Jeong J , Bertsch J , Hess V , et al. Energy conservation model based on genomic and experimental analyses of a carbon monoxide‐utilizing, butyrate‐forming acetogen, eubacterium limosum KIST612. Appl Environ Microbiol. 2015;81(14):4782‐4790. 25956767 10.1128/AEM.00675-15 PMC4551209

[517] Thesing CS , Bot M , Milaneschi Y , Giltay EJ , Penninx BWJH . Bidirectional longitudinal associations of omega‐3 polyunsaturated fatty acid plasma levels with depressive disorders. J Psychiatr Res. 2020;124:1‐8. 32087424 10.1016/j.jpsychires.2020.02.011

[518] Wang S , Mu G , Qiu B , et al. The function and related diseases of protein crotonylation. Int J Biol Sci. 2021;17(13):3441‐3455. 34512158 10.7150/ijbs.58872 PMC8416722

[519] Liu X , Zhang Y , Li W , Zhou X . Lactylation, an emerging hallmark of metabolic reprogramming: current progress and open challenges. Front Cell Dev Biol. 2022;10:972020. 36092712 10.3389/fcell.2022.972020 PMC9462419

[520] Ferguson BS , Rogatzki MJ , Goodwin ML , Kane DA , Rightmire Z , Gladden LA‐O . Lactate metabolism: historical context, prior misinterpretations, and current understanding. Eur J Appl Physiol. 2018;118(1439‐6327 (Electronic)):691‐728. 29322250 10.1007/s00421-017-3795-6

[521] Brooks GA . Lactate as a fulcrum of metabolism. Redox Biol. 2020;35(2213‐2317 (Electronic)):101454. 32113910 10.1016/j.redox.2020.101454 PMC7284908

[522] Bhagat TD , Von Ahrens D , Dawlaty M , et al. Lactate‐mediated epigenetic reprogramming regulates formation of human pancreatic cancer‐associated fibroblasts. Elife. 2019;8:e50663. 31663852 10.7554/eLife.50663 PMC6874475

[523] Trefely S , Lovell CD , Snyder NW , Wellen KE . Compartmentalised acyl‐CoA metabolism and roles in chromatin regulation. Mol Metab. 2020;38:100941. 32199817 10.1016/j.molmet.2020.01.005 PMC7300382

[524] Colak G , Pougovkina O , Dai L , et al. Proteomic and biochemical studies of lysine malonylation suggest Its malonic aciduria‐associated regulatory role in mitochondrial function and fatty acid oxidation[S]. Mol Cell Proteomics. 2015;14(11):3056‐3071. 26320211 10.1074/mcp.M115.048850 PMC4638046

[525] Nishida Y , Rardin MJ , Carrico C , et al. SIRT5 Regulates both Cytosolic and Mitochondrial Protein Malonylation with Glycolysis as a Major Target. Mol Cell. 2015;59(2):321‐332. 26073543 10.1016/j.molcel.2015.05.022 PMC4571487

[526] Nie L , Shuai L , Zhu M , et al. The landscape of histone modifications in a high‐fat diet‐induced obese (DIO) mouse model. Mol Cell Proteomics. 2017;16(7):1324‐1334. 28450421 10.1074/mcp.M117.067553 PMC5500764

[527] Figlia G , Willnow P , Teleman AA . Metabolites regulate cell signaling and growth via covalent modification of proteins. Dev Cell. 2020;54(2):156‐170. 32693055 10.1016/j.devcel.2020.06.036

[528] Sadhukhan S , Liu X , Ryu D , et al. Metabolomics‐assisted proteomics identifies succinylation and SIRT5 as important regulators of cardiac function. Proc Natl Acad Sci U S A. 2016;113(16):4320‐4325. 27051063 10.1073/pnas.1519858113 PMC4843474

[529] Frank RAW , Price AJ , Northrop FD , Perham RN , Luisi BF . Crystal structure of the E1 component of the escherichia coli 2‐oxoglutarate dehydrogenase multienzyme complex. J Mol Biol. 2007;368(3):639‐651. 17367808 10.1016/j.jmb.2007.01.080 PMC7611002

[530] Liu J , Shangguan Y , Tang D , Dai Y . Histone succinylation and its function on the nucleosome. J Cell Mol Med. 2021;25(15):7101‐7109. 34160884 10.1111/jcmm.16676 PMC8335665

[531] Smestad J , Erber L , Chen Y , Maher LJ, III . Chromatin succinylation correlates with active gene expression and is perturbed by defective TCA cycle metabolism. iScience. 2018;2:63‐75. 29888767 10.1016/j.isci.2018.03.012 PMC5993049

[532] Tucci S , Alatibi KI , Wehbe Z . Altered metabolic flexibility in inherited metabolic diseases of mitochondrial fatty acid metabolism. Int J Mol Sci. 2021;22(7):3799. 33917608 10.3390/ijms22073799 PMC8038842

[533] Zhou L , Wang F , Sun R , et al. SIRT 5 promotes IDH 2 desuccinylation and G6PD deglutarylation to enhance cellular antioxidant defense. EMBO reports. 2016;17(6):811‐822. 27113762 10.15252/embr.201541643 PMC5278614

[534] Schmiesing J , Storch S , Dörfler A‐C , et al. Disease‐linked glutarylation impairs function and interactions of mitochondrial proteins and contributes to mitochondrial heterogeneity. Cell Reports. 2018;24(11):2946‐2956. 30208319 10.1016/j.celrep.2018.08.014

[535] Cheng Y‐M , Hu X‐N , Peng Z , et al. Lysine glutarylation in human sperm is associated with progressive motility. Hum Reprod. 2019;34(7):1186‐1194. 31194865 10.1093/humrep/dez068

[536] Xie Z , Zhang D , Chung D , et al. Metabolic regulation of gene expression by histone lysine β‐hydroxybutyrylation. Mol Cell. 2016;62(2):194‐206. 27105115 10.1016/j.molcel.2016.03.036 PMC5540445

[537] Huang S , Tang D , Dai Y . Metabolic functions of lysine 2‐hydroxyisobutyrylation. Cureus. 2020;12(2168‐8184 (Print)):e9651. 32923251 10.7759/cureus.9651 PMC7482994

[538] Cheng YM , Peng Z , Chen HY , et al. Posttranslational lysine 2‐hydroxyisobutyrylation of human sperm tail proteins affects motility. Hum Reprod. 2020;35(1460‐2350 (Electronic)):494‐503. 32142584 10.1093/humrep/dez296

[539] Cahill GF, Jr. Fuel metabolism in starvation. Annu Rev Nutr. 2006;26(0199‐9885 (Print)):1‐22. 16848698 10.1146/annurev.nutr.26.061505.111258

[540] Li Z , Zhang Y , Han M , et al. Lysine β‐hydroxybutyrylation improves stability of COVID‐19 antibody. Biomacromolecules. 2022;23(1):454‐463. 34879647 10.1021/acs.biomac.1c01435

[541] Nitsch S , Zorro Shahidian L , Schneider RA‐O . Histone acylations and chromatin dynamics: concepts, challenges, and links to metabolism. Nat Rev Mol Cell Biol. 2021;22(1469‐3178 (Electronic)):e52774. 10.15252/embr.202152774 PMC8406397 34159701

[542] Koh A , De Vadder F , Kovatcheva‐Datchary P , et al. From dietary fiber to host physiology: short‐chain fatty acids as key bacterial metabolites. Cell. 2016;165(6):1332‐1345. 27259147 10.1016/j.cell.2016.05.041

[543] Chen X‐F , Chen X , Tang X . Short‐chain fatty acid, acylation and cardiovascular diseases. Clin Sci 2020;134(6):657‐676. 10.1042/CS20200128 32219347

[544] Xu Y , Shi Z , Bao L . An expanding repertoire of protein acylations. Mol Cell Proteomics. 2022;21(3):100193. 34999219 10.1016/j.mcpro.2022.100193 PMC8933697

[545] Lin H Fau ‐ Begley T , Begley T . Protein posttranslational modifications: chemistry, biology, and applications. Mol Biosyst. 2011;7(1742‐2051 (Electronic)):14‐15. 21107473 10.1039/c0mb90037k

[546] Borsche M , Pereira SL , Klein C , Grünewald A . Mitochondria and parkinson's disease: clinical, molecular, and translational aspects. J Parkinsons Dis. 2021;11(1):45‐60. 33074190 10.3233/JPD-201981 PMC7990451

[547] Yang Z , He M , Austin J , Pfleger J , Abdellatif M . Histone H3K9 butyrylation is regulated by dietary fat and stress via an Acyl‐CoA dehydrogenase short chain‐dependent mechanism. Mol Metab. 2019;(2212‐8778 (Electronic)) 10.1016/j.molmet.2021.101249 PMC8188563 33989779

[548] Bentley RA , Ross CN , O'Brien MJ . Obesity, metabolism, and aging: A multiscalar approach. Prog Mol Biol Transl Sci. 2018;155:25‐42. 29653680 10.1016/bs.pmbts.2017.11.016

[549] Salas‐Huetos A , Maghsoumi‐Norouzabad L , James ER , et al. Male adiposity, sperm parameters and reproductive hormones: An updated systematic review and collaborative meta‐analysis. Obes Rev. 2021;22(1):e13082. 32705766 10.1111/obr.13082

[550] Wang F , Chen H , Chen Y , et al. Diet‐induced obesity is associated with altered expression of sperm motility‐related genes and testicular post‐translational modifications in a mouse model. Theriogenology. 2020;158:233‐238. 32980686 10.1016/j.theriogenology.2020.09.023

[551] Zhu S , Batushansky A , Jopkiewicz A , et al. Sirt5 deficiency causes posttranslational protein malonylation and dysregulated cellular metabolism in chondrocytes under obesity conditions. Cartilage. 2021;13(2_suppl):1185S‐1199S. 33567897 10.1177/1947603521993209 PMC8804736

[552] Mao T , Wei Q , Zhao F , Zhang C . Short‐term fasting reshapes fat tissue. Endocr J. 2021;68(4):387‐398. 33441502 10.1507/endocrj.EJ20-0405

[553] Nie L , Shuai L , Zhu M , et al. The landscape of histone modifications in a high‐fat diet‐induced obese (DIO) Mouse Model. Mol Cell Proteomics. 2017;16(1535‐9484 (Electronic)):1324‐1334. 28450421 10.1074/mcp.M117.067553 PMC5500764

[554] Nishitani S , Fukuhara A , Shin J , Okuno Y , Otsuki M , Shimomura I . Metabolomic and microarray analyses of adipose tissue of dapagliflozin‐treated mice, and effects of 3‐hydroxybutyrate on induction of adiponectin in adipocytes. Sci Rep. 2018;8(2045‐2322 (Electronic)):8805. 29891844 10.1038/s41598-018-27181-y PMC5995811

[555] Ruiz‐Andres O , Sanchez‐Nino MD , Cannata‐Ortiz P , et al. Histone lysine crotonylation during acute kidney injury in mice. Dis Model Mech. 2016;9(6):633‐645. 27125278 10.1242/dmm.024455 PMC4920150

[556] Dang L , Cao X , Zhang T , et al. Nuclear condensation of CDYL links histone crotonylation and cystogenesis in autosomal dominant polycystic kidney disease. J Am Soc Nephrol. 2022;33(9):1708‐1725. 35918147 10.1681/ASN.2021111425 PMC9529191

[557] Zhou T , Xu H , Cheng X , et al. Sodium butyrate attenuates diabetic kidney disease partially via histone butyrylation modification. Mediators Inflamm. 2022;2022:7643322. 35909658 10.1155/2022/7643322 PMC9329006

[558] Dong J , Li Y , Zheng F , et al. Co‐occurrence of protein crotonylation and 2‐hydroxyisobutyrylation in the proteome of End‐Stage Renal Disease. ACS Omega. 2021;6(24):15782‐15793. 34179622 10.1021/acsomega.1c01161 PMC8223210

[559] Luo W , Yu Y , Wang H , et al. Up‐regulation of MMP‐2 by histone H3K9 β‐hydroxybutyrylation to antagonize glomerulosclerosis in diabetic rat. Acta Diabetol. 2020;57(12):1501‐1509. 32772200 10.1007/s00592-020-01552-2

[560] Xia L , Oyang L , Lin J , et al. The cancer metabolic reprogramming and immune response. Mol Cancer. 2021;20(1):28. 33546704 10.1186/s12943-021-01316-8 PMC7863491

[561] Gillies RJ , Robey I , Gatenby RA . Causes and consequences of increased glucose metabolism of cancers. J Nucl Med. 2008;49(Suppl 2):24S‐42S. 18523064 10.2967/jnumed.107.047258

[562] Yu J , Chai P , Xie M , et al. Histone lactylation drives oncogenesis by facilitating m6A reader protein YTHDF2 expression in ocular melanoma. Genome Biol. 2021;22(1):85. 33726814 10.1186/s13059-021-02308-z PMC7962360

[563] Yang D , Yin J , Shan L , Yi X , Zhang W , Ding Y . Identification of lysine‐lactylated substrates in gastric cancer cells. iScience. 2022;25(7):104630. 35800753 10.1016/j.isci.2022.104630 PMC9253728

[564] Tong Y , Guo D , Lin S‐H , et al. SUCLA2‐coupled regulation of GLS succinylation and activity counteracts oxidative stress in tumor cells. Mol Cell. 2021;81(11):2303‐2316. 33991485 10.1016/j.molcel.2021.04.002

[565] Verma S , Crawford D , Khateb A , et al. NRF2 mediates melanoma addiction to GCDH by modulating apoptotic signalling. Nat Cell Biol. 2022;24(9):1422‐1432. 36050469 10.1038/s41556-022-00985-x PMC9977532

[566] Wan J , Liu H , Ming L . Lysine crotonylation is involved in hepatocellular carcinoma progression. Biomed Pharmacother. 2019;111:976‐982. 30841477 10.1016/j.biopha.2018.12.148

[567] Han X , Xiang X , Yang H , et al. p300‐Catalyzed lysine crotonylation promotes the proliferation, invasion, and migration of heLa cells via heterogeneous nuclear ribonucleoprotein A1. Anal Cell Pathol (Amst). 2020;2020:1‐6. 10.1155/2020/5632342 PMC7787849 33457194

[568] Xu X , Zhu X , Liu F , Lu W , Wang Y , Yu J . The effects of histone crotonylation and bromodomain protein 4 on prostate cancer cell lines. Transl Androl Urol. 2021;10(2):900‐914. 33718091 10.21037/tau-21-53 PMC7947446

[569] Liu B , Lin Y , Darwanto A , Song X , Xu G , Zhang K . Identification and characterization of propionylation at Histone H3 lysine 23 in mammalian cells. J Biol Chem. 2009;284(47):32288‐32295. 19801601 10.1074/jbc.M109.045856 PMC2781642

[570] Høgh RI , Møller SH , Jepsen SD , et al. Metabolism of short‐chain fatty acid propionate induces surface expression of NKG2D ligands on cancer cells. FASEB J. 2020;34(11):15531‐15546. 32996653 10.1096/fj.202000162R

[571] Yang G , Yuan Y , Yuan H , et al. Histone acetyltransferase 1 is a succinyltransferase for histones and non‐histones and promotes tumorigenesis. EMBO reports. 2021;22(2):e50967. 33372411 10.15252/embr.202050967 PMC7857430

[572] Huang Q , Wu D , Zhao J , et al. TFAM loss induces nuclear actin assembly upon mDia2 malonylation to promote liver cancer metastasis. The EMBO Journal. 2022;41(11):e110324. 35451091 10.15252/embj.2021110324 PMC9156967

[573] Wang H , Lu J , Gao WC , et al. Donepezil down‐regulates propionylation, 2‐hydroxyisobutyrylation, butyrylation, succinylation, and crotonylation in the brain of bilateral common carotid artery occlusion‐induced vascular dementia rats. Clin Exp Pharmacol Physiol. 2020;47(10):1731‐1739. 32424975 10.1111/1440-1681.13352

[574] Wang M , Chang Q , Yang H , et al. Elevated lysine crotonylation and succinylation in the brains of BTBR mice. Int J Dev Neurosci. 2019;76:61‐64. 31255717 10.1016/j.ijdevneu.2019.06.011

[575] Hagihara H , Shoji H , Otabi H , et al. Protein lactylation induced by neural excitation. Cell Rep. 2021;37(2):109820. 34644564 10.1016/j.celrep.2021.109820

[576] Liu Y , Li M , Fan M , et al. Chromodomain Y‐like protein‐mediated histone crotonylation regulates stress‐induced depressive behaviors. Biol Psychiatry. 2019;85(8):635‐649. 30665597 10.1016/j.biopsych.2018.11.025

[577] Smith BJ , Brandão‐Teles C , Zuccoli GS , et al. Protein succinylation and malonylation as potential biomarkers in schizophrenia. J Pers Med. 2022;12(9):1408. 36143193 10.3390/jpm12091408 PMC9500613

[578] McNally MA , Hartman AL . Ketone bodies in epilepsy. J Neurochem. 2012;121(1):28‐35. 22268909 10.1111/j.1471-4159.2012.07670.x PMC3969728

[579] Lim S , Chesser AS , Grima JC , et al. D‐β‐hydroxybutyrate is protective in mouse models of Huntington's disease. PloS One. 2011;6(9):e24620. 21931779 10.1371/journal.pone.0024620 PMC3171454

[580] Chen L , Miao Z , Xu X . β‐hydroxybutyrate alleviates depressive behaviors in mice possibly by increasing the histone3‐lysine9‐β‐hydroxybutyrylation. Biochem Biophys Res Commun. 2017;490(2):117‐122. 28583851 10.1016/j.bbrc.2017.05.184

[581] Yang Y , Tapias V , Acosta D , et al. Altered succinylation of mitochondrial proteins, APP and tau in Alzheimer's disease. Nat Commun. 2022;13(1):159. 35013160 10.1038/s41467-021-27572-2 PMC8748865

[582] Pan RY , He L , Zhang J , et al. Positive feedback regulation of microglial glucose metabolism by histone H4 lysine 12 lactylation in Alzheimer's disease. Cell Metab. 2022;34(4):634‐648.e6. 35303422 10.1016/j.cmet.2022.02.013

[583] Dai SK , Liu PP , Li X , Jiao LF , Teng ZQ , Liu CM . Dynamic profiling and functional interpretation of histone lysine crotonylation and lactylation during neural development. Development. 2022;149(14):dev200049. 35735108 10.1242/dev.200049

[584] Kimura I , Ichimura A , Ohue‐Kitano R , Igarashi M . Free fatty acid receptors in health and disease. Physiol Rev. 2020;100(1):171‐210. 31487233 10.1152/physrev.00041.2018

[585] Timms RT , Zhang Z , Rhee DY , Harper JW , Koren I , Elledge SJ . A glycine‐specific N‐degron pathway mediates the quality control of protein N‐myristoylation. Science. 2019;365(6448):eaaw4912. 31273098 10.1126/science.aaw4912 PMC7090375

[586] Jiang H , Zhang X , Chen X , Aramsangtienchai P , Tong Z , Lin H . Protein lipidation: occurrence, mechanisms, biological functions, and enabling technologies. Chem Rev. 2018;118(3):919‐988. 29292991 10.1021/acs.chemrev.6b00750 PMC5985209

[587] Jin J , Zhi X , Wang X , Meng D . Protein palmitoylation and its pathophysiological relevance. J Cell Physiol. 2021;236(5):3220‐3233. 33094504 10.1002/jcp.30122

[588] Mousnier A , Bell AS , Swieboda DP , et al. Fragment‐derived inhibitors of human N‐myristoyltransferase block capsid assembly and replication of the common cold virus. Nat Chem. 2018;10(6):599‐606. 29760414 10.1038/s41557-018-0039-2 PMC6015761

[589] Wang B , Dai T , Sun W , et al. Protein N‐myristoylation: functions and mechanisms in control of innate immunity. Cell Mol Immunol. 2021;18(4):878‐888. 33731917 10.1038/s41423-021-00663-2 PMC7966921

[590] Finlay DK . N‐myristoylation of AMPK controls T cell inflammatory function. Nat Immunol. 2019;20(3):252‐254. 30718915 10.1038/s41590-019-0322-4

[591] Bagchi RA , Robinson EL , Hu T , et al. Reversible lysine fatty acylation of an anchoring protein mediates adipocyte adrenergic signaling. Proc Natl Acad Sci U S A. 2022;119(7):e2119678119. 35149557 10.1073/pnas.2119678119 PMC8851525

[592] Zhang Q , Zhou W , Yu S , et al. Metabolic reprogramming of ovarian cancer involves ACSL1‐mediated metastasis stimulation through upregulated protein myristoylation. Oncogene. 2021;40(1):97‐111. 33082557 10.1038/s41388-020-01516-4

[593] Cho E , Park M . Palmitoylation in Alzheimer's disease and other neurodegenerative diseases. Pharmacol Res. 2016;111:133‐151. 27293050 10.1016/j.phrs.2016.06.008

[594] Hornemann T . Palmitoylation and depalmitoylation defects. J Inherit Metab Dis. 2015;38(1):179‐186. 25091425 10.1007/s10545-014-9753-0

[595] Zhang Z , Li X , Yang F , et al. DHHC9‐mediated GLUT1 S‐palmitoylation promotes glioblastoma glycolysis and tumorigenesis. Nat Commun. 2021;12(1):5872. 34620861 10.1038/s41467-021-26180-4 PMC8497546

[596] Yuan W , Lu L , Rao M , et al. GFAP hyperpalmitoylation exacerbates astrogliosis and neurodegenerative pathology in PPT1‐deficient mice. Proc Natl Acad Sci U S A. 2021;118(13):e2022261118. 33753498 10.1073/pnas.2022261118 PMC8020761

[597] Uzbekova S , Teixeira‐Gomes AP , Marestaing A , et al. Protein palmitoylation in bovine ovarian follicle. Int J Mol Sci. 2021;22(21):11757. 34769186 10.3390/ijms222111757 PMC8583988

[598] Shen ZC , Xia ZX , Liu JM , et al. APT1‐mediated depalmitoylation regulates hippocampal synaptic plasticity. J Neurosci. 2022;42(13):2662‐2677. 35165175 10.1523/JNEUROSCI.1741-21.2022 PMC8973429

[599] Chen S , Han C , Miao X , et al. Targeting MC1R depalmitoylation to prevent melanomagenesis in redheads. Nat Commun. 2019;10(1):877. 30787281 10.1038/s41467-019-08691-3 PMC6382811

[600] Dore K , Carrico Z , Alfonso S , et al. PSD‐95 protects synapses from β‐amyloid. Cell Rep. 2021;35(9):109194. 34077732 10.1016/j.celrep.2021.109194 PMC8237704

[601] Calero G , Gupta P , Nonato MC , et al. The crystal structure of palmitoyl protein thioesterase‐2 (PPT2) reveals the basis for divergent substrate specificities of the two lysosomal thioesterases, PPT1 and PPT2. J Biol Chem. 2003;278(39):37957‐37964. 12855696 10.1074/jbc.M301225200

[602] Sadhukhan T , Bagh MB , Appu AP , et al. In a mouse model of INCL reduced S‐palmitoylation of cytosolic thioesterase APT1 contributes to microglia proliferation and neuroinflammation. J Inherit Metab Dis. 2021;44(4):1051‐1069. 33739454 10.1002/jimd.12379

[603] Tian L , McClafferty H , Knaus HG , Ruth P , Shipston MJ . Distinct acyl protein transferases and thioesterases control surface expression of calcium‐activated potassium channels. J Biol Chem. 2012;287(18):14718‐14725. 22399288 10.1074/jbc.M111.335547 PMC3340283

[604] Remsberg JR , Suciu RM , Zambetti NA , et al. ABHD17 regulation of plasma membrane palmitoylation and N‐Ras‐dependent cancer growth. Nat Chem Biol. 2021;17(8):856‐864. 33927411 10.1038/s41589-021-00785-8 PMC8900659

[605] Tang J , Peng W , Feng Y , et al. Cancer cells escape p53's tumor suppression through ablation of ZDHHC1‐mediated p53 palmitoylation. Oncogene. 2021;40(35):5416‐5426. 34282274 10.1038/s41388-021-01949-5 PMC8413129

[606] Zhou B , Yang W , Li W , et al. Zdhhc2 is essential for plasmacytoid dendritic cells mediated inflammatory response in psoriasis. Front Immunol. 2020;11:607442. 33488612 10.3389/fimmu.2020.607442 PMC7819861

[607] Zhao R , Zhang H , Zhang Y , Li D , Huang C , Li F . In vivo screen identifies Zdhhc2 as a critical regulator of germinal center B cell differentiation. Front Immunol. 2020;11:1025. 32587588 10.3389/fimmu.2020.01025 PMC7297983

[608] Zhang H , Li X , Ma C , et al. Fine‐mapping of ZDHHC2 identifies risk variants for schizophrenia in the Han Chinese population. Mol Genet Genomic Med. 2020;8(7):e1190. 32180374 10.1002/mgg3.1190 PMC7336764

[609] Xia L , Ou J , Li K , et al. Genome‐wide association analysis of autism identified multiple loci that have been reported as strong signals for neuropsychiatric disorders. Autism Res. 2020;13(3):382‐396. 31647196 10.1002/aur.2229

[610] Smeland OB , Shadrin A , Bahrami S , et al. Genome‐wide association analysis of Parkinson's disease and schizophrenia reveals shared genetic architecture and identifies novel risk loci. Biol Psychiatry. 2021;89(3):227‐235. 32201043 10.1016/j.biopsych.2020.01.026 PMC7416467

[611] Salaun C , Ritchie L , Greaves J , Bushell TJ , Chamberlain LH . The C‐terminal domain of zDHHC2 contains distinct sorting signals that regulate intracellular localisation in neurons and neuroendocrine cells. Mol Cell Neurosci. 2017;85:235‐246. 28768144 10.1016/j.mcn.2017.07.007 PMC5711357

[612] Li D , Liu Y , Lu Y , Gao S , Zhang L . Palmitoylation of SARS‐CoV‐2 S protein is critical for S‐mediated syncytia formation and virus entry. J Med Virol. 2022;94(1):342‐348. 34528721 10.1002/jmv.27339 PMC8661603

[613] Yao H , Lan J , Li C , et al. Inhibiting PD‐L1 palmitoylation enhances T‐cell immune responses against tumours. Nat Biomed Eng. 2019;3(4):306‐317. 30952982 10.1038/s41551-019-0375-6

[614] Xie F , Su P , Pan T , et al. Engineering extracellular vesicles enriched with palmitoylated ACE2 as COVID‐19 therapy. Adv Mater. 2021;33(49):e2103471. 34665481 10.1002/adma.202103471 PMC8646473

[615] Sharma C , Wang HX , Li Q , et al. Protein acyltransferase DHHC3 regulates breast tumor growth, oxidative stress, and senescence. Cancer Res. 2017;77(24):6880‐6890. 29055014 10.1158/0008-5472.CAN-17-1536 PMC5819883

[616] Spinelli M , Fusco S , Mainardi M , et al. Brain insulin resistance impairs hippocampal synaptic plasticity and memory by increasing GluA1 palmitoylation through FoxO3a. Nat Commun. 2017;8(1):2009. 29222408 10.1038/s41467-017-02221-9 PMC5722929

[617] Wang S , Mott KR , Cilluffo M , et al. The absence of DHHC3 affects primary and latent herpes simplex virus 1 infection. J Virol. 2018;92(4):e01599‐17. 29187538 10.1128/JVI.01599-17 PMC5790959

[618] Lee JW , Hur J , Kwon YW , et al. KAI1(CD82) is a key molecule to control angiogenesis and switch angiogenic milieu to quiescent state. J Hematol Oncol. 2021;14(1):148. 34530889 10.1186/s13045-021-01147-6 PMC8444549

[619] Zhao C , Yu H , Fan X , et al. GSK3β palmitoylation mediated by ZDHHC4 promotes tumorigenicity of glioblastoma stem cells in temozolomide‐resistant glioblastoma through the EZH2‐STAT3 axis. Oncogenesis. 2022;11(1):28. 35606353 10.1038/s41389-022-00402-w PMC9126914

[620] Ebersole B , Petko J , Woll M , et al. Effect of C‐terminal S‐palmitoylation on D2 dopamine receptor trafficking and stability. PloS One. 2015;10(11):e0140661. 26535572 10.1371/journal.pone.0140661 PMC4633242

[621] Zeng XT , Yu XX , Cheng W . The interactions of ZDHHC5/GOLGA7 with SARS‐CoV‐2 spike (S) protein and their effects on S protein's subcellular localization, palmitoylation and pseudovirus entry. Virol J. 2021;18(1):257. 34961524 10.1186/s12985-021-01722-w PMC8711289

[622] Lv K , Ren JG , Han X , Gui J , Gong C , Tong W . Depalmitoylation rewires FLT3‐ITD signaling and exacerbates leukemia progression. Blood. 2021;138(22):2244‐2255. 34111291 10.1182/blood.2021011582 PMC8832469

[623] Kim YC , Lee SE , Kim SK , et al. Toll‐like receptor mediated inflammation requires FASN‐dependent MYD88 palmitoylation. Nat Chem Biol. 2019;15(9):907‐916. 31427815 10.1038/s41589-019-0344-0

[624] Qiu N , Abegg D , Guidi M , Gilmore K , Seeberger PH , Adibekian A . Artemisinin inhibits NRas palmitoylation by targeting the protein acyltransferase ZDHHC6. Cell Chem Biol. 2022;29(3):530‐537. e7. 34358442 10.1016/j.chembiol.2021.07.012

[625] Kerkenberg N , Wachsmuth L , Zhang M , et al. Brain microstructural changes in mice persist in adulthood and are modulated by the palmitoyl acyltransferase ZDHHC7. Eur J Neurosci. 2021;54(6):5951‐5967. 34355442 10.1111/ejn.15415

[626] Kerkenberg N , Hohoff C , Zhang M , et al. Acute stress reveals different impacts in male and female Zdhhc7‐deficient mice. Brain Struct Funct. 2021;226(5):1613‐1626. 33880616 10.1007/s00429-021-02275-y PMC8096773

[627] Yang S , Jia L , Xiang J , et al. KLF10 promotes nonalcoholic steatohepatitis progression through transcriptional activation of zDHHC7. EMBO Rep. 2022;23(6):e54229. 35492028 10.15252/embr.202154229 PMC9171407

[628] Yang Q , Zheng F , Hu Y , et al. ZDHHC8 critically regulates seizure susceptibility in epilepsy. Cell Death Dis. 2018;9(8):795. 30038264 10.1038/s41419-018-0842-0 PMC6056564

[629] Strassburger K , Kang E , Teleman AA . Drosophila ZDHHC8 palmitoylates scribble and Ras64B and controls growth and viability. PloS One. 2019;14(2):e0198149. 30735487 10.1371/journal.pone.0198149 PMC6368284

[630] Collura KM , Niu J , Sanders SS , Montersino A , Holland SM , Thomas GM . The palmitoyl acyltransferases ZDHHC5 and ZDHHC8 are uniquely present in DRG axons and control retrograde signaling via the Gp130/JAK/STAT3 pathway. J Biol Chem. 2020;295(46):15427‐15437. 32958558 10.1074/jbc.RA120.013815 PMC7667964

[631] Shimell JJ , Shah BS , Cain SM , et al. The X‐Linked intellectual disability gene Zdhhc9 is essential for dendrite outgrowth and inhibitory synapse formation. Cell Rep. 2019;29(8):2422‐2437. e8. 31747610 10.1016/j.celrep.2019.10.065

[632] Kouskou M , Thomson DM , Brett RR , et al. Disruption of the Zdhhc9 intellectual disability gene leads to behavioural abnormalities in a mouse model. Exp Neurol. 2018;308:35‐46. 29944857 10.1016/j.expneurol.2018.06.014 PMC6104741

[633] Liu E , Sun J , Yang J , et al. ZDHHC11 positively regulates NF‐κB activation by enhancing TRAF6 oligomerization. Front Cell Dev Biol. 2021;9:710967. 34490261 10.3389/fcell.2021.710967 PMC8417235

[634] Liu Y , Zhou Q , Zhong L , et al. ZDHHC11 modulates innate immune response to DNA virus by mediating MITA‐IRF3 association. Cell Mol Immunol. 2018;15(10):907‐916. 29429998 10.1038/cmi.2017.146 PMC6207569

[635] Dzikiewicz‐Krawczyk A , Kok K , Slezak‐Prochazka I , et al. ZDHHC11 and ZDHHC11B are critical novel components of the oncogenic MYC‐miR‐150‐MYB network in Burkitt lymphoma. Leukemia. 2017;31(6):1470‐1473. 28331227 10.1038/leu.2017.94

[636] Yuan M , Chen X , Sun Y , et al. ZDHHC12‐mediated claudin‐3 S‐palmitoylation determines ovarian cancer progression. Acta Pharm Sin B. 2020;10(8):1426‐1439. 32963941 10.1016/j.apsb.2020.03.008 PMC7488353

[637] Lu F , Shen SH , Wu S , et al. Hypomethylation‐induced prognostic marker zinc finger DHHC‐type palmitoyltransferase 12 contributes to glioblastoma progression. Ann Transl Med. 2022;10(6):334. 35434031 10.21037/atm-22-520 PMC9011314

[638] Dejanovic B , Semtner M , Ebert S , et al. Palmitoylation of gephyrin controls receptor clustering and plasticity of GABAergic synapses. PLoS Biol. 2014;12(7):e1001908. 25025157 10.1371/journal.pbio.1001908 PMC4099074

[639] Chen S , Zhu B , Yin C , et al. Palmitoylation‐dependent activation of MC1R prevents melanomagenesis. Nature. 2017;549(7672):399‐403. 28869973 10.1038/nature23887 PMC5902815

[640] Napoli E , Song G , Liu S , et al. Zdhhc13‐dependent Drp1 S‐palmitoylation impacts brain bioenergetics, anxiety, coordination and motor skills. Sci Rep. 2017;7(1):12796. 29038583 10.1038/s41598-017-12889-0 PMC5643561

[641] Chen LY , Lin KR , Chen YJ , et al. Palmitoyl acyltransferase activity of ZDHHC13 regulates skin barrier development partly by controlling PADi3 and TGM1 protein stability. J Invest Dermatol. 2020;140(5):959‐970. e3. 31669413 10.1016/j.jid.2019.09.017

[642] Shen LF , Chen YJ , Liu KM , et al. Role of S‐palmitoylation by ZDHHC13 in mitochondrial function and metabolism in liver. Sci Rep. 2017;7(1):2182. 28526873 10.1038/s41598-017-02159-4 PMC5438363

[643] Sanders SS , Hernandez LM , Soh H , et al. The palmitoyl acyltransferase ZDHHC14 controls Kv1‐family potassium channel clustering at the axon initial segment. Elife. 2020;9:e56058. 33185190 10.7554/eLife.56058 PMC7685708

[644] Lai Z , Lin P , Weng X , et al. MicroRNA‐574‐5p promotes cell growth of vascular smooth muscle cells in the progression of coronary artery disease. Biomed Pharmacother. 2018;97:162‐167. 29091861 10.1016/j.biopha.2017.10.062

[645] Lewis SA , Bakhtiari S , Heim J , et al. Mutation in ZDHHC15 leads to hypotonic cerebral palsy, autism, epilepsy, and intellectual disability. Neurol Genet. 2021;7(4):e602. 34345675 10.1212/NXG.0000000000000602 PMC8323736

[646] Mejias R , Rodriguez‐Gotor JJ , Niwa M , et al. Increased novelty‐induced locomotion, sensitivity to amphetamine, and extracellular dopamine in striatum of Zdhhc15‐deficient mice. Transl Psychiatry. 2021;11(1):65. 33462194 10.1038/s41398-020-01194-6 PMC7813841

[647] Shah BS , Shimell JJ , Bamji SX . Regulation of dendrite morphology and excitatory synapse formation by zDHHC15. J Cell Sci. 2019;132(13):jcs230052. 31189538 10.1242/jcs.230052 PMC6633394

[648] Fan X , Yang H , Zhao C , et al. Local anesthetics impair the growth and self‐renewal of glioblastoma stem cells by inhibiting ZDHHC15‐mediated GP130 palmitoylation. Stem Cell Res Ther. 2021;12(1):107. 33541421 10.1186/s13287-021-02175-2 PMC7863430

[649] Fan X , Sun S , Yang H , et al. SETD2 palmitoylation mediated by ZDHHC16 in epidermal growth factor receptor‐mutated glioblastoma promotes ionizing radiation‐induced DNA damage. Int J Radiat Oncol Biol Phys. 2022;113(3):648‐660. 35192890 10.1016/j.ijrobp.2022.02.018

[650] Shi W , Chen X , Wang F , et al. ZDHHC16 modulates FGF/ERK dependent proliferation of neural stem/progenitor cells in the zebrafish telencephalon. Dev Neurobiol. 2016;76(9):1014‐1028. 26663717 10.1002/dneu.22372

[651] Cao N , Li JK , Rao YQ , et al. A potential role for protein palmitoylation and zDHHC16 in DNA damage response. BMC Mol Biol. 2016;17(1):12. 27159997 10.1186/s12867-016-0065-9 PMC4862184

[652] Chen X , Hao A , Li X , et al. Activation of JNK and p38 MAPK mediated by ZDHHC17 drives glioblastoma multiforme development and malignant progression. Theranostics. 2020;10(3):998‐1015. 31938047 10.7150/thno.40076 PMC6956818

[653] Nthiga TM , Shrestha BK , Bruun JA , Larsen KB , Lamark T , Johansen T . Regulation of Golgi turnover by CALCOCO1‐mediated selective autophagy. J Cell Biol. 2021;220(6):e202006128. 33871553 10.1083/jcb.202006128 PMC8059076

[654] Niu J , Sanders SS , Jeong HK , et al. Coupled control of distal axon integrity and somal responses to axonal damage by the palmitoyl acyltransferase ZDHHC17. Cell Rep. 2020;33(7):108365. 33207199 10.1016/j.celrep.2020.108365 PMC7803378

[655] Shi C , Yang X , Liu Y , et al. ZDHHC18 negatively regulates cGAS‐mediated innate immunity through palmitoylation. EMBO J. 2022;41(11):e109272. 35438208 10.15252/embj.2021109272 PMC9156970

[656] Pei X , Li KY , Shen Y , et al. Palmitoylation of MDH2 by ZDHHC18 activates mitochondrial respiration and accelerates ovarian cancer growth. Sci China Life Sci. 2022;65(10):2017‐2030. 35366151 10.1007/s11427-021-2048-2

[657] Chen X , Hu L , Yang H , et al. DHHC protein family targets different subsets of glioma stem cells in specific niches. J Exp Clin Cancer Res. 2019;38(1):25. 30658672 10.1186/s13046-019-1033-2 PMC6339410

[658] Wang S , Qiao H , Wang P , Wang Y , Qin D . ZDHHC19 is dispensable for spermatogenesis, but is essential for sperm functions in mice. Int J Mol Sci. 2021;22(16):8894. 34445597 10.3390/ijms22168894 PMC8396176

[659] Wu Y , Zhang X , Zhang X , et al. ZDHHC19 localizes to the cell membrane of spermatids and is involved in spermatogenesis†. Biol Reprod. 2022;106(3):477‐486. 34897408 10.1093/biolre/ioab224

[660] Liang S , Zhang X , Li J . Zinc finger Asp‐His‐His‐Cys palmitoyl ‐acyltransferase 19 accelerates tumor progression through wnt/β‐catenin pathway and is upregulated by miR‐940 in osteosarcoma. Bioengineered. 2022;13(3):7367‐7379. 35297315 10.1080/21655979.2022.2040827 PMC9278973

[661] Kwon H , Choi M , Ahn Y , Jang D , Pak Y . Flotillin‐1 palmitoylation turnover by APT‐1 and ZDHHC‐19 promotes cervical cancer progression by suppressing IGF‐1 receptor desensitization and proteostasis. Cancer Gene Ther. 2022. 10.1038/s41417-022-00546-2 36257975

[662] Mesquita FS , Abrami L , Sergeeva O , et al. S‐acylation controls SARS‐CoV‐2 membrane lipid organization and enhances infectivity. Dev Cell. 2021;56(20):2790‐2807. e8. 34599882 10.1016/j.devcel.2021.09.016 PMC8486083

[663] Carreras‐Sureda A , Abrami L , Ji‐Hee K , et al. S‐acylation by ZDHHC20 targets ORAI1 channels to lipid rafts for efficient Ca(2+) signaling by Jurkat T cell receptors at the immune synapse. Elife. 2021;10:e72051. 34913437 10.7554/eLife.72051 PMC8683079

[664] McMichael TM , Zhang L , Chemudupati M , et al. The palmitoyltransferase ZDHHC20 enhances interferon‐induced transmembrane protein 3 (IFITM3) palmitoylation and antiviral activity. J Biol Chem. 2017;292(52):21517‐21526. 29079573 10.1074/jbc.M117.800482 PMC5766958

[665] Gorinski N , Bijata M , Prasad S , et al. Attenuated palmitoylation of serotonin receptor 5‐HT1A affects receptor function and contributes to depression‐like behaviors. Nat Commun. 2019;10(1):3924. 31477731 10.1038/s41467-019-11876-5 PMC6718429

[666] Haines RJ , Wang CY , Yang CGY , Eitnier RA , Wang F , Wu MH . Targeting palmitoyl acyltransferase ZDHHC21 improves gut epithelial barrier dysfunction resulting from burn‐induced systemic inflammation. Am J Physiol Gastrointest Liver Physiol. 2017;313(6):G549‐g557. 28838985 10.1152/ajpgi.00145.2017 PMC5814670

[667] Marin EP , Jozsef L , Di Lorenzo A , et al. The protein acyl transferase ZDHHC21 modulates α1 adrenergic receptor function and regulates hemodynamics. Arterioscler Thromb Vasc Biol. 2016;36(2):370‐379. 26715683 10.1161/ATVBAHA.115.306942 PMC4984414

[668] Beard RS, Jr. , Yang X , Meegan JE , et al. Palmitoyl acyltransferase DHHC21 mediates endothelial dysfunction in systemic inflammatory response syndrome. Nat Commun. 2016;7:12823. 27653213 10.1038/ncomms12823 PMC5036164

[669] Huang J , Li J , Tang J , et al. ZDHHC22‐mediated mTOR palmitoylation restrains breast cancer growth and endocrine therapy resistance. Int J Biol Sci. 2022;18(7):2833‐2850. 35541896 10.7150/ijbs.70544 PMC9066102

[670] Kim Y , Yang H , Min JK , et al. CCN3 secretion is regulated by palmitoylation via ZDHHC22. Biochem Biophys Res Commun. 2018;495(4):2573‐2578. 29287726 10.1016/j.bbrc.2017.12.128

[671] Duncan PJ , Bi D , McClafferty H , Chen L , Tian L , Shipston MJ . S‐Acylation controls functional coupling of BK channel pore‐forming α‐subunits and β1‐subunits. J Biol Chem. 2019;294(32):12066‐12076. 31213527 10.1074/jbc.RA119.009065 PMC6690687

[672] Tang F , Yang C , Li FP , et al. Palmitoyl transferases act as potential regulators of tumor‐infiltrating immune cells and glioma progression. Mol Ther Nucleic Acids. 2022;28:716‐731. 35664705 10.1016/j.omtn.2022.04.030 PMC9126852

[673] Wirth A , Labus J , Abdel Galil D , et al. Palmitoylation of the small GTPase Cdc42 by DHHC5 modulates spine formation and gene transcription. J Biol Chem. 2022;298(6):102048. 35597282 10.1016/j.jbc.2022.102048 PMC9190017

[674] Ulengin‐Talkish I , Parson MAH , Jenkins ML , et al. Palmitoylation targets the calcineurin phosphatase to the phosphatidylinositol 4‐kinase complex at the plasma membrane. Nat Commun. 2021;12(1):6064. 34663815 10.1038/s41467-021-26326-4 PMC8523714

[675] Zhang Y , Dong D , Xu X , et al. Oxidized high‐density lipoprotein promotes CD36 palmitoylation and increases lipid uptake in macrophages. J Biol Chem. 2022;298(6):102000. 35500650 10.1016/j.jbc.2022.102000 PMC9144050

[676] Hao JW , Wang J , Guo H , et al. CD36 facilitates fatty acid uptake by dynamic palmitoylation‐regulated endocytosis. Nat Commun. 2020;11(1):4765. 32958780 10.1038/s41467-020-18565-8 PMC7505845

[677] Zhao L , Zhang C , Luo X , et al. CD36 palmitoylation disrupts free fatty acid metabolism and promotes tissue inflammation in non‐alcoholic steatohepatitis. J Hepatol. 2018;69(3):705‐717. 29705240 10.1016/j.jhep.2018.04.006

[678] Wang J , Hao JW , Wang X , et al. DHHC4 and DHHC5 facilitate fatty acid uptake by palmitoylating and targeting CD36 to the plasma membrane. Cell Rep. 2019;26(1):209‐221. e5. 30605677 10.1016/j.celrep.2018.12.022

[679] Lu Y , Zheng Y , Coyaud É , et al. Palmitoylation of NOD1 and NOD2 is required for bacterial sensing. Science. 2019;366(6464):460‐467. 31649195 10.1126/science.aau6391

[680] Resh MD . Palmitoylation of proteins in cancer. Biochem Soc Trans. 2017;45(2):409‐416. 28408481 10.1042/BST20160233

[681] Zhang Y , Zhang B , Li Y , et al. Palmitoylation of GNAQ/11 is critical for tumor cell proliferation and survival in GNAQ/11‐mutant uveal melanoma. Front Med. 2022;16(5):784‐798. 35997986 10.1007/s11684-021-0911-0

[682] Chen X , Li H , Fan X , et al. Protein palmitoylation regulates cell survival by modulating XBP1 activity in glioblastoma multiforme. Mol Ther Oncolytics. 2020;17:518‐530. 33024813 10.1016/j.omto.2020.05.007 PMC7525067

[683] Castilla LH . NRAS palmitoylation and oncogenic fitness. Blood. 2020;135(20):1725‐1726. 32407527 10.1182/blood.2020005720

[684] Petit F , Drecourt A , Dussiot M , et al. Defective palmitoylation of transferrin receptor triggers iron overload in Friedreich ataxia fibroblasts. Blood. 2021;137(15):2090‐2102. 33529321 10.1182/blood.2020006987

[685] Wei X , Adak S , Zayed M , et al. Endothelial palmitoylation cycling coordinates vessel remodeling in peripheral artery disease. Circ Res. 2020;127(2):249‐265. 32233916 10.1161/CIRCRESAHA.120.316752 PMC7334103

[686] Zhou L , He X , Wang L , et al. Palmitoylation restricts SQSTM1/p62‐mediated autophagic degradation of NOD2 to modulate inflammation. Cell Death Differ. 2022;29(8):1541‐1551. 35066577 10.1038/s41418-022-00942-z PMC9346120

[687] Mukai K , Konno H , Akiba T , et al. Activation of STING requires palmitoylation at the Golgi. Nat Commun. 2016;7:11932. 27324217 10.1038/ncomms11932 PMC4919521

[688] Mukai J , Tamura M , Fénelon K , et al. Molecular substrates of altered axonal growth and brain connectivity in a mouse model of schizophrenia. Neuron. 2015;86(3):680‐695. 25913858 10.1016/j.neuron.2015.04.003 PMC4603834

[689] Virlogeux A , Scaramuzzino C , Lenoir S , et al. Increasing brain palmitoylation rescues behavior and neuropathology in Huntington disease mice. Sci Adv. 2021;7(14):eabb0799. 33789888 10.1126/sciadv.abb0799 PMC8011966

[690] Ge X , He Z , Cao C , et al. Protein palmitoylation‐mediated palmitic acid sensing causes blood‐testis barrier damage via inducing ER stress. Redox Biol. 2022;54:102380. 35803125 10.1016/j.redox.2022.102380 PMC9287734

[691] Chen X , Niu W , Fan X , et al. Oct4A palmitoylation modulates tumorigenicity and stemness in human glioblastoma cells. Neuro Oncol. 2022. 10.1093/neuonc/noac157 PMC9825352 35727735

[692] Runkle KB , Kharbanda A , Stypulkowski E , et al. Inhibition of DHHC20‐mediated EGFR palmitoylation creates a dependence on EGFR signaling. Mol Cell. 2016;62(3):385‐396. 27153536 10.1016/j.molcel.2016.04.003 PMC4860254

[693] Ali A , Levantini E , Teo JT , et al. Fatty acid synthase mediates EGFR palmitoylation in EGFR mutated non‐small cell lung cancer. EMBO Mol Med. 2018;10(3):e8313. 29449326 10.15252/emmm.201708313 PMC5840543

[694] Beauchamp E , Yap MC , Iyer A , et al. Targeting N‐myristoylation for therapy of B‐cell lymphomas. Nat Commun. 2020;11(1):5348. 33093447 10.1038/s41467-020-18998-1 PMC7582192

[695] Kosciuk T , Price IR , Zhang X , et al. NMT1 and NMT2 are lysine myristoyltransferases regulating the ARF6 GTPase cycle. Nat Commun. 2020;11(1):1067. 32103017 10.1038/s41467-020-14893-x PMC7044312

[696] Burnaevskiy N , Fox TG , Plymire DA , et al. Proteolytic elimination of N‐myristoyl modifications by the Shigella virulence factor IpaJ. Nature. 2013;496(7443):106‐109. 23535599 10.1038/nature12004 PMC3722872

[697] Martin DD , Beauchamp E , Berthiaume LG . Post‐translational myristoylation: fat matters in cellular life and death. Biochimie. 2011;93(1):18‐31. 21056615 10.1016/j.biochi.2010.10.018

[698] Yang SH , Shrivastav A , Kosinski C , et al. N‐myristoyltransferase 1 is essential in early mouse development. J Biol Chem. 2005;280(19):18990‐18995. 15753093 10.1074/jbc.M412917200

[699] Udenwobele DI , Su RC , Good SV , Ball TB , Varma Shrivastav S , Shrivastav A . Myristoylation: an important protein modification in the immune response. Front Immunol. 2017;8:751. 28713376 10.3389/fimmu.2017.00751 PMC5492501

[700] Xiong WH , Qin M , Zhong H . Myristoylation alone is sufficient for PKA catalytic subunits to associate with the plasma membrane to regulate neuronal functions. Proc Natl Acad Sci U S A. 2021;118(15):e2021658118. 33876760 10.1073/pnas.2021658118 PMC8053988

[701] York J , Nunberg JH . Myristoylation of the arenavirus envelope glycoprotein stable signal peptide is critical for membrane fusion but dispensable for virion morphogenesis. J Virol. 2016;90(18):8341‐50. 27412594 10.1128/JVI.01124-16 PMC5008094

[702] Cao J , Qu M , Liu H , et al. Myristoylation of EV71 VP4 is essential for infectivity and interaction with membrane structure. Virol Sin. 2020;35(5):599‐613. 32399947 10.1007/s12250-020-00226-1 PMC7736455

[703] Weyand CM , Goronzy JJ . Immunometabolism in the development of rheumatoid arthritis. Immunol Rev. 2020;294(1):177‐187. 31984519 10.1111/imr.12838 PMC7047523

[704] Wen Z , Jin K , Shen Y , et al. N‐myristoyltransferase deficiency impairs activation of kinase AMPK and promotes synovial tissue inflammation. Nat Immunol. 2019;20(3):313‐325. 30718913 10.1038/s41590-018-0296-7 PMC6396296

[705] Zhang J , Zeng Y , Xing Y , et al. Myristoylation‐mediated phase separation of EZH2 compartmentalizes STAT3 to promote lung cancer growth. Cancer Lett. 2021;516:84‐98. 34102285 10.1016/j.canlet.2021.05.035

[706] Govatati S , Pichavaram P , Janjanam J , Guo L , Virmani R , Rao GN . Myristoylation of LMCD1 leads to its species‐specific derepression of E2F1 and NFATc1 in the modulation of CDC6 and IL‐33 expression during development of vascular lesions. Arterioscler Thromb Vasc Biol. 2020;40(5):1256‐1274. 32160773 10.1161/ATVBAHA.120.314147 PMC7180120

[707] Sun Y , Guan Z , Sheng Q , et al. N‐myristoyltransferase‐1 deficiency blocks myristoylation of LAMTOR1 and inhibits bladder cancer progression. Cancer Lett. 2022;529:126‐138. 34999170 10.1016/j.canlet.2022.01.001

[708] Kim S , Alsaidan OA , Goodwin O , et al. Blocking myristoylation of src inhibits its kinase activity and suppresses prostate cancer progression. Cancer Res. 2017;77(24):6950‐6962. 29038344 10.1158/0008-5472.CAN-17-0981 PMC5732839

[709] Malecki JM , Davydova E , Falnes PO . Protein methylation in mitochondria. J Biol Chem. 2022;298(4):101791. 35247388 10.1016/j.jbc.2022.101791 PMC9006661

[710] Atik AE , Guray MZ , Yalcin T . Observation of the side chain O‐methylation of glutamic acid or aspartic acid containing model peptides by electrospray ionization‐mass spectrometry. J Chromatogr B Analyt Technol Biomed Life Sci. 2017;1047:75‐83. 10.1016/j.jchromb.2016.12.043 28063777

[711] Bedford MT , Clarke SG . Protein arginine methylation in mammals: who, what, and why. Mol Cell. 2009;33(1):1‐13. 19150423 10.1016/j.molcel.2008.12.013 PMC3372459

[712] Martin C , Zhang Y . The diverse functions of histone lysine methylation. Nat Rev Mol Cell Biol. 2005;6(11):838‐849. 16261189 10.1038/nrm1761

[713] Dillon SC , Zhang X , Trievel RC , Cheng X . The SET‐domain protein superfamily: protein lysine methyltransferases. Genome Biol. 2005;6(8):227. 16086857 10.1186/gb-2005-6-8-227 PMC1273623

[714] Falnes PO , Jakobsson ME , Davydova E , Ho A , Malecki J . Protein lysine methylation by seven‐beta‐strand methyltransferases. Biochem J. 2016;473(14):1995‐2009. 27407169 10.1042/BCJ20160117

[715] Dai XJ , Liu Y , Xiong XP , Xue LP , Zheng YC , Liu HM . Tranylcypromine based lysine‐specific demethylase 1 inhibitor: summary and perspective. J Med Chem. 2020;63(23):14197‐14215. 32931269 10.1021/acs.jmedchem.0c00919

[716] Tsukada Y , Fang J , Erdjument‐Bromage H , et al. Histone demethylation by a family of JmjC domain‐containing proteins. Nature. 2006;439(7078):811‐816. 16362057 10.1038/nature04433

[717] Pedersen MT , Helin K . Histone demethylases in development and disease. Trends Cell Biol. 2010;20(11):662‐671. 20863703 10.1016/j.tcb.2010.08.011

[718] Stavropoulos P , Blobel G , Hoelz A . Crystal structure and mechanism of human lysine‐specific demethylase‐1. Nat Struct Mol Biol. 2006;13(7):626‐632. 16799558 10.1038/nsmb1113

[719] Chen F , Yang H , Dong Z , et al. Structural insight into substrate recognition by histone demethylase LSD2/KDM1b. Cell Res. 2013;23(2):306‐309. 23357850 10.1038/cr.2013.17 PMC3567815

[720] Dai X , Ren T , Zhang Y , Nan N . Methylation multiplicity and its clinical values in cancer. Expert Rev Mol Med. 2021;23:e2. 33787478 10.1017/erm.2021.4 PMC8086398

[721] Shalaby NA , Sayed R , Zhang Q , et al. Systematic discovery of genetic modulation by Jumonji histone demethylases in Drosophila. Sci Rep. 2017;7(1):5240. 28701701 10.1038/s41598-017-05004-w PMC5507883

[722] Xu J , Richard S . Cellular pathways influenced by protein arginine methylation: implications for cancer. Mol Cell. 2021;81(21):4357‐4368. 34619091 10.1016/j.molcel.2021.09.011 PMC8571027

[723] Lorton BM , Shechter D . Cellular consequences of arginine methylation. Cell Mol Life Sci. 2019;76(15):2933‐2956. 31101937 10.1007/s00018-019-03140-2 PMC6642692

[724] Wang Y , Wysocka J , Sayegh J , et al. Human PAD4 regulates histone arginine methylation levels via demethylimination. Science. 2004;306(5694):279‐283. 15345777 10.1126/science.1101400

[725] Zhang J , Jing L , Li M , He L , Guo Z . Regulation of histone arginine methylation/demethylation by methylase and demethylase (Review). Mol Med Rep. 2019;19(5):3963‐3971. 30942418 10.3892/mmr.2019.10111 PMC6471501

[726] Ng SS , Yue WW , Oppermann U , Klose RJ . Dynamic protein methylation in chromatin biology. Cell Mol Life Sci. 2009;66(3):407‐422. 18923809 10.1007/s00018-008-8303-z PMC2794343

[727] Chang B , Chen Y , Zhao Y , Bruick RK . JMJD6 is a histone arginine demethylase. Science. 2007;318(5849):444‐447. 17947579 10.1126/science.1145801

[728] Greer EL , Shi Y . Histone methylation: a dynamic mark in health, disease and inheritance. Nat Rev Genet. 2012;13(5):343‐357. 22473383 10.1038/nrg3173 PMC4073795

[729] Hyun K , Jeon J , Park K , Kim J . Writing, erasing and reading histone lysine methylations. Exp Mol Med. 2017;49(4):e324. 28450737 10.1038/emm.2017.11 PMC6130214

[730] Chen YC , Hsu PY , Chin CH , et al. H3K23/H3K36 hypoacetylation and HDAC1 up‐regulation are associated with adverse consequences in obstructive sleep apnea patients. Sci Rep. 2021;11(1):20697. 34667186 10.1038/s41598-021-00052-9 PMC8526826

[731] Rice JC , Briggs SD , Ueberheide B , et al. Histone methyltransferases direct different degrees of methylation to define distinct chromatin domains. Mol Cell. 2003;12(6):1591‐1598. 14690610 10.1016/s1097-2765(03)00479-9

[732] Zhou X , Chen H , Li J , Shi Y , Zhuang S , Liu N . The role and mechanism of lysine methyltransferase and arginine methyltransferase in kidney diseases. Front Pharmacol. 2022;13:885527. 35559246 10.3389/fphar.2022.885527 PMC9086358

[733] Boisvert F‐M , Rhie A , Richard S , Doherty AJ . The GAR motif of 53BP1 is arginine methylated by PRMT1 and is necessary for 53BP1 DNA binding activity. Cell Cycle. 2005;4(12):1834‐1841. 16294045 10.4161/cc.4.12.2250

[734] Dai W , Zhang J , Li S , et al. Protein arginine methylation: an emerging modification in cancer immunity and immunotherapy. Front Immunol. 2022;13:865964. 35493527 10.3389/fimmu.2022.865964 PMC9046588

[735] Carlson SM , Gozani O . Nonhistone lysine methylation in the regulation of cancer pathways. Cold Spring Harb Perspect Med. 2016;6(11):a026435. 27580749 10.1101/cshperspect.a026435 PMC5088510

[736] Di Blasi R , Blyuss O , Timms JF , Conole D , Ceroni F , Whitwell HJ . Non‐histone protein methylation: biological significance and bioengineering potential. ACS Chem Biol. 2021;16(2):238‐250. 33411495 10.1021/acschembio.0c00771

[737] Biggar KK , Li SS . Non‐histone protein methylation as a regulator of cellular signalling and function. Nat Rev Mol Cell Biol. 2015;16(1):5‐17. 25491103 10.1038/nrm3915

[738] Zhang X , Wen H , Shi X . Lysine methylation: beyond histones. Acta Biochim Biophys Sin (Shanghai). 2012;44(1):14‐27. 22194010 10.1093/abbs/gmr100

[739] Saddic LA , West LE , Aslanian A , et al. Methylation of the retinoblastoma tumor suppressor by SMYD2. J Biol Chem. 2010;285(48):37733‐37740. 20870719 10.1074/jbc.M110.137612 PMC2988378

[740] Bhat KP , Umit Kaniskan H , Jin J , Gozani O . Epigenetics and beyond: targeting writers of protein lysine methylation to treat disease. Nat Rev Drug Discov. 2021;20(4):265‐286. 33469207 10.1038/s41573-020-00108-x PMC8035164

[741] Gyory I , Wu J , Fejer G , Seto E , Wright KL . PRDI‐BF1 recruits the histone H3 methyltransferase G9a in transcriptional silencing. Nat Immunol. 2004;5(3):299‐308. 14985713 10.1038/ni1046

[742] Purcell DJ , Jeong KW , Bittencourt D , Gerke DS , Stallcup MR . A distinct mechanism for coactivator versus corepressor function by histone methyltransferase G9a in transcriptional regulation. J Biol Chem. 2011;286(49):41963‐41971. 21984853 10.1074/jbc.M111.298463 PMC3234941

[743] Hwang S , Kim S , Kim K , Yeom J , Park S , Kim I . Euchromatin histone methyltransferase II (EHMT2) regulates the expression of ras‐related GTP binding C (RRAGC) protein. BMB Rep. 2020;53(11):576‐581. 32684241 10.5483/BMBRep.2020.53.11.055 PMC7704221

[744] Zeng TB , Han L , Pierce N , Pfeifer GP , Szabó PE . EHMT2 and SETDB1 protect the maternal pronucleus from 5mC oxidation. Proc Natl Acad Sci U S A. 2019;116(22):10834‐10841. 31088968 10.1073/pnas.1819946116 PMC6561192

[745] Ling BM , Bharathy N , Chung TK , et al. Lysine methyltransferase G9a methylates the transcription factor MyoD and regulates skeletal muscle differentiation. Proc Natl Acad Sci U S A. 2012;109(3):841‐846. 22215600 10.1073/pnas.1111628109 PMC3271886

[746] Nishiguchi MA , Spencer CA , Leung DH , Leung TH . Aging suppresses skin‐derived circulating SDF1 to promote full‐thickness tissue regeneration. Cell Rep. 2018;24(13):3383‐3392. e5. 30257200 10.1016/j.celrep.2018.08.054 PMC6261459

[747] He J , Kallin EM , Tsukada Y , Zhang Y . The H3K36 demethylase Jhdm1b/Kdm2b regulates cell proliferation and senescence through p15(Ink4b). Nat Struct Mol Biol. 2008;15(11):1169‐1175. 18836456 10.1038/nsmb.1499 PMC2612995

[748] Tzatsos A , Pfau R , Kampranis SC , Tsichlis PN . Ndy1/KDM2B immortalizes mouse embryonic fibroblasts by repressing the Ink4a/Arf locus. Proc Natl Acad Sci U S A. 2009;106(8):2641‐2646. 19202064 10.1073/pnas.0813139106 PMC2650317

[749] Chen F , Chen J , Wang H , et al. Histone lysine methyltransferase SETD2 regulates coronary vascular development in embryonic mouse hearts. Front Cell Dev Biol. 2021;9:651655. 33898448 10.3389/fcell.2021.651655 PMC8063616

[750] Cheung MY‐Q , Roberts C , Scambler P , Stathopoulou A . Setd5 is required in cardiopharyngeal mesoderm for heart development and its haploinsufficiency is associated with outflow tract defects in mouse. Genesis. 2021;59(7‐8):e23421. 34050709 10.1002/dvg.23421 PMC8564859

[751] Lee J , Shao N‐Y , Paik DT , et al. SETD7 drives cardiac lineage commitment through stage‐specific transcriptional activation. Cell Stem Cell. 2018;22(3):428‐444. e5. 29499155 10.1016/j.stem.2018.02.005 PMC5929163

[752] Kim J‐D , Kim E , Koun S , et al. Proper activity of histone H3 lysine 4 (H3K4) methyltransferase is required for morphogenesis during zebrafish cardiogenesis. Mol Cells. 2015;38(6):580‐586. 25997738 10.14348/molcells.2015.0053 PMC4469916

[753] Jiang X , Li T , Sun J , Liu J , Wu H . SETD3 negatively regulates VEGF expression during hypoxic pulmonary hypertension in rats. Hypertens Res. 2018;41(9):691‐698. 29950684 10.1038/s41440-018-0068-7

[754] Zhou X‐L , Huang F‐J , Li Y , Huang H , Wu Q‐C . SEDT2/METTL14‐mediated m6A methylation awakening contributes to hypoxia‐induced pulmonary arterial hypertension in mice. Aging. 2021;13(5):7538‐7548. 33658391 10.18632/aging.202616 PMC7993666

[755] Ugai K , Matsuda S , Mikami H , et al. Inhibition of the SET8 pathway ameliorates lung fibrosis even through fibroblast dedifferentiation. Front Mol Biosci. 2020;7:192. 32850975 10.3389/fmolb.2020.00192 PMC7419601

[756] Elkouris M , Kontaki H , Stavropoulos A , et al. SET9‐mediated regulation of TGF‐β signaling links protein methylation to pulmonary fibrosis. Cell Rep. 2016;15(12):2733‐2744. 27292644 10.1016/j.celrep.2016.05.051 PMC4920893

[757] Yu S , Li Y , Zhao H , Wang Q , Chen P . The histone demethylase JMJD1C regulates CAMKK2‐AMPK signaling to participate in cardiac hypertrophy. Front Physiol. 2020;11:539. 32625104 10.3389/fphys.2020.00539 PMC7314990

[758] Cheng Y , Chen Y , Wang G , et al. Protein methylation in diabetic kidney disease. Front Med (Lausanne). 2022;9:736006. 35647002 10.3389/fmed.2022.736006 PMC9133329

[759] Choi D , Oh KJ , Han HS , et al. Protein arginine methyltransferase 1 regulates hepatic glucose production in a FoxO1‐dependent manner. Hepatology. 2012;56(4):1546‐1556. 22532369 10.1002/hep.25809

[760] Kanamaluru D , Xiao Z , Fang S , et al. Arginine methylation by PRMT5 at a naturally occurring mutation site is critical for liver metabolic regulation by small heterodimer partner. Mol Cell Biol. 2011;31(7):1540‐1550. 21262773 10.1128/MCB.01212-10 PMC3135303

[761] Paneni F , Costantino S , Battista R , et al. Adverse epigenetic signatures by histone methyltransferase Set7 contribute to vascular dysfunction in patients with type 2 diabetes mellitus. Circ Cardiovasc Genet. 2015;8(1):150‐158. 25472959 10.1161/CIRCGENETICS.114.000671

[762] Keating ST , El‐Osta A . Chromatin modifications associated with diabetes. J Cardiovasc Transl Res. 2012;5(4):399‐412. 22639343 10.1007/s12265-012-9380-9

[763] Natarajan R . Epigenetic mechanisms in diabetic vascular complications and metabolic memory: The 2020 Edwin Bierman Award Lecture. Diabetes. 2021;70(2):328‐337. 33472942 10.2337/dbi20-0030 PMC7881871

[764] Okabe J , Orlowski C , Balcerczyk A , et al. Distinguishing hyperglycemic changes by Set7 in vascular endothelial cells. Circ Res. 2012;110(8):1067‐1076. 22403242 10.1161/CIRCRESAHA.112.266171

[765] Chen X , Wu Q , Jiang H , et al. SET8 is involved in the regulation of hyperglycemic memory in human umbilical endothelial cells. Acta Biochim Biophys Sin (Shanghai). 2018;50(7):635‐642. 29762637 10.1093/abbs/gmy051

[766] Reintjes A , Fuchs JE , Kremser L , et al. Asymmetric arginine dimethylation of RelA provides a repressive mark to modulate TNFα/NF‐κB response. Proc Natl Acad Sci U S A. 2016;113(16):4326‐4331. 27051065 10.1073/pnas.1522372113 PMC4843428

[767] Tanaka Y , Nagai Y , Okumura M , Greene MI , Kambayashi T . PRMT5 is required for T cell survival and proliferation by maintaining cytokine signaling. Front Immunol. 2020;11:621. 32328070 10.3389/fimmu.2020.00621 PMC7160866

[768] Srour N , Khan S , Richard S . The Influence of Arginine Methylation in Immunity and Inflammation. J Inflamm Res. 2022;15:2939‐2958. 35602664 10.2147/JIR.S364190 PMC9114649

[769] Snyder KJ , Zitzer NC , Gao Y , et al. PRMT5 regulates T cell interferon response and is a target for acute graft‐versus‐host disease. JCI Insight. 2020;5(8):e131099. 32191634 10.1172/jci.insight.131099 PMC7205431

[770] Caporali R , Bugatti S , Bruschi E , Cavagna L , Montecucco C . Autoantibodies to heterogeneous nuclear ribonucleoproteins. Autoimmunity. 2005;38(1):25‐32. 15804702 10.1080/08916930400022590

[771] Rowe EM , Xing V , Biggar KK . Lysine methylation: Implications in neurodegenerative disease. Brain Res. 2019;1707:164‐171. 30465751 10.1016/j.brainres.2018.11.024

[772] Quan X , Yue W , Luo Y , et al. The protein arginine methyltransferase PRMT5 regulates Abeta‐induced toxicity in human cells and Caenorhabditis elegans models of Alzheimer's disease. J Neurochem. 2015;134(5):969‐977. 26086249 10.1111/jnc.13191

[773] Labonne JDJ , Lee K‐H , Iwase S , et al. An atypical 12q24.31 microdeletion implicates six genes including a histone demethylase KDM2B and a histone methyltransferase SETD1B in syndromic intellectual disability. Hum Genet. 2016;135(7):757‐771. 27106595 10.1007/s00439-016-1668-4

[774] Chen X , Wang S , Zhou Y , et al. Phf8 histone demethylase deficiency causes cognitive impairments through the mTOR pathway. Nat Commun. 2018;9(1):114. 29317619 10.1038/s41467-017-02531-y PMC5760733

[775] Ratovitski T , Arbez N , Stewart JC , Chighladze E , Ross CA . PRMT5‐ mediated symmetric arginine dimethylation is attenuated by mutant huntingtin and is impaired in Huntington's disease (HD). Cell Cycle. 2015;14(11):1716‐1729. 25927346 10.1080/15384101.2015.1033595 PMC4615115

[776] Sugeno N , Jäckel S , Voigt A , Wassouf Z , Schulze‐Hentrich J , Kahle PJ . α‐Synuclein enhances histone H3 lysine‐9 dimethylation and H3K9me2‐dependent transcriptional responses. Sci Rep. 2016;6:36328. 27808254 10.1038/srep36328 PMC5093762

[777] Elakoum R , Gauchotte G , Oussalah A , et al. CARM1 and PRMT1 are dysregulated in lung cancer without hierarchical features. Biochimie. 2014;97:210‐218. 24211191 10.1016/j.biochi.2013.10.021

[778] Yoshimatsu M , Toyokawa G , Hayami S , et al. Dysregulation of PRMT1 and PRMT6, Type I arginine methyltransferases, is involved in various types of human cancers. Int J Cancer. 2011;128(3):562‐573. 20473859 10.1002/ijc.25366

[779] Baldwin RM , Morettin A , Côté J . Role of PRMTs in cancer: Could minor isoforms be leaving a mark? World J Biol Chem. 2014;5(2):115‐129. 24921003 10.4331/wjbc.v5.i2.115 PMC4050107

[780] Li Z , Wang D , Lu J , et al. Methylation of EZH2 by PRMT1 regulates its stability and promotes breast cancer metastasis. Cell Death Differ. 2020;27(12):3226‐3242. 32895488 10.1038/s41418-020-00615-9 PMC7853151

[781] Hua Z‐Y , Hansen JN , He M , et al. PRMT1 promotes neuroblastoma cell survival through ATF5. Oncogenesis. 2020;9(5):50. 32415090 10.1038/s41389-020-0237-9 PMC7229216

[782] Wang J , Wang C , Xu P , et al. PRMT1 is a novel molecular therapeutic target for clear cell renal cell carcinoma. Theranostics. 2021;11(11):5387‐5403. 33859753 10.7150/thno.42345 PMC8039964

[783] Cao H , Li L , Yang D , et al. Recent progress in histone methyltransferase (G9a) inhibitors as anticancer agents. Eur J Med Chem. 2019;179:537‐546. 31276898 10.1016/j.ejmech.2019.06.072

[784] Fu L , Wu H , Cheng SY , Gao D , Zhang L , Zhao Y . Set7 mediated Gli3 methylation plays a positive role in the activation of Sonic Hedgehog pathway in mammals. Elife. 2016;5:e15690. 27146893 10.7554/eLife.15690 PMC4884081

[785] Bottino C , Peserico A , Simone C , Caretti GA‐O . SMYD3: an oncogenic driver targeting epigenetic regulation and signaling pathways. Cancers (Basel). 2020;12(2072‐6694 (Print)):142. 31935919 10.3390/cancers12010142 PMC7017119

[786] Watanabe T , Kobunai T , Yamamoto Y , et al. Differential gene expression signatures between colorectal cancers with and without KRAS mutations: Crosstalk between the KRAS pathway and other signalling pathways. Eur J Cancer. 2011;47(13):1946‐1954. 21531130 10.1016/j.ejca.2011.03.029

[787] Hamamoto R , Furukawa Y , Fau‐Morita M , et al. SMYD3 encodes a histone methyltransferase involved in the proliferation of cancer cells. Nat Cell Biol. 2004;6(1465‐7392 (Print)):731‐740. 15235609 10.1038/ncb1151

[788] Mazur PK , Reynoird N , Khatri P , et al. SMYD3 links lysine methylation of MAP3K2 to Ras‐driven cancer. Nature. 2014;510(1476‐4687 (Electronic)):283‐287. 24847881 10.1038/nature13320 PMC4122675

[789] Vieira FQ , Costa‐Pinheiro P , Almeida‐Rios D , et al. SMYD3 contributes to a more aggressive phenotype of prostate cancer and targets Cyclin D2 through H4K20me3. Oncotarget. 2015;6(1949‐2553 (Electronic)):13644‐13657. 25980436 10.18632/oncotarget.3767 PMC4537039

[790] Hall RK , Wang XL , George L , Koch SR , Granner DK . Insulin represses phosphoenolpyruvate carboxykinase gene transcription by causing the rapid disruption of an active transcription complex: a potential epigenetic effect. Mol Endocrinol. 2007;21(2):550‐563. 17095578 10.1210/me.2006-0307

[791] Yang Y , Luan Y , Yuan R‐X , Luan Y . Histone methylation related therapeutic challenge in cardiovascular diseases. Front Cardiovasc Med. 2021;8:710053. 34568453 10.3389/fcvm.2021.710053 PMC8458636

[792] Zhuang L , Jang Y , Park Y‐K , et al. Depletion of Nsd2‐mediated histone H3K36 methylation impairs adipose tissue development and function. Nat Commun. 2018;9(1):1796. 29728617 10.1038/s41467-018-04127-6 PMC5935725

[793] Lu L , Li X , Zhong Z , et al. KMT5A downregulation participated in high glucose‐mediated EndMT via upregulation of ENO1 expression in diabetic nephropathy. Int J Biol Sci. 2021;17(15):4093‐4107. 34803485 10.7150/ijbs.62867 PMC8579450

[794] Schliehe C , Flynn EK , Vilagos B , et al. The methyltransferase Setdb2 mediates virus‐induced susceptibility to bacterial superinfection. Nat Immunol. 2015;16(1):67‐74. 25419628 10.1038/ni.3046 PMC4320687

[795] Yan Z , Wu H , Liu H , et al. The protein arginine methyltransferase PRMT1 promotes TBK1 activation through asymmetric arginine methylation. Cell Rep. 2021;36(12):109731. 34551290 10.1016/j.celrep.2021.109731

[796] von Schimmelmann M , Feinberg PA , Sullivan JM , et al. Polycomb repressive complex 2 (PRC2) silences genes responsible for neurodegeneration. Nat Neurosci. 2016;19(10):1321‐1330. 27526204 10.1038/nn.4360 PMC5088783

[797] Zheng Y , Liu A , Wang Z‐J , et al. Inhibition of EHMT1/2 rescues synaptic and cognitive functions for Alzheimer's disease. Brain. 2019;142(3):787‐807. 30668640 10.1093/brain/awy354 PMC6391616

[798] Guhathakurta S , Kim J , Adams L , et al. Targeted attenuation of elevated histone marks at SNCA alleviates α‐synuclein in Parkinson's disease. EMBO Mol Med. 2021;13(2):e12188. 33428332 10.15252/emmm.202012188 PMC7863397

[799] Mu M‐D , Qian Z‐M , Yang S‐X , Rong K‐L , Yung W‐H , Ke Y . Therapeutic effect of a histone demethylase inhibitor in Parkinson's disease. Cell Death Dis. 2020;11(10):927. 33116116 10.1038/s41419-020-03105-5 PMC7595123

[800] Belzil VV , Bauer PO , Prudencio M , et al. Reduced C9orf72 gene expression in c9FTD/ALS is caused by histone trimethylation, an epigenetic event detectable in blood. Acta Neuropathol. 2013;126(6):895‐905. 24166615 10.1007/s00401-013-1199-1 PMC3830740

[801] Liao Y , Gou L , Chen L , et al. NADPH oxidase 4 and endothelial nitric oxide synthase contribute to endothelial dysfunction mediated by histone methylations in metabolic memory. Free Radic Biol Med. 2018;115:383‐394. 29269309 10.1016/j.freeradbiomed.2017.12.017

[802] Zhang C , Ge S , Gong W , et al. LncRNA ANRIL acts as a modular scaffold of WDR5 and HDAC3 complexes and promotes alteration of the vascular smooth muscle cell phenotype. Cell Death Dis. 2020;11(6):435. 32513988 10.1038/s41419-020-2645-3 PMC7280314

[803] Papait R , Serio S , Pagiatakis C , et al. Histone methyltransferase G9a is required for cardiomyocyte homeostasis and hypertrophy. Circulation. 2017;136(13):1233‐1246. 28778944 10.1161/CIRCULATIONAHA.117.028561

[804] Cai S , Wang P , Xie T , et al. Histone H4R3 symmetric di‐methylation by Prmt5 protects against cardiac hypertrophy via regulation of Filip1L/β‐catenin. Pharmacol Res. 2020;161:105104. 32739429 10.1016/j.phrs.2020.105104

[805] Wang YF , Zhang J , Su Y , et al. G9a regulates breast cancer growth by modulating iron homeostasis through the repression of ferroxidase hephaestin. Nat Commun. 2017;3789(1):3789. 10.1038/s41467-017-00350-9 PMC5561105 28819251

[806] Tsang JY , Lai S‐T , Ni Y‐B , et al. SETD2 alterations and histone H3K36 trimethylation in phyllodes tumor of breast. Breast Cancer Res Treat. 2021;187(2):339‐347. 33844099 10.1007/s10549-021-06181-z

[807] Wei L , Chiu DK‐C , Tsang FH‐C , et al. Histone methyltransferase G9a promotes liver cancer development by epigenetic silencing of tumor suppressor gene RARRES3. J Hepatol. 2017;67(4):758‐769. 28532996 10.1016/j.jhep.2017.05.015

[808] Chen B‐Y , Song J , Hu C‐L , et al. SETD2 deficiency accelerates MDS‐associated leukemogenesis via S100a9 in NHD13 mice and predicts poor prognosis in MDS. Blood. 2020;135(25):2271‐2285. 32202636 10.1182/blood.2019001963 PMC7316210

[809] Wu X , Li R , Song Q , et al. JMJD2C promotes colorectal cancer metastasis via regulating histone methylation of MALAT1 promoter and enhancing β‐catenin signaling pathway. J Exp Clin Cancer Res. 2019;38(1):435. 31665047 10.1186/s13046-019-1439-x PMC6819649

[810] Niu N , Lu P , Yang Y , et al. Loss of Setd2 promotes Kras‐induced acinar‐to‐ductal metaplasia and epithelia‐mesenchymal transition during pancreatic carcinogenesis. Gut. 2020;69(4):715‐726. 31300513 10.1136/gutjnl-2019-318362

[811] Zhou Y , Zheng X , Xu B , Deng H , Chen L , Jiang J . Histone methyltransferase SETD2 inhibits tumor growth via suppressing CXCL1‐mediated activation of cell cycle in lung adenocarcinoma. Aging. 2020;12(24):25189‐25206. 33223508 10.18632/aging.104120 PMC7803529

[812] Gulati N , Beguelin W , Giulino‐Roth L . Enhancer of zeste homolog 2 (EZH2) inhibitors. Leuk Lymphoma. 2018;59(7):1574‐1585. 29473431 10.1080/10428194.2018.1430795 PMC6659997

[813] Lue JK , Amengual JE . Emerging EZH2 inhibitors and their application in lymphoma. Curr Hematol Malig Rep. 2018;13(5):369‐382. 30112706 10.1007/s11899-018-0466-6

[814] Venkatesan N , Wong JF , Tan KP , et al. EZH2 promotes neoplastic transformation through VAV interaction‐dependent extranuclear mechanisms. Oncogene. 2018;37(4):461‐477. 28967906 10.1038/onc.2017.309

[815] Hamamoto R , Nakamura Y . Dysregulation of protein methyltransferases in human cancer: an emerging target class for anticancer therapy. Cancer Sci. 2016;107(4):377‐84. 26751963 10.1111/cas.12884 PMC4832871

[816] Fedoriw A , Shi L , O'Brien S , et al. Inhibiting type I arginine methyltransferase activity promotes T cell‐mediated antitumor immune responses. Cancer Immunol Res. 2022;10(4):420‐436. 35181787 10.1158/2326-6066.CIR-21-0614 PMC8976792

[817] Jarrold J , Davies CC . PRMTs and arginine methylation: cancer's best‐kept secret? Trends Mol Med. 2019;25(11):993‐1009. 31230909 10.1016/j.molmed.2019.05.007

[818] Liu B , Ruan J , Chen M , et al. Deubiquitinating enzymes (DUBs): decipher underlying basis of neurodegenerative diseases. Mol Psychiatry. 2022;27(1):259‐268. 34285347 10.1038/s41380-021-01233-8

[819] Tracz M , Bialek W . Beyond K48 and K63: non‐canonical protein ubiquitination. Cell Mol Biol Lett. 2021;26(1):1. 33402098 10.1186/s11658-020-00245-6 PMC7786512

[820] Jussupow A , Messias AC , Stehle R , et al. The dynamics of linear polyubiquitin. Sci Adv. 2020;6(42):eabc3786. 33055165 10.1126/sciadv.abc3786 PMC7556843

[821] Callis J . The ubiquitination machinery of the ubiquitin system. Arabidopsis Book. 2014;12:e0174. 25320573 10.1199/tab.0174 PMC4196676

[822] Buneeva OA , Medvedeva MV , Kopylov AT , Medvedev AE . Ubiquitin subproteome of brain mitochondria and its changes induced by experimental parkinsonism and action of neuroprotectors. Biochemistry (Mosc). 2019;84(11):1359‐1374. 31760923 10.1134/S0006297919110117

[823] Foot N , Henshall T , Kumar S . Ubiquitination and the regulation of membrane proteins. Physiol Rev. 2017;97(1):253‐281. 27932395 10.1152/physrev.00012.2016

[824] Liu W , Tang X , Qi X , et al. The ubiquitin conjugating enzyme: an important ubiquitin transfer platform in ubiquitin‐proteasome system. Int J Mol Sci. 2020;21(8):2894. 32326224 10.3390/ijms21082894 PMC7215765

[825] Lange SM , Armstrong LA , Kulathu Y . Deubiquitinases: From mechanisms to their inhibition by small molecules. Mol Cell. 2022;82(1):15‐29. 34813758 10.1016/j.molcel.2021.10.027

[826] Hu Y , Bai X , Zhang C , et al. Ubiquitination‐activating enzymes UBE1 and UBA6 regulate ubiquitination and expression of cardiac sodium channel Nav1.5. Biochem J. 2020;477(9):1683‐1700. 32315024 10.1042/BCJ20200138

[827] Barghout SH , Schimmer AD . E1 enzymes as therapeutic targets in cancer. Pharmacol Rev. 2021;73(1):1‐58. 10.1124/pharmrev.120.000053 33177128

[828] Stewart MD , Ritterhoff T , Klevit RE , Brzovic PS . E2 enzymes: more than just middle men. Cell Res. 2016;26(4):423‐440. 27002219 10.1038/cr.2016.35 PMC4822130

[829] Bricelj A , Steinebach C , Kuchta R , Gutschow M , Sosic I . E3 ligase ligands in successful PROTACs: an overview of syntheses and linker attachment points. Front Chem. 2021;9:707317. 34291038 10.3389/fchem.2021.707317 PMC8287636

[830] Buetow L , Huang DT . Structural insights into the catalysis and regulation of E3 ubiquitin ligases. Nat Rev Mol Cell Biol. 2016;17(10):626‐642. 27485899 10.1038/nrm.2016.91 PMC6211636

[831] Hermanns T , Pichlo C , Baumann U , Hofmann K . A structural basis for the diverse linkage specificities within the ZUFSP deubiquitinase family. Nat Commun. 2022;13(1):401. 35058438 10.1038/s41467-022-28049-6 PMC8776766

[832] Mevissen TET , Komander D . Mechanisms of deubiquitinase specificity and regulation. Annu Rev Biochem. 2017;86(1):159‐192. 28498721 10.1146/annurev-biochem-061516-044916

[833] Akutsu M , Dikic I , Bremm A . Ubiquitin chain diversity at a glance. J Cell Sci. 2016;129(5):875‐80. 26906419 10.1242/jcs.183954

[834] Popovic D , Vucic D , Dikic I . Ubiquitination in disease pathogenesis and treatment. Nat Med. 2014;20(11):1242‐1253. 25375928 10.1038/nm.3739

[835] Mukhopadhyay D , Riezman H . Proteasome‐independent functions of ubiquitin in endocytosis and signaling. Science. 2007;315(5809):201‐205. 17218518 10.1126/science.1127085

[836] Ohtake F , Saeki Y , Ishido S , Kanno J , Tanaka K . The K48‐K63 branched ubiquitin chain regulates NF‐kappaB signaling. Mol Cell. 2016;64(2):251‐266. 27746020 10.1016/j.molcel.2016.09.014

[837] Ohtake F , Tsuchiya H , Saeki Y , Tanaka K . K63 ubiquitylation triggers proteasomal degradation by seeding branched ubiquitin chains. Proc Natl Acad Sci U S A. 2018;115(7):E1401‐E1408. 29378950 10.1073/pnas.1716673115 PMC5816176

[838] Swatek KN , Komander D . Ubiquitin modifications. Cell Res. 2016;26(4):399‐422. 27012465 10.1038/cr.2016.39 PMC4822133

[839] Yau R , Rape M . The increasing complexity of the ubiquitin code. Nat Cell Biol. 2016;18(6):579‐586. 27230526 10.1038/ncb3358

[840] Husnjak K , Dikic I . Ubiquitin‐binding proteins: decoders of ubiquitin‐mediated cellular functions. Annu Rev Biochem. 2012;81:291‐322. 22482907 10.1146/annurev-biochem-051810-094654

[841] Komander D , Rape M . The ubiquitin code. Annu Rev Biochem. 2012;81:203‐229. 22524316 10.1146/annurev-biochem-060310-170328

[842] van Huizen M , Kikkert M . The role of atypical ubiquitin chains in the regulation of the antiviral innate immune response. Front Cell Dev Biol. 2019;7:392. 32039206 10.3389/fcell.2019.00392 PMC6987411

[843] Gatti M , Pinato S , Maiolica A , et al. RNF168 promotes noncanonical K27 ubiquitination to signal DNA damage. Cell Rep. 2015;10(2):226‐238. 25578731 10.1016/j.celrep.2014.12.021

[844] Gerlach B , Cordier SM , Schmukle AC , et al. Linear ubiquitination prevents inflammation and regulates immune signalling. Nature. 2011;471(7340):591‐596. 21455173 10.1038/nature09816

[845] Yang Q , Zhao J , Chen D , Wang Y . E3 ubiquitin ligases: styles, structures and functions. Mol Biomed. 2021;2(1):23. 35006464 10.1186/s43556-021-00043-2 PMC8607428

[846] Elia AE , Boardman AP , Wang DC , et al. Quantitative proteomic atlas of ubiquitination and acetylation in the DNA damage response. Mol Cell. 2015;59(5):867‐881. 26051181 10.1016/j.molcel.2015.05.006 PMC4560960

[847] Gersch M , Gladkova C , Schubert AF , Michel MA , Maslen S , Komander D . Mechanism and regulation of the Lys6‐selective deubiquitinase USP30. Nat Struct Mol Biol. 2017;24(11):920‐930. 28945249 10.1038/nsmb.3475 PMC5757785

[848] Yau RG , Doerner K , Castellanos ER , et al. Assembly and function of heterotypic ubiquitin chains in cell‐cycle and protein quality control. Cell. 2017;171(4):918‐933. e20. 29033132 10.1016/j.cell.2017.09.040 PMC5669814

[849] Yu Y , Zheng Q , Erramilli SK , et al. K29‐linked ubiquitin signaling regulates proteotoxic stress response and cell cycle. Nat Chem Biol. 2021;17(8):896‐905. 34239127 10.1038/s41589-021-00823-5 PMC8717942

[850] Yuan WC , Lee YR , Lin SY , et al. K33‐linked polyubiquitination of coronin 7 by Cul3‐KLHL20 ubiquitin E3 ligase regulates protein trafficking. Mol Cell. 2014;54(4):586‐600. 24768539 10.1016/j.molcel.2014.03.035

[851] Emmerich CH , Ordureau A , Strickson S , et al. Activation of the canonical IKK complex by K63/M1‐linked hybrid ubiquitin chains. Proc Natl Acad Sci U S A. 2013;110(38):15247‐15252. 23986494 10.1073/pnas.1314715110 PMC3780889

[852] Swatek KN , Usher JL , Kueck AF , et al. Insights into ubiquitin chain architecture using Ub‐clipping. Nature. 2019;572(7770):533‐537. 31413367 10.1038/s41586-019-1482-y PMC6823057

[853] Wang YS , Wu KP , Jiang HK , Kurkute P , Chen RH . Branched ubiquitination: detection methods, biological functions and chemical synthesis. Molecules. 2020;25(21):5200. 33182242 10.3390/molecules25215200 PMC7664869

[854] Malynn BA , Ma A . Ubiquitin makes its mark on immune regulation. Immunity. 2010;33(6):843‐852. 21168777 10.1016/j.immuni.2010.12.007 PMC3030984

[855] Park CW , Ryu KY . Cellular ubiquitin pool dynamics and homeostasis. BMB Rep. 2014;47(9):475‐482. 24924398 10.5483/BMBRep.2014.47.9.128 PMC4206721

[856] Wang J , Zhou Q , Ding J , Yin T , Ye P , Zhang Y . The conceivable functions of protein ubiquitination and deubiquitination in reproduction. Front Physiol. 2022;13:886261. 35910557 10.3389/fphys.2022.886261 PMC9326170

[857] Alpaugh WF , Voigt AL , Dardari R , et al. Loss of ubiquitin carboxy‐terminal hydrolase L1 impairs long‐term differentiation competence and metabolic regulation in murine spermatogonial stem cells. Cells. 2021;10(9):2265. 34571914 10.3390/cells10092265 PMC8465610

[858] Hashimoto M , Kimura S , Kanno C , et al. Macrophage ubiquitin‐specific protease 2 contributes to motility, hyperactivation, capacitation, and in vitro fertilization activity of mouse sperm. Cell Mol Life Sci. 2021;78(6):2929‐2948. 33104844 10.1007/s00018-020-03683-9 PMC11073191

[859] Oss‐Ronen L , Sarusi T , Cohen I . Histone mono‐ubiquitination in transcriptional regulation and its mark on life: emerging roles in tissue development and disease. Cells. 2022;11(15):2404. 35954248 10.3390/cells11152404 PMC9368181

[860] Li X , Gong L , Gu H . Regulation of immune system development and function by Cbl‐mediated ubiquitination. Immunol Rev. 2019;291(1):123‐133. 31402498 10.1111/imr.12789

[861] Fernando R , Drescher C , Nowotny K , Grune T , Castro JP . Impaired proteostasis during skeletal muscle aging. Free Radic Biol Med. 2019;132:58‐66. 30194981 10.1016/j.freeradbiomed.2018.08.037

[862] Kevei É , Hoppe T . Ubiquitin sets the timer: impacts on aging and longevity. Nat Struct Mol Biol. 2014;21(4):290‐292. 24699075 10.1038/nsmb.2806

[863] Koyuncu S , Loureiro R , Lee HJ , Wagle P , Krueger M , Vilchez D . Rewiring of the ubiquitinated proteome determines ageing in C. elegans. Nature. 2021;596(7871):285‐290. 34321666 10.1038/s41586-021-03781-z PMC8357631

[864] Gao B , Yu W , Lv P , Liang X , Sun S , Zhang Y . Parkin overexpression alleviates cardiac aging through facilitating K63‐polyubiquitination of TBK1 to facilitate mitophagy. Biochim Biophys Acta Mol Basis Dis. 2021;1867(1):165997. 33164878 10.1016/j.bbadis.2020.165997

[865] Basisty N , Meyer JG , Schilling B . Protein turnover in aging and longevity. Proteomics. 2018;18(5‐6):e1700108. 29453826 10.1002/pmic.201700108 PMC6022828

[866] Ullah K , Chen S , Lu J , et al. The E3 ubiquitin ligase STUB1 attenuates cell senescence by promoting the ubiquitination and degradation of the core circadian regulator BMAL1. J Biol Chem. 2020;295(14):4696‐4708. 32041778 10.1074/jbc.RA119.011280 PMC7135990

[867] Kwon J , Han E , Bui CB , et al. Assurance of mitochondrial integrity and mammalian longevity by the p62‐Keap1‐Nrf2‐Nqo1 cascade. EMBO Rep. 2012;13(2):150‐156. 22222206 10.1038/embor.2011.246 PMC3271336

[868] Silva‐Palacios A , Ostolga‐Chavarria M , Zazueta C , Konigsberg M . Nrf2: molecular and epigenetic regulation during aging. Ageing Res Rev. 2018;47:31‐40. 29913211 10.1016/j.arr.2018.06.003

[869] Kwon SK , Lee DH , Kim SY , Park JH , Choi J , Baek KH . Ubiquitin‐specific protease 21 regulating the K48‐linked polyubiquitination of NANOG. Biochem Biophys Res Commun. 2017;482(4):1443‐1448. 27956178 10.1016/j.bbrc.2016.12.055

[870] Ben‐Neriah Y . Regulatory functions of ubiquitination in the immune system. Nat Immunol. 2002;3(1):20‐26. 11753406 10.1038/ni0102-20

[871] Martín‐Vicente M , Resino S , Martínez I . Early innate immune response triggered by the human respiratory syncytial virus and its regulation by ubiquitination/deubiquitination processes. J Biomed Sci. 2022;29(1):11. 35152905 10.1186/s12929-022-00793-3 PMC8841119

[872] Qi F , Zhang X , Wang L , et al. E3 ubiquitin ligase NEURL3 promotes innate antiviral response through catalyzing K63‐linked ubiquitination of IRF7. FASEB J. 2022;36(8):e22409. 35792897 10.1096/fj.202200316R PMC12166272

[873] Li L , Luo J , Zhu Z , et al. SRA suppresses antiviral innate immune response in macrophages by limiting TBK1 K63 ubiquitination via deubiquitinase USP15. Microbiol Spectr. 2022;10(6):e0202822. 36342281 10.1128/spectrum.02028-22 PMC9769732

[874] Xiao Y , Jin J , Chang M , et al. Peli1 promotes microglia‐mediated CNS inflammation by regulating Traf3 degradation. Nat Med. 2013;19(5):595‐602. 23603814 10.1038/nm.3111 PMC3899792

[875] Chang M , Jin W , Chang J‐H , et al. The ubiquitin ligase Peli1 negatively regulates T cell activation and prevents autoimmunity. Nat Immunol. 2011;12(10):1002‐1009. 21874024 10.1038/ni.2090 PMC3178748

[876] Hage A , Bharaj P , van Tol S , et al. The RNA helicase DHX16 recognizes specific viral RNA to trigger RIG‐I‐dependent innate antiviral immunity. Cell Rep. 2022;38(10):110434. 35263596 10.1016/j.celrep.2022.110434 PMC8903195

[877] Zhong T , Lei K , Lin X , et al. Protein ubiquitination in T cell development. Front Immunol. 2022;13:941962. 35990660 10.3389/fimmu.2022.941962 PMC9386135

[878] Zhang T , Sun J , Cheng J , et al. The role of ubiquitinase in B cell development and function. J Leukoc Biol. 2021;109(2):395‐405. 32816356 10.1002/JLB.1MR0720-185RR

[879] Liu Y , Liang Q‐Z , Lu W , et al. A comparative analysis of coronavirus nucleocapsid (N) proteins reveals the SADS‐CoV N protein antagonizes IFN‐β production by inducing ubiquitination of RIG‐I. Front Immunol. 2021;12:688758. 34220846 10.3389/fimmu.2021.688758 PMC8242249

[880] Jiang J , Li Y , Sun Z , et al. LncNSPL facilitates influenza A viral immune escape by restricting TRIM25‐mediated K63‐linked RIG‐I ubiquitination. iScience. 2022;25(7):104607. 35800772 10.1016/j.isci.2022.104607 PMC9253711

[881] Dou Y , Xie Y , Zhang L , et al. Host MKRN1‐mediated mycobacterial PPE protein ubiquitination suppresses innate immune response. Front Immunol. 2022;13:880315. 35603194 10.3389/fimmu.2022.880315 PMC9114769

[882] Hou J , Han L , Zhao Z , et al. USP18 positively regulates innate antiviral immunity by promoting K63‐linked polyubiquitination of MAVS. Nat Commun. 2021;12(1):2970. 34016972 10.1038/s41467-021-23219-4 PMC8137702

[883] Zhang Z‐D , Xiong T‐C , Yao S‐Q , et al. RNF115 plays dual roles in innate antiviral responses by catalyzing distinct ubiquitination of MAVS and MITA. Nat Commun. 2020;11(1):5536. 33139700 10.1038/s41467-020-19318-3 PMC7606512

[884] Kamoshita K , Ishii K‐A , Tahira Y , et al. Insulin suppresses ubiquitination via the deubiquitinating enzyme USP14, independent of proteasome activity in H4IIEC3 hepatocytes. J Pharmacol Exp Ther. 2022. 10.1124/jpet.122.001088 36328485

[885] Li X , Wang T , Tao Y , Wang X , Li L , Liu J . Inhibition of USP7 suppresses advanced glycation end‐induced cell cycle arrest and senescence of human umbilical vein endothelial cells through ubiquitination of p53. Acta Biochim Biophys Sin (Shanghai). 2022;54(3):311‐320. 35538032 10.3724/abbs.2022003 PMC9828104

[886] Guo Y , Li J , Fan S , Hu Q . Suppressive role of E3 ubiquitin ligase FBW7 in type I diabetes in non‐obese diabetic mice through mediation of ubiquitination of EZH2. Cell Death Dis. 2021;7(1):361. 10.1038/s41420-021-00605-x PMC8606006 34802056

[887] Song R , Peng W , Zhang Y , et al. Central role of E3 ubiquitin ligase MG53 in insulin resistance and metabolic disorders. Nature. 2013;494(7437):375‐379. 23354051 10.1038/nature11834

[888] Li X , Sun X , Li L , Luo Y , Chi Y , Zheng G . MDM2‐mediated ubiquitination of LKB1 contributes to the development of diabetic cataract. Exp Cell Res. 2022;417(1):113191. 35513074 10.1016/j.yexcr.2022.113191

[889] Aye ILMH , Rosario FJ , Kramer A , et al. Insulin increases adipose adiponectin in pregnancy by inhibiting ubiquitination and degradation: impact of obesity. J Clin Endocrinol Metab. 2022;107(1):53‐66. 34519830 10.1210/clinem/dgab680 PMC8684469

[890] Schwartz AL , Ciechanover A . Targeting proteins for destruction by the ubiquitin system: implications for human pathobiology. Annu Rev Pharmacol Toxicol. 2009;49:73‐96. 18834306 10.1146/annurev.pharmtox.051208.165340

[891] Sun Y . Targeting E3 ubiquitin ligases for cancer therapy. Cancer Biol Ther. 2003;2(6):623‐629. 14688465

[892] Wang Z , Liu P , Inuzuka H , Wei W . Roles of F‐box proteins in cancer. Nat Rev Cancer. 2014;14(4):233‐247. 24658274 10.1038/nrc3700 PMC4306233

[893] Schmidt MHH , Dikic I . The Cbl interactome and its functions. Nat Rev Mol Cell Biol. 2005;6(12):907‐918. 16227975 10.1038/nrm1762

[894] Kao H‐W , Ogawa S , Sanada M , et al. Roles of TET2 and C‐CBL mutations in the progression of de novo myelodysplastic syndrome to acute myeloid leukemia. Blood. 2010;116(21):4019‐4019.

[895] Bedford L , Lowe J , Dick LR , Mayer RJ , Brownell JE . Ubiquitin‐like protein conjugation and the ubiquitin‐proteasome system as drug targets. Nat Rev Drug Discov. 2011;10(1):29‐46. 21151032 10.1038/nrd3321 PMC7097807

[896] Liu W , Yan B , Yu H , et al. OTUD1 stabilizes PTEN to inhibit the PI3K/AKT and TNF‐alpha/NF‐kappaB signaling pathways and sensitize ccRCC to TKIs. Int J Biol Sci. 2022;18(4):1401‐1414. 35280681 10.7150/ijbs.68980 PMC8898358

[897] Zhang M‐H , Zhang H‐H , Du X‐H , et al. UCHL3 promotes ovarian cancer progression by stabilizing TRAF2 to activate the NF‐κB pathway. Oncogene. 2020;39(2):322‐333. 31477831 10.1038/s41388-019-0987-z

[898] Qi SM , Cheng G , Cheng XD , et al. Targeting USP7‐mediated deubiquitination of MDM2/MDMX‐p53 pathway for cancer therapy: are we there yet? Front Cell Dev Biol. 2020;8:233. 32300595 10.3389/fcell.2020.00233 PMC7142254

[899] Yuan J , Luo K , Zhang L , Cheville JC , Lou Z . USP10 regulates p53 localization and stability by deubiquitinating p53. Cell. 2010;140(3):384‐396. 20096447 10.1016/j.cell.2009.12.032 PMC2820153

[900] Yuan T , Chen Z , Yan F , et al. Deubiquitinating enzyme USP10 promotes hepatocellular carcinoma metastasis through deubiquitinating and stabilizing Smad4 protein. Mol Oncol. 2020;14(1):197‐210. 31721429 10.1002/1878-0261.12596 PMC6944132

[901] Zhang X , Berger FG , Yang J , Lu X . USP4 inhibits p53 through deubiquitinating and stabilizing ARF‐BP1. EMBO J. 2011;30(11):2177‐2189. 21522127 10.1038/emboj.2011.125 PMC3117646

[902] Wang Y , Zhou L , Lu J , et al. Ubiquitin‐specific protease 4 predicts an unfavorable prognosis and promotes malignant behaviors in vitro in pancreatic cancer. Exp Cell Res. 2020;396(2):112317. 33038351 10.1016/j.yexcr.2020.112317

[903] Diao W , Guo Q , Zhu C , et al. USP18 promotes cell proliferation and suppressed apoptosis in cervical cancer cells via activating AKT signaling pathway. BMC Cancer. 2020;20(1):741. 32770981 10.1186/s12885-020-07241-1 PMC7414560

[904] Shen J , Hong L , Chen L . Ubiquitin‐specific protease 14 regulates ovarian cancer cisplatin‐resistance by stabilizing BCL6 oncoprotein. Biochem Biophys Res Commun. 2020;524(3):683‐688. 32033748 10.1016/j.bbrc.2020.01.150

[905] Bai Z , Du Y , Cong L , Cheng Y . The USP22 promotes the growth of cancer cells through the DYRK1A in pancreatic ductal adenocarcinoma. Gene. 2020;758:144960. 32687947 10.1016/j.gene.2020.144960

[906] Dou N , Hu Q , Li L , Wu Q , Li Y , Gao Y . USP32 promotes tumorigenesis and chemoresistance in gastric carcinoma via upregulation of SMAD2. Int J Biol Sci. 2020;16(9):1648‐1657. 32226309 10.7150/ijbs.43117 PMC7097920

[907] Li Y , Xu Y , Gao C , et al. USP1 maintains the survival of liver circulating tumor cells by deubiquitinating and stabilizing TBLR1. Front Oncol. 2020;10:554809. 33102219 10.3389/fonc.2020.554809 PMC7545832

[908] Xiong B , Huang J , Liu Y , et al. Ubiquitin‐specific protease 2a promotes hepatocellular carcinoma progression via deubiquitination and stabilization of RAB1A. Cell Oncol (Dordr). 2021;44(2):329‐343. 33074477 10.1007/s13402-020-00568-8 PMC12980744

[909] Sasaki AT , Carracedo A , Locasale JW , et al. Ubiquitination of K‐Ras enhances activation and facilitates binding to select downstream effectors. Sci Signal. 2011;4(163):ra13. 21386094 10.1126/scisignal.2001518 PMC3437993

[910] Galasso G , De Rosa R , Piscione F , et al. Myocardial expression of FOXO3a‐Atrogin‐1 pathway in human heart failure. Eur J Heart Fail. 2010;12(12):1290‐1296. 21098579 10.1093/eurjhf/hfq102

[911] Predmore JM , Wang P , Davis F , et al. Ubiquitin proteasome dysfunction in human hypertrophic and dilated cardiomyopathies. Circulation. 2010;121(8):997‐1004. 20159828 10.1161/CIRCULATIONAHA.109.904557 PMC2857348

[912] Powell SR , Herrmann J , Lerman A , Patterson C , Wang X . The ubiquitin‐proteasome system and cardiovascular disease. Prog Mol Biol Transl Sci. 2012;109:295‐346. 22727426 10.1016/B978-0-12-397863-9.00009-2 PMC3743449

[913] Song J , Zhu Y , Li J , et al. Pellino1‐mediated TGF‐β1 synthesis contributes to mechanical stress induced cardiac fibroblast activation. J Mol Cell Cardiol. 2015;79:145‐156. 25446187 10.1016/j.yjmcc.2014.11.006

[914] Bennett EJ , Bence NF , Jayakumar R , Kopito RR . Global impairment of the ubiquitin‐proteasome system by nuclear or cytoplasmic protein aggregates precedes inclusion body formation. Mol Cell. 2005;17(3):351‐365. 15694337 10.1016/j.molcel.2004.12.021

[915] Silva MVF , Loures CdMG , Alves LCV , de Souza LC , Borges KBG , Carvalho MdG . Alzheimer's disease: risk factors and potentially protective measures. J Biomed Sci. 2019;26(1):33. 31072403 10.1186/s12929-019-0524-y PMC6507104

[916] Tai HC , Serrano‐Pozo A , Hashimoto T , Frosch MP , Spires‐Jones TL , Hyman BT . The synaptic accumulation of hyperphosphorylated tau oligomers in Alzheimer disease is associated with dysfunction of the ubiquitin‐proteasome system. Am J Pathol. 2012;181(4):1426‐1435. 22867711 10.1016/j.ajpath.2012.06.033 PMC3463637

[917] Wang Y , Martinez‐Vicente M , Kruger U , et al. Tau fragmentation, aggregation and clearance: the dual role of lysosomal processing. Hum Mol Genet. 2009;18(21):4153‐4170. 19654187 10.1093/hmg/ddp367 PMC2758146

[918] van Leeuwen FW , de Kleijn DP , van den Hurk HH , et al. Frameshift mutants of beta amyloid precursor protein and ubiquitin‐B in Alzheimer's and down patients. Science. 1998;279(5348):242‐247. 9422699 10.1126/science.279.5348.242

[919] Chadwick L , Gentle L , Strachan J , Layfield R . Review: unchained maladie ‐ a reassessment of the role of Ubb(+1) ‐capped polyubiquitin chains in Alzheimer's disease. Neuropathol Appl Neurobiol. 2012;38(2):118‐131. 22082077 10.1111/j.1365-2990.2011.01236.x

[920] Song S , Kim S‐Y , Hong Y‐M , et al. Essential role of E2‐25K/Hip‐2 in mediating amyloid‐beta neurotoxicity. Mol Cell. 2003;12(3):553‐563. 14527403 10.1016/j.molcel.2003.08.005

[921] Singh AK , Pati U . CHIP stabilizes amyloid precursor protein via proteasomal degradation and p53‐mediated trans‐repression of beta‐secretase. Aging Cell. 2015;14(4):595‐604. 25773675 10.1111/acel.12335 PMC4531073

[922] Okamoto T , Imaizumi K , Kaneko M . The role of tissue‐specific ubiquitin ligases, RNF183, RNF186, RNF182 and RNF152, in disease and biological function. Int J Mol Sci. 2020;21(11):3921. 32486221 10.3390/ijms21113921 PMC7313026

[923] Lonskaya I , Hebron ML , Desforges NM , Schachter JB , Moussa CE . Nilotinib‐induced autophagic changes increase endogenous parkin level and ubiquitination, leading to amyloid clearance. J Mol Med (Berl). 2014;92(4):373‐386. 24337465 10.1007/s00109-013-1112-3 PMC3975659

[924] Gerakis Y , Dunys J , Bauer C , Checler F . Abeta42 oligomers modulate beta‐secretase through an XBP‐1s‐dependent pathway involving HRD1. Sci Rep. 2016;6:37436. 27853315 10.1038/srep37436 PMC5112606

[925] Zhang M , Cai F , Zhang S , Zhang S , Song W . Overexpression of ubiquitin carboxyl‐terminal hydrolase L1 (UCHL1) delays Alzheimer's progression in vivo. Sci Rep. 2014;4:7298. 25466238 10.1038/srep07298 PMC4252905

[926] Chauhan M , Modi PK , Sharma P . Aberrant activation of neuronal cell cycle caused by dysregulation of ubiquitin ligase Itch results in neurodegeneration. Cell Death Dis. 2020;11(6):441. 32513985 10.1038/s41419-020-2647-1 PMC7280246

[927] Owais A , Mishra RK , Kiyokawa H . The HECT E3 ligase E6AP/UBE3A as a therapeutic target in cancer and neurological disorders. Cancers. 2020;12(8)2108. 32751183 10.3390/cancers12082108 PMC7464832

[928] Ndoja A , Reja R , Lee S‐H , et al. Ubiquitin ligase COP1 suppresses neuroinflammation by degrading c/EBPβ in microglia. Cell. 2020;182(5):1156‐1169. 32795415 10.1016/j.cell.2020.07.011

[929] Xu J , Yu T , Pietronigro EC , et al. Peli1 impairs microglial Aβ phagocytosis through promoting C/EBPβ degradation. PLoS Biol. 2020;18(10):e3000837. 33017390 10.1371/journal.pbio.3000837 PMC7561136

[930] Canever JB , Soares ES , de Avelar NCP , Cimarosti HI . Targeting α‐synuclein post‐translational modifications in Parkinson's disease. Behav Brain Res. 2022:114204. 36372243 10.1016/j.bbr.2022.114204

[931] Lee JT , Wheeler TC , Li L , Chin LS . Ubiquitination of alpha‐synuclein by Siah‐1 promotes alpha‐synuclein aggregation and apoptotic cell death. Hum Mol Genet. 2008;17(6):906‐917. 18065497 10.1093/hmg/ddm363

[932] Rott R , Szargel R , Haskin J , et al. Monoubiquitylation of alpha‐synuclein by seven in absentia homolog (SIAH) promotes its aggregation in dopaminergic cells. J Biol Chem. 2008;283(6):3316‐3328. 18070888 10.1074/jbc.M704809200

[933] Engelender S . Ubiquitination of alpha‐synuclein and autophagy in Parkinson's disease. Autophagy. 2008;4(3):372‐374. 18216494 10.4161/auto.5604

[934] Scotter EL , Chen HJ , Shaw CE . TDP‐43 proteinopathy and ALS: insights into disease mechanisms and therapeutic targets. Neurotherapeutics. 2015;12(2):352‐363. 25652699 10.1007/s13311-015-0338-x PMC4404432

[935] Nakayama Y , Tsuji K , Ayaki T , Mori M , Tokunaga F , Ito H . Linear polyubiquitin chain modification of TDP‐43‐positive neuronal cytoplasmic inclusions in amyotrophic lateral sclerosis. J Neuropathol Exp Neurol. 2020;79(3):256‐265. 31951008 10.1093/jnen/nlz135

[936] Lee BH , Lee MJ , Park S , et al. Enhancement of proteasome activity by a small‐molecule inhibitor of USP14. Nature. 2010;467(7312):179‐184. 20829789 10.1038/nature09299 PMC2939003

[937] Hans F , Fiesel FC , Strong JC , et al. UBE2E ubiquitin‐conjugating enzymes and ubiquitin isopeptidase Y regulate TDP‐43 protein ubiquitination. J Biol Chem. 2014;289(27):19164‐19179. 24825905 10.1074/jbc.M114.561704 PMC4081952

[938] Ullah K , Chen S , Lu J , et al. Correction: the E3 ubiquitin ligase STUB1 attenuates cell senescence by promoting the ubiquitination and degradation of the core circadian regulator BMAL1. J Biol Chem. 2020;295(32):11378. 32769176 10.1074/jbc.AAC120.015160 PMC7415962

[939] Baldewijns MM , van Vlodrop IJ , Vermeulen PB , Soetekouw PM , van Engeland M , de Bruine AP . VHL and HIF signalling in renal cell carcinogenesis. J Pathol. 2010;221(2):125‐138. 20225241 10.1002/path.2689

[940] Gonzalez‐Barbosa E , Mejia‐Garcia A , Bautista E , Gonzalez FJ , Segovia J , Elizondo G . TCDD induces UbcH7 expression and synphilin‐1 protein degradation in the mouse ventral midbrain. J Biochem Mol Toxicol. 2017;31(10). 10.1002/jbt.21947 PMC6309283 28621812

[941] Zhang Q , Terawaki S , Oikawa D , et al. Suppression of linear ubiquitination ameliorates cytoplasmic aggregation of truncated TDP‐43. Cells. 2022;11(15):2398. 35954242 10.3390/cells11152398 PMC9367985

[942] Barley K , He W , Agarwal S , Jagannath S , Chari A . Outcomes and management of lenalidomide‐associated rash in patients with multiple myeloma. Leuk Lymphoma. 2016;57(11):2510‐2515. 26943456 10.3109/10428194.2016.1151507

[943] Fischer ES , Bohm K , Lydeard JR , et al. Structure of the DDB1‐CRBN E3 ubiquitin ligase in complex with thalidomide. Nature. 2014;512(7512):49‐53. 25043012 10.1038/nature13527 PMC4423819

[944] Gombodorj N , Yokobori T , Yoshiyama S , et al. Inhibition of ubiquitin‐conjugating enzyme E2 may activate the degradation of hypoxia‐inducible factors and, thus, overcome cellular resistance to radiation in colorectal cancer. Anticancer Res. 2017;37(5):2425‐2436. 28476810 10.21873/anticanres.11582

[945] Gollner A , Rudolph D , Arnhof H , et al. Discovery of novel spiro[3H‐indole‐3,2'‐pyrrolidin]‐2(1H)‐one compounds as chemically stable and orally active inhibitors of the MDM2‐p53 interaction. J Med Chem. 2016;59(22):10147‐10162. 27775892 10.1021/acs.jmedchem.6b00900

[946] Wu K , Huynh KQ , Lu I , et al. Inhibitors of cullin‐RING E3 ubiquitin ligase 4 with antitumor potential. Proc Natl Acad Sci U S A. 2021;118(8):e2007328118. 33602808 10.1073/pnas.2007328118 PMC7923628

[947] Tamanini E , Buck IM , Chessari G , et al. Discovery of a potent nonpeptidomimetic, small‐molecule antagonist of cellular inhibitor of apoptosis protein 1 (cIAP1) and X‐linked inhibitor of apoptosis protein (XIAP). J Med Chem. 2017;60(11):4611‐4625. 28492317 10.1021/acs.jmedchem.6b01877

[948] Frost J , Galdeano C , Soares P , et al. Potent and selective chemical probe of hypoxic signalling downstream of HIF‐alpha hydroxylation via VHL inhibition. Nat Commun. 2016;7:13312. 27811928 10.1038/ncomms13312 PMC5097156

[949] Chuang SJ , Cheng SC , Tang HC , Sun CY , Chou CY . 6‐Thioguanine is a noncompetitive and slow binding inhibitor of human deubiquitinating protease USP2. Sci Rep. 2018;8(1):3102. 29449607 10.1038/s41598-018-21476-w PMC5814560

[950] Li P , Liu HM . Recent advances in the development of ubiquitin‐specific‐processing protease 7 (USP7) inhibitors. Eur J Med Chem. 2020;191:112107. 32092586 10.1016/j.ejmech.2020.112107

[951] Pal A , Dziubinski M , Di Magliano MP , et al. Usp9x promotes survival in human pancreatic cancer and its inhibition suppresses pancreatic ductal adenocarcinoma in vivo tumor growth. Neoplasia. 2018;20(2):152‐164. 29248719 10.1016/j.neo.2017.11.007 PMC5735260

[952] Boselli M , Lee BH , Robert J , et al. An inhibitor of the proteasomal deubiquitinating enzyme USP14 induces tau elimination in cultured neurons. J Biol Chem. 2017;292(47):19209‐19225. 28972160 10.1074/jbc.M117.815126 PMC5702663

[953] Jing C , Li X , Zhou M , et al. The PSMD14 inhibitor Thiolutin as a novel therapeutic approach for esophageal squamous cell carcinoma through facilitating SNAIL degradation. Theranostics. 2021;11(12):5847‐5862. 33897885 10.7150/thno.46109 PMC8058732

[954] Farrell BM , Gerth F , Yang C‐HR , Yeh JTH . A synthetic KLHL20 ligand to validate CUL3 as a potent E3 ligase for targeted protein degradation. Genes Dev. 2022;36(17‐18):1031‐1042. 36328355 10.1101/gad.349717.122 PMC9732910

[955] Zhou X , Dong R , Zhang JY , Zheng X , Sun LP . PROTAC: a promising technology for cancer treatment. Eur J Med Chem. 2020;203:112539. 32698111 10.1016/j.ejmech.2020.112539

[956] Paiva SL , Crews CM . Targeted protein degradation: elements of PROTAC design. Curr Opin Chem Biol. 2019;50:111‐119. 31004963 10.1016/j.cbpa.2019.02.022 PMC6930012

[957] Zeng S , Huang W , Zheng X , et al. Proteolysis targeting chimera (PROTAC) in drug discovery paradigm: recent progress and future challenges. Eur J Med Chem. 2021;210:112981. 33160761 10.1016/j.ejmech.2020.112981

[958] Zhou XL , Zhao F , Xu YT , et al. A comprehensive review of BET‐targeting PROTACs for cancer therapy. Bioorg Med Chem. 2022;73:117033. 36202064 10.1016/j.bmc.2022.117033

[959] Burslem GM , Crews CM . Proteolysis‐targeting chimeras as therapeutics and tools for biological discovery. Cell. 2020;181(1):102‐114. 31955850 10.1016/j.cell.2019.11.031 PMC7319047

[960] Webb T , Craigon C , Ciulli A . Targeting epigenetic modulators using PROTAC degraders: current status and future perspective. Bioorg Med Chem Lett. 2022;63:128653. 35257896 10.1016/j.bmcl.2022.128653

[961] Qi SM , Dong J , Xu ZY , Cheng XD , Zhang WD , Qin JJ . PROTAC: an effective targeted protein degradation strategy for cancer therapy. Front Pharmacol. 2021;12:692574. 34025443 10.3389/fphar.2021.692574 PMC8138175

[962] Kargbo RB . PROTAC compounds targeting androgen receptor for cancer therapeutics: prostate cancer and kennedy's disease. ACS Med Chem Lett. 2020;11(6):1092‐1093. 32550986 10.1021/acsmedchemlett.0c00236 PMC7294551

[963] Qin H , Zhang Y , Lou Y , et al. Overview of PROTACs targeting the estrogen receptor: achievements for biological and drug discovery. Curr Med Chem. 2022;29(22):3922‐3944. 34758713 10.2174/0929867328666211110101018

[964] Vertegaal ACO . Signalling mechanisms and cellular functions of SUMO. Nat Rev Mol Cell Biol. 2022;23(11):715‐731. 35750927 10.1038/s41580-022-00500-y

[965] Wu H , Chen X , Cheng J , Qi Y . SUMOylation and potassium channels: links to epilepsy and sudden death. Adv Protein Chem Struct Biol. 2016;103:295‐321. 26920693 10.1016/bs.apcsb.2015.11.009

[966] Chang HM , Yeh ETH . SUMO: from bench to bedside. Physiol Rev. 2020;100(4):1599‐1619. 32666886 10.1152/physrev.00025.2019 PMC7717128

[967] Yuan Y , Gaither K , Kim E , et al. SUMO2/3 modification of activating transcription factor 5 (ATF5) controls its dynamic translocation at the centrosome. J Biol Chem. 2018;293(8):2939‐2948. 29326161 10.1074/jbc.RA117.001151 PMC5827429

[968] Han ZJ , Feng YH , Gu BH , Li YM , Chen H . The post‐translational modification, SUMOylation, and cancer (Review). Int J Oncol. 2018;52(4):1081‐1094. 29484374 10.3892/ijo.2018.4280 PMC5843405

[969] Hirano S , Udagawa O . SUMOylation regulates the number and size of promyelocytic leukemia‐nuclear bodies (PML‐NBs) and arsenic perturbs SUMO dynamics on PML by insolubilizing PML in THP‐1 cells. Arch Toxicol. 2022;96(2):545‐558. 35001170 10.1007/s00204-021-03195-w

[970] Liang YC , Lee CC , Yao YL , Lai CC , Schmitz ML , Yang WM . SUMO5, a novel poly‐SUMO isoform, regulates PML nuclear bodies. Sci Rep. 2016;6:26509. 27211601 10.1038/srep26509 PMC4876461

[971] Sundvall M , Korhonen A , Vaparanta K , et al. Protein inhibitor of activated STAT3 (PIAS3) protein promotes SUMOylation and nuclear sequestration of the intracellular domain of ErbB4 protein. J Biol Chem. 2012;287(27):23216‐23226. 22584572 10.1074/jbc.M111.335927 PMC3391121

[972] Tokarz P , Wozniak K . SENP proteases as potential targets for cancer therapy. Cancers (Basel). 2021;13(9):2059. 33923236 10.3390/cancers13092059 PMC8123143

[973] Liu Y , Liu F , Wang L , et al. Localization analysis of seven de‐sumoylation enzymes (SENPs) in ocular cell lines. Curr Mol Med. 2018;18(8):523‐532. 30636609 10.2174/1566524019666190112142025

[974] Xiao H , Zhou H , Zeng G , Mao Z , Zeng J , Gao A . SUMOylation targeting mitophagy in cardiovascular diseases. J Mol Med (Berl). 2022;100(11):1511‐1538. 36163375 10.1007/s00109-022-02258-4

[975] Yeh ETH . SUMOylation and De‐SUMOylation: wrestling with life's processes. J Biol Chem. 2009;284(13):8223‐8227. 19008217 10.1074/jbc.R800050200 PMC2659178

[976] Flotho A , Melchior F . Sumoylation: a regulatory protein modification in health and disease. Annu Rev Biochem. 2013;82:357‐385. 23746258 10.1146/annurev-biochem-061909-093311

[977] Cong L , Pakala SB , Ohshiro K , Li DQ , Kumar R . SUMOylation and SUMO‐interacting motif (SIM) of metastasis tumor antigen 1 (MTA1) synergistically regulate its transcriptional repressor function. J Biol Chem. 2011;286(51):43793‐43808. 21965678 10.1074/jbc.M111.267237 PMC3243521

[978] Müller S , Hoege C , Pyrowolakis G , Jentsch S . SUMO, ubiquitin's mysterious cousin. Nat Rev Mol Cell Biol. 2001;2(3):202‐210. 11265250 10.1038/35056591

[979] Ulrich HD . Mutual interactions between the SUMO and ubiquitin systems: a plea of no contest. Trends Cell Biol. 2005;15(10):525‐532. 16125934 10.1016/j.tcb.2005.08.002

[980] K ST , Joshi G , Arya P , Mahajan V , Chaturvedi A , Mishra RK . SUMO and SUMOylation pathway at the forefront of host immune response. Front Cell Dev Biol. 2021;9:681057. 34336833 10.3389/fcell.2021.681057 PMC8316833

[981] Wang X‐D , Gong Y , Chen Z‐L , et al. TCR‐induced sumoylation of the kinase PKC‐θ controls T cell synapse organization and T cell activation. Nat Immunol. 2015;16(11):1195‐1203. 26390157 10.1038/ni.3259

[982] Fan Y , Li X , Zhang L , et al. SUMOylation in viral replication and antiviral defense. Adv Sci (Weinh). 2022;9(7):e2104126. 35060688 10.1002/advs.202104126 PMC8895153

[983] Dehnavi S , Sadeghi M , Johnston TP , Barreto G , Shohan M , Sahebkar A . The role of protein SUMOylation in rheumatoid arthritis. J Autoimmun. 2019;102:1‐7. 31078376 10.1016/j.jaut.2019.05.006

[984] Lao M , Zhan Z , Li N , et al. Role of small ubiquitin‐like modifier proteins‐1 (SUMO‐1) in regulating migration and invasion of fibroblast‐like synoviocytes from patients with rheumatoid arthritis. Exp Cell Res. 2019;375(1):52‐61. 30562482 10.1016/j.yexcr.2018.12.011

[985] Mustfa SA , Singh M , Suhail A , et al. SUMOylation pathway alteration coupled with downregulation of SUMO E2 enzyme at mucosal epithelium modulates inflammation in inflammatory bowel disease. Open Biol. 2017;7(6):170024. 28659381 10.1098/rsob.170024 PMC5493774

[986] Cao J , Courey AJ . SUMO in drosophila development. Adv Exp Med Biol. 2017;963:249‐257. 28197917 10.1007/978-3-319-50044-7_15

[987] Broday L . The SUMO system in Caenorhabditis elegans development. Int J Dev Biol. 2017;61(3‐4‐5):159‐164. 28621413 10.1387/ijdb.160388LB

[988] Wen B , Yuan H , Liu X , et al. GATA5 SUMOylation is indispensable for zebrafish cardiac development. Biochim Biophys Acta Gen Subj. 2017;1861(7):1691‐1701. 28285006 10.1016/j.bbagen.2017.03.005

[989] Bertke MM , Dubiak KM , Cronin L , Zeng E , Huber PW . A deficiency in SUMOylation activity disrupts multiple pathways leading to neural tube and heart defects in Xenopus embryos. BMC Genomics. 2019;20(1):386. 31101013 10.1186/s12864-019-5773-3 PMC6525467

[990] Kitagawa T , Takiya S . Regulation of genes for ubiquitination and SUMO‐specific protease involved in larval development of the silkworm, Bombyx mori. Dev Growth Differ. 2020;62(6):438‐449. 32573769 10.1111/dgd.12687

[991] Elrouby N . Regulation of plant cellular and organismal development by SUMO. Adv Exp Med Biol. 2017;963:227‐247. 28197916 10.1007/978-3-319-50044-7_14

[992] Nie M , Xie Y , Loo JA , Courey AJ . Genetic and proteomic evidence for roles of Drosophila SUMO in cell cycle control, Ras signaling, and early pattern formation. PloS One. 2009;4(6):e5905. 19529778 10.1371/journal.pone.0005905 PMC2692000

[993] Pauws E , Stanier P . Sumoylation in craniofacial disorders. Adv Exp Med Biol. 2017;963:323‐335. 28197921 10.1007/978-3-319-50044-7_19

[994] Evdokimov E , Sharma P , Lockett SJ , Lualdi M , Kuehn MR . Loss of SUMO1 in mice affects RanGAP1 localization and formation of PML nuclear bodies, but is not lethal as it can be compensated by SUMO2 or SUMO3. J Cell Sci. 2008;121(Pt 24):4106‐4113. 19033381 10.1242/jcs.038570

[995] Zhang FP , Mikkonen L , Toppari J , Palvimo JJ , Thesleff I , Jänne OA . Sumo‐1 function is dispensable in normal mouse development. Mol Cell Biol. 2008;28(17):5381‐5390. 18573887 10.1128/MCB.00651-08 PMC2519746

[996] Mendler L , Braun T , Müller S . The ubiquitin‐like SUMO system and heart function: from development to disease. Circ Res. 2016;118(1):132‐144. 26837744 10.1161/CIRCRESAHA.115.307730

[997] Zhu X , Ding S , Qiu C , et al. SUMOylation negatively regulates angiogenesis by targeting endothelial NOTCH signaling. Circ Res. 2017;121(6):636‐649. 28760777 10.1161/CIRCRESAHA.117.310696 PMC5581236

[998] Rodriguez A , Pangas SA . Regulation of germ cell function by SUMOylation. Cell Tissue Res. 2016;363(1):47‐55. 26374733 10.1007/s00441-015-2286-5 PMC4703547

[999] Chen Y‐D , Liu J‐Y , Lu Y‐M , et al. Functional roles of C/EBPα and SUMO‑modification in lung development. Int J Mol Med. 2017;40(4):1037‐1046. 28902364 10.3892/ijmm.2017.3111 PMC5593452

[1000] García‐Gutiérrez P , García‐Domínguez M . SUMO control of nervous system development. Semin Cell Dev Biol. 2022;132:203‐212. 34848148 10.1016/j.semcdb.2021.11.022

[1001] Wang J , Chen L , Wen S , et al. Defective sumoylation pathway directs congenital heart disease. Birth Defects Res A Clin Mol Teratol. 2011;91(6):468‐476. 21563299 10.1002/bdra.20816 PMC5031480

[1002] Kim EY , Chen L , Ma Y , et al. Enhanced desumoylation in murine hearts by overexpressed SENP2 leads to congenital heart defects and cardiac dysfunction. J Mol Cell Cardiol. 2012;52(3):638‐649. 22155005 10.1016/j.yjmcc.2011.11.011 PMC3294171

[1003] Zhu X , Qiu C , Wang Y , et al. FGFR1 SUMOylation coordinates endothelial angiogenic signaling in angiogenesis. Proc Natl Acad Sci U S A. 2022;119(26):e2202631119. 35733256 10.1073/pnas.2202631119 PMC9245619

[1004] La Salle S, Sun F , Zhang XD , Matunis MJ , Handel MA . Developmental control of sumoylation pathway proteins in mouse male germ cells. Dev Biol. 2008;321(1):227‐237. 18602382 10.1016/j.ydbio.2008.06.020 PMC2599952

[1005] Chen YD , Liu JY , Lu YM , et al. Functional roles of C/EBPα and SUMO‑modification in lung development. Int J Mol Med. 2017;40(4):1037‐1046. 28902364 10.3892/ijmm.2017.3111 PMC5593452

[1006] Correa‐Vázquez JF , Juárez‐Vicente F , García‐Gutiérrez P , Barysch SV , Melchior F , García‐Domínguez M . The Sumo proteome of proliferating and neuronal‐differentiating cells reveals Utf1 among key Sumo targets involved in neurogenesis. Cell Death Dis. 2021;12(4):305. 33753728 10.1038/s41419-021-03590-2 PMC7985304

[1007] Chen X , Qin Y , Zhang Y , et al. SENP2‐PLCβ4 signaling regulates neurogenesis through the maintenance of calcium homeostasis. Cell Death Differ. 2022;29(2):337‐350. 34465891 10.1038/s41418-021-00857-1 PMC8817034

[1008] Mojsa B , Tatham MH , Davidson L , Liczmanska M , Branigan E , Hay RT . Identification of SUMO targets associated with the pluripotent state in human stem cells. Mol Cell Proteomics. 2021;20:100164. 34673284 10.1016/j.mcpro.2021.100164 PMC8604812

[1009] Chymkowitch P , Nguea PA , Enserink JM . SUMO‐regulated transcription: challenging the dogma. Bioessays. 2015;37(10):1095‐1105. 26354225 10.1002/bies.201500065

[1010] Stankova T , Piepkorn L , Bayer TA , Jahn O , Tirard M . SUMO1‐conjugation is altered during normal aging but not by increased amyloid burden. Aging Cell. 2018;17(4):e12760. 29633471 10.1111/acel.12760 PMC6052395

[1011] Moll L , Roitenberg N , Bejerano‐Sagie M , et al. The insulin/IGF signaling cascade modulates SUMOylation to regulate aging and proteostasis in Caenorhabditis elegans. Elife. 2018;7:e38635. 30403374 10.7554/eLife.38635 PMC6277199

[1012] McManus FP , Bourdeau V , Acevedo M , et al. Quantitative SUMO proteomics reveals the modulation of several PML nuclear body associated proteins and an anti‐senescence function of UBC9. Sci Rep. 2018;8(1):7754. 29773808 10.1038/s41598-018-25150-z PMC5958138

[1013] Swift ML , Sell C , Azizkhan‐Clifford J . DNA damage‐induced degradation of Sp1 promotes cellular senescence. Geroscience. 2022;44(2):683‐698. 34550526 10.1007/s11357-021-00456-5 PMC9135943

[1014] Chhunchha B , Kubo E , Singh P , Singh DP . SUMOylation‐deficient Prdx6 repairs aberrant SUMOylation‐mediated Sp1 dysregulation‐dependent Prdx6 repression and cell injury in aging and oxidative stress. Aging. 2018;10(9):2284‐2315. 30215601 10.18632/aging.101547 PMC6188488

[1015] Shyu YC , Lee TL , Chen X , et al. Tight regulation of a timed nuclear import wave of EKLF by PKCtheta and FOE during Pro‐E to Baso‐E transition. Dev Cell. 2014;28(4):409‐422. 24576425 10.1016/j.devcel.2014.01.007

[1016] Shyu YC , Liao PC , Huang TS , et al. Genetic disruption of KLF1 K74 SUMOylation in hematopoietic system promotes healthy longevity in mice. Adv Sci (Weinh). 2022;9(25):e2201409. 35822667 10.1002/advs.202201409 PMC9443461

[1017] Gao K , Li Y , Hu S , Liu Y . SUMO peptidase ULP‐4 regulates mitochondrial UPR‐mediated innate immunity and lifespan extension. Elife. 2019;8:e41792. 30642431 10.7554/eLife.41792 PMC6355198

[1018] Talamillo A , Ajuria L , Grillo M , Barroso‐Gomila O , Mayor U , Barrio R . SUMOylation in the control of cholesterol homeostasis. Open Biol. 2020;10(5):200054. 32370667 10.1098/rsob.200054 PMC7276529

[1019] Mziaut H , Trajkovski M , Kersting S , et al. Synergy of glucose and growth hormone signalling in islet cells through ICA512 and STAT5. Nat Cell Biol. 2006;8(5):435‐445. 16622421 10.1038/ncb1395

[1020] Hu A , Zou H , Chen B , Zhong J . Posttranslational modifications in diabetes: mechanisms and functions. Rev Endocr Metab Disord. 2022;23(5):1011‐1033. 35697961 10.1007/s11154-022-09740-x

[1021] Shishido T , Woo C‐H , Ding B , et al. Effects of MEK5/ERK5 association on small ubiquitin‐related modification of ERK5: implications for diabetic ventricular dysfunction after myocardial infarction. Circ Res. 2008;102(11):1416‐1425. 18467627 10.1161/CIRCRESAHA.107.168138 PMC2614366

[1022] Guo D , Li M , Zhang Y , et al. A functional variant of SUMO4, a new I kappa B alpha modifier, is associated with type 1 diabetes. Nat Genet. 2004;36(8):837‐841. 15247916 10.1038/ng1391

[1023] Rabellino A , Melegari M , Tompkins VS , et al. PIAS1 Promotes Lymphomagenesis through MYC Upregulation. Cell Rep. 2016;15(10):2266‐2278. 27239040 10.1016/j.celrep.2016.05.015 PMC4899214

[1024] Puhr M , Hoefer J , Neuwirt H , et al. PIAS1 is a crucial factor for prostate cancer cell survival and a valid target in docetaxel resistant cells. Oncotarget. 2014;5(23):12043‐12056. 25474038 10.18632/oncotarget.2658 PMC4322998

[1025] Li H , Gao H , Bijukchhe SM , Wang Y , Li T . PIAS3 may represent a potential biomarker for diagnosis and therapeutic of human colorectal cancer. Med Hypotheses. 2013;81(6):1151‐1154. 24120699 10.1016/j.mehy.2013.09.022

[1026] Galanty Y , Belotserkovskaya R , Coates J , Jackson SP . RNF4, a SUMO‐targeted ubiquitin E3 ligase, promotes DNA double‐strand break repair. Genes Dev. 2012;26(11):1179‐1195. 22661229 10.1101/gad.188284.112 PMC3371407

[1027] Xia L , Jiang Y , Zhang X‐H , et al. SUMOylation disassembles the tetrameric pyruvate kinase M2 to block myeloid differentiation of leukemia cells. Cell Death Dis. 2021;12(1):101. 33473116 10.1038/s41419-021-03400-9 PMC7817830

[1028] Shangguan X , He J , Ma Z , et al. SUMOylation controls the binding of hexokinase 2 to mitochondria and protects against prostate cancer tumorigenesis. Nat Commun. 2021;12(1):1812. 33753739 10.1038/s41467-021-22163-7 PMC7985146

[1029] Xiao M , Bian Q , Lao Y , et al. SENP3 loss promotes M2 macrophage polarization and breast cancer progression. Mol Oncol. 2022;16(4):1026‐1044. 33932085 10.1002/1878-0261.12967 PMC8847990

[1030] Hu Z , Teng X‐L , Zhang T , et al. SENP3 senses oxidative stress to facilitate STING‐dependent dendritic cell antitumor function. Mol Cell. 2021;81(5):940‐952. 33434504 10.1016/j.molcel.2020.12.024

[1031] Wu Z , Huang H , Han Q , et al. SENP7 senses oxidative stress to sustain metabolic fitness and antitumor functions of CD8+ T cells. J Clin Invest. 2022;132(7):e155224. 35143421 10.1172/JCI155224 PMC8970670

[1032] Biederstädt A , Hassan Z , Schneeweis C , et al. SUMO pathway inhibition targets an aggressive pancreatic cancer subtype. Gut. 2020;69(8):1472‐1482. 32001555 10.1136/gutjnl-2018-317856 PMC7398468

[1033] Rohrberg J , Van de Mark D , Amouzgar M , et al. MYC dysregulates mitosis, revealing cancer vulnerabilities. Cell Rep. 2020;30(10):3368‐3382. 32160543 10.1016/j.celrep.2020.02.041 PMC7085414

[1034] Schneeweis C , Hassan Z , Schick M , Keller U , Schneider G . The SUMO pathway in pancreatic cancer: insights and inhibition. Br J Cancer. 2021;124(3):531‐538. 33071285 10.1038/s41416-020-01119-6 PMC7851129

[1035] Du L , Liu W , Rosen ST . Targeting SUMOylation in cancer. Curr Opin Oncol. 2021;33(5):520‐525. 34280172 10.1097/CCO.0000000000000765

[1036] Sanyal S , Mondal P , Sen S , Sengupta Bandyopadhyay S , Das C . SUMO E3 ligase CBX4 regulates hTERT‐mediated transcription of CDH1 and promotes breast cancer cell migration and invasion. Biochem J. 2020;477(19):3803‐3818. 32926159 10.1042/BCJ20200359

[1037] Xu H , Wang H , Zhao W , et al. SUMO1 modification of methyltransferase‐like 3 promotes tumor progression via regulating Snail mRNA homeostasis in hepatocellular carcinoma. Theranostics. 2020;10(13):5671‐5686. 32483411 10.7150/thno.42539 PMC7254988

[1038] Krumova P , Weishaupt JH . Sumoylation in neurodegenerative diseases. Cell Mol Life Sci. 2013;70(12):2123‐2138. 23007842 10.1007/s00018-012-1158-3 PMC11113377

[1039] Henley JM , Craig TJ , Wilkinson KA . Neuronal SUMOylation: mechanisms, physiology, and roles in neuronal dysfunction. Physiol Rev. 2014;94(4):1249‐1285. 25287864 10.1152/physrev.00008.2014 PMC4187031

[1040] Chenfei Z , Haizhen Y , Jie X , Na Z , Bo X . Effects of aerobic exercise on hippocampal SUMOylation in APP/PS1 transgenic mice. Neurosci Lett. 2022;767:136303. 34695453 10.1016/j.neulet.2021.136303

[1041] Nistico R , Ferraina C , Marconi V , et al. Age‐related changes of protein SUMOylation balance in the AbetaPP Tg2576 mouse model of Alzheimer's disease. Front Pharmacol. 2014;5:63. 24778618 10.3389/fphar.2014.00063 PMC3985012

[1042] Princz A , Tavernarakis N . SUMOylation in neurodegenerative diseases. Gerontology. 2020;66(2):122‐130. 31505513 10.1159/000502142

[1043] Liu Y‐C , Hsu W‐L , Ma Y‐L , Lee EHY . Melatonin induction of APP intracellular domain 50 SUMOylation alleviates AD through enhanced transcriptional activation and Aβ degradation. Mol Ther. 2021;29(1):376‐395. 32950104 10.1016/j.ymthe.2020.09.003 PMC7791018

[1044] Dorval V , Fraser PE . Small ubiquitin‐like modifier (SUMO) modification of natively unfolded proteins tau and alpha‐synuclein. J Biol Chem. 2006;281(15):9919‐9924. 16464864 10.1074/jbc.M510127200

[1045] Savyon M , Engelender S . SUMOylation in α‐Synuclein homeostasis and pathology. Front Aging Neurosci. 2020;12:167. 32670048 10.3389/fnagi.2020.00167 PMC7330056

[1046] Yau TY , Molina O , Courey AJ . SUMOylation in development and neurodegeneration. Development. 2020;147(6):dev175703. 32188601 10.1242/dev.175703 PMC7097199

[1047] Marmor‐Kollet H , Siany A , Kedersha N , et al. Spatiotemporal proteomic analysis of stress granule disassembly using APEX reveals regulation by SUMOylation and links to ALS pathogenesis. Mol Cell. 2020;80(5):876‐891. 33217318 10.1016/j.molcel.2020.10.032 PMC7816607

[1048] Wada H , Suzuki D , Niikura T . Regulation of ALS‐associated SOD1 mutant SUMOylation and aggregation by SENP and PIAS family proteins. J Mol Neurosci. 2020;70(12):2007‐2014. 32462635 10.1007/s12031-020-01604-w

[1049] Maurel C , Chami AA , Thépault R‐A , et al. A role for SUMOylation in the formation and cellular localization of TDP‐43 aggregates in amyotrophic lateral sclerosis. Mol Neurobiol. 2020;57(3):1361‐1373. 31728929 10.1007/s12035-019-01810-7

[1050] Li W , Chopp M , Zacharek A , et al. SUMO1 deficiency exacerbates neurological and cardiac dysfunction after intracerebral hemorrhage in aged mice. Transl Stroke Res. 2021;12(4):631‐642. 32761461 10.1007/s12975-020-00837-6

[1051] Du C , Chen X , Su Q , et al. The function of SUMOylation and its critical roles in cardiovascular diseases and potential clinical implications. Int J Mol Sci. 2021;22(19):10618. 34638970 10.3390/ijms221910618 PMC8509021

[1052] Chang E , Abe JI . Kinase‐SUMO networks in diabetes‐mediated cardiovascular disease. Metabolism. 2016;65(5):623‐633. 27085771 10.1016/j.metabol.2016.01.007 PMC5226250

[1053] Desterro JM , Rodriguez MS , Hay RT . SUMO‐1 modification of IkappaBalpha inhibits NF‐kappaB activation. Mol Cell. 1998;2(2):233‐239. 9734360 10.1016/s1097-2765(00)80133-1

[1054] Kho C , Lee A , Jeong D , et al. Small‐molecule activation of SERCA2a SUMOylation for the treatment of heart failure. Nat Commun. 2015;6:7229. 26068603 10.1038/ncomms8229 PMC4467461

[1055] Huang C , Han Y , Wang Y , et al. SENP3 is responsible for HIF‐1 transactivation under mild oxidative stress via p300 de‐SUMOylation. EMBO J. 2009;28(18):2748‐2762. 19680224 10.1038/emboj.2009.210 PMC2750016

[1056] Gu J , Fan Y , Liu X , et al. SENP1 protects against myocardial ischaemia/reperfusion injury via a HIF1α‐dependent pathway. Cardiovasc Res. 2014;104(1):83‐92. 25082844 10.1093/cvr/cvu177

[1057] Qin Y , Li Q , Liang W , et al. TRIM28 SUMOylates and stabilizes NLRP3 to facilitate inflammasome activation. Nat Commun. 2021;12(1):4794. 34373456 10.1038/s41467-021-25033-4 PMC8352945

[1058] Alquezar C , Arya S , Kao AW . Tau post‐translational modifications: dynamic transformers of Tau function, degradation, and aggregation. Front Neurol. 2020;11:595532. 33488497 10.3389/fneur.2020.595532 PMC7817643

[1059] Soares ES , Prediger RD , Brocardo PS , Cimarosti HI . SUMO‐modifying Huntington's disease. IBRO Neurosci Rep. 2022;12:203‐209. 35746980 10.1016/j.ibneur.2022.03.002 PMC9210482

[1060] Ma R , Ma L , Weng W , et al. DUSP6 SUMOylation protects cells from oxidative damage via direct regulation of Drp1 dephosphorylation. Sci Adv. 2020;6(13):eaaz0361. 32232156 10.1126/sciadv.aaz0361 PMC7096176

[1061] Xie B , Liu X , Yang J , Cheng J , Gu J , Xue S . PIAS1 protects against myocardial ischemia‐reperfusion injury by stimulating PPARγ SUMOylation. BMC Cell Biol. 2018;19(1):24. 30419807 10.1186/s12860-018-0176-x PMC6233564

[1062] Du Y , Liu P , Xu T , et al. Luteolin modulates SERCA2a leading to attenuation of myocardial ischemia/reperfusion injury via Sumoylation at lysine 585 in mice. Cell Physiol Biochem. 2018;45(3):883‐898. 29421780 10.1159/000487283

[1063] Fan L , Yang X , Zheng M , et al. Regulation of SUMOylation targets associated with Wnt/β‐Catenin pathway. Front Oncol. 2022;12:943683. 35847921 10.3389/fonc.2022.943683 PMC9280480

[1064] Wu R , Cui Y , Yuan X , et al. SUMO‐specific protease 1 modulates cadmium‐augmented transcriptional activity of androgen receptor (AR) by reversing AR SUMOylation. Toxicol Lett. 2014;229(2):405‐413. 25014244 10.1016/j.toxlet.2014.07.003

[1065] Vitaliano‐Prunier A , Halftermeyer J , Ablain J , et al. Clearance of PML/RARA‐bound promoters suffice to initiate APL differentiation. Blood. 2014;124(25):3772‐3780. 25258343 10.1182/blood-2014-03-561852

[1066] Xie H , Wang Y‐H , Liu X , et al. SUMOylation of ERp44 enhances Ero1α ER retention contributing to the pathogenesis of obesity and insulin resistance. Metabolism. 2022:155351. 36427672 10.1016/j.metabol.2022.155351

[1067] Benoit YD , Mitchell RR , Wang W , et al. Targeting SUMOylation dependency in human cancer stem cells through a unique SAE2 motif revealed by chemical genomics. Cell Chem Biol. 2021;28(10):1394‐1406. e10. 33979648 10.1016/j.chembiol.2021.04.014 PMC8542640

[1068] Bellail AC , Jin HR , Lo H‐Y , et al. Ubiquitination and degradation of SUMO1 by small‐molecule degraders extends survival of mice with patient‐derived tumors. Sci Transl Med. 2021;13(615):eabh1486. 34644148 10.1126/scitranslmed.abh1486 PMC9450956

[1069] Lightcap ES , Yu P , Grossman S , et al. A small‐molecule SUMOylation inhibitor activates antitumor immune responses and potentiates immune therapies in preclinical models. Sci Transl Med. 2021;13(611):eaba7791. 34524860 10.1126/scitranslmed.aba7791 PMC9719791

[1070] Cox OF , Huber PW . Developing practical therapeutic strategies that target protein SUMOylation. Curr Drug Targets. 2019;20(9):960‐969. 30362419 10.2174/1389450119666181026151802 PMC6700758

[1071] Steentoft C , Vakhrushev SY , Joshi HJ , et al. Precision mapping of the human O‐GalNAc glycoproteome through SimpleCell technology. EMBO J. 2013;32(10):1478‐1488. 23584533 10.1038/emboj.2013.79 PMC3655468

[1072] Varki A . Biological roles of glycans. Glycobiology. 2017;27(1):3‐49. 27558841 10.1093/glycob/cww086 PMC5884436

[1073] Zielinska DF , Gnad F , Wiśniewski JR , Mann M . Precision mapping of an in vivo N‐glycoproteome reveals rigid topological and sequence constraints. Cell. 2010;141(5):897‐907. 20510933 10.1016/j.cell.2010.04.012

[1074] Xu S , Tong M , Suttapitugsakul S , Wu R . Spatial and temporal proteomics reveals the distinct distributions and dynamics of O‐GlcNAcylated proteins. Cell Rep. 2022;39(11):110946. 35705054 10.1016/j.celrep.2022.110946 PMC9244862

[1075] Zhu Q , Zhou H , Wu L , et al. O‐GlcNAcylation promotes pancreatic tumor growth by regulating malate dehydrogenase 1. Nat Chem Biol. 2022;18(10):1087‐1095. 35879546 10.1038/s41589-022-01085-5

[1076] Schjoldager KT , Narimatsu Y , Joshi HJ , Clausen H . Global view of human protein glycosylation pathways and functions. Nat Rev Mol Cell Biol. 2020;21(12):729‐749. 33087899 10.1038/s41580-020-00294-x

[1077] Moremen KW , Tiemeyer M , Nairn AV . Vertebrate protein glycosylation: diversity, synthesis and function. Nat Rev Mol Cell Biol. 2012;13(7):448‐462. 22722607 10.1038/nrm3383 PMC3934011

[1078] Joshi HJ , Hansen L , Narimatsu Y , et al. Glycosyltransferase genes that cause monogenic congenital disorders of glycosylation are distinct from glycosyltransferase genes associated with complex diseases. Glycobiology. 2018;28(5):284‐294. 29579191 10.1093/glycob/cwy015 PMC6279177

[1079] Hansen L , Husein DM , Gericke B , et al. A mutation map for human glycoside hydrolase genes. Glycobiology. 2020;30(8):500‐515. 32039448 10.1093/glycob/cwaa010 PMC7372926

[1080] Cummings RD . The repertoire of glycan determinants in the human glycome. Mol Biosyst. 2009;5(10):1087‐1104. 19756298 10.1039/b907931a

[1081] Laine RA . A calculation of all possible oligosaccharide isomers both branched and linear yields 1.05×10(12) structures for a reducing hexasaccharide: the Isomer Barrier to development of single‐method saccharide sequencing or synthesis systems. Glycobiology. 1994;4(6):759‐767. 7734838 10.1093/glycob/4.6.759

[1082] Reily C , Stewart TJ , Renfrow MB , Novak J . Glycosylation in health and disease. Nat Rev Nephrol. 2019;15(6):346‐366. 30858582 10.1038/s41581-019-0129-4 PMC6590709

[1083] Cao L , Diedrich JK , Ma Y , et al. Global site‐specific analysis of glycoprotein N‐glycan processing. Nat Protoc. 2018;13(6):1196‐1212. 29725121 10.1038/nprot.2018.024 PMC5941933

[1084] Ruiz‐Canada C , Kelleher DJ , Gilmore R . Cotranslational and posttranslational N‐glycosylation of polypeptides by distinct mammalian OST isoforms. Cell. 2009;136(2):272‐283. 19167329 10.1016/j.cell.2008.11.047 PMC2859625

[1085] Cherepanova NA , Gilmore R . Mammalian cells lacking either the cotranslational or posttranslocational oligosaccharyltransferase complex display substrate‐dependent defects in asparagine linked glycosylation. Sci Rep. 2016;6:20946. 26864433 10.1038/srep20946 PMC4750078

[1086] Lu H , Fermaintt CS , Cherepanova NA , Gilmore R , Yan N , Lehrman MA . Mammalian STT3A/B oligosaccharyltransferases segregate N‐glycosylation at the translocon from lipid‐linked oligosaccharide hydrolysis. Proc Natl Acad Sci U S A. 2018;115(38):9557‐9562. 30181269 10.1073/pnas.1806034115 PMC6156661

[1087] Hounsell EF , Davies MJ , Renouf DV . O‐linked protein glycosylation structure and function. Glycoconj J. 1996;13(1):19‐26. 8785483 10.1007/BF01049675

[1088] Zauner G , Kozak RP , Gardner RA , Fernandes DL , Deelder AM , Wuhrer M . Protein O‐glycosylation analysis. Biol Chem. 2012;393(8):687‐708. 22944673 10.1515/hsz-2012-0144

[1089] Magalhães A , Duarte HO , Reis CA . The role of O‐glycosylation in human disease. Mol Aspects Med. 2021;79:100964. 33775405 10.1016/j.mam.2021.100964

[1090] Nielsen MI , de Haan N , Kightlinger W , et al. Global mapping of GalNAc‐T isoform‐specificities and O‐glycosylation site‐occupancy in a tissue‐forming human cell line. Nat Commun. 2022;13(1):6257. 36270990 10.1038/s41467-022-33806-8 PMC9587226

[1091] Bennett EP , Mandel U , Clausen H , Gerken TA , Fritz TA , Tabak LA . Control of mucin‐type O‐glycosylation: a classification of the polypeptide GalNAc‐transferase gene family. Glycobiology. 2012;22(6):736‐756. 22183981 10.1093/glycob/cwr182 PMC3409716

[1092] Shan A , Lu J , Xu Z , et al. Polypeptide N‐acetylgalactosaminyltransferase 18 non‐catalytically regulates the ER homeostasis and O‐glycosylation. Biochim Biophys Acta Gen Subj. 2019;1863(5):870‐882. 30797803 10.1016/j.bbagen.2019.01.009

[1093] de Las Rivas M , Lira‐Navarrete E , Gerken TA , Hurtado‐Guerrero R . Polypeptide GalNAc‐Ts: from redundancy to specificity. Curr Opin Struct Biol. 2019;56:87‐96. 30703750 10.1016/j.sbi.2018.12.007 PMC6656595

[1094] Gill DJ , Clausen H , Bard F . Location, location, location: new insights into O‐GalNAc protein glycosylation. Trends Cell Biol. 2011;21(3):149‐158. 21145746 10.1016/j.tcb.2010.11.004

[1095] Bagdonaite I , Pallesen EMH , Nielsen MI , Bennett EP , Wandall HH . Mucin‐type O‐GalNAc glycosylation in health and disease. Adv Exp Med Biol. 2021;1325:25‐60. 34495529 10.1007/978-3-030-70115-4_2

[1096] Harvey BM , Haltiwanger RS . Regulation of Notch function by O‐glycosylation. Adv Exp Med Biol. 2018;1066:59‐78. 30030822 10.1007/978-3-319-89512-3_4

[1097] Lo PW , Shie JJ , Chen CH , Wu CY , Hsu TL , Wong CH . O‐GlcNAcylation regulates the stability and enzymatic activity of the histone methyltransferase EZH2. Proc Natl Acad Sci U S A. 2018;115(28):7302‐7307. 29941599 10.1073/pnas.1801850115 PMC6048490

[1098] Varshney S , Stanley P . Multiple roles for O‐glycans in Notch signalling. FEBS Lett. 2018;592(23):3819‐3834. 30207383 10.1002/1873-3468.13251 PMC6289669

[1099] Vosseller K , Wells L , Hart GW . Nucleocytoplasmic O‐glycosylation: O‐GlcNAc and functional proteomics. Biochimie. 2001;83(7):575‐781. 11522385 10.1016/s0300-9084(01)01295-0

[1100] Rexach JE , Clark PM , Hsieh‐Wilson LC . Chemical approaches to understanding O‐GlcNAc glycosylation in the brain. Nat Chem Biol. 2008;4(2):97‐106. 18202679 10.1038/nchembio.68 PMC3250351

[1101] Chatham JC , Zhang J , Wende AR . Role of ‐linked ‐acetylglucosamine protein modification in cellular (patho)physiology. Physiol Rev. 2021;101(2):427‐493. 32730113 10.1152/physrev.00043.2019 PMC8428922

[1102] Hegyi B , Fasoli A , Ko CY , et al. CaMKII serine 280 O‐GlcNAcylation links diabetic hyperglycemia to proarrhythmia. Circ Res. 2021;129(1):98‐113. 33926209 10.1161/CIRCRESAHA.120.318402 PMC8221539

[1103] Li Z , Xu J , Song Y , et al. PRMT5 prevents dilated cardiomyopathy via suppression of protein O‐GlcNAcylation. Circ Res. 2021;129(9):857‐871. 34503365 10.1161/CIRCRESAHA.121.319456

[1104] Crine SL , Acharya KR . Molecular basis of C‐mannosylation ‐ a structural perspective. FEBS J. 2021; 10.1111/febs.16265 34741587

[1105] Shcherbakova A , Tiemann B , Buettner FF , Bakker H . Distinct C‐mannosylation of netrin receptor thrombospondin type 1 repeats by mammalian DPY19L1 and DPY19L3. Proc Natl Acad Sci U S A. 2017;114(10):2574‐2579. 28202721 10.1073/pnas.1613165114 PMC5347603

[1106] Shcherbakova A , Preller M , Taft MH , et al. C‐mannosylation supports folding and enhances stability of thrombospondin repeats. Elife. 2019;8:e52978. 31868591 10.7554/eLife.52978 PMC6954052

[1107] Minakata S , Manabe S , Inai Y , et al. Protein C‐mannosylation and C‐mannosyl tryptophan in chemical biology and medicine. Molecules. 2021;26(17):5258. 34500691 10.3390/molecules26175258 PMC8433626

[1108] Niwa Y , Simizu S , C‐mannosylation: previous studies and future research perspectives. Trends Glycosci Glyc. 2018;30(177):E231‐E238.

[1109] Lovelace LL , Cooper CL , Sodetz JM , Lebioda L . Structure of human C8 protein provides mechanistic insight into membrane pore formation by complement. J Biol Chem. 2011;286(20):17585‐17592. 21454577 10.1074/jbc.M111.219766 PMC3093833

[1110] Thomas JR , Dwek RA , Rademacher TW . Structure, biosynthesis, and function of glycosylphosphatidylinositols. Biochemistry. 1990;29(23):5413‐5422. 2143679 10.1021/bi00475a001

[1111] Vainauskas S , Menon AK . Ethanolamine phosphate linked to the first mannose residue of glycosylphosphatidylinositol (GPI) lipids is a major feature of the GPI structure that is recognized by human GPI transamidase. J Biol Chem. 2006;281(50):38358‐38364. 17060324 10.1074/jbc.M608896200

[1112] Menon AK , Eppinger M , Mayor S , Schwarz RT . Phosphatidylethanolamine is the donor of the terminal phosphoethanolamine group in trypanosome glycosylphosphatidylinositols. EMBO J. 1993;12(5):1907‐1914. 8491183 10.1002/j.1460-2075.1993.tb05839.x PMC413411

[1113] Menon AK , Stevens VL . Phosphatidylethanolamine is the donor of the ethanolamine residue linking a glycosylphosphatidylinositol anchor to protein. J Biol Chem. 1992;267(22):15277‐15280. 1322394

[1114] Lehto MT , Sharom FJ . PI‐specific phospholipase C cleavage of a reconstituted GPI‐anchored protein: modulation by the lipid bilayer. Biochemistry. 2002;41(4):1398‐1408. 11802743 10.1021/bi011579w

[1115] Müller A , Klöppel C , Smith‐Valentine M , Van Houten J , Simon M . Selective and programmed cleavage of GPI‐anchored proteins from the surface membrane by phospholipase C. Biochim Biophys Acta. 2012;1818(1):117‐124. 22024023 10.1016/j.bbamem.2011.10.009

[1116] Liu SS , Liu YS , Guo XY , et al. A knockout cell library of GPI biosynthetic genes for functional studies of GPI‐anchored proteins. Commun Biol. 2021;4(1):777. 34162996 10.1038/s42003-021-02337-1 PMC8222316

[1117] Du L , Sohr A , Li Y , Roy S . GPI‐anchored FGF directs cytoneme‐mediated bidirectional contacts to regulate its tissue‐specific dispersion. Nat Commun. 2022;13(1):3482. 35710780 10.1038/s41467-022-30417-1 PMC9203819

[1118] Zhou Q , Qiu H . The mechanistic impact of N‐glycosylation on stability, pharmacokinetics, and immunogenicity of therapeutic proteins. J Pharm Sci. 2019;108(4):1366‐1377. 30471292 10.1016/j.xphs.2018.11.029

[1119] Roth J , Zuber C , Park S , et al. Protein N‐glycosylation, protein folding, and protein quality control. Mol Cells. 2010;30(6):497‐506. 21340671 10.1007/s10059-010-0159-z

[1120] Green RS , Stone EL , Tenno M , Lehtonen E , Farquhar MG , Marth JD . Mammalian N‐glycan branching protects against innate immune self‐recognition and inflammation in autoimmune disease pathogenesis. Immunity. 2007;27(2):308‐320. 17681821 10.1016/j.immuni.2007.06.008

[1121] Gu J , Isaji T , Xu Q , et al. Potential roles of N‐glycosylation in cell adhesion. Glycoconj J. 2012;29(8‐9):599‐607. 22565826 10.1007/s10719-012-9386-1

[1122] He X , Xu C . Immune checkpoint signaling and cancer immunotherapy. Cell Res. 2020;30(8):660‐669. 32467592 10.1038/s41422-020-0343-4 PMC7395714

[1123] Huang X , Ye Q , Chen M , et al. N‐glycosylation‐defective splice variants of neuropilin‐1 promote metastasis by activating endosomal signals. Nat Commun. 2019;10(1):3708. 31420553 10.1038/s41467-019-11580-4 PMC6697747

[1124] Esmail S , Manolson MF . Advances in understanding N‐glycosylation structure, function, and regulation in health and disease. Eur J Cell Biol. 2021;100(7‐8):151186. 34839178 10.1016/j.ejcb.2021.151186

[1125] Zhang A , Tsukamoto Y , Takeuchi H , Nishiwaki K , Tashima Y , Okajima T . Secretory expression of mammalian NOTCH tandem epidermal growth factor‐like repeats based on increased O‐glycosylation. Anal Biochem. 2022;656:114881. 36067866 10.1016/j.ab.2022.114881

[1126] Madsen TD , Hansen LH , Hintze J , et al. An atlas of O‐linked glycosylation on peptide hormones reveals diverse biological roles. Nat Commun. 2020;11(1):4033. 32820167 10.1038/s41467-020-17473-1 PMC7441158

[1127] Zhao P , Praissman JL , Grant OC , et al. Virus‐receptor interactions of glycosylated SARS‐CoV‐2 spike and human ACE2 receptor. Cell Host Microbe. 2020;28(4):586‐601. e6. 32841605 10.1016/j.chom.2020.08.004 PMC7443692

[1128] McGuckin MA , Lindén SK , Sutton P , Florin TH . Mucin dynamics and enteric pathogens. Nat Rev Microbiol. 2011;9(4):265‐278. 21407243 10.1038/nrmicro2538

[1129] Johannes L , Billet A . Glycosylation and raft endocytosis in cancer. Cancer Metastasis Rev. 2020;39(2):375‐396. 32388640 10.1007/s10555-020-09880-z PMC7311491

[1130] Wandall HH , Nielsen MAI , King‐Smith S , de Haan N , Bagdonaite I . Global functions of O‐glycosylation: promises and challenges in O‐glycobiology. FEBS J. 2021;288(24):7183‐7212. 34346177 10.1111/febs.16148

[1131] Tian E , Wang S , Zhang L , et al. Galnt11 regulates kidney function by glycosylating the endocytosis receptor megalin to modulate ligand binding. Proc Natl Acad Sci U S A. 2019;116(50):25196‐25202. 31740596 10.1073/pnas.1909573116 PMC6911204

[1132] Varshney S , Stanley P . EOGT and O‐GlcNAc on secreted and membrane proteins. Biochem Soc Trans. 2017;45(2):401‐408. 28408480 10.1042/BST20160165 PMC8837192

[1133] Ihara Y , Inai Y , Ikezaki M , Matsui ISL , Manabe S , Ito Y . C‐mannosylation: modification on tryptophan in cellular proteins. Glycoscience: biology and medicine. Springer Japan; 2015:1091‐1100.

[1134] Haltiwanger RS , Lowe JB . Role of glycosylation in development. Annu Rev Biochem. 2004;73:491‐537. 15189151 10.1146/annurev.biochem.73.011303.074043

[1135] Wang J , Miao Y , Wicklein R , et al. RTN4/NoGo‐receptor binding to BAI adhesion‐GPCRs regulates neuronal development. Cell. 2021;184(24):5869‐5885. 34758294 10.1016/j.cell.2021.10.016 PMC8620742

[1136] Hansen L , Lind‐Thomsen A , Joshi HJ , et al. A glycogene mutation map for discovery of diseases of glycosylation. Glycobiology. 2015;25(2):211‐224. 25267602 10.1093/glycob/cwu104 PMC4351397

[1137] Al Teneiji A, Bruun TU , Sidky S , et al. Phenotypic and genotypic spectrum of congenital disorders of glycosylation type I and type II. Mol Genet Metab. 2017;120(3):235‐242. 28122681 10.1016/j.ymgme.2016.12.014

[1138] Hennet T , Cabalzar J . Congenital disorders of glycosylation: a concise chart of glycocalyx dysfunction. Trends Biochem Sci. 2015;40(7):377‐384. 25840516 10.1016/j.tibs.2015.03.002

[1139] Kasper DM , Hintzen J , Wu Y , et al. The N‐glycome regulates the endothelial‐to‐hematopoietic transition. Science. 2020;370(6521):1186‐1191. 33273096 10.1126/science.aaz2121 PMC8312266

[1140] Miura Y , Endo T . Glycomics and glycoproteomics focused on aging and age‐related diseases–glycans as a potential biomarker for physiological alterations. Biochim Biophys Acta. 2016;1860(8):1608‐1614. 26801879 10.1016/j.bbagen.2016.01.013

[1141] Dall'Olio F , Vanhooren V , Chen CC , Slagboom PE , Wuhrer M , Franceschi C . N‐glycomic biomarkers of biological aging and longevity: a link with inflammaging. Ageing Res Rev. 2013;12(2):685‐698. 22353383 10.1016/j.arr.2012.02.002

[1142] Dall'Olio F . Glycobiology of aging. Subcell Biochem. 2018;90:505‐526. 30779020 10.1007/978-981-13-2835-0_17

[1143] Knezevic A , Gornik O , Polasek O , et al. Effects of aging, body mass index, plasma lipid profiles, and smoking on human plasma N‐glycans. Glycobiology. 2010;20(8):959‐969. 20356825 10.1093/glycob/cwq051

[1144] Rocha VZ , Libby P . Obesity, inflammation, and atherosclerosis. Nat Rev Cardiol. 2009;6(6):399‐409. 19399028 10.1038/nrcardio.2009.55

[1145] Sha J , Fan J , Zhang R , et al. B‐cell‐specific ablation of β‐1,4‐galactosyltransferase 1 prevents aging‐related IgG glycans changes and improves aging phenotype in mice. J Proteomics. 2022;268:104717. 36084919 10.1016/j.jprot.2022.104717

[1146] Wang Y , Khan A , Antonopoulos A , et al. Loss of α2‐6 sialylation promotes the transformation of synovial fibroblasts into a pro‐inflammatory phenotype in arthritis. Nat Commun. 2021;12(1):2343. 33879788 10.1038/s41467-021-22365-z PMC8058094

[1147] Macauley MS , Crocker PR , Paulson JC . Siglec‐mediated regulation of immune cell function in disease. Nat Rev Immunol. 2014;14(10):653‐666. 25234143 10.1038/nri3737 PMC4191907

[1148] Bordon Y . Inflammation: live long and prosper with siglecs. Nat Rev Immunol. 2015;15(5):266‐267. 10.1038/nri3851 25882243

[1149] Zhou JY , Cobb BA . Glycans in immunologic health and disease. Annu Rev Immunol. 2021;39:511‐536. 33577348 10.1146/annurev-immunol-101819-074237

[1150] Grüneboom A , Aust O , Cibir Z , Weber F , Hermann DM , Gunzer M . Imaging innate immunity. Immunol Rev. 2022;306(1):293‐303. 34837251 10.1111/imr.13048

[1151] Kalafati L , Hatzioannou A , Hajishengallis G , Chavakis T . The role of neutrophils in trained immunity. Immunol Rev. 2022. 10.1111/imr.13142 PMC10050090 36190144

[1152] Ugonotti J , Chatterjee S , Thaysen‐Andersen M . Structural and functional diversity of neutrophil glycosylation in innate immunity and related disorders. Mol Aspects Med. 2021;79:100882. 32847678 10.1016/j.mam.2020.100882

[1153] Mayadas TN , Cullere X , Lowell CA . The multifaceted functions of neutrophils. Annu Rev Pathol. 2014;9:181‐218. 24050624 10.1146/annurev-pathol-020712-164023 PMC4277181

[1154] Chatterjee S , Lee LY , Kawahara R , et al. Protein paucimannosylation is an enriched N‐glycosylation signature of human cancers. Proteomics. 2019;19(21‐22):e1900010. 31419058 10.1002/pmic.201900010

[1155] Tjondro HC , Loke I , Chatterjee S , Thaysen‐Andersen M . Human protein paucimannosylation: cues from the eukaryotic kingdoms. Biol Rev Camb Philos Soc. 2019;94(6):2068‐2100. 31410980 10.1111/brv.12548

[1156] Johnson JL , Jones MB , Ryan SO , Cobb BA . The regulatory power of glycans and their binding partners in immunity. Trends Immunol. 2013;34(6):290‐298. 23485517 10.1016/j.it.2013.01.006 PMC3674136

[1157] Wolfert MA , Boons GJ . Adaptive immune activation: glycosylation does matter. Nat Chem Biol. 2013;9(12):776‐784. 24231619 10.1038/nchembio.1403 PMC3966069

[1158] Giovannone N , Liang J , Antonopoulos A , et al. Galectin‐9 suppresses B cell receptor signaling and is regulated by I‐branching of N‐glycans. Nat Commun. 2018;9(1):3287. 30120234 10.1038/s41467-018-05770-9 PMC6098069

[1159] Xu Z , Liu Y , He S , et al. Integrative proteomics and N‐glycoproteomics analyses of rheumatoid arthritis synovium reveal immune‐associated glycopeptides. Mol Cell Proteom. 2023; 100540. 10.1016/j.mcpro.2023.100540 PMC10176071 37019382

[1160] Hansson GC . Mucins and the Microbiome. Annu Rev Biochem. 2020;89:769‐793. 32243763 10.1146/annurev-biochem-011520-105053 PMC8442341

[1161] Kudelka MR , Stowell SR , Cummings RD , Neish AS . Intestinal epithelial glycosylation in homeostasis and gut microbiota interactions in IBD. Nat Rev Gastroenterol Hepatol. 2020;17(10):597‐617. 32710014 10.1038/s41575-020-0331-7 PMC8211394

[1162] Bergstrom K , Shan X , Casero D , et al. Proximal colon‐derived O‐glycosylated mucus encapsulates and modulates the microbiota. Science. 2020;370(6515):467‐472. 33093110 10.1126/science.aay7367 PMC8132455

[1163] Nagao‐Kitamoto H , Leslie JL , Kitamoto S , et al. Interleukin‐22‐mediated host glycosylation prevents Clostridioides difficile infection by modulating the metabolic activity of the gut microbiota. Nat Med. 2020;26(4):608‐617. 32066975 10.1038/s41591-020-0764-0 PMC7160049

[1164] Chen Z , Yu Q , Yu Q , et al. In‐depth site‐specific analysis of N‐glycoproteome in human cerebrospinal fluid and glycosylation landscape changes in Alzheimer's Disease. Mol Cell Proteomics. 2021;20:100081. 33862227 10.1016/j.mcpro.2021.100081 PMC8724636

[1165] Zhang Q , Ma C , Chin LS , Li L . Integrative glycoproteomics reveals protein N‐glycosylation aberrations and glycoproteomic network alterations in Alzheimer's disease. Sci Adv. 2020;6(40):eabc5802. 33008897 10.1126/sciadv.abc5802 PMC7852392

[1166] Frenkel‐Pinter M , Shmueli MD , Raz C , et al. Interplay between protein glycosylation pathways in Alzheimer's disease. Sci Adv. 2017;3(9):e1601576. 28929132 10.1126/sciadv.1601576 PMC5600531

[1167] Fang P , Xie J , Sang S , et al. Multilayered N‐glycoproteome profiling reveals highly heterogeneous and dysregulated protein N‐glycosylation related to Alzheimer's Disease. Anal Chem. 2020;92(1):867‐874. 31751117 10.1021/acs.analchem.9b03555

[1168] Akasaka‐Manya K , Manya H . The role of APP O‐glycosylation in Alzheimer's disease. Biomolecules. 2020;10(11):1569. 33218200 10.3390/biom10111569 PMC7699271

[1169] O'Brien RJ , Wong PC . Amyloid precursor protein processing and Alzheimer's disease. Annu Rev Neurosci. 2011;34:185‐204. 21456963 10.1146/annurev-neuro-061010-113613 PMC3174086

[1170] Saito F , Yanagisawa K , Miyatake T . Soluble derivatives of beta/A4 amyloid protein precursor in human cerebrospinal fluid are both N‐ and O‐glycosylated. Brain Res Mol Brain Res. 1993;19(1‐2):171‐174. 8361341 10.1016/0169-328x(93)90164-k

[1171] Kizuka Y , Kitazume S , Taniguchi N . N‐glycan and Alzheimer's disease. Biochim Biophys Acta Gen Subj. 2017;1861(10):2447‐2454. 28465241 10.1016/j.bbagen.2017.04.012

[1172] Nakagawa K , Kitazume S , Oka R , et al. Sialylation enhances the secretion of neurotoxic amyloid‐beta peptides. J Neurochem. 2006;96(4):924‐933. 16412100 10.1111/j.1471-4159.2005.03595.x

[1173] Halim A , Brinkmalm G , Rüetschi U , et al. Site‐specific characterization of threonine, serine, and tyrosine glycosylations of amyloid precursor protein/amyloid beta‐peptides in human cerebrospinal fluid. Proc Natl Acad Sci U S A. 2011;108(29):11848‐11853. 21712440 10.1073/pnas.1102664108 PMC3141957

[1174] Jacobsen KT , Iverfeldt K . O‐GlcNAcylation increases non‐amyloidogenic processing of the amyloid‐β precursor protein (APP). Biochem Biophys Res Commun. 2011;404(3):882‐886. 21182826 10.1016/j.bbrc.2010.12.080

[1175] Losev Y , Paul A , Frenkel‐Pinter M , et al. Novel model of secreted human tau protein reveals the impact of the abnormal N‐glycosylation of tau on its aggregation propensity. Sci Rep. 2019;9(1):2254. 30783169 10.1038/s41598-019-39218-x PMC6381127

[1176] Zhu Y , Shan X , Yuzwa SA , Vocadlo DJ . The emerging link between O‐GlcNAc and Alzheimer disease. J Biol Chem. 2014;289(50):34472‐34481. 25336656 10.1074/jbc.R114.601351 PMC4263855

[1177] Losev Y , Frenkel‐Pinter M , Abu‐Hussien M , et al. Differential effects of putative N‐glycosylation sites in human Tau on Alzheimer's disease‐related neurodegeneration. Cell Mol Life Sci. 2021;78(5):2231‐2245. 32926180 10.1007/s00018-020-03643-3 PMC11072875

[1178] Liu F , Iqbal K , Grundke‐Iqbal I , Hart GW , Gong CX . O‐GlcNAcylation regulates phosphorylation of tau: a mechanism involved in Alzheimer's disease. Proc Natl Acad Sci U S A. 2004;101(29):10804‐10809. 15249677 10.1073/pnas.0400348101 PMC490015

[1179] Rayaprolu S , Mullen B , Baker M , et al. TREM2 in neurodegeneration: evidence for association of the p.R47H variant with frontotemporal dementia and Parkinson's disease. Mol Neurodegener. 2013;8:19. 23800361 10.1186/1750-1326-8-19 PMC3691612

[1180] Park JS , Ji IJ , An HJ , et al. Disease‐associated mutations of TREM2 alter the processing of N‐linked oligosaccharides in the Golgi apparatus. Traffic. 2015;16(5):510‐518. 25615530 10.1111/tra.12264

[1181] Alfaro JF , Gong CX , Monroe ME , et al. Tandem mass spectrometry identifies many mouse brain O‐GlcNAcylated proteins including EGF domain‐specific O‐GlcNAc transferase targets. Proc Natl Acad Sci U S A. 2012;109(19):7280‐7285. 22517741 10.1073/pnas.1200425109 PMC3358849

[1182] Marotta NP , Lin YH , Lewis YE , et al. O‐GlcNAc modification blocks the aggregation and toxicity of the protein α‐synuclein associated with Parkinson's disease. Nat Chem. 2015;7(11):913‐920. 26492012 10.1038/nchem.2361 PMC4618406

[1183] Zhang XL , Qu H . The role of glycosylation in infectious diseases. Adv Exp Med Biol. 2021;1325:219‐237. 34495538 10.1007/978-3-030-70115-4_11

[1184] Lin B , Qing X , Liao J , Zhuo K . Role of protein glycosylation in host‐pathogen interaction. Cells. 2020;9(4):1022. 32326128 10.3390/cells9041022 PMC7226260

[1185] Hayashi S , Nagaoka K , Tanaka Y . Blood‐based biomarkers in hepatitis B virus‐related hepatocellular carcinoma, including the viral genome and glycosylated proteins. Int J Mol Sci. 2021;22(20):11051. 34681709 10.3390/ijms222011051 PMC8540379

[1186] Inoue T , Tanaka Y . Novel biomarkers for the management of chronic hepatitis B. Clin Mol Hepatol. 2020;26(3):261‐279. 32536045 10.3350/cmh.2020.0032 PMC7364351

[1187] Baudi I , Inoue T , Tanaka Y . Novel biomarkers of hepatitis B and hepatocellular carcinoma: clinical significance of HBcrAg and M2BPGi. Int J Mol Sci. 2020;21(3):949. 32023902 10.3390/ijms21030949 PMC7037346

[1188] Pongracz T , Nouta J , Wang W , et al. Immunoglobulin G1 Fc glycosylation as an early hallmark of severe COVID‐19. EBioMedicine. 2022;78:103957. 35334306 10.1016/j.ebiom.2022.103957 PMC8938159

[1189] Petrović T , Lauc G , Trbojević‐Akmačić I. The importance of glycosylation in COVID‐19 infection. Adv Exp Med Biol. 2021;1325:239‐264. 34495539 10.1007/978-3-030-70115-4_12

[1190] Kumar R , Tuen M , Li H , Tse DB , Hioe CE . Improving immunogenicity of HIV‐1 envelope gp120 by glycan removal and immune complex formation. Vaccine. 2011;29(48):9064‐9074. 21945958 10.1016/j.vaccine.2011.09.057 PMC3328143

[1191] Mathys L , François KO , Quandte M , Braakman I , Balzarini J . Deletion of the highly conserved N‐glycan at Asn260 of HIV‐1 gp120 affects folding and lysosomal degradation of gp120, and results in loss of viral infectivity. PloS One. 2014;9(6):e101181. 24967714 10.1371/journal.pone.0101181 PMC4072736

[1192] Vigerust DJ , Shepherd VL . Virus glycosylation: role in virulence and immune interactions. Trends Microbiol. 2007;15(5):211‐218. 17398101 10.1016/j.tim.2007.03.003 PMC7127133

[1193] Wolk T , Schreiber M . N‐Glycans in the gp120 V1/V2 domain of the HIV‐1 strain NL4‐3 are indispensable for viral infectivity and resistance against antibody neutralization. Med Microbiol Immunol. 2006;195(3):165‐172. 16547752 10.1007/s00430-006-0016-z

[1194] Watanabe Y , Bowden TA , Wilson IA , Crispin M . Exploitation of glycosylation in enveloped virus pathobiology. Biochim Biophys Acta Gen Subj. 2019;1863(10):1480‐1497. 31121217 10.1016/j.bbagen.2019.05.012 PMC6686077

[1195] Yolitz J , Schwing C , Chang J , et al. Signal peptide of HIV envelope protein impacts glycosylation and antigenicity of gp120. Proc Natl Acad Sci U S A. 2018;115(10):2443‐2448. 29463753 10.1073/pnas.1722627115 PMC5877976

[1196] Li H , Chien PC, Jr. , Tuen M , et al. Identification of an N‐linked glycosylation in the C4 region of HIV‐1 envelope gp120 that is critical for recognition of neighboring CD4 T cell epitopes. J Immunol. 2008;180(6):4011‐4021. 18322210 10.4049/jimmunol.180.6.4011

[1197] Moore JS , Wu X , Kulhavy R , et al. Increased levels of galactose‐deficient IgG in sera of HIV‐1‐infected individuals. Aids. 2005;19(4):381‐389. 15750391 10.1097/01.aids.0000161767.21405.68

[1198] Fischer W , Giorgi EE , Chakraborty S , et al. HIV‐1 and SARS‐CoV‐2: patterns in the evolution of two pandemic pathogens. Cell Host Microbe. 2021;29(7):1093‐1110. 34242582 10.1016/j.chom.2021.05.012 PMC8173590

[1199] Liu J , Hao Y , He Y , et al. Quantitative and site‐specific chemoproteomic profiling of protein O‐GlcNAcylation in the cell cycle. ACS Chem Biol. 2021;16(10):1917‐1923. 34161081 10.1021/acschembio.1c00301

[1200] Kim S , Seo Y , Chowdhury T , et al. Inhibition of MUC1 exerts cell‐cycle arrest and telomerase suppression in glioblastoma cells. Sci Rep. 2020;10(1):18238. 33106534 10.1038/s41598-020-75457-z PMC7589558

[1201] Nath S , Mukherjee P . MUC1: a multifaceted oncoprotein with a key role in cancer progression. Trends Mol Med. 2014;20(6):332‐342. 24667139 10.1016/j.molmed.2014.02.007 PMC5500204

[1202] Chou CH , Huang MJ , Chen CH , et al. Up‐regulation of C1GALT1 promotes breast cancer cell growth through MUC1‐C signaling pathway. Oncotarget. 2015;6(8):6123‐6135. 25762620 10.18632/oncotarget.3045 PMC4467426

[1203] Dalziel M , Whitehouse C , McFarlane I , et al. The relative activities of the C2GnT1 and ST3Gal‐I glycosyltransferases determine O‐glycan structure and expression of a tumor‐associated epitope on MUC1. J Biol Chem. 2001;276(14):11007‐1115. 11118434 10.1074/jbc.M006523200

[1204] Sewell R , Bäckström M , Dalziel M , et al. The ST6GalNAc‐I sialyltransferase localizes throughout the Golgi and is responsible for the synthesis of the tumor‐associated sialyl‐Tn O‐glycan in human breast cancer. J Biol Chem. 2006;281(6):3586‐3594. 16319059 10.1074/jbc.M511826200

[1205] Cascio S , Finn OJ . Intra‐ and extra‐cellular events related to altered glycosylation of MUC1 promote chronic Inflammation, tumor progression, invasion, and metastasis. Biomolecules. 2016;6(4):39. 27754373 10.3390/biom6040039 PMC5197949

[1206] Greco B , Malacarne V , De Girardi F , et al. Disrupting N‐glycan expression on tumor cells boosts chimeric antigen receptor T cell efficacy against solid malignancies. Sci Transl Med. 2022;14(628):eabg3072. 35044789 10.1126/scitranslmed.abg3072

[1207] RodrIguez E , Schetters STT , van Kooyk Y . The tumour glyco‐code as a novel immune checkpoint for immunotherapy. Nat Rev Immunol. 2018;18(3):204‐211. 29398707 10.1038/nri.2018.3

[1208] Perdicchio M , Cornelissen LA , Streng‐Ouwehand I , et al. Tumor sialylation impedes T cell mediated anti‐tumor responses while promoting tumor associated‐regulatory T cells. Oncotarget. 2016;7(8):8771‐8782. 26741508 10.18632/oncotarget.6822 PMC4891003

[1209] Lenos K , Goos JA , Vuist IM , et al. MGL ligand expression is correlated to BRAF mutation and associated with poor survival of stage III colon cancer patients. Oncotarget. 2015;6(28):26278‐26290. 26172302 10.18632/oncotarget.4495 PMC4694901

[1210] Dammen‐Brower K , Epler P , Zhu S , et al. Strategies for glycoengineering therapeutic proteins. Front Chem. 2022;10:863118. 35494652 10.3389/fchem.2022.863118 PMC9043614

[1211] Yang Z , Wang S , Halim A , et al. Engineered CHO cells for production of diverse, homogeneous glycoproteins. Nat Biotechnol. 2015;33(8):842‐844. 26192319 10.1038/nbt.3280

[1212] Hudak JE , Bertozzi CR . Glycotherapy: new advances inspire a reemergence of glycans in medicine. Chem Biol. 2014;21(1):16‐37. 24269151 10.1016/j.chembiol.2013.09.010 PMC4111574

[1213] Van Landuyt L , Lonigro C , Meuris L , Callewaert N . Customized protein glycosylation to improve biopharmaceutical function and targeting. Curr Opin Biotechnol. 2019;60:17‐28. 30554064 10.1016/j.copbio.2018.11.017

[1214] Broxmeyer HE . Erythropoietin: multiple targets, actions, and modifying influences for biological and clinical consideration. J Exp Med. 2013;210(2):205‐208. 23401569 10.1084/jem.20122760 PMC3570099

[1215] Walsh G , Jefferis R . Post‐translational modifications in the context of therapeutic proteins. Nat Biotechnol. 2006;24(10):1241‐1252. 17033665 10.1038/nbt1252

[1216] Fukuda MN , Sasaki H , Lopez L , Fukuda M . Survival of recombinant erythropoietin in the circulation: the role of carbohydrates. Blood. 1989;73(1):84‐89. 2910371

[1217] Erbayraktar S , Grasso G , Sfacteria A , et al. Asialoerythropoietin is a nonerythropoietic cytokine with broad neuroprotective activity in vivo. Proc Natl Acad Sci U S A. 2003;100(11):6741‐6746. 12746497 10.1073/pnas.1031753100 PMC164517

[1218] Albright RA , Stabach P , Cao W , et al. ENPP1‐Fc prevents mortality and vascular calcifications in rodent model of generalized arterial calcification of infancy. Nat Commun. 2015;6:10006. 26624227 10.1038/ncomms10006 PMC4686714

[1219] Stabach PR , Zimmerman K , Adame A , et al. Improving the pharmacodynamics and in vivo activity of ENPP1‐Fc through protein and glycosylation engineering. Clin Transl Sci. 2021;14(1):362‐372. 33064927 10.1111/cts.12887 PMC7877847

[1220] Jefferis R . Glycosylation as a strategy to improve antibody‐based therapeutics. Nat Rev Drug Discov. 2009;8(3):226‐234. 19247305 10.1038/nrd2804

[1221] Shields RL , Lai J , Keck R , et al. Lack of fucose on human IgG1 N‐linked oligosaccharide improves binding to human Fcgamma RIII and antibody‐dependent cellular toxicity. J Biol Chem. 2002;277(30):26733‐26740. 11986321 10.1074/jbc.M202069200

[1222] Mimura Y , Katoh T , Saldova R , et al. Glycosylation engineering of therapeutic IgG antibodies: challenges for the safety, functionality and efficacy. Protein Cell. 2018;9(1):47‐62. 28597152 10.1007/s13238-017-0433-3 PMC5777974

[1223] Raska M , Czernekova L , Moldoveanu Z , et al. Differential glycosylation of envelope gp120 is associated with differential recognition of HIV‐1 by virus‐specific antibodies and cell infection. AIDS Res Ther. 2014;11:23. 25120578 10.1186/1742-6405-11-23 PMC4130436

[1224] Valesini G , Gerardi MC , Iannuccelli C , Pacucci VA , Pendolino M , Shoenfeld Y . Citrullination and autoimmunity. Autoimmun Rev. 2015;14(6):490‐497. 25636595 10.1016/j.autrev.2015.01.013

[1225] Aratani S , Fujita H , Yagishita N , et al. Inhibitory effects of ubiquitination of synoviolin by PADI4. Mol Med Rep. 2017;16(6):9203‐9209. 29039504 10.3892/mmr.2017.7764

[1226] Travers TS , Harlow L , Rosas IO , et al. Extensive citrullination promotes immunogenicity of HSP90 through protein unfolding and exposure of cryptic epitopes. J Immunol. 2016;197(5):1926‐1936. 27448590 10.4049/jimmunol.1600162 PMC5061338

[1227] Tarcsa E , Marekov LN , Mei G , Melino G , Lee SC , Steinert PM . Protein unfolding by peptidylarginine deiminase. Substrate specificity and structural relationships of the natural substrates trichohyalin and filaggrin. J Biol Chem. 1996;271(48):30709‐30716. 8940048 10.1074/jbc.271.48.30709

[1228] Mondal S , Thompson PR . Protein arginine deiminases (PADs): biochemistry and chemical biology of protein citrullination. Acc Chem Res. 2019;52(3):818‐832. 30844238 10.1021/acs.accounts.9b00024 PMC6443095

[1229] Tilvawala R , Nguyen SH , Maurais AJ , et al. The rheumatoid arthritis‐associated citrullinome. Cell Chemical Biology. 2018;25(6):691‐704. e6. 29628436 10.1016/j.chembiol.2018.03.002 PMC6014894

[1230] Lee CY , Wang D , Wilhelm M , et al. Mining the human tissue proteome for protein citrullination. Mol Cell Proteomics. 2018;17(7):1378‐1391. 29610271 10.1074/mcp.RA118.000696 PMC6030718

[1231] Chavanas S , Mechin MC , Nachat R , et al. Peptidylarginine deiminases and deimination in biology and pathology: relevance to skin homeostasis. J Dermatol Sci. 2006;44(2):63‐72. 16973334 10.1016/j.jdermsci.2006.07.004

[1232] Guerrin M , Ishigami A , Mechin MC , et al. cDNA cloning, gene organization and expression analysis of human peptidylarginine deiminase type I. Biochem J. 2003;370(Pt 1):167‐174. 12416996 10.1042/BJ20020870 PMC1223146

[1233] Darrah E , Rosen A , Giles JT , Andrade F . Peptidylarginine deiminase 2, 3 and 4 have distinct specificities against cellular substrates: novel insights into autoantigen selection in rheumatoid arthritis. Ann Rheum Dis. 2012;71(1):92‐98. 21859690 10.1136/ard.2011.151712 PMC3302156

[1234] Watanabe K , Senshu T . Isolation and characterization of cDNA clones encoding rat skeletal muscle peptidylarginine deiminase. Journal of Biological Chemistry. 1989;264(26):15255‐15260. 2768262

[1235] Ishigami A , Ohsawa T , Hiratsuka M , et al. Abnormal accumulation of citrullinated proteins catalyzed by peptidylarginine deiminase in hippocampal extracts from patients with Alzheimer's disease. J Neurosci Res. 2005;80(1):120‐128. 15704193 10.1002/jnr.20431

[1236] Vossenaar ER , Radstake TR , van der Heijden A , et al. Expression and activity of citrullinating peptidylarginine deiminase enzymes in monocytes and macrophages. Ann Rheum Dis. 2004;63(4):373‐381. 15020330 10.1136/ard.2003.012211 PMC1754951

[1237] Chang X , Han J , Pang L , Zhao Y , Yang Y , Shen Z . Increased PADI4 expression in blood and tissues of patients with malignant tumors. BMC Cancer. 2009;9(1):40. 19183436 10.1186/1471-2407-9-40 PMC2637889

[1238] Wang S , Wang Y . Peptidylarginine deiminases in citrullination, gene regulation, health and pathogenesis. Biochim Biophys Acta. 2013;1829(10):1126‐1135. 23860259 10.1016/j.bbagrm.2013.07.003 PMC3775966

[1239] Liu X , Arfman T , Wichapong K , Reutelingsperger CPM , Voorberg J , Nicolaes GAF . PAD4 takes charge during neutrophil activation: impact of PAD4 mediated NET formation on immune‐mediated disease. J Thromb Haemost. 2021;19(7):1607‐1617. 33773016 10.1111/jth.15313 PMC8360066

[1240] Papayannopoulos V . Neutrophil extracellular traps in immunity and disease. Nat Rev Immunol. 2018;18(2):134‐147. 28990587 10.1038/nri.2017.105

[1241] Cristinziano L , Modestino L , Antonelli A , et al. Neutrophil extracellular traps in cancer. Semin Cancer Biol. 2022;79:91‐104. 34280576 10.1016/j.semcancer.2021.07.011

[1242] Yang M‐L , Sodré FMC , Mamula MJ , Overbergh L . Citrullination and PAD enzyme biology in Type 1 diabetes ‐ regulators of inflammation, autoimmunity, and pathology. Front Immunol. 2021;12:678953. 34140951 10.3389/fimmu.2021.678953 PMC8204103

[1243] Kan R , Yurttas P , Kim B , et al. Regulation of mouse oocyte microtubule and organelle dynamics by PADI6 and the cytoplasmic lattices. Dev Biol. 2011;350(2):311‐322. 21147087 10.1016/j.ydbio.2010.11.033 PMC3031771

[1244] Yu K , Proost P . Insights into peptidylarginine deiminase expression and citrullination pathways. Trends Cell Biol. 2022;32(9):746‐761. 35197210 10.1016/j.tcb.2022.01.014

[1245] Zhu D , Zhang Y , Wang S . Histone citrullination: a new target for tumors. Mol Cancer. 2021;20(1):90. 34116679 10.1186/s12943-021-01373-z PMC8192683

[1246] Ciesielski O , Biesiekierska M , Panthu B , Soszyński M , Pirola L , Balcerczyk A . Citrullination in the pathology of inflammatory and autoimmune disorders: recent advances and future perspectives. Cell Mol Life Sci. 2022;79(2):94. 35079870 10.1007/s00018-022-04126-3 PMC8788905

[1247] Asaga H , Yamada M , Senshu T . Selective deimination of vimentin in calcium ionophore‐induced apoptosis of mouse peritoneal macrophages. Biochem Biophys Res Commun. 1998;243(3):641‐646. 9500980 10.1006/bbrc.1998.8148

[1248] Vossenaar ER , Zendman AJ , van Venrooij WJ , Pruijn GJ . PAD, a growing family of citrullinating enzymes: genes, features and involvement in disease. Bioessays. 2003;25(11):1106‐1118. 14579251 10.1002/bies.10357

[1249] Sawata M , Shima H , Murayama K , et al. Autocitrullination and changes in the activity of peptidylarginine deiminase 3 induced by high Ca concentrations. ACS Omega. 2022;7(32):28378‐28387. 35990454 10.1021/acsomega.2c02972 PMC9386831

[1250] Mattson MP , Chan SL . Calcium orchestrates apoptosis. Nat Cell Biol. 2003;5(12):1041‐1043. 14647298 10.1038/ncb1203-1041

[1251] Hsu PC , Liao YF , Lin CL , Lin WH , Liu GY , Hung HC . Vimentin is involved in peptidylarginine deiminase 2‐induced apoptosis of activated Jurkat cells. Mol Cells. 2014;37(5):426‐434. 24850148 10.14348/molcells.2014.2359 PMC4044315

[1252] György B , Tóth E , Tarcsa E , Falus A , Buzás EI . Citrullination: a posttranslational modification in health and disease. Int J Biochem Cell Biol. 2006;38(10):1662‐1677. 16730216 10.1016/j.biocel.2006.03.008

[1253] Mondal S , Thompson PR . Chemical biology of protein citrullination by the protein A arginine deiminases. Curr Opin Chem Biol. 2021;63:19‐27. 33676233 10.1016/j.cbpa.2021.01.010 PMC8384633

[1254] Zhang X , Bolt M , Guertin MJ , et al. Peptidylarginine deiminase 2‐catalyzed histone H3 arginine 26 citrullination facilitates estrogen receptor alpha target gene activation. Proc Natl Acad Sci U S A. 2012;109(33):13331‐13336. 22853951 10.1073/pnas.1203280109 PMC3421185

[1255] Guo W , Zheng Y , Xu B , et al. Investigating the expression, effect and tumorigenic pathway of PADI2 in tumors. Onco Targets Ther. 2017;10:1475‐1485. 28331341 10.2147/OTT.S92389 PMC5352236

[1256] Wang Y , Chen R , Gan Y , Ying S . The roles of PAD2‐ and PAD4‐mediated protein citrullination catalysis in cancers. Int J Cancer. 2021;148(2):267‐276. 33459350 10.1002/ijc.33205

[1257] Wang H , Xu B , Zhang X , Zheng Y , Zhao Y , Chang X . PADI2 gene confers susceptibility to breast cancer and plays tumorigenic role via ACSL4, BINC3 and CA9 signaling. Cancer Cell Int. 2016;16:61. 27478411 10.1186/s12935-016-0335-0 PMC4966586

[1258] Lu J , Wu J , Xia X , Peng H , Wang S . Follicular helper T cells: potential therapeutic targets in rheumatoid arthritis. Cell Mol Life Sci. 2021;78(12):5095‐5106. 33880615 10.1007/s00018-021-03839-1 PMC11073436

[1259] Raptopoulou A , Sidiropoulos P , Katsouraki M , Boumpas DT . Anti‐citrulline antibodies in the diagnosis and prognosis of rheumatoid arthritis: evolving concepts. Crit Rev Clin Lab Sci. 2007;44(4):339‐363. 17558653 10.1080/10408360701295623

[1260] Visser H , le Cessie S , Vos K , Breedveld FC , Hazes JM . How to diagnose rheumatoid arthritis early: a prediction model for persistent (erosive) arthritis. Arthritis Rheum. 2002;46(2):357‐365. 11840437 10.1002/art.10117

[1261] Trouw LA , Mahler M . Closing the serological gap: promising novel biomarkers for the early diagnosis of rheumatoid arthritis. Autoimmun Rev. 2012;12(2):318‐322. 22664776 10.1016/j.autrev.2012.05.007

[1262] Mechin MC , Sebbag M , Arnaud J , et al. Update on peptidylarginine deiminases and deimination in skin physiology and severe human diseases. Int J Cosmet Sci. 2007;29(3):147‐168. 18489346 10.1111/j.1467-2494.2007.00377.x

[1263] Wegner N , Lundberg K , Kinloch A , et al. Autoimmunity to specific citrullinated proteins gives the first clues to the etiology of rheumatoid arthritis. Immunol Rev. 2010;233(1):34‐54. 20192991 10.1111/j.0105-2896.2009.00850.x

[1264] Zhu D , Song W , Jiang Z , Zhou H , Wang S . Citrullination: a modification important in the pathogenesis of autoimmune diseases. Clin Immunol. 2022;245:109134. 36184053 10.1016/j.clim.2022.109134

[1265] Chang X , Yamada R , Sawada T , Suzuki A , Kochi Y , Yamamoto K . The inhibition of antithrombin by peptidylarginine deiminase 4 may contribute to pathogenesis of rheumatoid arthritis. Rheumatology (Oxford). 2005;44(3):293‐298. 15561738 10.1093/rheumatology/keh473

[1266] Darrah E , Andrade F . Rheumatoid arthritis and citrullination. Curr Opin Rheumatol. 2018;30(1):72‐78. 28937414 10.1097/BOR.0000000000000452 PMC5848217

[1267] Bradford CM , Ramos I , Cross AK , et al. Localisation of citrullinated proteins in normal appearing white matter and lesions in the central nervous system in multiple sclerosis. J Neuroimmunol. 2014;273(1‐2):85‐95. 24907905 10.1016/j.jneuroim.2014.05.007

[1268] Wood DD , Ackerley CA , Brand B , et al. Myelin localization of peptidylarginine deiminases 2 and 4: comparison of PAD2 and PAD4 activities. Lab Invest. 2008;88(4):354‐364. 18227806 10.1038/labinvest.3700748

[1269] Musse AA , Li Z , Ackerley CA , et al. Peptidylarginine deiminase 2 (PAD2) overexpression in transgenic mice leads to myelin loss in the central nervous system. Dis Model Mech. 2008;1(4‐5):229‐240. 19093029 10.1242/dmm.000729 PMC2590822

[1270] Anzilotti C , Pratesi F , Tommasi C , Migliorini P . Peptidylarginine deiminase 4 and citrullination in health and disease. Autoimmun Rev. 2010;9(3):158‐160. 19540364 10.1016/j.autrev.2009.06.002

[1271] Kiriakidou M , Ching CL . Systemic lupus erythematosus. Ann Intern Med. 2020;172(11):ITC81‐ITC96. 32479157 10.7326/AITC202006020

[1272] Lande R , Palazzo R , Gestermann N , et al. Native/citrullinated LL37‐specific T‐cells help autoantibody production in Systemic Lupus Erythematosus. Sci Rep. 2020;10(1):5851. 32245990 10.1038/s41598-020-62480-3 PMC7125190

[1273] Li P , Li M , Lindberg MR , Kennett MJ , Xiong N , Wang Y . PAD4 is essential for antibacterial innate immunity mediated by neutrophil extracellular traps. J Exp Med. 2010;207(9):1853‐1862. 20733033 10.1084/jem.20100239 PMC2931169

[1274] Fresneda Alarcon M , McLaren Z , Wright HL . Neutrophils in the pathogenesis of rheumatoid arthritis and systemic lupus erythematosus: same foe different M.O. Front Immunol. 2021;12:649693. 33746988 10.3389/fimmu.2021.649693 PMC7969658

[1275] Bieging KT , Mello SS , Attardi LD . Unravelling mechanisms of p53‐mediated tumour suppression. Nat Rev Cancer. 2014;14(5):359‐370. 24739573 10.1038/nrc3711 PMC4049238

[1276] Chang X , Han J . Expression of peptidylarginine deiminase type 4 (PAD4) in various tumors. Mol Carcinog. 2006;45(3):183‐196. 16355400 10.1002/mc.20169

[1277] Tanikawa C , Ueda K , Nakagawa H , Yoshida N , Nakamura Y , Matsuda K . Regulation of protein citrullination through p53/PADI4 network in DNA damage response. Cancer Res. 2009;69(22):8761‐8769. 19843866 10.1158/0008-5472.CAN-09-2280

[1278] Algaba A , Guerra I , Ricart E , et al. Extraintestinal manifestations in patients with inflammatory bowel disease: study based on the ENEIDA registry. Dig Dis Sci. 2021;66(6):2014‐2023. 32671587 10.1007/s10620-020-06424-x

[1279] Chumanevich AA , Causey CP , Knuckley BA , et al. Suppression of colitis in mice by Cl‐amidine: a novel peptidylarginine deiminase inhibitor. Am J Physiol Gastrointest Liver Physiol. 2011;300(6):G929‐G938. 21415415 10.1152/ajpgi.00435.2010 PMC3119113

[1280] Dinallo V , Marafini I , Di Fusco D , et al. Neutrophil extracellular traps sustain inflammatory signals in ulcerative colitis. J Crohns Colitis. 2019;13(6):772‐784. 30715224 10.1093/ecco-jcc/jjy215

[1281] Kobayashi T , Siegmund B , Le Berre C , et al. Ulcerative colitis. Nat Rev Dis Primers. 2020;6(1):74. 32913180 10.1038/s41572-020-0205-x

[1282] Leppkes M , Lindemann A , Gößwein S , et al. Neutrophils prevent rectal bleeding in ulcerative colitis by peptidyl‐arginine deiminase‐4‐dependent immunothrombosis. Gut. 2022;71(12):2414‐2429. 34862250 10.1136/gutjnl-2021-324725 PMC9667856

[1283] Jaisson S , Pietrement C , Gillery P . Protein carbamylation: chemistry, pathophysiological involvement, and biomarkers. Adv Clin Chem. 2018;84:1‐38. 29478512 10.1016/bs.acc.2017.12.001

[1284] Rwatambuga FA , Ali ER , Bramble MS , et al. Motor control and cognition deficits associated with protein carbamoylation in food (cassava) cyanogenic poisoning: Neurodegeneration and genomic perspectives. Food Chem Toxicol. 2021;148:111917. 33296712 10.1016/j.fct.2020.111917 PMC7855927

[1285] Badar A , Arif Z , Alam K . Role of carbamylated biomolecules in human diseases. IUBMB Life. 2018;70(4):267‐275. 29542227 10.1002/iub.1732

[1286] Shi J , van Veelen PA , Mahler M , et al. Carbamylation and antibodies against carbamylated proteins in autoimmunity and other pathologies. Autoimmun Rev. 2014;13(3):225‐230. 24176675 10.1016/j.autrev.2013.10.008

[1287] Verbrugge FH , Tang WH , Hazen SL . Protein carbamylation and cardiovascular disease. Kidney Int. 2015;88(3):474‐478. 26061545 10.1038/ki.2015.166 PMC4556561

[1288] Wang Z , Nicholls SJ , Rodriguez ER , et al. Protein carbamylation links inflammation, smoking, uremia and atherogenesis. Nat Med. 2007;13(10):1176‐1184. 17828273 10.1038/nm1637

[1289] Anaya JM , Restrepo‐Jiménez P , Ramírez‐Santana C . The autoimmune ecology: an update. Curr Opin Rheumatol. 2018;30(4):350‐360. 29438164 10.1097/BOR.0000000000000498

[1290] Ospelt C , Bang H , Feist E , et al. Carbamylation of vimentin is inducible by smoking and represents an independent autoantigen in rheumatoid arthritis. Ann Rheum Dis. 2017;76(7):1176‐1183. 28183721 10.1136/annrheumdis-2016-210059 PMC5530349

[1291] Jaisson S , Pietrement C , Gillery P . Carbamylation‐derived products: bioactive compounds and potential biomarkers in chronic renal failure and atherosclerosis. Clin Chem. 2011;57(11):1499‐1505. 21768218 10.1373/clinchem.2011.163188

[1292] Simsek B , Yanar K , Çakatay U . Proatherogenic importance of carbamylation‐induced protein damage and type 2 diabetes mellitus: a systematic review. Curr Diabetes Rev. 2020;16(6):608‐618. 31914914 10.2174/1573399816666200107102918

[1293] Koro C , Hellvard A , Delaleu N , et al. Carbamylated LL‐37 as a modulator of the immune response. Innate Immun. 2016;22(3):218‐229. 26878866 10.1177/1753425916631404 PMC5143673

[1294] Nakabo S , Hashimoto M , Ito S , et al. Carbamylated albumin is one of the target antigens of anti‐carbamylated protein antibodies. Rheumatology (Oxford). 2017;56(7):1217‐1226. 28398552 10.1093/rheumatology/kex088 PMC5850724

[1295] Binder V , Bergum B , Jaisson S , et al. Impact of fibrinogen carbamylation on fibrin clot formation and stability. Thromb Haemost. 2017;117(5):899‐910. 28382370 10.1160/TH16-09-0704 PMC5442607

[1296] Gorisse L , Pietrement C , Vuiblet V , et al. Protein carbamylation is a hallmark of aging. Proc Natl Acad Sci U S A. 2016;113(5):1191‐1196. 26712018 10.1073/pnas.1517096113 PMC4747743

[1297] Maciejczyk M , Nesterowicz M , Szulimowska J , Zalewska A . Oxidation, glycation, and carbamylation of salivary biomolecules in healthy children, adults, and the elderly: can saliva be used in the assessment of aging? J Inflamm Res. 2022;15:2051‐2073. 35378954 10.2147/JIR.S356029 PMC8976116

[1298] Carracedo J , Ramírez‐Carracedo R , Martínez de Toda I , et al. Protein carbamylation: a marker reflecting increased age‐related cell oxidation. Int J Mol Sci. 2018;19(5):1495. 29772765 10.3390/ijms19051495 PMC5983744

[1299] Harding JJ . Viewing molecular mechanisms of ageing through a lens. Ageing Res Rev. 2002;1(3):465‐479. 12067598 10.1016/s1568-1637(02)00012-0

[1300] Derham BK , Harding JJ . Alpha‐crystallin as a molecular chaperone. Prog Retin Eye Res. 1999;18(4):463‐509. 10217480 10.1016/s1350-9462(98)00030-5

[1301] Gorisse L , Jaisson S , Pietrement C , Gillery P . Carbamylated proteins in renal disease: aggravating factors or just biomarkers? Int J Mol Sci. 2022;23(1):574‐593. 35008998 10.3390/ijms23010574 PMC8745352

[1302] Pietrement C , Gorisse L , Jaisson S , Gillery P . Chronic increase of urea leads to carbamylated proteins accumulation in tissues in a mouse model of CKD. PLoS One. 2013;8(12):e82506. 24324801 10.1371/journal.pone.0082506 PMC3853192

[1303] Drechsler C , Kalim S , Wenger JB , et al. Protein carbamylation is associated with heart failure and mortality in diabetic patients with end‐stage renal disease. Kidney Int. 2015;87(6):1201‐1208. 25671766 10.1038/ki.2014.429 PMC4449819

[1304] Tan KCB , Cheung C‐L , Lee ACH , Lam JKY , Wong Y , Shiu SWM . Carbamylated lipoproteins and progression of diabetic kidney disease. Clin J Am Soc Nephrol. 2020;15(3):359‐366. 32075807 10.2215/CJN.11710919 PMC7057307

[1305] Apostolov EO , Ray D , Savenka AV , Shah SV , Basnakian AG . Chronic uremia stimulates LDL carbamylation and atherosclerosis. J Am Soc Nephrol. 2010;21(11):1852‐1857. 20947625 10.1681/ASN.2010040365 PMC3014000

[1306] Speer T , Ridker PM , von Eckardstein A , Schunk SJ , Fliser D . Lipoproteins in chronic kidney disease: from bench to bedside. Eur Heart J. 2021;42(22):2170‐2185. 33393990 10.1093/eurheartj/ehaa1050

[1307] Holy EW , Akhmedov A , Speer T , et al. Carbamylated low‐density lipoproteins induce a prothrombotic state via LOX‐1: impact on arterial thrombus formation in vivo. J Am Coll Cardiol. 2016;68(15):1664‐1676. 27712780 10.1016/j.jacc.2016.07.755

[1308] Hawkins CL . Protein carbamylation: a key driver of vascular calcification during chronic kidney disease. Kidney Int. 2018;94(1):12‐14. 29933841 10.1016/j.kint.2018.03.022

[1309] Mori D , Matsui I , Shimomura A , et al. Protein carbamylation exacerbates vascular calcification. Kidney Int. 2018;94(1):72‐90. 29716796 10.1016/j.kint.2018.01.033

[1310] Shah SV , Shukla AM , Bose C , Basnakian AG , Rajapurkar M . Recent advances in understanding the pathogenesis of atherosclerosis in CKD patients. J Ren Nutr. 2015;25(2):205‐208. 25556310 10.1053/j.jrn.2014.10.024

[1311] Santana JM , Brown CD . High‐density lipoprotein carbamylation and dysfunction in vascular disease. Front Biosci (Landmark Ed). 2018;23(12):2227‐2234. 29772557 10.2741/4701

[1312] Holzer M , Gauster M , Pfeifer T , et al. Protein carbamylation renders high‐density lipoprotein dysfunctional. Antioxid Redox Signal. 2011;14(12):2337‐2346. 21235354 10.1089/ars.2010.3640 PMC3380531

[1313] Doué M , Okwieka A , Berquand A , et al. Carbamylation of elastic fibers is a molecular substratum of aortic stiffness. Sci Rep. 2021;11(1):17827. 34497312 10.1038/s41598-021-97293-5 PMC8426361

[1314] van Venrooij WJ , van Beers JJ , Pruijn GJ . Anti‐CCP antibodies: the past, the present and the future. Nat Rev Rheumatol. 2011;7(7):391‐398. 21647203 10.1038/nrrheum.2011.76

[1315] Shi J , Knevel R , Suwannalai P , et al. Autoantibodies recognizing carbamylated proteins are present in sera of patients with rheumatoid arthritis and predict joint damage. Proc Natl Acad Sci U S A. 2011;108(42):17372‐17377. 21987802 10.1073/pnas.1114465108 PMC3198314

[1316] O'Neil LJ , Barrera‐Vargas A , Sandoval‐Heglund D , et al. Neutrophil‐mediated carbamylation promotes articular damage in rheumatoid arthritis. Sci Adv. 2020;6(44):eabd2688. 33115748 10.1126/sciadv.abd2688 PMC7608797

[1317] Kwon EJ , Ju JH . Impact of posttranslational modification in pathogenesis of rheumatoid arthritis: focusing on citrullination, carbamylation, and acetylation. Int J Mol Sci. 2021;22(19):10576‐10604. 34638916 10.3390/ijms221910576 PMC8508717

[1318] Farías G , González‐Billault C , Maccioni RB . Immunological characterization of epitopes on tau of Alzheimer's type and chemically modified tau. Mol Cell Biochem. 1997;168(1‐2):59‐66. 9062894 10.1023/a:1006838626730

[1319] Moterroso SKV . Carbamoylation correlates of cyanate neuropathy and cyanide poisoning: relevance to the biomarkers of cassava cyanogenesis and motor system toxicity. SpringerPlus. 2013;2:647‐655. 24349951 10.1186/2193-1801-2-647 PMC3862856

[1320] Go YM , Chandler JD , Jones DP . The cysteine proteome. Free Radic Biol Med. 2015;84:227‐245. 25843657 10.1016/j.freeradbiomed.2015.03.022 PMC4457640

[1321] UniProt C . UniProt: a worldwide hub of protein knowledge. Nucleic Acids Res. 2019;47(D1):D506‐D515. 30395287 10.1093/nar/gky1049 PMC6323992

[1322] Yang J . Mapping the cysteine redoxome: advances and prospects. SCIENTIA SINICA Vitae. 2021;51(11):1558‐1570.

[1323] Young A , Gill R , Mailloux RJ . Protein S‐glutathionylation: the linchpin for the transmission of regulatory information on redox buffering capacity in mitochondria. Chem Biol Interact. 2019;299:151‐162. 30537466 10.1016/j.cbi.2018.12.003

[1324] Depuydt M , Leonard SE , Vertommen D , et al. A periplasmic reducing system protects single cysteine residues from oxidation. Science. 2009;326(5956):1109‐1111. 19965429 10.1126/science.1179557

[1325] Bhatia V , Elnagary L , Dakshinamurti S . Tracing the path of inhaled nitric oxide: biological consequences of protein nitrosylation. Pediatr Pulmonol. 2021;56(2):525‐538. 33289321 10.1002/ppul.25201

[1326] Evangelista AM , Kohr MJ , Murphy E . S‐nitrosylation: specificity, occupancy, and interaction with other post‐translational modifications. Antioxid Redox Signal. 2013;19(11):1209‐1219. 23157187 10.1089/ars.2012.5056 PMC3785808

[1327] Kornberg MD , Sen N , Hara MR , et al. GAPDH mediates nitrosylation of nuclear proteins. Nat Cell Biol. 2010;12(11):1094‐1100. 20972425 10.1038/ncb2114 PMC2972384

[1328] Hosseininasab V , McQuilken AC , Bakhoda AG , Bertke JA , Timerghazin QK , Warren TH . Lewis acid coordination redirects S‐nitrosothiol signaling output. Angew Chem Int Ed Engl. 2020;59(27):10854‐10858. 32090399 10.1002/anie.202001450 PMC7385465

[1329] Wojdyla K , Rogowska‐Wrzesinska A . Differential alkylation‐based redox proteomics–Lessons learnt. Redox Biol. 2015;6:240‐252. 26282677 10.1016/j.redox.2015.08.005 PMC4543216

[1330] Furuta S . Basal S‐nitrosylation is the guardian of tissue homeostasis. Trends Cancer. 2017;3(11):744‐748. 29120749 10.1016/j.trecan.2017.09.003

[1331] Hess DT , Stamler JS . Regulation by S‐nitrosylation of protein post‐translational modification. J Biol Chem. 2012;287(7):4411‐4418. 22147701 10.1074/jbc.R111.285742 PMC3281651

[1332] Zhao QF , Yu JT , Tan L . S‐nitrosylation in Alzheimer's disease. Mol Neurobiol. 2015;51(1):268‐280. 24664522 10.1007/s12035-014-8672-2

[1333] Perrin‐Sarrado C , Zhou Y , Salgues V , et al. Protein S‐nitrosylation as a therapeutic target for neurodegenerative diseases. Biochem Pharmacol. 2020;173:113686. 31678494 10.1016/j.bcp.2019.113686

[1334] Tan C , Li Y , Huang X , et al. Extensive protein S‐nitrosylation associated with human pancreatic ductal adenocarcinoma pathogenesis. Cell Death Dis. 2019;10(12):914. 31801946 10.1038/s41419-019-2144-6 PMC6892852

[1335] Chia SB , Elko EA , Aboushousha R , et al. Dysregulation of the glutaredoxin/S‐glutathionylation redox axis in lung diseases. Am J Physiol Cell Physiol. 2020;318(2):C304‐C327. 31693398 10.1152/ajpcell.00410.2019 PMC7052607

[1336] Kukulage DSK , Matarage Don NNJ , Ahn YH . Emerging chemistry and biology in protein glutathionylation. Curr Opin Chem Biol. 2022;71:102221. 36223700 10.1016/j.cbpa.2022.102221 PMC9844265

[1337] Ye ZW , Zhang J , Ancrum T , Manevich Y , Townsend DM , Tew KD . Glutathione S‐transferase P‐mediated protein S‐glutathionylation of resident endoplasmic reticulum proteins influences sensitivity to drug‐induced unfolded protein response. Antioxid Redox Signal. 2017;26(6):247‐261. 26838680 10.1089/ars.2015.6486 PMC5312626

[1338] Burns M , Rizvi SHM , Tsukahara Y , et al. Role of glutaredoxin‐1 and glutathionylation in cardiovascular diseases. Int J Mol Sci. 2020;21(18):6803. 32948023 10.3390/ijms21186803 PMC7555996

[1339] Zhang J , Ye ZW , Singh S , Townsend DM , Tew KD . An evolving understanding of the S‐glutathionylation cycle in pathways of redox regulation. Free Radic Biol Med. 2018;120:204‐216. 29578070 10.1016/j.freeradbiomed.2018.03.038 PMC5940525

[1340] Shih YY , Lin HY , Jan HM , et al. S‐glutathionylation of Hsp90 enhances its degradation and correlates with favorable prognosis of breast cancer. Redox Biol. 2022;57:102501. 36279628 10.1016/j.redox.2022.102501 PMC9594641

[1341] Watanabe Y , Watanabe K , Fujioka D , et al. Protein S‐glutathionylation stimulate adipogenesis by stabilizing C/EBPβ in 3T3L1 cells. FASEB J. 2020;34(4):5827‐5837. 32141127 10.1096/fj.201902575R PMC8491561

[1342] Mustafa Rizvi SH , Shao D , Tsukahara Y , et al. Oxidized GAPDH transfers S‐glutathionylation to a nuclear protein Sirtuin‐1 leading to apoptosis. Free Radic Biol Med. 2021;174:73‐83. 34332079 10.1016/j.freeradbiomed.2021.07.037 PMC8432375

[1343] Corteselli E , Aboushousha R , Janssen‐Heininger Y . S‐glutathionylation‐controlled apoptosis of lung epithelial cells; potential implications for lung fibrosis. Antioxidants (Basel). 2022;11(9):1789. 36139863 10.3390/antiox11091789 PMC9495907

[1344] Gupta V , Carroll KS . Sulfenic acid chemistry, detection and cellular lifetime. Biochim Biophys Acta. 2014;1840(2):847‐875. 23748139 10.1016/j.bbagen.2013.05.040 PMC4184475

[1345] Alcock LJ , Perkins MV , Chalker JM . Chemical methods for mapping cysteine oxidation. Chem Soc Rev. 2018;47(1):231‐268. 29242887 10.1039/c7cs00607a

[1346] Wojdyla K , Williamson J , Roepstorff P , Rogowska‐Wrzesinska A . The SNO/SOH TMT strategy for combinatorial analysis of reversible cysteine oxidations. J Proteomics. 2015;113:415‐434. 25449835 10.1016/j.jprot.2014.10.015

[1347] Truong TH , Carroll KS . Redox regulation of epidermal growth factor receptor signaling through cysteine oxidation. Biochemistry. 2012;51(50):9954‐9965. 23186290 10.1021/bi301441e PMC3525721

[1348] Heppner DE , Dustin CM , Liao C , et al. Direct cysteine sulfenylation drives activation of the Src kinase. Nat Commun. 2018;9(1):4522. 30375386 10.1038/s41467-018-06790-1 PMC6207713

[1349] Yang M , Li W , Harberg C , et al. Cysteine sulfenylation by CD36 signaling promotes arterial thrombosis in dyslipidemia. Blood Adv. 2020;4(18):4494‐4507. 32946569 10.1182/bloodadvances.2020001609 PMC7509873

[1350] Chouchani ET , Kazak L , Jedrychowski MP , et al. Mitochondrial ROS regulate thermogenic energy expenditure and sulfenylation of UCP1. Nature. 2016;532(7597):112‐116. 27027295 10.1038/nature17399 PMC5549630

[1351] Meng J , Fu L , Liu K , et al. Global profiling of distinct cysteine redox forms reveals wide‐ranging redox regulation in C. elegans. Nat Commun. 2021;12(1):1415. 33658510 10.1038/s41467-021-21686-3 PMC7930113

[1352] Hourihan JM , Moronetti Mazzeo LE , Fernandez‐Cardenas LP , Blackwell TK . Cysteine sulfenylation directs IRE‐1 to activate the SKN‐1/Nrf2 antioxidant response. Mol Cell. 2016;63(4):553‐566. 27540856 10.1016/j.molcel.2016.07.019 PMC4996358

[1353] Kim Y‐M , Krantz S , Jambusaria A , et al. Mitofusin‐2 stabilizes adherens junctions and suppresses endothelial inflammation via modulation of β‐catenin signaling. Nat Commun. 2021;12(1):2736. 33980844 10.1038/s41467-021-23047-6 PMC8115264

[1354] Lo Conte M , Carroll KS . The redox biochemistry of protein sulfenylation and sulfinylation. J Biol Chem. 2013;288(37):26480‐26488. 23861405 10.1074/jbc.R113.467738 PMC3772195

[1355] Woo HA , Chae HZ , Hwang SC , et al. Reversing the inactivation of peroxiredoxins caused by cysteine sulfinic acid formation. Science. 2003;300(5619):653‐656. 12714748 10.1126/science.1080273

[1356] Biteau B , Labarre J , Toledano MB . ATP‐dependent reduction of cysteine‐sulphinic acid by S. cerevisiae sulphiredoxin. Nature. 2003;425(6961):980‐984. 14586471 10.1038/nature02075

[1357] Chang TS , Jeong W , Woo HA , Lee SM , Park S , Rhee SG . Characterization of mammalian sulfiredoxin and its reactivation of hyperoxidized peroxiredoxin through reduction of cysteine sulfinic acid in the active site to cysteine. J Biol Chem. 2004;279(49):50994‐51001. 15448164 10.1074/jbc.M409482200

[1358] Akter S , Fu L , Jung Y , et al. Chemical proteomics reveals new targets of cysteine sulfinic acid reductase. Nat Chem Biol. 2018;14(11):995‐1004. 30177848 10.1038/s41589-018-0116-2 PMC6192846

[1359] Chae HZ , Kim HJ , Kang SW , Rhee SG . Characterization of three isoforms of mammalian peroxiredoxin that reduce peroxides in the presence of thioredoxin. Diabetes Res Clin Pract. 1999;45(2‐3):101‐112. 10588361 10.1016/s0168-8227(99)00037-6

[1360] Jacob C , Holme AL , Fry FH . The sulfinic acid switch in proteins. Org Biomol Chem. 2004;2(14):1953‐1956. 15254616 10.1039/b406180b

[1361] Blackinton J , Lakshminarasimhan M , Thomas KJ , et al. Formation of a stabilized cysteine sulfinic acid is critical for the mitochondrial function of the parkinsonism protein DJ‐1. J Biol Chem. 2009;284(10):6476‐6485. 19124468 10.1074/jbc.M806599200 PMC2649108

[1362] Maret W . Zinc coordination environments in proteins as redox sensors and signal transducers. Antioxid Redox Signal. 2006;8(9‐10):1419‐1441. 16987000 10.1089/ars.2006.8.1419

[1363] Tombling BJ , Wang CK , Craik DJ . EGF‐like and other disulfide‐rich microdomains as therapeutic scaffolds. Angew Chem Int Ed Engl. 2020;59(28):11218‐11232. 31867866 10.1002/anie.201913809

[1364] Orr CM , Fisher H , Yu X , et al. Hinge disulfides in human IgG2 CD40 antibodies modulate receptor signaling by regulation of conformation and flexibility. Sci Immunol. 2022;7(73):eabm3723. 35857577 10.1126/sciimmunol.abm3723

[1365] Paulsen CE , Carroll KS . Cysteine‐mediated redox signaling: chemistry, biology, and tools for discovery. Chem Rev. 2013;113(7):4633‐4679. 23514336 10.1021/cr300163e PMC4303468

[1366] Bazopoulou D , Knoefler D , Zheng Y , et al. Developmental ROS individualizes organismal stress resistance and lifespan. Nature. 2019;576(7786):301‐305. 31801997 10.1038/s41586-019-1814-y PMC7039399

[1367] Zheng X , Yang Z , Gu Q , et al. The protease activity of human ATG4B is regulated by reversible oxidative modification. Autophagy. 2020;16(10):1838‐1850. 31880198 10.1080/15548627.2019.1709763 PMC8386634

[1368] Wood MJ , Storz G , Tjandra N . Structural basis for redox regulation of Yap1 transcription factor localization. Nature. 2004;430(7002):917‐921. 15318225 10.1038/nature02790

[1369] Yang H , Lundback P , Ottosson L , et al. Redox modifications of cysteine residues regulate the cytokine activity of HMGB1. Mol Med. 2021;27(1):58. 34098868 10.1186/s10020-021-00307-1 PMC8185929

[1370] Martina JA , Guerrero‐Gómez D , Gómez‐Orte E , et al. A conserved cysteine‐based redox mechanism sustains TFEB/HLH‐30 activity under persistent stress. EMBO J. 2021;40(3):e105793. 33314217 10.15252/embj.2020105793 PMC7849306

[1371] Zamorano Cuervo N, Fortin A , Caron E , Chartier S , Grandvaux N . Pinpointing cysteine oxidation sites by high‐resolution proteomics reveals a mechanism of redox‐dependent inhibition of human STING. Sci Signal. 2021;14(680):eaaw4673. 33906974 10.1126/scisignal.aaw4673

[1372] Finelli MJ . Redox post‐translational modifications of protein thiols in brain aging and neurodegenerative conditions‐focus on S‐nitrosation. Front Aging Neurosci. 2020;12:254. 33088270 10.3389/fnagi.2020.00254 PMC7497228

[1373] Pham TK , Buczek WA , Mead RJ , Shaw PJ , Collins MO . Proteomic approaches to study cysteine oxidation: applications in neurodegenerative diseases. Front Mol Neurosci. 2021;14:678837. 34177463 10.3389/fnmol.2021.678837 PMC8219902

[1374] Andreadou I , Efentakis P , Frenis K , Daiber A , Schulz R . Thiol‐based redox‐active proteins as cardioprotective therapeutic agents in cardiovascular diseases. Basic Res Cardiol. 2021;116(1):44. 34275052 10.1007/s00395-021-00885-5

[1375] Xiao H , Jedrychowski MP , Schweppe DK , et al. A quantitative tissue‐specific landscape of protein redox regulation during aging. Cell. 2020;180(5):968‐983. e24. 32109415 10.1016/j.cell.2020.02.012 PMC8164166

[1376] Gusarov I , Shamovsky I , Pani B , et al. Dietary thiols accelerate aging of C. elegans. Nat Commun. 2021;12(1):4336. 34267196 10.1038/s41467-021-24634-3 PMC8282788

[1377] Qiao X , Zhang Y , Ye A , et al. ER reductive stress caused by Ero1α S‐nitrosation accelerates senescence. Free Radic Biol Med. 2022;180:165‐178. 35033630 10.1016/j.freeradbiomed.2022.01.006

[1378] Matsumaru D , Motohashi H . The KEAP1‐NRF2 system in healthy aging and longevity. Antioxidants (Basel). 2021;10(12):1929. 34943032 10.3390/antiox10121929 PMC8750203

[1379] Montagna C , Cirotti C , Rizza S , Filomeni G . When S‐nitrosylation gets to mitochondria: from signaling to age‐related diseases. Antioxid Redox Signal. 2020;32(12):884‐905. 31931592 10.1089/ars.2019.7872

[1380] Foreman NA , Hesse AS , Ji LL . Redox signaling and sarcopenia: searching for the primary suspect. Int J Mol Sci. 2021;22(16):9045. 34445751 10.3390/ijms22169045 PMC8396474

[1381] Campbell MD , Duan J , Samuelson AT , et al. Improving mitochondrial function with SS‐31 reverses age‐related redox stress and improves exercise tolerance in aged mice. Free Radic Biol Med. 2019;134:268‐281. 30597195 10.1016/j.freeradbiomed.2018.12.031 PMC6588449

[1382] Kramer PA , Duan J , Gaffrey MJ , et al. Fatiguing contractions increase protein S‐glutathionylation occupancy in mouse skeletal muscle. Redox Biol. 2018;17:367‐376. 29857311 10.1016/j.redox.2018.05.011 PMC6007084

[1383] Korac B , Kalezic A , Pekovic‐Vaughan V , Korac A , Jankovic A . Redox changes in obesity, metabolic syndrome, and diabetes. Redox Biol. 2021;42:101887. 33579666 10.1016/j.redox.2021.101887 PMC8113039

[1384] Ciciliot S , Fadini GP . Modulation of obesity and insulin resistance by the redox enzyme and adaptor protein p66(Shc). Int J Mol Sci. 2019;20(4):985. 30813483 10.3390/ijms20040985 PMC6412263

[1385] Zhang P , Li T , Wu X , Nice EC , Huang C , Zhang Y . Oxidative stress and diabetes: antioxidative strategies. Front Med. 2020;14(5):583‐600. 32248333 10.1007/s11684-019-0729-1

[1386] Kapahi P , Takahashi T , Natoli G , et al. Inhibition of NF‐kappa B activation by arsenite through reaction with a critical cysteine in the activation loop of Ikappa B kinase. J Biol Chem. 2000;275(46):36062‐36066. 10967126 10.1074/jbc.M007204200

[1387] Qian Q , Zhang Z , Orwig A , et al. S‐nitrosoglutathione reductase dysfunction contributes to obesity‐associated hepatic insulin resistance via regulating autophagy. Diabetes. 2018;67(2):193‐207. 29074597 10.2337/db17-0223 PMC10515702

[1388] Zhou HL , Premont RT , Stamler JS . The manifold roles of protein S‐nitrosylation in the life of insulin. Nat Rev Endocrinol. 2022;18(2):111‐128. 34789923 10.1038/s41574-021-00583-1 PMC8889587

[1389] Bahadoran Z , Mirmiran P , Ghasemi A . Role of nitric oxide in insulin secretion and glucose metabolism. Trends Endocrinol Metab. 2020;31(2):118‐130. 31690508 10.1016/j.tem.2019.10.001

[1390] Markwardt ML , Nkobena A , Ding SY , Rizzo MA . Association with nitric oxide synthase on insulin secretory granules regulates glucokinase protein levels. Mol Endocrinol. 2012;26(9):1617‐1629. 22771492 10.1210/me.2012-1183 PMC3434526

[1391] Hotamisligil GS . Endoplasmic reticulum stress and the inflammatory basis of metabolic disease. Cell. 2010;140(6):900‐917. 20303879 10.1016/j.cell.2010.02.034 PMC2887297

[1392] Choromanska B , Mysliwiec P , Dadan J , Maleckas A , Zalewska A , Maciejczyk M . Effects of age and gender on the redox homeostasis of morbidly obese people. Free Radic Biol Med. 2021;175:108‐120. 34390781 10.1016/j.freeradbiomed.2021.08.009

[1393] Lee HY , Lee GH , Yoon Y , Hoang TH , Chae HJ . IBF‐R regulates IRE1α post‐translational modifications and ER stress in high‐fat diet‐induced obese mice. Nutrients. 2022;14(1):217. 35011092 10.3390/nu14010217 PMC8746979

[1394] Oo SM , Oo HK , Takayama H , et al. Selenoprotein P‐mediated reductive stress impairs cold‐induced thermogenesis in brown fat. Cell Rep. 2022;38(13):110566. 35354056 10.1016/j.celrep.2022.110566

[1395] Chouchani ET , Kazak L , Jedrychowski MP , et al. Corrigendum: mitochondrial ROS regulate thermogenic energy expenditure and sulfenylation of UCP1. Nature. 2016;536(7616):360. 10.1038/nature18279 27281219

[1396] Sharma M , Gupta S , Singh K , et al. Association of glutathione‐S‐transferase with patients of type 2 diabetes mellitus with and without nephropathy. Diabetes Metab Syndr. 2016;10(4):194‐197. 27377684 10.1016/j.dsx.2016.06.006

[1397] Monzo‐Beltran L , Vazquez‐Tarragon A , Cerda C , et al. One‐year follow‐up of clinical, metabolic and oxidative stress profile of morbid obese patients after laparoscopic sleeve gastrectomy. 8‐oxo‐dG as a clinical marker. Redox Biol. 2017;12:389‐402. 28319890 10.1016/j.redox.2017.02.003 PMC5357674

[1398] Crunkhorn S . Cardiovascular disease: thioredoxin lowers hypertension. Nat Rev Drug Discov. 2017;16(4):240. 10.1038/nrd.2017.53 28356597

[1399] Tinkov AA , Bjorklund G , Skalny AV , et al. The role of the thioredoxin/thioredoxin reductase system in the metabolic syndrome: towards a possible prognostic marker? Cell Mol Life Sci. 2018;75(9):1567‐1586. 29327078 10.1007/s00018-018-2745-8 PMC11105605

[1400] Haldar SM , Stamler JS . S‐nitrosylation: integrator of cardiovascular performance and oxygen delivery. J Clin Invest. 2013;123(1):101‐110. 23281416 10.1172/JCI62854 PMC3533273

[1401] Lau B , Fazelinia H , Mohanty I , et al. Endogenous S‐nitrosocysteine proteomic inventories identify a core of proteins in heart metabolic pathways. Redox Biol. 2021;47:102153. 34610554 10.1016/j.redox.2021.102153 PMC8497991

[1402] Zhao S , Song TY , Wang ZY , et al. S‐nitrosylation of Hsp90 promotes cardiac hypertrophy in mice through GSK3β signaling. Acta Pharmacol Sin. 2022;43(8):1979‐1988. 34934196 10.1038/s41401-021-00828-9 PMC9343375

[1403] Tang X , Pan L , Zhao S , et al. SNO‐MLP (S‐nitrosylation of Muscle LIM Protein) facilitates myocardial hypertrophy through TLR3 (Toll‐Like Receptor 3)‐mediated RIP3 (Receptor‐Interacting Protein Kinase 3) and NLRP3 (NOD‐Like Receptor Pyrin Domain Containing 3) inflammasome activation. Circulation. 2020;141(12):984‐1000. 31902237 10.1161/CIRCULATIONAHA.119.042336

[1404] Zhang X , Zhang Y , Miao Q , et al. Inhibition of HSP90 S‐nitrosylation alleviates cardiac fibrosis via TGFbeta/SMAD3 signalling pathway. Br J Pharmacol. 2021;178(23):4608‐4625. 34265086 10.1111/bph.15626

[1405] Zhou M , Chen JY , Chao ML , et al. S‐nitrosylation of c‐Jun N‐terminal kinase mediates pressure overload‐induced cardiac dysfunction and fibrosis. Acta Pharmacol Sin. 2022;43(3):602‐612. 34011968 10.1038/s41401-021-00674-9 PMC8888706

[1406] Zhao S , Tang X , Miao Z , et al. Hsp90 S‐nitrosylation at Cys521, as a conformational switch, modulates cycling of Hsp90‐AHA1‐CDC37 chaperone machine to aggravate atherosclerosis. Redox Biol. 2022;52:102290. 35334246 10.1016/j.redox.2022.102290 PMC8942817

[1407] Chao ML , Luo S , Zhang C , et al. S‐nitrosylation‐mediated coupling of G‐protein alpha‐2 with CXCR5 induces Hippo/YAP‐dependent diabetes‐accelerated atherosclerosis. Nat Commun. 2021;12(1):4452. 34294713 10.1038/s41467-021-24736-y PMC8298471

[1408] Majumdar U , Manivannan S , Basu M , et al. Nitric oxide prevents aortic valve calcification by S‐nitrosylation of USP9X to activate NOTCH signaling. Sci Adv. 2021;7(6):eabe3706. 33547080 10.1126/sciadv.abe3706 PMC7864581

[1409] Valerio V , Keceli G , Moschetta D , et al. Enduring reactive oxygen species emission causes aberrant protein S‐glutathionylation transitioning human aortic valve cells from a sclerotic to a stenotic phenotype. Antioxid Redox Signal. 2022;37(13‐15):1051‐1071. 35459416 10.1089/ars.2021.0133 PMC9689771

[1410] Yang X , Ji Y , Wang W , et al. Amyotrophic lateral sclerosis: molecular mechanisms, biomarkers, and therapeutic strategies. Antioxidants (Basel). 2021;10(7):1012. 34202494 10.3390/antiox10071012 PMC8300638

[1411] Kim K . Glutathione in the nervoussystem as a potential therapeutic target to control the development and progression of amyotrophic lateral sclerosis. Antioxidants (Basel). 2021;10(7):1011. 34201812 10.3390/antiox10071011 PMC8300718

[1412] Nakamura T , Oh CK , Liao L , et al. Noncanonical transnitrosylation network contributes to synapse loss in Alzheimer's disease. Science. 2021;371(6526):eaaw0843. 33273062 10.1126/science.aaw0843 PMC8091809

[1413] Yang R , Gao Y , Li H , et al. Posttranslational S‐nitrosylation modification regulates HMGB1 secretion and promotes its proinflammatory and neurodegenerative effects. Cell Rep. 2022;40(11):111330. 36103834 10.1016/j.celrep.2022.111330 PMC9531316

[1414] Panda SP , Prasanth D , Gorla US , Dewanjee S . Interlinked role of ASN, TDP‐43 and Miro1 with parkinopathy: focus on targeted approach against neuropathy in parkinsonism. Ageing Res Rev. 2022:101783. 36371014 10.1016/j.arr.2022.101783

[1415] Yuan Y , Li H , Pu W , et al. Cancer metabolism and tumor microenvironment: fostering each other? Sci China Life Sci. 2022;65(2):236‐279. 34846643 10.1007/s11427-021-1999-2

[1416] Sies H , Jones DP . Reactive oxygen species (ROS) as pleiotropic physiological signalling agents. Nat Rev Mol Cell Biol. 2020;21(7):363‐383. 32231263 10.1038/s41580-020-0230-3

[1417] Hayes JD , Dinkova‐Kostova AT , Tew KD . Oxidative stress in cancer. Cancer Cell. 2020;38(2):167‐197. 32649885 10.1016/j.ccell.2020.06.001 PMC7439808

[1418] He Q , Qu M , Shen T , et al. Suppression of VEGFD expression by S‐nitrosylation promotes the development of lung adenocarcinoma. J Exp Clin Cancer Res. 2022;41(1):239. 35941690 10.1186/s13046-022-02453-8 PMC9358865

[1419] Zhao Q , Zheng K , Ma C , et al. PTPS facilitates compartmentalized LTBP1 S‐nitrosylation and promotes tumor growth under hypoxia. Mol Cell. 2020;77(1):95‐107. e5. 31628042 10.1016/j.molcel.2019.09.018

[1420] Chio IIC , Jafarnejad SM , Ponz‐Sarvise M , et al. NRF2 promotes tumor maintenance by modulating mRNA translation in pancreatic cancer. Cell. 2016;166(4):963‐976. 27477511 10.1016/j.cell.2016.06.056 PMC5234705

[1421] Bar‐Peled L , Kemper EK , Suciu RM , et al. Chemical proteomics identifies druggable vulnerabilities in a genetically defined cancer. Cell. 2017;171(3):696‐709. e23. 28965760 10.1016/j.cell.2017.08.051 PMC5728659

[1422] Li XX , Wang ZJ , Zheng Y , et al. Nuclear receptor Nur77 facilitates melanoma cell survival under metabolic stress by protecting fatty acid oxidation. Mol Cell. 2018;69(3):480‐492. e7. 29395065 10.1016/j.molcel.2018.01.001

[1423] Gao W , Huang M , Chen X , et al. The role of S‐nitrosylation of PFKM in regulation of glycolysis in ovarian cancer cells. Cell Death Dis. 2021;12(4):408. 33859186 10.1038/s41419-021-03681-0 PMC8050300

[1424] Kim B , Lee KJ . Activation of Nm23‐H1 to suppress breast cancer metastasis via redox regulation. Exp Mol Med. 2021;53(3):346‐357. 33753879 10.1038/s12276-021-00575-1 PMC8080780

[1425] Zhang Y , Park J , Han SJ , et al. Redox regulation of tumor suppressor PTEN in cell signaling. Redox Biol. 2020;34:101553. 32413744 10.1016/j.redox.2020.101553 PMC7226887

[1426] He D , Feng H , Sundberg B , et al. Methionine oxidation activates pyruvate kinase M2 to promote pancreatic cancer metastasis. Mol Cell. 2022;82(16):3045‐3060. e11. 35752173 10.1016/j.molcel.2022.06.005 PMC9391305

[1427] Cohen MS , Chang P . Insights into the biogenesis, function, and regulation of ADP‐ribosylation. Nat Chem Biol. 2018;14(3):236‐243. 29443986 10.1038/nchembio.2568 PMC5922452

[1428] Li P , Lei Y , Qi J , Liu W , Yao K . Functional roles of ADP‐ribosylation writers, readers and erasers. Front Cell Dev Biol. 2022;10:941356. 36035988 10.3389/fcell.2022.941356 PMC9404506

[1429] Rattan SI . Synthesis, modification and turnover of proteins during aging. Adv Exp Med Biol. 2010;694:1‐13. 20886752 10.1007/978-1-4419-7002-2_1

[1430] Gibson BA , Kraus WL . New insights into the molecular and cellular functions of poly(ADP‐ribose) and PARPs. Nat Rev Mol Cell Biol. 2012;13(7):411‐424. 22713970 10.1038/nrm3376

[1431] Liu C , Vyas A , Kassab MA , Singh AK , Yu X . The role of poly ADP‐ribosylation in the first wave of DNA damage response. Nucleic Acids Res. 2017;45(14):8129‐8141. 28854736 10.1093/nar/gkx565 PMC5737498

[1432] Li M , Yu X . Function of BRCA1 in the DNA damage response is mediated by ADP‐ribosylation. Cancer Cell. 2013;23(5):693‐704. 23680151 10.1016/j.ccr.2013.03.025 PMC3759356

[1433] Li Y , Liu C‐F , Rao G‐W . A review on poly (ADP‐ribose) polymerase (PARP) inhibitors and synthetic methodologies. Curr Med Chem. 2021;28(8):1565‐1584. 32164505 10.2174/0929867327666200312113011

[1434] Langelier M‐F , Zandarashvili L , Aguiar PM , Black BE , Pascal JM . NAD+ analog reveals PARP‐1 substrate‐blocking mechanism and allosteric communication from catalytic center to DNA‐binding domains. Nat Commun. 2018;9(1):844. 29487285 10.1038/s41467-018-03234-8 PMC5829251

[1435] Murai J , Huang S‐yN , Das BB , et al. Trapping of PARP1 and PARP2 by clinical PARP inhibitors. Cancer Res. 2012;72(21):5588‐5599. 23118055 10.1158/0008-5472.CAN-12-2753 PMC3528345

[1436] Huang H , Zhang D , Wang Y , et al. Lysine benzoylation is a histone mark regulated by SIRT2. Nat Commun. 2018;9(1):3374. 30154464 10.1038/s41467-018-05567-w PMC6113264

[1437] Brusilow SW , Danney M , Waber LJ , et al. Treatment of episodic hyperammonemia in children with inborn errors of urea synthesis. N Engl J Med. 1984;310(25):1630‐1634. 6427608 10.1056/NEJM198406213102503

[1438] Pu W‐R , An D‐Y , Wang Y , Zhang X , Huang Y‐P , Liu Z‐S . Improving identification of molecularly imprinted monolith to benzoylation modified peptides by a deep eutectic solvents monomer‐induced cooperation. Anal Chim Acta. 2022;1204:339697. 35397907 10.1016/j.aca.2022.339697

[1439] Harwood CS , Burchhardt G , Herrmann H , Fuchs G . Anaerobic metabolism of aromatic compounds via the benzoyl‐CoA pathway. Eur J Biochem. 1998;22(5):439‐458.

[1440] Wang D , Yan F , Wu P , et al. Global profiling of regulatory elements in the histone benzoylation pathway. Nat Commun. 2022;13(1):1369. 35296687 10.1038/s41467-022-29057-2 PMC8927147

[1441] Tan D , Wei W , Han Z , et al. HBO1 catalyzes lysine benzoylation in mammalian cells. iScience. 2022;25(11):105443. 36388951 10.1016/j.isci.2022.105443 PMC9647509

[1442] Ren X , Zhou Y , Xue Z , et al. Histone benzoylation serves as an epigenetic mark for DPF and YEATS family proteins. Nucleic Acids Res. 2021;49(1):114‐126. 33290558 10.1093/nar/gkaa1130 PMC7797077

[1443] Beezhold BL , Johnston CS , Fau ‐ Nochta KA , Nochta KA . Sodium benzoate‐rich beverage consumption is associated with increased reporting of ADHD symptoms in college students: a pilot investigation. J Atten Disord. 2014;18(1557‐1246 (Electronic)):236‐241. 22538314 10.1177/1087054712443156

[1444] Enchev RI , Schulman BA , Peter M . Protein neddylation: beyond cullin‐RING ligases. Nat Rev Mol Cell Biol. 2015;16(1471‐0080 (Electronic)):30‐44. 25531226 10.1038/nrm3919 PMC5131867

[1445] Li J , Zou J , Littlejohn R , Liu J , Su H . Neddylation, an emerging mechanism regulating cardiac development and function. Front Physiol. 2020;11:612927. 33391028 10.3389/fphys.2020.612927 PMC7773599

[1446] Zhou Q , Zheng Y , Sun Y . Neddylation regulation of mitochondrial structure and functions. Cell Biosci. 2021;11(1):55. 33731189 10.1186/s13578-021-00569-6 PMC7968265

[1447] Walden H , Podgorski MS , Fau ‐ Huang DT , Huang DT , Fau ‐ Miller DW , et al. The structure of the APPBP1‐UBA3‐NEDD8‐ATP complex reveals the basis for selective ubiquitin‐like protein activation by an E1. Mol Cell. 2003;12(1097‐2765 (Print)):1427‐1437. 14690597 10.1016/s1097-2765(03)00452-0

[1448] Huang DT , Paydar A , Zhuang M , Waddell MB , Holton JM , Schulman BA . Structural basis for recruitment of Ubc12 by an E2 binding domain in NEDD8's E1. Mol Cell. 2005;17(3):341‐350. 15694336 10.1016/j.molcel.2004.12.020

[1449] Zhou L , Zhang W , Sun Y , Jia L . Protein neddylation and its alterations in human cancers for targeted therapy. Cell Signal. 2018;44:92‐102. 29331584 10.1016/j.cellsig.2018.01.009 PMC5829022

[1450] Wu J‐T , Chan Y‐R , Chien C‐T . Protection of cullin‐RING E3 ligases by CSN‐UBP12. Trends Cell Biol. 2006;16(7):362‐369. 16762551 10.1016/j.tcb.2006.05.001

[1451] Li L , Wang M , Yu G , et al. Overactivated neddylation pathway as a therapeutic target in lung cancer. JNCI. 2014;106(6):dju083. 24853380 10.1093/jnci/dju083

[1452] Hammill JT , Scott DC , Min J , et al. Piperidinyl ureas chemically control defective in cullin neddylation 1 (DCN1)‐mediated cullin neddylation. J Med Chem. 2018;61(7):2680‐2693. 29547696 10.1021/acs.jmedchem.7b01277 PMC5898815

[1453] Chen P , Hu T , Liang Y , et al. Neddylation inhibition activates the extrinsic apoptosis pathway through ATF4–CHOP–DR5 axis in human esophageal cancer cells. Clin Cancer Res. 2016;22(16):4145‐4157. 26983464 10.1158/1078-0432.CCR-15-2254

[1454] Zhu J , Chu F , Zhang M , Sun W , Zhou F . Association between neddylation and immune response. Front Cell Dev Biol. 2022;10:890121. 35602593 10.3389/fcell.2022.890121 PMC9117624

[1455] Brownell JE , Sintchak MD , Gavin JM , et al. Substrate‐assisted inhibition of ubiquitin‐like protein‐activating enzymes: the NEDD8 E1 inhibitor MLN4924 forms a NEDD8‐AMP mimetic in situ. Mol Cell. 2010;37(1):102‐111. 20129059 10.1016/j.molcel.2009.12.024

[1456] He X , Zhu A , Feng J , Wang X . Role of neddylation in neurological development and diseases. Biotechnol Appl Biochem. 2022;69(1):330‐341. 33469954 10.1002/bab.2112

[1457] Liu K , Chen K , Zhang Q , et al. TRAF6 neddylation drives inflammatory arthritis by increasing NF‐κB activation. Lab Invest. 2019;99(4):528‐538. 30626891 10.1038/s41374-018-0175-8 PMC6484715

[1458] Le‐Trilling VTK , Megger DA , Katschinski B , et al. Broad and potent antiviral activity of the NAE inhibitor MLN4924. Sci Rep. 2016;6:19977. 26829401 10.1038/srep19977 PMC4734293

[1459] Hao R , Song Y , Li R , et al. MLN4924 protects against interleukin‐17A‐induced pulmonary inflammation by disrupting ACT1‐mediated signaling. Am J Physiol Lung Cell Mol Physiol. 2019;316(6):L1070‐L1080. 30892082 10.1152/ajplung.00349.2018

[1460] Cho WC . Proteomics technologies and challenges. Genomics Proteomics Bioinformatics. 2007;5(2):77‐85. 17893073 10.1016/S1672-0229(07)60018-7 PMC5054093

[1461] Burns J , Wilding CP , R LJ , P HH . Proteomic research in sarcomas ‐ current status and future opportunities. Semin Cancer Biol. 2020;61:56‐70. 31722230 10.1016/j.semcancer.2019.11.003 PMC7083238

[1462] Hendriks IA , D'Souza RC , Yang B , Verlaan‐de Vries M , Mann M , Vertegaal AC . Uncovering global SUMOylation signaling networks in a site‐specific manner. Nat Struct Mol Biol. 2014;21(10):927‐936. 25218447 10.1038/nsmb.2890 PMC4259010

[1463] Lumpkin RJ , Gu H , Zhu Y , et al. Site‐specific identification and quantitation of endogenous SUMO modifications under native conditions. Nat Commun. 2017;8(1):1171. 29079793 10.1038/s41467-017-01271-3 PMC5660086

[1464] Meyer JG , Kim S , Maltby DA , Ghassemian M , Bandeira N , Komives EA . Expanding proteome coverage with orthogonal‐specificity alpha‐lytic proteases. Mol Cell Proteomics. 2014;13(3):823‐835. 24425750 10.1074/mcp.M113.034710 PMC3945911

[1465] Pan S , Chen R . Pathological implication of protein post‐translational modifications in cancer. Mol Aspects Med. 2022;86:101097. 35400524 10.1016/j.mam.2022.101097 PMC9378605

[1466] Shi SP , Xu HD , Wen PP , Qiu JD . Progress and challenges in predicting protein methylation sites. Mol Biosyst. 2015;11(10):2610‐2619. 26080040 10.1039/c5mb00259a

[1467] Li Z , Wang B , Yu Q , Shi Y , Li L . 12‐Plex DiLeu isobaric labeling enabled high‐throughput investigation of citrullination alterations in the DNA damage response. Anal Chem. 2022;94(7):3074‐3081. 35129972 10.1021/acs.analchem.1c04073 PMC9055876

[1468] Burt RA , Dejanovic B , Peckham HJ , et al. Novel antibodies for the simple and efficient enrichment of native O‐GlcNAc modified peptides. Mol Cell Proteomics. 2021;20:100167. 34678516 10.1016/j.mcpro.2021.100167 PMC8605273

[1469] Avin A , Levy M , Porat Z , Abramson J . Quantitative analysis of protein‐protein interactions and post‐translational modifications in rare immune populations. Nat Commun. 2017;8(1):1524. 29142256 10.1038/s41467-017-01808-6 PMC5688095

[1470] Virág D , Dalmadi‐Kiss B , Vékey K , et al. Current trends in the analysis of post‐translational modifications. Chromatographia. 2019;83(1):1‐10.

[1471] Gonzalez‐Freire M , Semba RD , Ubaida‐Mohien C , et al. The Human Skeletal Muscle Proteome Project: a reappraisal of the current literature. J Cachexia Sarcopenia Muscle. 2017;8(1):5‐18. 27897395 10.1002/jcsm.12121 PMC5326819

[1472] Beltrao P , Bork P , Krogan NJ , van Noort V . Evolution and functional cross‐talk of protein post‐translational modifications. Mol Syst Biol. 2013;9:714. 24366814 10.1002/msb.201304521 PMC4019982

[1473] Soufi B , Soares NC , Ravikumar V , Macek B . Proteomics reveals evidence of cross‐talk between protein modifications in bacteria: focus on acetylation and phosphorylation. Curr Opin Microbiol. 2012;15(3):357‐363. 22633124 10.1016/j.mib.2012.05.003

[1474] Vu LD , Gevaert K , De Smet I . Protein language: post‐translational modifications talking to each other. Trends Plant Sci. 2018;23(12):1068‐1080. 30279071 10.1016/j.tplants.2018.09.004

[1475] van der Laarse SAM , Leney AC , Heck AJR . Crosstalk between phosphorylation and O‐GlcNAcylation: friend or foe. FEBS J. 2018;285(17):3152‐3167. 29717537 10.1111/febs.14491

[1476] Ali I , Ruiz DG , Ni Z , et al. Crosstalk between RNA Pol II C‐terminal domain acetylation and phosphorylation via RPRD proteins. Mol Cell. 2019;74(6):1164‐1174. e4. 31054975 10.1016/j.molcel.2019.04.008 PMC6588463

[1477] Leutert M , Entwisle SW , Villen J . Decoding post‐translational modification crosstalk with proteomics. Mol Cell Proteomics. 2021;20:100129. 34339852 10.1016/j.mcpro.2021.100129 PMC8430371

[1478] Nickerson JL , Baghalabadi V , Rajendran S , et al. Recent advances in top‐down proteome sample processing ahead of MS analysis. Mass Spectrom Rev. 2021. 10.1002/mas.21706 34047392

[1479] Rotilio D , Della Corte A , D'Imperio M , et al. Proteomics: bases for protein complexity understanding. Thromb Res. 2012;129(3):257‐262. 22283976 10.1016/j.thromres.2011.12.035

[1480] Wang D , Liu D , Yuchi J , et al. MusiteDeep: a deep‐learning based webserver for protein post‐translational modification site prediction and visualization. Nucleic Acids Res. 2020;48(W1):W140‐W146. 32324217 10.1093/nar/gkaa275 PMC7319475

[1481] Xu H , Wang Y , Lin S , et al. PTMD: a database of human disease‐associated post‐translational modifications. Genomics Proteomics Bioinformatics. 2018;16(4):244‐251. 30244175 10.1016/j.gpb.2018.06.004 PMC6205080

[1482] Huang KY , Lee TY , Kao HJ , et al. dbPTM in 2019: exploring disease association and cross‐talk of post‐translational modifications. Nucleic Acids Res. 2019;47(D1):D298‐D308. 30418626 10.1093/nar/gky1074 PMC6323979

[1483] Noujaim J , Payne LS , Judson I , Jones RL , Huang PH . Phosphoproteomics in translational research: a sarcoma perspective. Ann Oncol. 2016;27(5):787‐794. 26802162 10.1093/annonc/mdw030

[1484] Du Z , Lovly CM . Mechanisms of receptor tyrosine kinase activation in cancer. Mol Cancer. 2018;17(1):58. 29455648 10.1186/s12943-018-0782-4 PMC5817791

[1485] Drummond E , Pires G , MacMurray C , et al. Phosphorylated tau interactome in the human Alzheimer's disease brain. Brain. 2020;143(9):2803‐2817. 32812023 10.1093/brain/awaa223 PMC7526722

[1486] Chai X , Guo J , Dong R , et al. Quantitative acetylome analysis reveals histone modifications that may predict prognosis in hepatitis B‐related hepatocellular carcinoma. Clin Transl Med. 2021;11(3):e313. 33783990 10.1002/ctm2.313 PMC7939233

[1487] Zhang W , Yang Y , Lin L , et al. Comprehensive characterization of ubiquitinome of human colorectal cancer and identification of potential survival‐related ubiquitination. J Transl Med. 2022;20(1):445. 36184622 10.1186/s12967-022-03645-8 PMC9528151

[1488] Verhelst X , Dias AM , Colombel JF , et al. Protein glycosylation as a diagnostic and prognostic marker of chronic inflammatory gastrointestinal and liver diseases. Gastroenterology. 2020;158(1):95‐110. 31626754 10.1053/j.gastro.2019.08.060

[1489] Wang L‐B , Karpova A , Gritsenko MA , et al. Proteogenomic and metabolomic characterization of human glioblastoma. Cancer Cell. 2021;39(4):509‐528. 33577785 10.1016/j.ccell.2021.01.006 PMC8044053

[1490] Vasaikar SV , Straub P , Wang J , Zhang B . LinkedOmics: analyzing multi‐omics data within and across 32 cancer types. Nucleic Acids Res. 2018;46(D1):D956‐D963. 29136207 10.1093/nar/gkx1090 PMC5753188

[1491] Montaner J , Ramiro L , Simats A , et al. Multilevel omics for the discovery of biomarkers and therapeutic targets for stroke. Nat Rev Neurol. 2020;16(5):247‐264. 32322099 10.1038/s41582-020-0350-6

[1492] Scherer HU , van der Woude D , Toes REM . From risk to chronicity: evolution of autoreactive B cell and antibody responses in rheumatoid arthritis. Nat Rev Rheumatol. 2022;18(7):371‐383. 35606567 10.1038/s41584-022-00786-4

[1493] Xie X , van Delft MAM , Shuweihdi F , et al. Auto‐antibodies to post‐translationally modified proteins in osteoarthritis. Osteoarthritis Cartilage. 2021;29(6):924‐933. 33757859 10.1016/j.joca.2021.03.008

[1494] Kissel T , Reijm S , Slot LM , et al. Antibodies and B cells recognising citrullinated proteins display a broad cross‐reactivity towards other post‐translational modifications. Ann Rheum Dis. 2020;79(4):472‐480. 32041746 10.1136/annrheumdis-2019-216499

